# Influence of Substitution on the Interplay of Photochemical Switching and Phase Transitions in Azobenzene‐Based Molecular Materials

**DOI:** 10.1002/anie.8543657

**Published:** 2026-09-24

**Authors:** Conrad Averdunk, Hermann A. Wegner

**Affiliations:** ^1^ Center of Materials Research (LaMa), Institute of Organic Chemistry Justus Liebig University Giessen Germany

**Keywords:** azobenzene, molecular interactions, molecular solar thermal energy storage, phase change

## Abstract

Understanding and translating molecular‐scale switching of azobenzenes into a macroscopic, functional response in the solid state remains a challenge. Herein, the photochemical and thermal properties of different substituted *p*‐Ohexyl‐azobenzenes were systematically investigated to establish structure‐property relationships to optimize the performance of azobenzene (AB) based materials for energy storage applications. The influence of various alkyl substituents in different positions on the AB was screened to address the effect of increasing bulkiness on the multidimensional parameters required for efficient energy storage. For example, substituents in the *meta*‐ and *para*‐positions increased the isomerization enthalpy (∆*H*_iso._) due to attractive intermolecular interactions; 2,6‐F‐ and 2,6‐Me‐substitution induces an increased half‐life, however, it also a reduced isomerization enthalpy. Substitution in *ortho*‐ and *meta*‐positions leads to a suppressed crystallization process and ineffective energy release. Interestingly, it was demonstrated that even sterically demanding *para*‐substituents exhibit sharp crystallization behavior. Additionally, photoisomerization efficiency in the condensed state was investigated, which revealed a strong relation with the melting points (*T*_melt._) of the solid (*E*)‐isomers. These systematic findings connecting photochemistry, thermal properties, and phase change parameters will be a solid foundation for the rational development of new AB‐based materials also beyond energy storage applications.

## Graphical abstract

This study investigates the influence of several substituents at different positions on photochemical and thermal properties of azobenzene‐based materials to provide valuable structure‐property relationships for the specific design of azobenzene‐based materials for future energy storage and phase change materials.

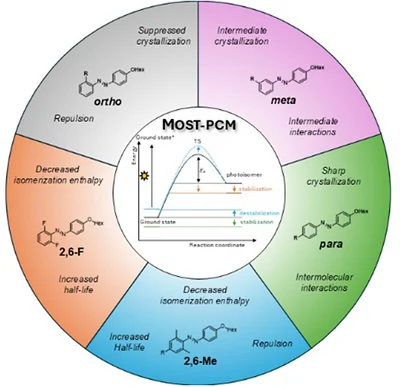

## 1 Introduction

Molecular photoswitches that interconvert reversibly between distinct states upon irradiation lie at the heart of modern efforts to design “smart” materials whose macroscopic properties can be controlled with light. In this respect, new, efficient, and environmentally friendly solar energy harvesting and storage systems based on photoswitches for a sustainable energy supply are of special interest [1]. Although a wide variety of systems have been developed over the past decades [2, 3], efficient and loss‐free storage of energy is still a major challenge. Molecular solar thermal (MOST) energy storage systems utilize photoswitches such as azobenzene (AB), norbornadiene (NBD) [4], dihydroazulenes (DHA) [5], or some more recent approaches such as azaborinine [6, 7], acetophenones [8], or pyrimidones [9] to process solar energy via photoisomerization of the ground state to a metastable, energetically excited state [10]. The stored energy can be released as thermal energy during back‐isomerization when triggered by an external stimulus. This process regenerates the ground state of the photoswitch, allowing the system to operate cyclically [11]. AB undergoes a reversible photoisomerization between the thermodynamically stable (*E*)‐isomer and the energetically excited (*Z*)‐isomer due to irradiation [12]. It has been investigated as a mostophore, a potential MOST system [13, 14, 15, 16, 17, 18, 19, 20, 21] as well as for other applications, including photopharmacology [22], functional materials [23, 24], or as a molecular wind‐up meter [25] to probe fundamental molecular interactions. However, ABs exhibit lower energy densities in MOST compared to some other mostophores. Nevertheless, their high chemical stability and relatively easy synthetic access are two major factors that contribute to the attractiveness of AB as a potential candidate for MOST systems [26]. Several attempts have been made to increase the energy storage density by using ABs in the condensed phase, as a solid [27, 28] or liquid state [29, 30, 31, 32, 33] switch. A recent approach to increase the energy storage density involves the combination of MOST with the concept of phase change materials (PCM) (Scheme 1) [34]. PCMs are capable of storing thermal energy through a reversible solid‐to‐liquid phase transition [35, 36, 37, 38].

**SCHEME 1:**
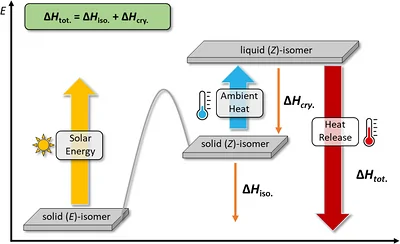
Energy diagram for simultaneous harvesting of photon and thermal energy and energy release.

AB is of particular interest for this approach, as it undergoes a significant geometry change upon photoisomerization compared to other photoswitches. This structural change has a substantial effect on the melting points of the respective (*E*)‐ and (*Z*)‐isomers, wherein the non‐planar (*Z*)‐isomers tend to have lower melting points than the corresponding planar (*E*)‐isomers [39]. The combination of the concepts of MOST and PCM enhances the overall energy storage density by coupling the photoisomerization from the (*E*)‐ to the (*Z*)‐isomer with a simultaneous solid‐to‐liquid phase transition. Consequently, this approach enables the storage of the isomerization enthalpy (∆*H*_iso._) with additional thermal energy in the form of crystallization enthalpy (∆*H*_cry._). The photoisomerization of pristine AB in the solid state is a limiting factor in the utilization of AB‐based MOST‐PCM systems, since the molecular mobility is usually significantly restricted due to strong π‐π‐interactions between AB moieties within the crystal structure [40, 41, 42, 43]. Therefore, photoisomerization is not feasible for all AB derivatives in the solid state. One strategy to improve the molecular mobility within the crystal structure is the introduction of alkyl chains, which exhibit greater conformational flexibility than the planar, conjugated AB core and reduce intermolecular interactions between AB units. Consequently, it increases structural freedom and decreases packing density within the crystal structure. In recent years, several AB‐based MOST‐PCM systems substituted with alkyl chains have been published, including various chains [44, 45, 46, 47, 48], heterocyclic AB [49, 50, 51, 52], different substituents [53, 54, 55, 56, 57, 58], or structural designs [59, 60]. Another possibility to increase the molecular mobility is the usage of AB in combination with organic PCM as a composite [61, 62, 63, 64].

The multidimensionality and interdependence of parameters result in the optimization process mostly in trial‐and‐error attempts. This study connects in a systematic fashion the properties of ABs in dilute solution to the molecular‐scale switching into a macroscopic, functional response in the solid state by addressing the influence of substitution and their positional effects on AB‐based MOST‐PCM systems. Methyl (Me), ethyl (Et), *iso*‐propyl (*i*Pr), and *tert*‐butyl (*t*Bu) groups were included to study the influence of the increasing steric demand as well as potential attractive London dispersion interactions [65, 66, 67, 68, 69] on properties such as melting points (*T*_melt._), crystallization temperature (*T*_cry._), glass transition temperature (*T*_glass._), melting (∆*H*_melt._) and crystallization enthalpies (∆*H*_cry._), the isomerization enthalpy (∆*H*_iso._), as well as the photoinduced liquefaction. In addition, the effect of symmetric and asymmetric substitution was investigated for *ortho‐* and *meta*‐substituted AB. In a previous study, the influence of alkyl chain length on *p*‐alkoxy‐AB was investigated [70]. It was found that an intermediate chain length exhibited the most favorable properties for MOST–PCM systems. Therefore, *p*‐OHex‐AB was used as the core structure for the present study. These findings provide a general design framework, complementary to existing tuning strategies mostly focused on electronic variations, for engineering AB‐based materials with tailored coupling between light‐driven molecular switching and macroscopic phase behavior, which go beyond MOST‐PCM applications.

## 2 Results and Discussion

### 2.1 Synthesis

For the synthesis of the substituted *p*‐OHex‐ABs **4**, the corresponding anilines **2** were converted into diazonium salts in a diazotization reaction (Scheme 2, conditions a). By subsequent addition of phenol under basic conditions, the substituted *p*‐OH‐ABs **3** were obtained via an azo coupling reaction. It should be mentioned that the yields of the azo coupling varied greatly depending on the aniline, which can be explained by solubility issues with increasing steric demand of the substituents, especially for *ortho*‐substituted anilines. Subsequently, all *p*‐OH‐ABs **3a‐r** were converted into the respective *p*‐OHex‐ABs **4a‐r** by Williamson etherification. An alternative synthesis method was required for the preparation of the *ortho*‐*t*Bu substituted *p*‐OH‐AB **3e**, as the initial method resulted exclusively in the formation of side products (Scheme 2, conditions b). Therefore, a Baeyer‐Mills reaction was used, in which 2‐*t*Bu‐aniline (**2e**) was converted to the nitroso compound using Oxone and then condensed with 4‐hydroxyaniline. The final molecule was again obtained by Williamson etherification with hexyl bromide.

**SCHEME 2:**
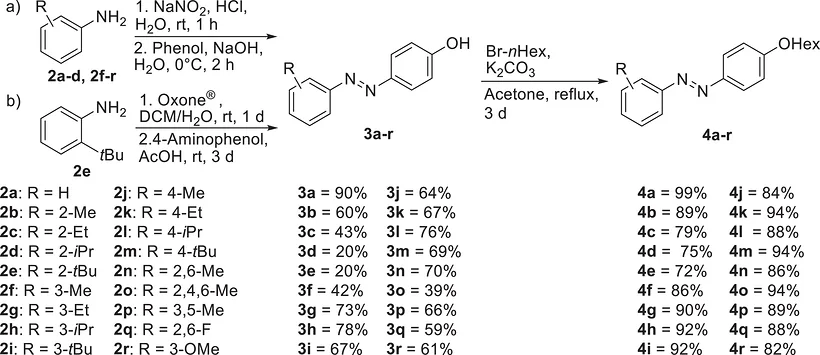
Synthesis of substituted *p*‐OHex‐ABs **4a‐r** via (a) azo coupling reaction and (b) via Baeyer‐Mills reaction followed by Williamson etherification.

### 2.2 Photochromic Properties in Solution

After the successful synthesis, all *p*‐OHex‐ABs **4a‐r** were investigated via UV‐Vis spectroscopy. Comparing the absorption maximum of the π–π* transition band of ABs **4b‐m** showed that all compounds experienced a red shift with the *ortho*‐ and *para*‐substituted ABs **4b‐e** and **4j‐m** being slightly stronger than *meta*‐substituted **4f‐i** (Table 1). This effect can be rationalized by the electron‐donating effect of the alkoxy chain in the *para*‐position [71]. Different wavelengths were tested to achieve the highest degree of photoisomerization. A wavelength of 365 nm gave the best results for the (*E*)‐ to the (*Z*)‐transition. The absorption spectrum of the (*Z*)‐isomer showed a minimum at 365 nm, which allows exclusively excitation of the (*E*)‐isomer, to mostly induce the (*E*)→(*Z*) isomerization. The absorption spectrum of the (*Z)*‐isomer of 2,6‐F‐AB **4q** exhibits a pronounced blue shift of the n–π* transition band, which is a well‐known characteristic of these AB derivatives [72, 73, 74]. A strong blue shift of the π–π* transition band was also observed for 2,6‐Me‐substituted ABs **4n** and **4o**, which is caused by a twisted geometry due to steric hindrance [75, 76]. Furthermore, the photostationary state (PSS) was determined for all *p*‐OHex‐ABs **4a‐r** with an average (*Z*)‐content of more than 90%. Interestingly, even sterically demanding substituents seem to have no significant influence on photoisomerisability, including substitution in the *ortho*‐position.

**TABLE 1:** Absorption maximum of the π–π* transition of the (*E*)‐isomers, content of (*Z*)‐isomer in the PSS_365_ in ACN, thermal half‐life in ACN at 20 °C, content of (*Z*)‐isomers in the PSS_365_ of the neat compounds after photoliquefication at rt, onset melting (*T*_melt_.) and crystallization temperature (*T*_cry._) of the (*E*)‐isomers and onset glass transition temperature (*T*_glass._) of the (*Z*)‐isomer.^a^

Entry	R	State rt	*λ*_max., ππ*_ [nm]	PSS_365, ACN_ (*Z*) [%]	Half‐life [h]	PSS_365, neat_ (*Z*) [%]	*T*_melt._ [°C]	*T*_cry._ [°C]	*T*_glass._ [°C]
**4a**	H	Solid	347	94.9 ± 0.2	74.8 ± 0.8	93.3	56.5	49.1	−60.2
**4b**	2‐Me	Solid	351	93.5 ± 0.2	120.7 ± 4.8	90.6	66.8	54.6	−58.6
**4c**	2‐Et	Liquid	351	95.1 ± 0.2	87.8 ± 1.4	92.5	31.4	−3.7	−61.0
**4d**	2‐*i*Pr	Solid	349	96.0 ± 0.3	97.7 ± 0.6	92.2	44.4	−31.8	−54.6
**4e**	2‐*t*Bu	Solid	345	94.7 ± 0.4	85.2 ± 3.5	90.0	54.6	15.0	−37.8
**4f**	3‐Me	Solid	347	94.4 ± 0.2	101.8 ± 4.1	90.8	50.8	37.4	−59.9
**4g**	3‐Et	Solid	348	95.8 ± 0.4	106.6 ± 0.1	90.0	44.2	23.1	−62.1
**4h**	3‐*i*Pr	Solid	349	95.2 ± 0.2	79.8 ± 1.1	92.3	36.7	5.9	−63.3
**4i**	3‐*t*Bu	Solid	347	94.7 ± 0.4	123.1 ± 0.3	92.1	54.5	3.5	−54.9
**4j**	4‐Me	Solid	349	96.8 ± 0.3	43.0 ± 1.0	87.3	77.0	61.0	−56.6
**4k**	4‐Et	Solid	350	96.4 ± 0.2	47.7 ± 0.3	89.3	64.6	43.8	−61.6
**4l**	4‐*i*Pr	Solid	350	95.4 ± 0.4	56.7 ± 0.1	86.3	92.7	75.1	−57.3
**4m**	4‐*t*Bu	Solid	351	96.4 ± 0.3	55.9 ± 0.3	88.9	116.6	94.9	−42.8
**4n**	2,6‐Me	Liquid	329	92.1 ± 0.3	722.5 ± 32.1^a^	90.7	25.1	10.0	−55.6
**4o**	2,4,6‐Me	Solid	335	93.5 ± 0.3	1008.9 ± 28.1^a^	85.1	59.5	51.7	−47.6
**4p**	3,5‐Me	Solid	350	94.7 ± 0.3	77.8 ± 1.6	87.5	57.9	26.7	−50.8
**4q**	2,6‐F	Liquid	341	93.5 ± 0.2	113.1 ± 3.0^a^	87.3	40.9	7.0	−59.8
**4r**	3‐OMe	Solid	351	95.4 ± 0.2	128.6 ± 2.1	83.1	47.0	31.4	−57.3

^a^ Thermal half‐life at 40 °C.

The thermal half‐life of each *p*‐OHex‐ABs **4a‐r** was determined via UV–Vis spectroscopy (Table 1). It was found that the average half‐life of *ortho*‐ and *meta*‐substituted ABs **4b‐i** is approximately 100 h and significantly shorter (about 50 h) for *para*‐substituted ABs **4j‐m**. Again, this result can be rationalized by the electron‐donating effect of the alkyl groups in the *para*‐position [77]. A significant exception is the 2,6‐substituted ABs **4n**, **4o** and **4q**, which show a drastically increased thermal half‐life. For the 2,6‐F‐AB **4q**, the (*Z*)‐isomer is stabilized due to the electron‐withdrawing effect of the fluorine atoms lowering the electronic repulsion of the lone pairs from the azo bond in the (*Z*)‐isomer [72, 73]. In the 2,6‐Me‐substituted ABs **4n** and **4o**, the transition state is destabilized due to the steric hindrance of the Me‐groups in the *ortho*‐position, leading to an increased activation energy for thermal back‐isomerization [78, 79, 80]. Therefore, substitution in the *ortho‐*position is an effective way to increase the optical and kinetic properties of AB‐based MOST‐PCM systems.

### 2.3 Photoliquefication Properties

All *p*‐OHex‐ABs **4a‐r** were successfully photoliquefied for their potential application as MOST‐PCM system by irradiating the neat solid compound with a wavelength of 365 nm at room temperature. The *p*‐OHex‐ABs **4c**, **4n,** and **4q** are liquid at room temperature. These also demonstrated successful photoliquefication by irradiating at temperatures below the melting points of the (*E*)‐isomers. Furthermore, the composition of the (*E*)‐ and (*Z*)‐ isomers in the photostationary states (PSS) of the photoliquefied AB were determined via HPLC analysis. At the PSS_365_ a content of about 83%–93% of the (*Z*)‐isomers was achieved. The process of reaching the PSS_365_ was the slowest for *p*‐*t*Bu‐AB **4m**. This compound revealed the highest values for *T*_melt._ of the (*E*)‐isomer and *T*_glass._ of the (*Z*)‐isomer, demonstrating that high melting points can result in less effective photoliquefication. Several effects must be considered during the irradiation of the solid‐state AB‐based MOST‐PCM. Initially, photoliquefication usually takes place at the surface of the compound as molecular movement is restricted [81]. However, light scattering and reflection effects from the surface can reduce the efficiency of the photoliquefication process [82]. Irradiation, specifically with UV light, suffers from a low penetration depth, which could result in incomplete photoisomerization of the bulk material [83]. Additionally, due to the spectral overlap of the (*E*)‐ and (*Z*)‐isomers, the penetration depth is expected to be significantly shorter. As photoisomerization progresses at the surface, melting is favored to occur on the surface first. The liquid phase on the surface can dissolve parts of the solid AB beneath, resulting in a mixture of (*E*)‐ and (*Z*)‐isomers resulting in a reduced *T*_melt_ [84]. To investigate the photoliquefication efficiency, crystalline thin films of several compounds were prepared by melting identical amounts of each compound between to glass slides to obtain evenly distributed surfaces. Each thin film was irradiated separately with 365 nm at room temperature with varying irradiation intervals. To gain a better understanding of the effect of the position of sterically demanding substituents on photoliquefication efficiency, the *o*‐, *m*‐ and *p*‐*t*Bu‐AB **4e**, **4i** and **4m** were investigated. Additionally, the unsubstituted AB **4a** and the 2,4,6‐Me‐AB **4o** were measured for enhanced comparability. These measurements reveal significant differences in the efficiency of photoliquefication process (Figure 1). The *o*‐ and *m*‐*t*Bu‐AB **4e** and **4i** showed a slightly higher photoliquefication efficiency than the unsubstituted AB **4a** indicating a minor influence of steric demanding substitutes in the *ortho*‐ and *meta*‐position. A significantly lower photoliquefication efficiency compared to the unsubstituted AB **4a** was observed for the 2,4,6‐Me‐AB **4o** and *p*‐*t*Bu‐AB **4m** indicating a stronger influence of substitution in the *para*‐position. However, a comparison of the *T*_melt._ of the (*E*)‐isomers shows a strong correlation, indicating a lower photoliquefication efficiency with increasing *T*_melt._ of the (*E*)‐isomers. The *T*_melt_ of *o*‐ and *m*‐*t*Bu‐AB **4e** and **4i** are almost identical, similar as the corresponding photoliquefication efficiencies. To strengthen this assumption *m*‐*i*Pr‐AB **4h**, which showed a significantly lower *T*_melt_, was investigated and showed to highest photoliquefication efficiency. To reach a content of the (*Z*)‐isomer of 50% irradiation of about 27 min. was necessary for *p*‐*t*Bu‐AB **4m**. In comparison, an irradiation time of only 1 min. was necessary to achieve a similar (*Z*)‐content for *m*‐*i*Pr‐AB **4h**. Since the *T*_melt._ correlated with the strength of intermolecular interactions, it potentially could be used as an indicator for the photoliquefication efficiency, as increasing intermolecular interactions could affect the photoisomerization in the solid or condensed phase.

**FIGURE 1:**
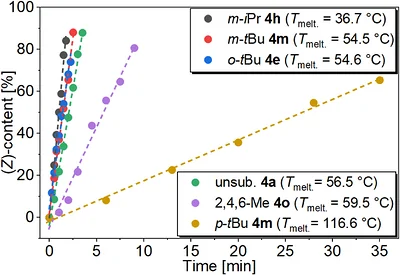
Change of the (*Z*)‐content during irradiation with 365 nm at room temperature of crystalline thin films for determination of the photoliquefication efficiency for selected AB.

### 2.4 Investigation of Thermodynamic Properties of (*E*)‐ and (*Z*)‐Isomers via DSC

An ideal MOST‐PCM system would feature a high *T*_melt._ of the (*E*)‐isomer and low *T*_cry._ or *T*_glass._ for the (*Z*)‐isomer, enabling the system to operate over a wide temperature range. However, sufficient photoliquefication can be reduced or even suppressed if the *T*_melt._ of the (*E*)‐ or (*Z*)‐isomers is too high. For the measurement of the thermodynamic properties the neat *p*‐OHex‐ABs **4a‐r** were irradiated at room temperature with 365 nm until full photoliquefication was accomplished. Twofold differential scanning calorimetry (DSC) measurements were performed in a temperature range from −90 °C to 180 °C under a nitrogen atmosphere with a heating and cooling rate of 10 K ∙ min^−1^ (Figure 2a). The composition of (*E*)‐ and (*Z*)‐isomers was determined via HPLC before each measurement. The photoliquefied *p*‐OHex‐ABs **4a‐r** were cooled from room temperature to −90 °C, held at this temperature for five minutes, and then heated to 180 °C. A glass transition was observed for each *p*‐OHex‐AB **4a‐r** in the first heating curve. The *o‐*, *m‐*, and *p*‐Me‐AB **4b, 4f, and 4j** showed a slight increase in the *T*_glass._ compared to unsubstituted AB **4a** (Figure S8). However, from Me to Et, a minor decrease was observed until the *T*_glass_ starts to increase again. The highest *T*_glass._ were observed for the *o‐*, *m‐*, and *p*‐*t*Bu‐AB **4e, 4i, and 4m**. Substitution in the *ortho*‐and *para*‐position showed the highest increase in *T*_glass._ indicating the strongest intermolecular interactions for the (*Z*)‐isomers, while substitution in the *meta*‐position had the lowest influence. In contrast, the 3,5‐Me‐AB **4p** showed a greater increase in *T*_glass._ than 2,6‐Me‐AB **4n**, while 2,4,6‐Me‐AB **4o** exhibited an even stronger increase in *T*_glass._ However, 2,6‐F‐AB **4q** shows an almost identical *T*_glass._ compared to the unsubstituted AB **4a** which could be caused by the absence of additional intermolecular interactions. That the interplay of different interactions with opposite effects might compensate each other out also needs to be considered. As the temperature further increased, the thermal back conversion from the (*Z*)‐isomer to the thermodynamically stable (*E*)‐isomer was induced. The onset temperature for *T*_iso._ remained for all *p*‐OHex‐ABs **4a‐r** in a similar temperature range between 65 °C and 75 °C. In comparison, all 2,6‐substituted ABs **4n**, **4o**, and **4q** showed a significantly higher *T*_iso._ of around 100 °C, which aligns with the highest thermal half‐life compared to the other derivatives. Consequently, the thermal half‐life can be considered an indicator of the temperature required for isomerization. Therefore, 2,6‐substituted ABs **4n**, **4o,** and **4q** are particularly attractive as MOST‐PCMs, as they enable long‐term energy storage over a broader temperature range. Usually, the *T*_iso._ of the (*Z*)‐isomer is higher than the *T*_melt._ of the (*E*)‐isomer. Consequently, thermal back‐isomerization generated the (*E*)‐isomer in its liquid state. An exception was *p*‐*t*Bu‐AB **4m**, which had a *T*_melt._ of about 116 °C. Therefore, it was the only derivative that undergoes thermal back‐isomerization and simultaneous crystallization of the (*E*)‐isomer (Figure 2a). However, *T*_iso._ is not an intrinsic material parameter because it depends significantly on the heating rate during the DSC measurement and therefore is more suitable as a relative rather than an absolute value. After completed thermal back‐isomerization, the liquid (*E*)‐isomer of the respective *p*‐OHex‐ABs **4a‐r** was cooled to −90 °C in order to determine *T*_cry._ afterwards. The thermograms showed that crystallization occurred not only during the cooling curve, but also during the subsequent heating cycle. This phenomenon was primarily observed for derivatives with substitutions in *ortho*‐position (Figure 2b). While for *o*‐Me‐AB **4b** a sharp crystallization signal was detected, the *o*‐Et‐AB **4c** did not completely crystallize during the cooling, and a weak crystallization was still observable while heating. For the *o*‐*i*Pr‐ and *o*‐*t*Bu‐AB **4d‐e**, crystallization was absent during cooling and was only observed during the heating phase. *m*‐Me‐ to *m*‐*i*Pr‐AB **4f‐h** exhibited a sharp crystallization signal, while for the *m*‐*t*Bu‐AB **4i** crystallization predominantly occurred during the heating curve. All *para*‐substituted ABs **4j‐m** showed a very sharp crystallization signal during the cooling curve indicating a favored crystallization process, probably due to higher symmetry. Consequently, substitution in the *para*‐position resulted in an increase in the *T*_cry._ from *p*‐Et‐ to *p*‐*t*Bu‐AB **4k‐m** while a decrease of the *T*_cry._ was observed for derivates substituted in *ortho*‐ and *meta*‐position. For almost all investigated *p*‐OHex‐AB the *T*_iso._ is higher than the *T*_cry._ leading to thermal back isomerization from the liquid (*Z*)‐ to the liquid (*E*)‐isomer. One exception was the *p*‐*t*Bu‐AB **4m** with a *T*_cry._ of about 95 °C which was in the same temperature range as the thermal back isomerization, which led to simultaneous crystallization during the thermal back isomerization of the liquid (*Z*)‐isomer to the solid (*E*)‐isomer followed by a subsequent melting of the (*E*)‐isomer (Figure 2a). The 2,4,6‐Me‐AB **4o** showed a significantly higher *T*_cry._ than the 2,6‐Me‐AB **4n** proving the favored crystallization due to *para*‐substitution. Subsequently, the *T*_melt._ of the *p*‐OHex‐AB **4a‐r** was determined as the heating curve proceeded. A decrease in *T*_melt._ was observed from Me‐ to Et‐substitution for all positions. For *ortho*‐ and *para*‐substitution a steady increase of the *T*_melt._ from Et to *t*Bu was measured. In contrast, for *meta*‐substitution, an increase was only present from *i*Pr to *t*Bu. Nevertheless, on average, *ortho*‐ and *meta*‐substituted AB **4b‐i** had lower *T*_melt._ while *para*‐substitution had a significant higher *T*_melt._ compared to the unsubstituted AB **4a** [85]. Furthermore, 2,6‐substituted *p*‐OHex‐ABs **4n** and **4q** had a significantly lower *T*_melt._ than the unsubstituted AB **4a**, with about 25°C for the 2,6‐Me‐AB **4n**. In contrast, the 2,4,6‐Me‐AB **4o** showed the highest *T*_melt._ among the 2,6‐substituted derivatives, demonstrating that additional *para*‐substitution can substantially increase the melting point. Comparing the observed state of 2‐*i*Pr‐AB **4c**, 2,6‐Me‐AB **4n,** and 2,6‐F‐AB **4q** with the corresponding *T*_melt._ shows that there is an inconsistency between the actual *T*_melt._ and state at room temperature. DSC analysis revealed that *T*_cry._ for those compounds is below room temperature. As only *ortho*‐substituted AB observed to be liquid at room temperature, *ortho*‐position probably decreased ability for π‐stacking due to twisted structure of the (*E*)‐isomer. Additionally, *T*_melt._ of 2‐*i*Pr‐AB **4c** and 2,6‐Me‐AB **4n** is close to room temperature. Therefore, asymmetric substitution and twisted structures favor supercooling and suppressed crystallization kinetics explain the observed discrepancy.

**FIGURE 2:**
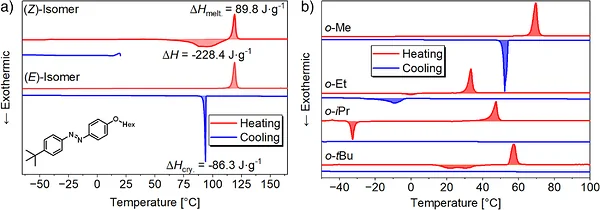
(a) DSC thermogram of the *p*‐*t*Bu‐AB **4m** from the photoliquefied state (top) and the (*E*)‐isomer (bottom) between −90 °C and 190 °C with a heating and cooling rate of 10 K∙min^−1^. (b) DSC thermogram of the *ortho*‐substituted AB **4b‐e** from the (*E*)‐isomer.

### 2.5 Investigation of Isomerization (∆*H*_iso._) and Crystallization Enthalpy (∆*H*_cry._) of (*E*)‐ and (*Z*)‐Isomers via DSC

DSC measurements were performed to determine the ∆*H*_iso._, ∆*H*_cry._ and ∆*H*_melt._ for each AB. The ∆*H*_iso._ of the photoliquefied *p*‐OHex‐AB **4a‐r** were obtained during the first heating cycle. After completing thermal back‐isomerization, ∆*H*_cry._ and ∆*H*_melt._ were determined in subsequent cooling and heating cycles, respectively (Figure 3). As ∆*H*_iso._ depends on the concentration of the (*Z*)‐isomer, the composition of the PSS_365_ was determined via HPLC before each measurement. For better comparability, the measured ∆*H*_iso._ were normalized to 100% of (*Z*)‐isomer concentration (Figure S14). The molar ∆*H*_iso._ of the *o*‐AB **4b‐e** showed constantly decreasing values compared to the unsubstituted AB **4a** (Figure 3a). In contrast, the molar ∆*H*_iso._ of the *meta*‐ and *para*‐substituted AB **4f‐m** were higher than the unsubstituted AB **4a**. The highest molar ∆*H*_iso._ was observed for *p*‐*t*Bu **4m**. For the 3,5‐Me‐AB **4p** and 3‐OMe‐AB **4r** similar molar ∆*H*_iso._ compared to the *meta*‐substituted AB **4f‐i** were observed. The high value for the *p*‐*t*Bu‐AB **4m** can be attributed to a stabilization of the (*E*)‐isomer or destabilization of the (*Z*)‐isomer. However, since the thermal half‐lives of all *para*‐substituted ABs **4j‐m** were nearly constant, stabilization of the (*Z*)‐isomer is unlikely. Therefore, it is reasonable that attractive interactions involving the *p*‐*t*Bu group lead to stabilization of the (*E*)‐isomer. Examination of the 2,6‐substituted ABs **4n**, **4o** and **4q** reveals the lowest molar ∆*H*_iso._, which could be attributed to a stabilization of the (*Z*)‐isomer for 2,6‐F‐AB **4q** and a destabilization of the (*E*)‐isomer for the 2,6‐Me‐substituted ABs **4n** and **4o** [86]. To enable more accurate comparisons between the thermal half‐life and the ∆*H*_iso_ of the mentioned compounds, it would be useful to determine the activation energy using Arrhenius analysis. However, as the kinetics differ substantially between the solution and neat state due to differences in intermolecular interactions, the activation parameters measured in solution are not directly comparable to those in the neat state [87]. Furthermore, when correlating the activation energy and the isomerization enthalpy, it should be noted that different isomerization mechanisms may occur during thermal relaxation in solution or in the neat state. Additionally, ∆*H*_cry._ and ∆*H*_melt._ were also determined via DSC. The *o*‐Me‐ **4b** and *p*‐Me‐AB **4j** showed a slight increase in ∆*H*_cry._ compared to the unsubstituted AB **4a**, which agrees with the corresponding *T*_melt._ (Figure 3b). However, a significant decrease was observed for *o*‐Et‐ and o‐*i*Pr‐AB **4c‐d**, followed by an increase for *o*‐*t*Bu‐AB **4e**. Similar trends were observed for *para*‐substitution, although an increase in ∆*H*_cry._ was already observed from the *o*‐*i*Pr‐AB **4d**. In contrast, substitution in the *meta*‐position results in a continuous decrease in ∆*H*_cry._ from *m*‐Me‐ to *m*‐*t*Bu‐AB **4f‐i**. Since crystallization and melting are a reversible process, the values for ∆*H*_cry._ and ∆*H*_melt._ should be identical. Comparing the values of ∆*H*_cry._ with ∆*H*_melt._ (Figure S12) show that ABs with suppressed crystallization behavior have a significantly reduced ∆*H*_cry._ The ∆*H*_cry._ of the *o*‐*i*Pr‐AB **4d** is about 50% lower than the corresponding ∆*H*_melt.,_ which is probably caused due to the broad signal for crystallization. In comparison, the ∆*H*_cry._ values for the ABs with sharp crystallization were almost identical to the corresponding values of ∆*H*_melt._, indicating that a sharp crystallization is necessary for an effective energy release.

**FIGURE 3:**
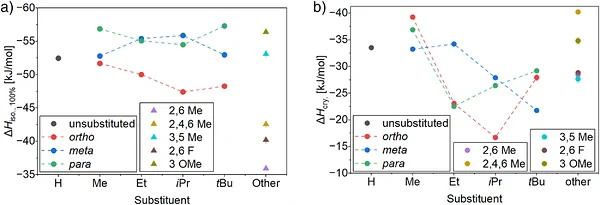
(a) Molar isomerization enthalpies (∆*H*_iso._) via DSC for 100% conversion. (b) Crystallization enthalpies (∆*H*_cry._).

A comparison of the solid state structures between the *m*‐*t*Bu‐AB **4i** and *p*‐*t*Bu‐AB **4m** shows that changing the *t*Bu‐group from *meta*‐ to the *para*‐position has a significant influence on the crystal packing (Figure 4). The unit cell of *m*‐*t*Bu‐AB **4i** exhibits two AB which are in plane with each *t*Bu‐group facing toward the hexyloxy‐chain. The unit cell of the *p*‐*t*Bu‐AB **4m** also contains two AB arranged in a head‐to‐tail structure. However, in this case, the AB are arranged almost orthogonally to each other, enabling London dispersion interactions as well as CH‐π interactions. A similar packing was observed for the *p*‐*i*Pr‐AB **4l**. This demonstrates the strong influence of the position of the substituents on intermolecular interactions, particularly within the crystal structure.

**FIGURE 4:**
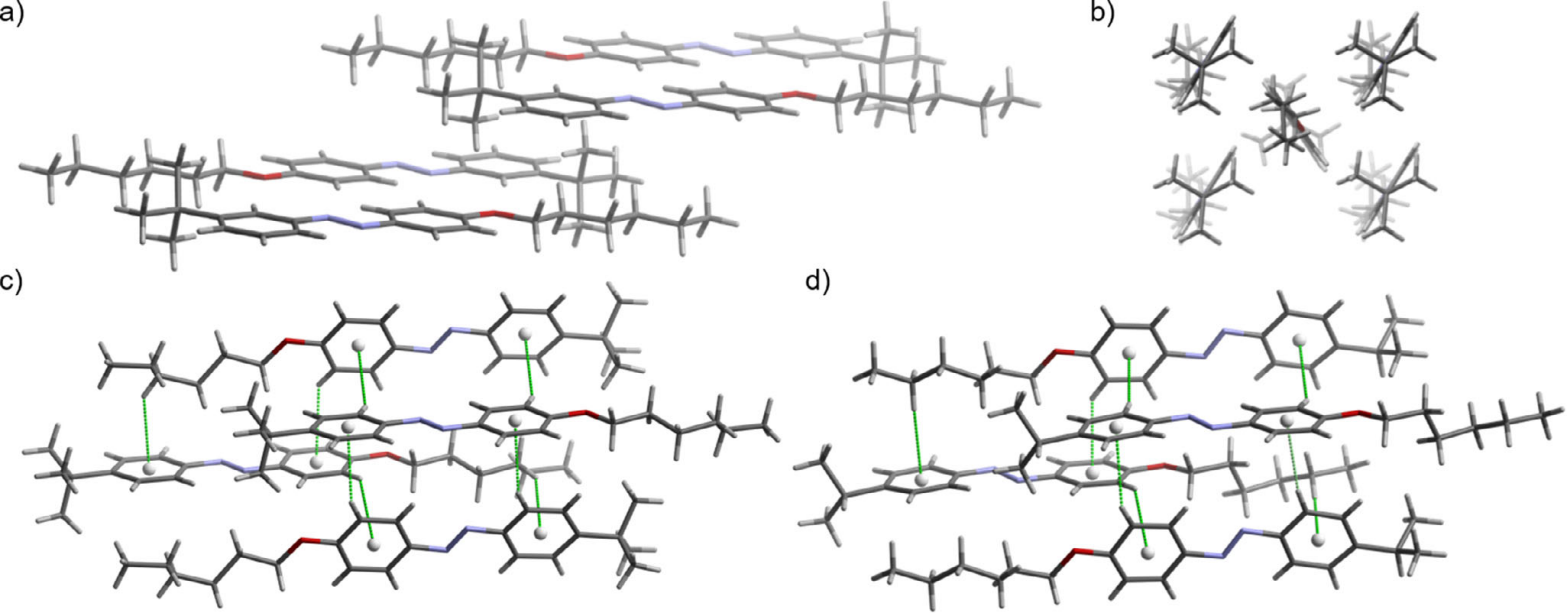
(a) SCXRD of *m*‐*t*Bu‐AB **4i**, (b) front view SCXRD of *p*‐*t*Bu‐AB **4m**, (c) side view of *p*‐*t*Bu‐AB **4m** and (d) SCXRD of *p*‐*i*Pr‐AB **4l**.

These results highlight the difficulty of predicting structure–property relationships in the condensed phase, especially for the crystal packing. For the *para*‐substitution specifically, a strong increase in *T*_melt._ was observed and suggests an increase in ∆*H*_cry._ but resulted in a decrease. Comparing ∆*H*_cry._ and ∆*H*_melt._ showed significant differences. For *ortho*‐substitution in particular, crystallization progressively shifted from the cooling curve to the subsequent heating curve as steric demand increased (Figure 2a). Among these derivatives, the *o*‐*i*Pr‐AB **4d** showed the greatest difference between ∆*H*_cry._ and ∆*H*_melt._ with almost 50 J∙g^−^^1^. The increasing steric demand in *ortho*‐ and *meta*‐substituted AB **4b‐i** significantly hindered the crystallization process. This can be attributed to a reduction in molecular symmetry.

## 3 Conclusion

In this systematic study, symmetric and asymmetric substituted *p*‐OHex‐ABs were successfully synthesized with various substituents in the *ortho*‐, *meta*‐, and *para*‐position. Photochromic properties, such as the absorption spectra, thermal half‐lives, and most efficient charging wavelength were obtained via UV‐Vis spectroscopy in solution. All compounds were successfully photoliquified by irradiation with 365 nm and could potentially be used as a MOST‐PCM system. The photoliquefication process was successfully quantified for selected compounds. For the development of an applicable MOST‐PCM system, efficient photoliquefication under visible light is beneficial. Although visible‐light photoliquefiable AB have already been described for tetra‐*ortho*‐halogenated AB [54, 58] and azopyrazoles [50, 52], this study provides valuable insights for improvement of future MOST‐PCM systems in general. Compounds with a higher *T*_melt._ of the (*E*)‐isomers were found to exhibit less efficient photoisomerization in the solid or condensed state. Furthermore, it was found that steric hindrance appears to have a weaker effect on photoliquefication efficiency. Since a higher *T*_melt._ can be associated with increased intermolecular interactions in the solid state, an increased *T*_melt._ hints at less effective photoisomerization in the solid state. Therefore, *T*_melt._ potentially could be used as an indicator for the efficiency of photoliquefication efficiency. The thermodynamic properties of the photoliquefied compounds and the corresponding (*E*)‐isomers were characterized via DSC measurements (Figure 5a). It has been shown that the molar ∆*H*_iso._ can be increased due to substitution in the *para*‐ and *meta*‐position, due to intermolecular interactions. The strongest influence was observed for the *p*‐*t*Bu‐AB **4m** with a ∆*H*_iso._ of almost 57.3 kJ∙mol^−^^1^ (Figure 4a). Additionally, all 2,6‐substituted ABs **4n**, **4o** and **4q** showed a decreased ∆*H*_iso._, which is probably caused by stabilization of the (*Z*)‐isomer of 2,6‐F‐AB **4q** or destabilization of the (*E*)‐isomer of 2,6‐Me‐ABs **4n** and **4o** [72]. Furthermore, it was shown that substitution with sterically demanding substituents in the *meta*‐ and especially *ortho*‐positions has a negative influence on phase change properties due to a suppressed crystallization process causing a decreased ∆*H*_cry._ and an ineffective energy release. For *para*‐substitution a sharp crystallization process was observed even for *p*‐*t*Bu‐AB **4m**. Furthermore, this compound exhibits the most pronounced temperature difference of all *p*‐OHex‐ABs **4a‐r** between the *T*_melt._ of the (*E*)‐isomer and *T*_glass._ of the (*Z*)‐isomer of almost 160°C. This property enables its utilization across a broad range of temperatures. These results demonstrate that predicting structure–property relationships is highly challenging, particularly in the condensed phase. Furthermore, it became evident that developing suitable MOST–PCM systems involves several underlying compromises. Increasing the thermal half‐life by stabilizing the (*Z*)‐isomer may lead to a reduced ∆*H*_iso._ (Figure 5b). However, this relationship is only applicable if attractive interactions predominantly contribute to a stabilization of the (Z)‐isomer, with minor impact on the transition state. Quantifying attractive interactions in the (*Z*)‐isomer using changes in thermal half‐life is already an established technique [25, 66]. Stabilization of the (*Z*)‐isomer, resulting in an increased thermal half‐life, has been demonstrated for tetra‐*ortho*‐halogenated [54] AB and heteroaryl‐AB that can form *T*‐shaped conformations, including azothiophenes [88, 89] and azopyrazoles [49]. Therefore, thermal half‐lives can be useful for estimating the influence on ∆*H*_iso._ for a series of MOST‐PCM candidates. An exception is 2,6‐Me‐ABs **4n** and **4o**, where the decreased ∆*H*_iso._ arises from destabilization of the (*E*)‐isomer and the increased thermal half‐life from destabilization of the transition state rather than (*Z*)‐isomer [80]. An alternative strategy could involve enhancing the crystallization enthalpy instead of the isomerization enthalpy to generate sufficiently high energy storage densities. These findings position steric substitution as a versatile design lever for AB‐based switchable materials, with implications reaching well beyond solar thermal energy storage.

**FIGURE 5:**
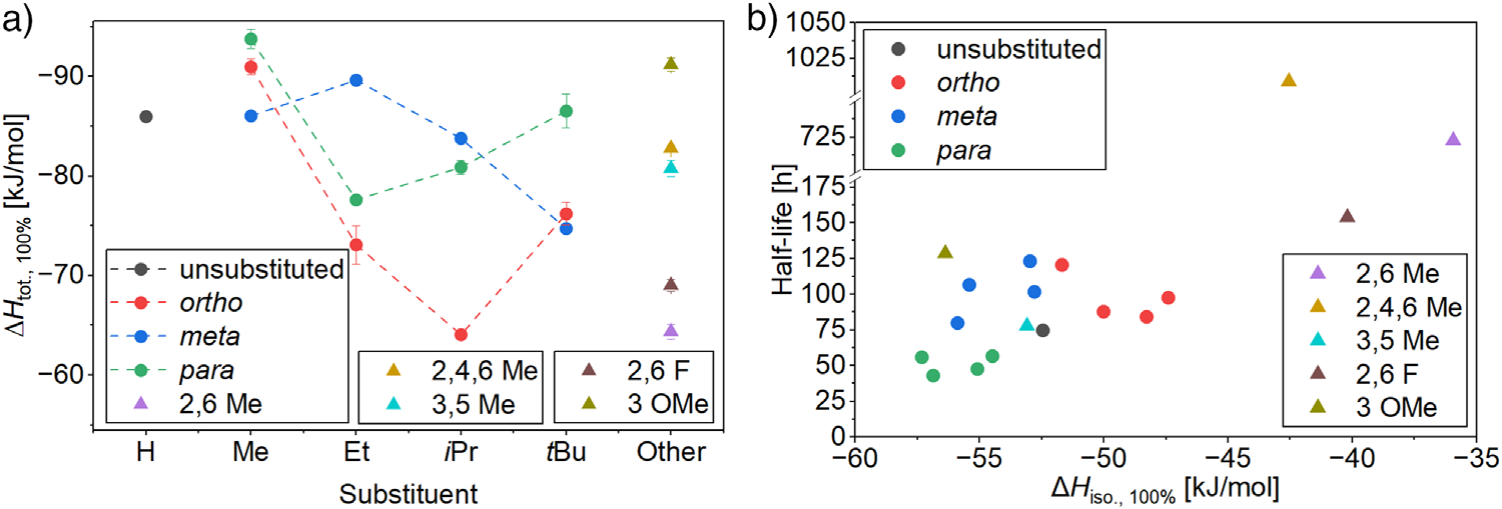
(a) Total storage enthalpy ∆*H*_tot._ for 100% conversion. (b) Isomerization enthalpy ∆*H*_iso._ versus thermal half‐life.

## Author Contributions

**Conrad Averdunk**: conceptualization, investigation, writing – original draft, methodology, validation, visualization, writing – review and editing, formal analysis, data curation. **Hermann A. Wegner**: conceptualization, funding acquisition, writing – original draft, visualization, writing – review and editing, formal analysis, project administration, resources, supervision.

## Conflicts of Interest

The authors declare no conflicts of interest.

## Supporting information




**Supporting File**: The authors have cited additional references within the Supporting Information [90, 91, 92, 93, 94, 95, 96, 97, 98, 99, 100, 101, 102].

## Data Availability

The data that support the findings of this study are available in the supplementary material of this article.
