# Supplementary material for: Influence of Substitution on the Interplay of Photochemical Switching and Phase Transitions in Azobenzene‐Based Molecular Materials
Source: Angew Chem Int Ed Engl. 2026 Sep 24;65(46):e8543657. doi: 10.1002/anie.8543657 (PMC13749359; doi:10.1002/anie.8543657)
Supplement: Supplementary file 1 [file ANIE-65-e8543657-s001.pdf]

Supporting Information - Influence of Substitution on the Interplay on Photochemical Switching and Phase Transitions in Azobenzene-Based Molecular Materials

Table of contents

1. General information 1
2. Synthesis 2
3. NMR spectra 24
4. Irradiation experiments 59
5. UV-Vis spectroscopy 60
6. Half-life in solution *via* UV-Vis spectroscopy 63
7. Determination of PSS in solution and neat *via* HPLC 66
8. DSC measurements 69
9. Photoliquefication efficiency 85
10. Solid state structures 87
11. References 114

1. General information

Chemicals were used as purchased from Sigma-Aldrich, Acros Organics, Alfa Aesar, TCI Europe or BLD Pharm. Technical grade solvents for workup and purification were distilled prior to use. Air and/or water-sensitive reactions were carried out under Schlenk-conditions or in a nitrogen-filled glovebox. Solids were dried under high vacuum (oil pump, ca 10^{-3} mbar) at room temperature (rt), 50 °C or 60 °C if necessary.

Flash column chromatography and column chromatography was carried out with Silica 60 M (0.04 – 0.063 mm) from Macherey Nagel GmbH & Co. KG. Thin layer chromatography was performed on Polygram® SIL G/UV254 from Macherey Nagel GmbH & Co. KG.

NMR spectra were measured on a Bruker Avance II 200 MHz, Avance II 400 MHz or Avance III 400 MHz HD spectrometer at rt, if not stated otherwise. Chemical shifts are reported in parts per million (ppm) relative to the solvent peak, coupling constants (*J*) are reported in Hertz (Hz). Deuterated solvents were obtained from Deutero GmbH (Kastellaun, Germany) or Euriso-Top GmbH.

Melting points were determined on a M5000 melting point meter from A. KRÜSS Optronic GmbH Germany. A heating rate of 1 °C min-1, a resolution of 0.1 °C and a measurement accuracy of ± 0.3 °C (25–200 °C) or ± 0,5 °C (200–400 °C) apply for this device.

ESI-MS spectra were recorded on a Bruker Daltonics Micro TOF. Melting points were measured on a Krüss M5000 capillary melting point meter with a heating rate of 1 °C min^{-1}.

2. Synthesis

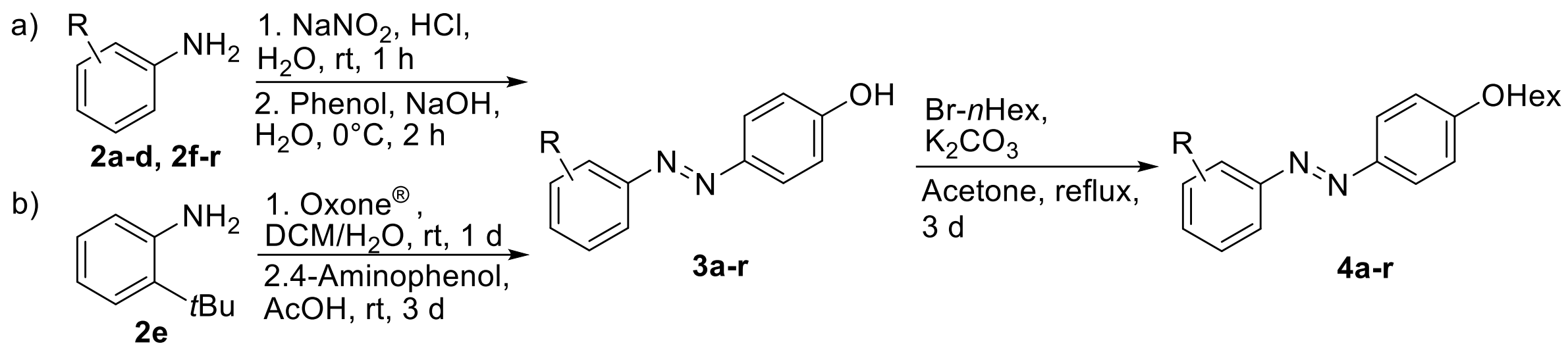

**2a**: R = H	**3a**: R = H; 90%	**4a**: R = H, 99%
**2b**: R = 2-Me	**3b**: R = 2-Me, 60%	**4b**: R = 2-Me, 89%
**2c**: R = 2-Et	**3c**: R = 2-Et, 43%	**4c**: R = 2-Et, 79%
**2d**:R = 2-*i*Pr	**3d**:R = 2-*i*Pr, 20%	**4d**: R = 2-*i*Pr, 75%
**2e**: R = 2-*t*Bu	**3e**: R = 2-*t*Bu, 20%	**4e**: R = 2-*t*Bu, 72%
**2f**: R = 3-Me	**3f**: R = 3-Me, 42%	**4f**:R = 3-Me, 86%
**2g**: R = 3-Et	**3g**: R = 3-Et, 73%	**4g**: R = 3-Et, 90%
**2h**: R = 3-*i*Pr	**3h**: R = 3-*i*Pro, 78%	**4h**: R = 3-*i*Pro, 92%
**2i**: R = 3-*t*Bu	**3i**: R = 3-*t*Bu, 67%	**4i**: R = 3-*t*Bu, 92%
**2j**: R = 4-Me	**3j**: R = 4-Me, 64%	**4j**: R = 4-Me, 84%
**2k**: R = 4-Et	**3k**: R = 4-Et, 67%	**4k**: R = 4-Et, 94%
**2l**: R = 4-*i*Pr	**3l**: R = 4-*i*Pr, 76%	**4l**: R = 4-*i*Pr, 88%
**2m**: R = 4-*t*Bu	**3m**: R = 4-*t*Bu, 69%	**4m**: R = 4-*t*Bu, 94%
**2n**: R = 2,6-Me	**3n**: R = 2,6-Me, 70%	**4n**: R = 2,6-Me, 86%
**2o**: R = 2,4,6-Me	**3o**: R = 2,4,6-Me,39%	**4o**: R = 2,4,6-Me, 94%
**2p**: R = 3,5-Me	**3p**: R = 3,5-Me, 66%	**4p**: R = 3,5-Me, 89%
**2q**: R = 2,6-F	**3q**: R = 2,6-F, 59%	**4q**: R = 2,6-F, 88%
**2r**: R = 3-OMe	**3r**: R = 3-OMe, 61%	**4r**: R = 3-OMe, 82%

Figure S1: Synthesis of substituted *p*-OHex-AB´s *via* a) azo coupling reaction and b) Beayer-Mills reaction followed by Williamson etherification.

Synthesis of 4-(phenyl)diazenylphenol (2a)

Aniline (2.20 mL 20.3 mmol, 1.00 eq.) was added to an aqueous solution containing H_2O (60 mL) and 37% HCl $_{(aq)}$. (10 mL) to prepare an aniline solution, which was added dropwise into an aqueous solution containing $NaNO_2$ (1.77 g, 25.7 mmol, 1.26 eq.) in H_2O (10 mL) cooled in an ice water bath (0 °C) under strong stirring. After mixing, the resulting solution was stirred for 1 h at 0 °C to form a diazonium salt solution. Then, a separated aqueous solution containing phenol (1.90 mL, 21.4 mmol, 1.05 eq.) and NaOH (8.09 g, 105 mmol, 10.2 eq.) in H_2O (40 mL) was prepared, and the diazonium salt solution was added to the phenol solution slowly with cooling by an ice water bath. The color of the solution became dark during this process. After stirring at 0 °C for 1 h,

the resulting solution was acidified by adding 20% $HCl_{(aq)}$ solution. Afterwards, the suspension was diluted with EtOAc (30 mL) and the phases were separated. The aqueous phase was extracted with EtOAc (3 x 20 mL) and the combined organic phases were dried over $MgSO_4$, filtered and the solvent removed under reduced pressure. The residue was purified by flash column chromatography (SiO_2, toluene) to yield an orange solid (3.87 g, 90%).

^{1}H NMR (400 MHz, $CDCl_3$) δ 7.98 – 7.86 (m, 4H), 7.54 – 7.48 (m, 2H), 7.47 – 7.42 (m, 1H), 7.03 – 6.94 (m, 2H). OH-signal not visible due to line broadening.

m.p. 147.8 °C

Analytical data corresponds to literature.[1]

Synthesis of 4-(2-methylphenyl)diazenylphenol (2b)

2-Methyl-aniline (2.20 mL 20.3 mmol, 1.00 eq.) was added to an aqueous solution containing H_2O (60 mL) and 37% HCl $_{(aq)}$. (10 mL) to prepare an aniline solution, which was added dropwise into an aqueous solution containing $NaNO_2$ (1.77 g, 25.7 mmol, 1.26 eq.) in H_2O (10 mL) cooled in an ice water bath (0 °C) under strong stirring. After mixing, the resulting solution was stirred for 1 h at 0 °C to form a diazonium salt solution. Then, a separated aqueous solution containing phenol (1.90 mL, 21.4 mmol, 1.05 eq.) and NaOH (8.09 g, 105 mmol, 10.2 eq.) in H_2O (40 mL) was prepared, and the diazonium salt solution was added to the phenol solution slowly with cooling by an ice water bath. The color of the solution became dark during this process. After stirring at 0 °C for 1 h, the resulting solution was acidified by adding 20% $HCl_{(aq)}$ solution. Afterwards, the suspension was diluted with EtOAc (30 mL) and the phases were separated. The aqueous phase was extracted with EtOAc (3 x 20 mL) and the combined organic phases were dried over $MgSO_4$, filtered and the solvent removed under reduced pressure. The residue was purified by flash column chromatography (SiO_2, toluene) to yield an orange solid (2.58 g, 60%).

^{1}H NMR (400 MHz, DMSO) δ 10.27 (s, 1H), 7.84 – 7.75 (m, 2H), 7.51 (dd, J = 7.7, 1.2 Hz, 1H), 7.43 – 7.36 (m, 2H), 7.34 – 7.24 (m, 1H), 6.98 – 6.90 (m, 2H), 2.63 (s, 3H).

^{13}C NMR (101 MHz, DMSO) δ 160.80, 150.05, 145.72, 136.72, 131.27, 130.34, 126.53, 124.85, 115.92, 115.00, 17.10.

HRMS (ESI): m/z for $C_{13}H_{13}N_2O^+$; calcd 213.1023, found 213.1023; m.p. 86.3 °C

Analytical data corresponds to literature.[2]

Synthesis of 4-(2-ethylphenyl)diazenylphenol (2c)

2-Ethyl-aniline (1.30 mL 10.3 mmol, 1.00 eq.) was added to an aqueous solution containing H_2O (30 mL) and 37% HCl $_{(aq)}$. (5 mL) to prepare an aniline solution, which was added dropwise into an aqueous solution containing $NaNO_2$ (927 mg, 13.4 mmol, 1.30 eq.) in H_2O (5 mL) cooled in an ice water bath (0 °C) under strong stirring. After mixing, the resulting solution was stirred for 1 h at 0 °C to form a diazonium salt solution. Then, a separated aqueous solution containing phenol (1.00 mL, 11.43mmol, 1.09 eq.) and NaOH (4.19 g, 105 mmol, 10.2 eq.) in H_2O (20 mL) was prepared, and the diazonium salt solution was added to the phenol solution slowly with cooling by an ice water bath. The color of the solution became dark during this process. After stirring at 0 °C for 1 h, the resulting solution was acidified by adding 20% $HCl_{(aq)}$ solution. Afterwards, the suspension was diluted with EtOAc (20 mL) and the phases were separated. The aqueous phase was extracted with EtOAc (3 x 10 mL) and the combined organic phases were dried over $MgSO_4$, filtered and the solvent removed under reduced pressure. The residue was purified by flash column chromatography (SiO_2, toluene) to yield a brown solid (790 mg, 43%).

^{1}H NMR (400 MHz, DMSO) δ 10.26 (s, 1H), 7.84 – 7.75 (m, 2H), 7.52 (dt, *J* = 8.0, 1.0 Hz, 1H), 7.43 – 7.39 (m, 2H), 7.33 – 7.26 (m, 1H), 6.98 – 6.91 (m, 2H), 3.07 (q, *J* = 7.5 Hz, 2H), 1.22 (t, *J* = 7.5 Hz, 3H).

^{13}C NMR (101 MHz, DMSO) δ 160.77, 149.50, 145.76, 142.73, 130.56, 129.83, 126.60, 124.78, 115.95, 114.91, 24.04, 16.45.

HRMS (ESI): m/z for $C_{14}H_{14}N_2ONa^+$; calcd 249.0998, found 249.0976; m.p. 92.8 °C

Synthesis of 4-(2-*iso*-propylphenyl)diazenylphenol (2d)

2-*Iso*-propyl-aniline (1.50 mL 10.1 mmol, 1.00 eq.) was added to an aqueous solution containing H_2O (30 mL) and 37% HCl $_{(aq)}$. (5 mL) to prepare an aniline solution, which was added dropwise into an aqueous solution containing $NaNO_2$ (910 mg, 13.2 mmol, 1.31 eq.) in H_2O (5 mL) cooled in an ice water bath (0 °C) under strong stirring. After mixing, the resulting solution was stirred for 1 h at 0 °C to form a diazonium salt solution. Then, a separated aqueous solution containing phenol (0.97 mL, 10.9 mmol, 1.08 eq.) and NaOH (4.10 g, 103 mmol, 10.2 eq.) in H_2O (20 mL) was prepared, and the diazonium salt solution was added to the phenol solution slowly with cooling by an ice water bath. The color of the solution became dark during this process. After stirring at 0 °C for 1 h, the resulting solution was acidified by adding 20% HCl $_{(aq)}$ solution.

Afterwards, the suspension was diluted with EtOAc (20 mL) and the phases were separated. The aqueous phase was extracted with EtOAc (3 x 10 mL) and the combined organic phases were dried over $MgSO_4$, filtered and the solvent removed under reduced pressure. The residue was purified by flash column chromatography (SiO_2, toluene) to yield a brown liquid (474 mg, 20%).

^1H NMR (400 MHz, DMSO) δ 10.26 (s, 1H), 7.83 – 7.75 (m, 2H), 7.49 (dd, J = 7.9, 1.5 Hz, 2H), 7.47 – 7.42 (m, 1H), 7.29 (ddd, J = 8.6, 7.1, 1.6 Hz, 1H), 6.99 – 6.91 (m, 2H), 4.03 – 3.93 (m, 1H), 1.28 (d, J = 7.0 Hz, 6H).

^{13}C NMR (101 MHz, DMSO) δ 160.76, 148.96, 146.55, 145.78, 130.66, 126.42, 126.36, 124.81, 115.96, 114.90, 27.26, 23.64.

HRMS (ESI): m/z for $C_{15}H_{17}N_2O^+$; calcd 241.1335, found 241.1316

Synthesis of 4-(2-*tert*-butylphenyl)diazenylphenol (2e)

2-*Tert*-butyl-aniline (1.60 mL 1.02 mmol, 1.00 eq.) was dissolved in DCM (25 mL). A solution of Oxone® (6.81 g, 12.2 mmol, 1.22 eq.) in water (25 mL) was added. The reaction mixture was stirred at rt overnight under nitrogen atmosphere. The reaction mixture was separated and extracted with DCM (3 x 10 mL). The combined organic phases were dried over $MgSO_4$ and filtered and the solvent removed under reduced pressure. The residue was dissolved in acetic acid (50 mL) and 4-Aminophenol (0.80 mL, 9.34 mmol, 0.92 eq.) was added. The reaction mixture was stirred for 3 day under nitrogen atmosphere. The reaction mixture was extracted with EtOAc (3 x 20 mL) and the combined organic phases were dried over $MgSO_4$, filtered and the solvent removed under reduced pressure. The residue was purified by flash column chromatography (SiO_2, toluene: EtOAc; 10:1) to yield a dark red liquid (527 mg, 20%).

^1H NMR (400 MHz, $CDCl_3$) δ 7.91 – 7.83 (m, 2H), 7.50 (ddd, J = 9.6, 7.9, 1.5 Hz, 2H), 7.39 – 7.33 (m, 1H), 7.30 – 7.26 (m, 1H), 7.00 – 6.92 (m, 2H), 1.53 (s, 9H).

^{13}C NMR (101 MHz, $CDCl_3$) δ 158.16, 151.98, 147.69, 147.55, 130.07, 126.82, 126.58, 125.43, 116.71, 116.00, 36.19, 32.05.

HRMS (ESI): m/z for $C_{16}H_{18}N_2ONa^+$; calcd 277.1311, found 277.1310

Synthesis of 4-(3-methylphenyl)diazenylphenol (2f)

3-Methyl-Aniline (2.20 mL 21.4 mmol, 1.00 eq.) was added to an aqueous solution containing H_2O (60 mL) and 37% HCl $_{(aq)}$. (10 mL) to prepare an aniline solution, which was added dropwise into an aqueous solution containing $NaNO_2$ (1.72 g, 24.9 mmol,

1.23 eq.) in H_2O (10 mL) cooled in an ice water bath (0 °C) under strong stirring. After mixing, the resulting solution was stirred for 1 h at 0 °C to form a diazonium salt solution. Then, a separated aqueous solution containing phenol (1.90 mL, 21.4 mmol, 1.05 eq.) and NaOH (8.05 g, 201 mmol, 9.90 eq.) in H_2O (40 mL) was prepared, and the diazonium salt solution was added to the phenol solution slowly with cooling by an ice water bath. The color of the solution became dark during this process. After stirring at 0 °C for 1 h, the resulting solution was acidified by adding 20% $HCl_{(aq)}$ solution. Afterwards, the suspension was diluted with EtOAc (30 mL) and the phases were separated. The aqueous phase was extracted with EtOAc (3 x 20 mL) and the combined organic phases were dried over $MgSO_4$, filtered and the solvent removed under reduced pressure. The residue was purified by flash column chromatography (SiO_2, toluene) to yield an orange solid (1.81 g, 42%).

^{1}H NMR (400 MHz, DMSO) δ 10.29 (s, 1H), 7.83 – 7.74 (m, 2H), 7.68 – 7.56 (m, 2H), 7.47 – 7.39 (m, 1H), 7.34 – 7.27 (m, 1H), 6.98 – 6.90 (m, 2H), 2.40 (s, 3H).

^{13}C NMR (101 MHz, DMSO) δ 160.88, 152.16, 145.24, 138.77, 131.12, 129.12, 124.77, 122.16, 119.75, 115.92, 20.93.

HRMS (ESI): m/z for $C_{13}H_{13}N_2O^+$; calcd 213.1023, found 213.1025; m.p. 159.3 °C

Analytical data corresponds to literature.[3]

Synthesis of 4-(3-ethylphenyl)diazenylphenol (2g)

3-Ethyl-aniline (1.30 mL 10.4 mmol, 1.00 eq.) was added to an aqueous solution containing H_2O (30 mL) and 37% HCl $_{(aq)}$. (5 mL) to prepare an aniline solution, which was added dropwise into an aqueous solution containing $NaNO_2$ (899 mg, 13.0 mmol, 1.25 eq.) in H_2O (5 mL) cooled in an ice water bath (0 °C) under strong stirring. After mixing, the resulting solution was stirred for 1 h at 0 °C to form a diazonium salt solution. Then, a separated aqueous solution containing phenol (1.02 mL, 11.4 mmol, 1.10 eq.) and NaOH (4.23 g, 103 mmol, 10.2 eq.) in H_2O (20 mL) was prepared, and the diazonium salt solution was added to the phenol solution slowly with cooling by an ice water bath. The color of the solution became dark during this process. After stirring at 0 °C for 1 h, the resulting solution was acidified by adding 20% $HCl_{(aq)}$ solution. Afterwards, the suspension was diluted with EtOAc (20 mL) and the phases were separated. The aqueous phase was extracted with EtOAc (3 x 10 mL) and the combined organic phases were dried over $MgSO_4$, filtered and the solvent removed

under reduced pressure. The residue was purified by flash column chromatography (SiO_2, toluene) to yield an orange solid (1.72 g, 73%).

^{1}H NMR (400 MHz, DMSO) δ 10.28 (s, 1H), 7.84 – 7.75 (m, 2H), 7.68 – 7.59 (m, 2H), 7.46 (t, *J* = 7.7 Hz, 1H), 7.34 (dt, *J* = 7.7, 1.4 Hz, 1H), 6.98 – 6.90 (m, 2H), 2.71 (q, *J* = 7.6 Hz, 2H), 1.23 (t, *J* = 7.6 Hz, 3H).

^{13}C NMR (101 MHz, DMSO) δ 160.86, 152.25, 145.24, 145.07, 129.99, 129.20, 124.76, 121.19, 119.75, 115.91, 28.03, 15.50.

HRMS (ESI): m/z for $C_{14}H_{14}N_2ONa^+$; calcd 249.0998, found 249.0976; m.p. 75.2°C

Synthesis of 4-(3-*iso*-propylphenyl)diazenylphenol (2h)

3-*Iso*-propyl-aniline (1.40 mL 9.94 mmol, 1.00 eq.) was added to an aqueous solution containing H_2O (30 mL) and 37% HCl $_{(aq)}$. (5 mL) to prepare an aniline solution, which was added dropwise into an aqueous solution containing $NaNO_2$ (831 mg, 12.0 mmol, 1.21 eq.) in H_2O (5 mL) cooled in an ice water bath (0 °C) under strong stirring. After mixing, the resulting solution was stirred for 1 h at 0 °C to form a diazonium salt solution. Then, a separated aqueous solution containing phenol (1.00 mL, 11.4 mmol, 1.14 eq.) and NaOH (4.11 g, 103 mmol, 10.3 eq.) in H_2O (20 mL) was prepared, and the diazonium salt solution was added to the phenol solution slowly with cooling by an ice water bath. The color of the solution became dark during this process. After stirring at 0 °C for 1 h, the resulting solution was acidified by adding 20% $HCl_{(aq)}$ solution. Afterwards, the suspension was diluted with EtOAc (20 mL) and the phases were separated. The aqueous phase was extracted with EtOAc (3 x 10 mL) and the combined organic phases were dried over $MgSO_4$, filtered and the solvent removed under reduced pressure. The residue was purified by flash column chromatography (SiO_2, toluene) to yield an orange solid (1.86 g, 78%).

^{1}H NMR (400 MHz, DMSO) δ 10.28 (s, 1H), 7.84 – 7.76 (m, 2H), 7.69 (t, *J* = 1.9 Hz, 1H), 7.62 (ddd, *J* = 7.8, 2.0, 1.2 Hz, 1H), 7.46 (t, *J* = 7.7 Hz, 1H), 7.38 (dt, *J* = 7.6, 1.6 Hz, 1H), 6.98 – 6.90 (m, 2H), 3.00 (hept, *J* = 6.9 Hz, 1H), 1.26 (d, *J* = 6.9 Hz, 6H).

^{13}C NMR (101 MHz, DMSO) δ 160.87, 152.26, 149.71, 145.24, 129.23, 128.60, 124.77, 120.06, 119.58, 115.91, 33.35, 23.78.

HRMS (ESI): m/z for $C_{16}H_{17}N_2O^+$; calcd 241.1335, found 241.1336; m.p. 97.3 °C

Analytical data corresponds to literature.[4]

Synthesis of 4-(3-*tert*-butylphenyl)diazenylphenol (2i)

3-*Tert*-butyl-aniline (1.40 mL 9.92 mmol, 1.00 eq.) was added to an aqueous solution containing H_2O (30 mL) and 37% HCl $_{(aq)}$. (5 mL) to prepare an aniline solution, which was added dropwise into an aqueous solution containing $NaNO_2$ (830 mg, 12.0 mmol, 1.21 eq.) in H_2O (5 mL) cooled in an ice water bath (0 °C) under strong stirring. After mixing, the resulting solution was stirred for 1 h at 0 °C to form a diazonium salt solution. Then, a separated aqueous solution containing phenol (1.00 mL, 1.14 mmol, 1.15 eq.) and NaOH (4.01 g, 100 mmol, 10.1 eq.) in H_2O (20 mL) was prepared, and the diazonium salt solution was added to the phenol solution slowly with cooling by an ice water bath. The color of the solution became dark during this process. After stirring at 0 °C for 1 h, the resulting solution was acidified by adding 20% HCl $_{(aq)}$ solution. Afterwards, the suspension was diluted with EtOAc (20 mL) and the phases were separated. The aqueous phase was extracted with EtOAc (3 x 10 mL) and the combined organic phases were dried over $MgSO_4$, filtered and the solvent removed under reduced pressure. The residue was purified by flash column chromatography (SiO_2, toluene) to yield an orange solid (1.70 g, 67%).

^{1}H NMR (400 MHz, DMSO) δ 10.29 (s, 1H), 7.84 – 7.78 (m, 3H), 7.61 (ddd, *J* = 7.7, 1.9, 1.2 Hz, 1H), 7.54 (ddd, *J* = 7.8, 2.0, 1.2 Hz, 1H), 7.47 (t, *J* = 7.8 Hz, 1H), 6.98 – 6.89 (m, 2H), 1.34 (s, 9H).

^{13}C NMR (101 MHz, DMSO) δ 160.85, 152.07, 152.04, 145.26, 129.03, 127.53, 124.80, 119.65, 118.62, 115.91, 34.60, 31.07.

HRMS (ESI): m/z for $C_{16}H_{19}N_2O^+$; calcd 255.1492, found 255.1488; m.p. 113.4 °C

Analytical data corresponds to literature.

Synthesis of 4-(4-methylphenyl)diazenylphenol (2j)

4-Methyl-aniline (2.10 mL 20.4 mmol, 1.00 eq.) was added to an aqueous solution containing H_2O (60 mL) and 37% HCl $_{(aq)}$. (10 mL) to prepare an aniline solution, which was added dropwise into an aqueous solution containing $NaNO_2$ (1.77 g, 25.7 mmol, 1.26 eq.) in H_2O (10 mL) cooled in an ice water bath (0 °C) under strong stirring. After mixing, the resulting solution was stirred for 1 h at 0 °C to form a diazonium salt solution. Then, a separated aqueous solution containing phenol (1.90 mL, 21.4 mmol, 1.05 eq.) and NaOH (8.21 g, 20.5 mmol, 10.1 eq.) in H_2O (40 mL) was prepared, and the diazonium salt solution was added to the phenol solution slowly with cooling by an ice water bath. The color of the solution became dark during this process. After stirring at

0 °C for 1 h, the resulting solution was acidified by adding 20% $HCl_{(aq)}$ solution. Afterwards, the suspension was diluted with EtOAc (30 mL) and the phases were separated. The aqueous phase was extracted with EtOAc (3 x 20 mL) and the combined organic phases were dried over $MgSO_4$, filtered and the solvent removed under reduced pressure. The residue was purified by flash column chromatography (SiO_2, toluene) to yield an orange solid (2.74 g, 64%).

^{1}H NMR (400 MHz, DMSO) δ 10.25 (s, 1H), 7.77 (d, *J* = 8.8 Hz, 2H), 7.72 (d, *J* = 8.3 Hz, 2H), 7.35 (d, *J* = 7.9 Hz, 2H), 6.93 (d, *J* = 8.9 Hz, 2H), 2.38 (s, 3H).

^{13}C NMR (101 MHz, DMSO) δ 160.68, 150.15, 145.21, 140.51, 129.83, 124.65, 122.11, 115.89, 20.96.

HRMS (ESI): m/z for $C_{13}H_{13}N_2O^+$; calcd 213.1023, found 213.1023; m.p. 137.2 °C

Analytical data corresponds to literature.[1]

Synthesis of 4-(4-ethylphenyl)diazenylphenol (2k)

4-Ethyl-aniline (1.30 mL 10.4 mmol, 1.00 eq.) was added to an aqueous solution containing H_2O (30 mL) and 37% HCl $_{(aq)}$. (5 mL) to prepare an aniline solution, which was added dropwise into an aqueous solution containing $NaNO_2$ (898 mg, 13.0 mmol, 1.26 eq.) in H_2O (5 mL) cooled in an ice water bath (0 °C) under strong stirring. After mixing, the resulting solution was stirred for 1 h at 0 °C to form a diazonium salt solution. Then, a separated aqueous solution containing phenol (1.00 mL, 11.3 mmol, 1.09 eq.) and NaOH (3.89 g, 97.3 mmol, 9.39 eq.) in H_2O (20 mL) was prepared, and the diazonium salt solution was added to the phenol solution slowly with cooling by an ice water bath. The color of the solution became dark during this process. After stirring at 0 °C for 1 h, the resulting solution was acidified by adding 20% $HCl_{(aq)}$ solution. Afterwards, the suspension was diluted with EtOAc (20 mL) and the phases were separated. The aqueous phase was extracted with EtOAc (3 x 10 mL) and the combined organic phases were dried over $MgSO_4$, filtered and the solvent removed under reduced pressure. The residue was purified by flash column chromatography (SiO_2, toluene) to yield an orange solid (1.56 g, 67%).

^{1}H NMR (400 MHz, DMSO) δ 10.25 (s, 1H), 7.82 – 7.70 (m, 4H), 7.39 (d, *J* = 8.6 Hz, 2H), 6.93 (d, *J* = 8.9 Hz, 2H), 2.69 (q, *J* = 7.6 Hz, 2H), 1.22 (t, *J* = 7.6 Hz, 3H).

^{13}C NMR (101 MHz, DMSO) δ 160.68, 150.34, 146.67, 145.22, 128.64, 124.65, 122.18, 115.88, 28.00, 15.37.

HRMS (ESI): m/z for $C_{14}H_{14}N_2ONa^+$; calcd 249.0998, found 249.0996; m.p. 71.7 °C

Analytical data corresponds to literature.[1]

Synthesis of 4-(4-*iso*-propylphenyl)diazenylphenol (2l)

4-*Iso*-proyl-aniline (1.60 mL 10.2 mmol, 1.00 eq.) was added to an aqueous solution containing H_2O (30 mL) and 37% HCl $_{(aq)}$. (5 mL) to prepare an aniline solution, which was added dropwise into an aqueous solution containing $NaNO_2$ (902 mg, 13.1 mmol, 1.28 eq.) in H_2O (5 mL) cooled in an ice water bath (0 °C) under strong stirring. After mixing, the resulting solution was stirred for 1 h at 0 °C to form a diazonium salt solution. Then, a separated aqueous solution containing phenol (1.00 mL, 11.3 mmol, 1.10 eq.) and NaOH (4.08 g, 102 mmol, 9.96 eq.) in H_2O (20 mL) was prepared, and the diazonium salt solution was added to the phenol solution slowly with cooling by an ice water bath. The color of the solution became dark during this process. After stirring at 0 °C for 1 h, the resulting solution was acidified by adding 20% $HCl_{(aq)}$ solution. Afterwards, the suspension was diluted with EtOAc (20 mL) and the phases were separated. The aqueous phase was extracted with EtOAc (3 x 10 mL) and the combined organic phases were dried over $MgSO_4$, filtered and the solvent removed under reduced pressure. The residue was purified by flash column chromatography (SiO_2, toluene) to yield an orange solid (1.87 g, 76%).

^{1}H NMR (400 MHz, DMSO) δ 10.26 (s, 1H), 7.76 (dd, *J* = 13.1, 8.7 Hz, 4H), 7.41 (d, *J* = 8.4 Hz, 2H), 6.94 (d, *J* = 8.9 Hz, 2H), 3.04 – 2.89 (m, 1H), 1.24 (s, 6H).

^{13}C NMR (101 MHz, DMSO) δ 160.71, 151.21, 150.47, 145.26, 127.18, 124.67, 122.21, 115.90, 33.35, 23.71.

HRMS (ESI): m/z for $C_{16}H_{17}N_2O^+$; calcd 241.1336, found 241.1322; m.p. 70.1 °C

Analytical data corresponds to literature.[1]

Synthesis of 4-(4-*tert*-butylphenyl)diazenylphenol (2m)

4-*Tert*-butyl-aniline (1.60 mL 9.85 mmol, 1.00 eq.) was added to an aqueous solution containing H_2O (30 mL) and 37% HCl $_{(aq)}$. (5 mL) to prepare an aniline solution, which was added dropwise into an aqueous solution containing $NaNO_2$ (902 mg, 13.1 mmol, 1.33 eq.) in H_2O (5 mL) cooled in an ice water bath (0 °C) under strong stirring. After mixing, the resulting solution was stirred for 1 h at 0 °C to form a diazonium salt solution. Then, a separated aqueous solution containing phenol (1.00 mL, 11.3 mmol, 1.14 eq.) and NaOH (4.08 g, 102 mmol, 10.4 eq.) in H_2O (20 mL) was prepared, and the diazonium salt solution was added to the phenol solution slowly with cooling by an ice

water bath. The color of the solution became dark during this process. After stirring at 0 °C for 1 h, the resulting solution was acidified by adding 20% $HCl_{(aq)}$ solution. Afterwards, the suspension was diluted with EtOAc (20 mL) and the phases were separated. The aqueous phase was extracted with EtOAc (3 x 10 mL) and the combined organic phases were dried over $MgSO_4$, filtered and the solvent removed under reduced pressure. The residue was purified by flash column chromatography (SiO_2, toluene) to yield an orange solid (1.72 g, 69%).

^{1}H NMR (400 MHz, DMSO) δ 10.26 (s, 1H), 7.77 (dd, *J* = 12.2, 8.8 Hz, 4H), 7.56 (d, *J* = 8.8 Hz, 2H), 6.94 (d, *J* = 8.9 Hz, 2H), 1.32 (s, 9H).

^{13}C NMR (101 MHz, DMSO) δ 160.71, 153.36, 150.03, 145.27, 126.05, 124.67, 121.87, 115.88, 34.67, 31.01.

HRMS (ESI): m/z for $C_{16}H_{19}N_2O^+$; calcd 255.1492, found 255.1388; m.p. 140.5 °C

Analytical data corresponds to literature.[5]

Synthesis of 4-(2,6-dimethylphenyl)diazenylphenol (2n)

2,6-Dimethyl-aniline (1.20 mL 9.62 mmol, 1.00 eq.) was added to an aqueous solution containing H_2O (30 mL) and 37% $HCl_{(aq)}$. (5 mL) to prepare an aniline solution, which was added dropwise into an aqueous solution containing $NaNO_2$ (913 mg, 13.2 mmol, 1.38 eq.) in H_2O (5 mL) cooled in an ice water bath (0 °C) under strong stirring. After mixing, the resulting solution was stirred for 1 h at 0 °C to form a diazonium salt solution. Then, a separated aqueous solution containing phenol (0.95 mL, 10.7 mmol, 1.11 eq.) and NaOH (3.97 g, 99.3 mmol, 10.3 eq.) in H_2O (20 mL) was prepared, and the diazonium salt solution was added to the phenol solution slowly with cooling by an ice water bath. The color of the solution became dark during this process. After stirring at 0 °C for 1 h, the resulting solution was acidified by adding 20% $HCl_{(aq)}$ solution. Afterwards, the suspension was diluted with EtOAc (20 mL) and the phases were separated. The aqueous phase was extracted with EtOAc (3 x 10 mL) and the combined organic phases were dried over $MgSO_4$, filtered and the solvent removed under reduced pressure. The residue was purified by flash column chromatography (SiO_2, toluene) to yield an orange solid (1.52 g, 70%).

^{1}H NMR (400 MHz, DMSO) δ 10.25 (s, 1H), 7.80 – 7.71 (m, 2H), 7.14 (s, 3H), 6.99 – 6.91 (m, 2H), 2.23 (s, 6H).

^{13}C NMR (101 MHz, DMSO) δ 160.88, 150.97, 145.59, 129.62, 128.91, 127.48, 124.31, 115.82, 18.31.

HRMS (ESI): m/z for $C_{14}H_{14}N_2ONa^+$; calcd 249.0998, found 249.1002; m.p. 147.1 °C
Analytical data corresponds to literature.[6]

Synthesis of 4-(2,4,6-trimethylphenyl)diazenylphenol (2o)

2,4,6-Trimethyl-aniline (1.50 mL 10.3 mmol, 1.00 eq.) was added to an aqueous solution containing H_2O (30 mL) and 37% HCl $_{(aq)}$. (5 mL) to prepare an aniline solution, which was added dropwise into an aqueous solution containing $NaNO_2$ (917 mg, 13.3 mmol, 1.29 eq.) in H_2O (5 mL) cooled in an ice water bath (0 °C) under strong stirring. After mixing, the resulting solution was stirred for 1 h at 0 °C to form a diazonium salt solution. Then, a separated aqueous solution containing phenol (1.00 mL, 11.3 mmol, 1.09 eq.) and NaOH (4.17 g, 104 mmol, 10.1 eq.) in H_2O (20 mL) was prepared, and the diazonium salt solution was added to the phenol solution slowly with cooling by an ice water bath. The color of the solution became dark during this process. After stirring at 0 °C for 1 h, the resulting solution was acidified by adding 20% $HCl_{(aq)}$ solution. Afterwards, the suspension was diluted with EtOAc (20 mL) and the phases were separated. The aqueous phase was extracted with EtOAc (3 x 10 mL) and the combined organic phases were dried over $MgSO_4$, filtered and the solvent removed under reduced pressure. The residue was purified by flash column chromatography (SiO_2, toluene) to yield an orange solid (960 mg, 39%).

^{1}H NMR (400 MHz, DMSO) δ 10.19 (s, 1H), 7.73 (d, *J* = 8.9 Hz, 2H), 6.97 – 6.85 (m, 4H), 2.26 (d, *J* = 7.3 Hz, 9H).

^{13}C NMR (101 MHz, DMSO) δ 160.59, 148.33, 145.71, 137.17, 130.24, 129.66, 124.15, 115.79, 20.61, 18.66.

HRMS (ESI): m/z for $C_{15}H_{17}N_2O^+$; calcd 241.1336, found 241.1332; m.p. 143.3 °C
Analytical data corresponds to literature.[7]

Synthesis of 4-(3,5-dimethylphenyl)diazenylphenol (2p)

3,5-Dimethyl-aniline (1.30 mL 10.2 mmol, 1.00 eq.) was added to an aqueous solution containing H_2O (30 mL) and 37% HCl $_{(aq)}$. (5 mL) to prepare an aniline solution, which was added dropwise into an aqueous solution containing $NaNO_2$ (884 mg, 18.8 mmol, 1.26 eq.) in H_2O (5 mL) cooled in an ice water bath (0 °C) under strong stirring. After mixing, the resulting solution was stirred for 1 h at 0 °C to form a diazonium salt solution. Then, a separated aqueous solution containing phenol (1.00 mL, 11.3 mmol, 1.10 eq.) and NaOH (4.25 g, 106 mmol, 10.4 eq.) in H_2O (20 mL) was prepared, and the

diazonium salt solution was added to the phenol solution slowly with cooling by an ice water bath. The color of the solution became dark during this process. After stirring at 0 °C for 1 h, the resulting solution was acidified by adding 20% $HCl_{(aq)}$ solution. Afterwards, the suspension was diluted with EtOAc (20 mL) and the phases were separated. The aqueous phase was extracted with EtOAc (3 x 10 mL) and the combined organic phases were dried over $MgSO_4$, filtered and the solvent removed under reduced pressure. The residue was purified by flash column chromatography (SiO_2, toluene) to yield an orange solid (1.53 g, 66%).

^{1}H NMR (400 MHz, DMSO) δ 10.26 (s, 1H), 7.77 (d, *J* = 8.8 Hz, 2H), 7.43 (s, 2H), 7.13 (s, 1H), 6.93 (d, *J* = 8.8 Hz, 2H), 2.36 (s, 6H).

^{13}C NMR (101 MHz, DMSO) δ 160.78, 152.23, 145.25, 138.48, 131.86, 124.67, 119.88, 115.89, 20.83.

HRMS (ESI): m/z for $C_{14}H_{14}N_2ONa^+$; calcd 249.0998, found 249.0992; m.p. 172.7 °C

Analytical data corresponds to literature.[8]

Synthesis of 4-(2,6-diflouro-phenyl)diazenylphenol (2q)

2,6-Difluoro-aniline (2.80 mL, 25.5 mmol, 1.00 eq.) was added to an aqueous solution containing H_2O (40 mL) and 37% $HCl_{(aq)}$. (10 mL) to prepare an aniline solution, which was added dropwise into an aqueous solution containing $NaNO_2$ (2.09 g, 30.3 mmol, 1.19 eq.) in H_2O (5 mL) cooled in an ice water bath (0 °C) under strong stirring. After mixing, the resulting solution was stirred for 1 h at 0 °C to form a diazonium salt solution. Then, a separated aqueous solution containing phenol (2.50 mL, 28.5 mmol, 1.12 eq.) and NaOH (2.60 g, 65.0 mmol, 2.55 eq.) in H_2O (20 mL) was prepared, and the diazonium salt solution was added to the phenol solution slowly with cooling by an ice water bath. The color of the solution became dark during this process. After stirring at 0 °C for 1 h, the resulting solution was acidified by adding 20% $HCl_{(aq)}$ solution. Afterwards, the suspension was diluted with EtOAc (20 mL) and the phases were separated. The aqueous phase was extracted with EtOAc (3 x 10 mL) and the combined organic phases were dried over $MgSO_4$, filtered and the solvent removed under reduced pressure. The residue was purified by flash column chromatography (SiO_2, toluene) to yield an orange solid (3.51 g, 59%).

^{1}H NMR (400 MHz, DMSO) δ 10.51 (s, 1H), 7.83 – 7.74 (m, 2H), 7.53 – 7.42 (m, 1H), 7.33 – 7.22 (m, 2H), 7.00 – 6.92 (m, 2H).

^{13}C NMR (101 MHz, DMSO) δ 162.02, 154.72 (dd, *J* = 255.0, 4.7 Hz), 145.99, 130.75 (t, *J* = 10.8 Hz), 130.34 (t, *J* = 10.3 Hz), 125.13, 116.08, 112.99 – 112.59 (m).
HRMS (ESI): m/z for $C_{12}H_9F_2N_2O^+$; calcd 235.0678, found 235.0673; m.p. 88.6 °C
Analytical data corresponds to literature.[9]

Synthesis of 4-(3-methoxy-phenyl)diazenylphenol (2r)

3-Methoxy-aniline (2.70 mL 24.1 mmol, 1.00 eq.) was added to an aqueous solution containing H_2O (30 mL) and 37% HCl $_{(aq)}$. (7.5 mL) to prepare an aniline solution, which was added dropwise into an aqueous solution containing $NaNO_2$ (2.20 g, 31.9 mmol, 1.32 eq.) in H_2O (5 mL) cooled in an ice water bath (0 °C) under strong stirring. After mixing, the resulting solution was stirred for 1 h at 0 °C to form a diazonium salt solution. Then, a separated aqueous solution containing phenol (2.20 mL, 24.8 mmol, 1.03 eq.) and NaOH (9.80 g, 245 mmol, 10.2 eq.) in H_2O (50 mL) was prepared, and the diazonium salt solution was added to the phenol solution slowly with cooling by an ice water bath. The color of the solution became dark during this process. After stirring at 0 °C for 1 h, the resulting solution was acidified by adding 20% $HCl_{(aq)}$ solution. Afterwards, the suspension was diluted with EtOAc (20 mL) and the phases were separated. The aqueous phase was extracted with EtOAc (3 x 20 mL) and the combined organic phases were dried over $MgSO_4$, filtered and the solvent removed under reduced pressure. The residue was purified by flash column chromatography (SiO_2, toluene) to yield an orange solid (3.33 g, 61%).
^{1}H NMR (400 MHz, DMSO) δ 10.32 (s, 1H), 7.81 (d, *J* = 8.9 Hz, 2H), 7.51 – 7.41 (m, 2H), 7.39 – 7.31 (m, 1H), 7.07 (d, *J* = 8.6 Hz, 1H), 6.95 (d, *J* = 8.9 Hz, 2H), 3.84 (s, 3H).
^{13}C NMR (101 MHz, DMSO) δ 161.03, 160.04, 153.38, 145.12, 130.09, 124.91, 116.70, 115.95, 115.70, 105.79, 55.31.
HRMS (ESI): m/z for $C_{13}H_{12}N_2O_2Na^+$; calcd 251.0791, found 251.0821; m.p. 116.7 °C

Synthesis of *p*-OHex-Azobenzene (3a)

A mixture of 4-hydroxyazobenzene **2a** (402 mg, 2.03 mmol, 1.00 eq.) and K_2CO_3 (1.21 g, 8.74 mmol, 4.31 eq.) in acetone (100 mL) was dissolved in a 250 mL flask while stirring. To this solution the 1-bromohexane (1.20 mL, 8.58 mmol, 4.23 eq.) was added and stirred for 3 days at 70 °C. The color of solution was changed to yellow. After cooling the solution to rt, K_2CO_3 was removed by filtration, and the solvents were

removed under *vacuo* to obtain the yellowish crude product. The product was purified by column chromatography (SiO_2, toluene: cyclohexane; 1 : 5) to obtain an orange solid (540 mg, 99%).

^{1}H NMR (400 MHz, $CDCl_3$) δ 7.98 – 7.92 (m, 2H), 7.92 – 7.87 (m, 2H), 7.50 (dd, *J* = 8.4, 6.5 Hz, 2H), 7.47 – 7.40 (m, 1H), 7.07 – 6.97 (m, 2H), 4.05 (t, *J* = 6.6 Hz, 2H), 1.82 (dt, *J* = 14.7, 6.7 Hz, 2H), 1.49 (dtd, *J* = 14.9, 7.2, 4.5 Hz, 2H), 1.36 (h, *J* = 3.7 Hz, 4H), 0.98 – 0.88 (m, 3H).

Melting point: 56.5 °C

Analytical data corresponds to literature.[10]

Synthesis of 1-(2-methylphenyl)-2-[4-(hexoxy)phenyl]diazene (3b)

A mixture of hydroxy-AB **2b** (161 mg, 0.757 mmol, 1.00 eq.), 1-bromohexane (0.13 mL, 0.929 mmol, 1.23 eq.) and K_2CO_3 (321 mg, 2.23 mmol, 3.07 eq) were dissolved in Acetone (15 mL) and stirred for 4 d at 70 °C. The color of solution was changed to yellow. After cooling the solution to room temperature, K_2CO_3 was filtered off, and the solvent was removed under reduced pressure to obtain yellowish crude product. The residue was purified by flash column chromatography (SiO_2, toluene: cyclohexane; 1:1) to yield an orange solid (199 mg, 89%).

^{1}H NMR (400 MHz, $CDCl_3$) δ 7.92 (d, *J* = 9.0 Hz, 2H), 7.61 (d, *J* = 7.7 Hz, 1H), 7.32 (d, *J* = 4.0 Hz, 2H), 7.28 – 7.23 (m, 1H), 7.00 (d, *J* = 9.0 Hz, 2H), 4.05 (t, *J* = 6.6 Hz, 2H), 2.70 (s, 3H), 1.88 – 1.77 (m, 2H), 1.55 – 1.43 (m, 2H), 1.36 (q, *J* = 3.5 Hz, 4H), 0.97 – 0.87 (m, 3H).

^{13}C NMR (101 MHz, $CDCl_3$) δ 161.75, 150.96, 147.36, 137.66, 131.29, 130.41, 126.55, 124.95, 115.64, 114.85, 68.54, 31.73, 29.32, 25.85, 22.76, 17.67, 14.18.

HRMS (ESI): m/z for $C_{19}H_{25}N_2O^+$; calcd 297.1962, found 297.1962; m.p. 66.8 °C

Synthesis of 1-(2-ethylphenyl)-2-[4-(hexoxy)phenyl]diazene (3c)

A mixture of hydroxy-AB **2c** (238 mg, 1.05 mmol, 1.00 eq.), 1-bromohexane (0.18 mL, 1.29 mmol, 1.22 eq.) and K_2CO_3 (500 mg, 3.03 mmol, 2.88 eq.) were dissolved in Acetone (15 mL) and stirred for 4 d at 70 °C. The color of solution was changed to yellow. After cooling the solution to room temperature, K_2CO_3 was filtered off, and the solvent was removed under reduced pressure to obtain yellowish crude product. The residue was purified by flash column chromatography (SiO_2, toluene cyclohexane 1:1) to yield an orange solid (257 mg, 79%).

^{1}H NMR (400 MHz, $CDCl_3$) δ 7.91 (d, *J* = 9.0 Hz, 2H), 7.61 (d, *J* = 9.0 Hz, 1H), 7.35 (d, *J* = 5.9 Hz, 2H), 7.28 – 7.21 (m, 2H), 7.00 (d, *J* = 9.0 Hz, 2H), 4.05 (t, *J* = 6.5 Hz, 2H), 3.14 (q, *J* = 7.6 Hz, 2H), 1.88 – 1.77 (m, 2H), 1.52 – 1.44 (m, 2H), 1.39 – 1.33 (m, 4H), 1.30 (t, *J* = 7.6 Hz, 3H), 0.97 – 0.86 (m, 3H).
^{13}C NMR (101 MHz, $CDCl_3$) δ 161.67, 150.46, 147.49, 143.67, 130.59, 129.80, 126.60, 124.91, 115.46, 114.85, 68.53, 31.73, 29.32, 25.85, 24.87, 22.75, 16.52, 14.18.
HRMS (ESI): m/z for $C_{20}H_{26}N_2ONa^+$; calcd 333.1937, found 333.1939; m.p. 31.4 °C

Synthesis of 1-(2-*iso*-propylphenyl)-2-[4-(hexoxy)phenyl]diazene (3d)

A mixture of hydroxy-AB **2d** (236 mg, 0.982 mmol, 1.00 eq.), 1-bromohexane (0.17 mL, 1.22 mmol, 1.24 eq.) and K_2CO_3 (496 mg, 3.00 mmol, 3.06 eq.) were dissolved in Acetone (15 mL) and stirred for 4 d at 70 °C. The color of solution was changed to yellow. After cooling the solution to room temperature, K_2CO_3 was filtered off, and the solvent was removed under reduced pressure to obtain yellowish crude product. The residue was purified by flash column chromatography (SiO_2, toluene cyclohexane 1:1) to yield an orange solid (239 mg, 75%).
^{1}H NMR (400 MHz, DMSO) δ 7.91 – 7.83 (m, 2H), 7.55 – 7.41 (m, 3H), 7.30 (ddd, *J* = 8.0, 6.9, 1.7 Hz, 1H), 7.20 – 7.09 (m, 2H), 4.08 (t, *J* = 6.5 Hz, 2H), 3.99 (h, *J* = 7.2 Hz, 1H), 1.75 (p, *J* = 6.6 Hz, 2H), 1.48 – 1.38 (m, 2H), 1.36 – 1.25 (m, 10H), 0.92 – 0.86 (m, 3H).
^{13}C NMR (101 MHz, DMSO) δ 161.35, 148.89, 146.79, 146.57, 130.98, 126.50, 126.40, 124.56, 115.07, 114.89, 67.98, 30.98, 28.54, 27.30, 25.14, 23.64, 22.06, 13.91.
HRMS (ESI): m/z for $C_{21}H_{29}N_2O^+$; calcd 325.2275, found 325.2278; m.p. 44.4 °C

Synthesis of 1-(2-*tert*-butylphenyl)-2-[4-(hexoxy)phenyl]diazene (3e)

A mixture of hydroxy-AB **2e** (127 mg, 501 µmol, 1.00 eq.), 1-bromohexane (90 µL, 634 µmol, 1.3 eq.) and K_2CO_3 (251 mg, 1.51 mmol, 3.03 eq) were dissolved in Acetone (15 mL) and stirred for 4 d at 70 °C. The color of solution was changed to yellow. After cooling the solution to room temperature, K_2CO_3 was filtered off, and the solvent was removed under reduced pressure to obtain yellowish crude product. The residue was purified by flash column chromatography (SiO_2, toluene cyclohexane 1:4) to yield an orange solid (122 mg, 72%).
^{1}H NMR (400 MHz, $CDCl_3$) δ 7.94 – 7.87 (m, 2H), 7.52 (dd, *J* = 7.8, 1.6 Hz, 1H), 7.49 (dd, *J* = 7.8, 1.5 Hz, 1H), 7.35 (td, *J* = 7.6, 1.6 Hz, 1H), 7.30 – 7.26 (m, 1H), 7.05 – 6.99

(m, 2H), 4.05 (t, *J* = 6.6 Hz, 2H), 1.83 (dq, *J* = 7.8, 6.5 Hz, 2H), 1.54 (s, 9H), 1.51 (s, 2H), 1.36 (tt, *J* = 7.2, 3.3 Hz, 4H), 0.97 – 0.88 (m, 3H).

^{13}C NMR (101 MHz, $CDCl_3$) δ 161.66, 152.08, 147.47, 147.37, 129.91, 126.81, 126.54, 125.19, 116.70, 114.93, 68.54, 36.19, 32.05, 31.72, 29.31, 25.85, 22.76, 14.19.

HRMS (ESI): m/z for $C_{22}H_{30}N_2ONa^+$; calcd 361.2240, found 361.-2212; m.p. 54.6 °C

Synthesis of 1-(3-methylphenyl)-2-[4-(hexoxy)phenyl]diazene (3f)

A mixture of hydroxy-AB **2f** (147 mg, 691 µmol, 1.00 eq.), 1-bromohexane (0.13 mL, 0.93 mmol, 1.3 eq.) and K_2CO_3 (301 mg, 2.18 mmol, 3.15 eq) were dissolved in Acetone (15 mL) and stirred for 4 d at 70 °C. The color of solution was changed to yellow. After cooling the solution to room temperature, K_2CO_3 was filtered off, and the solvent was removed under reduced pressure to obtain yellowish crude product. The residue was purified by flash column chromatography (SiO_2, toluene cyclohexane 1:1) to yield an orange solid (177 mg, 86%).

^{1}H NMR (400 MHz, $CDCl_3$) δ 7.89 – 7.80 (m, 2H), 7.66 – 7.57 (m, 2H), 7.36 – 7.27 (m, 1H), 7.20 – 7.17 (m, 1H), 6.97 – 6.89 (m, 2H), 3.97 (t, *J* = 6.6 Hz, 2H), 2.38 (s, 3H), 1.75 (dq, *J* = 7.9, 6.6 Hz, 2H), 1.41 (dq, *J* = 14.8, 7.2 Hz, 2H), 1.29 (dp, *J* = 7.1, 3.3 Hz, 4H), 0.89 – 0.80 (m, 3H).

^{13}C NMR (101 MHz, $CDCl_3$) δ 161.95, 152.82, 146.90, 139.06, 131.31, 129.00, 125.00, 122.77, 120.34, 114.89, 68.55, 31.72, 29.31, 25.84, 22.75, 21.53, 14.18.

HRMS (ESI): m/z for $C_{19}H_{25}N_2O^+$; calcd 297.1962, found 297.1959; m.p. 50.8 °C

Synthesis of 1-(3-ethylphenyl)-2-[4-(hexoxy)phenyl]diazene (3g)

A mixture of hydroxy-AB **2g** (248 mg, 1.10 mmol, 1.00 eq.), 1-bromohexane (0.18 mL, 1.3 mmol, 1.2 eq.) and K_2CO_3 (538 mg, 3.26 mmol, 2.97 eq) were dissolved in Acetone (15 mL) and stirred for 4 d at 70 °C. The color of solution was changed to yellow. After cooling the solution to room temperature, K_2CO_3 was filtered off, and the solvent was removed under reduced pressure to obtain yellowish crude product. The residue was purified by flash column chromatography (SiO_2, toluene cyclohexane 1:1) to yield an orange solid (307 mg, 90%).

^{1}H NMR (400 MHz, $CDCl_3$) δ 7.97 – 7.88 (m, 2H), 7.78 – 7.68 (m, 2H), 7.42 (t, *J* = 7.7 Hz, 1H), 7.32 – 7.27 (m, 1H), 7.05 – 6.97 (m, 2H), 4.04 (t, *J* = 6.6 Hz, 2H), 2.76 (q, *J* = 7.6 Hz, 2H), 1.82 (dq, *J* = 7.9, 6.6 Hz, 2H), 1.49 (dtd, *J* = 9.6, 7.3, 4.9 Hz, 2H), 1.37 (tt, *J* = 7.0, 3.2 Hz, 4H), 1.31 (t, *J* = 7.6 Hz, 3H), 0.99 – 0.87 (m, 3H).

^{13}C NMR (101 MHz, $CDCl_3$) δ 161.85, 153.02, 146.98, 145.42, 130.16, 129.07, 124.90, 121.88, 120.30, 114.85, 68.52, 31.72, 29.32, 28.95, 25.84, 22.75, 15.64, 14.18.
HRMS (ESI): m/z for $C_{20}H_{27}N_2O^+$; calcd 311.2118, found 311.2117; m.p. 44.2 °C

Synthesis of 1-(3-*iso*-propylphenyl)-2-[4-(hexoxy)phenyl]diazene (3h)

A mixture of hydroxy-AB **2h** (249 mg, 1.04 mmol, 1.00 eq.), 1-bromohexane (0.17 mL, 1.2 mmol, 1.2 eq.) and K_2CO_3 (504 mg, 3.05 mmol, 2.94 eq) were dissolved in Acetone (15 mL) and stirred for 4 d at 70 °C. The color of solution was changed to yellow. After cooling the solution to room temperature, K_2CO_3 was filtered off, and the solvent was removed under reduced pressure to obtain yellowish crude product. The residue was purified by flash column chromatography (SiO_2, toluene cyclohexane 1:1) to yield an orange solid (308 mg, 92%).
^1H NMR (400 MHz, $CDCl_3$) δ 8.00 – 7.88 (m, 2H), 7.78 (t, *J* = 1.9 Hz, 1H), 7.71 (ddd, *J* = 7.8, 2.0, 1.2 Hz, 1H), 7.42 (t, *J* = 7.7 Hz, 1H), 7.32 (dt, *J* = 7.7, 1.5 Hz, 1H), 7.07 – 6.97 (m, 2H), 4.05 (t, *J* = 6.6 Hz, 2H), 3.02 (hept, *J* = 6.9 Hz, 1H), 1.82 (dq, *J* = 7.9, 6.6 Hz, 2H), 1.49 (p, *J* = 7.0 Hz, 2H), 1.40 – 1.36 (m, 2H), 1.32 (d, *J* = 6.9 Hz, 6H), 0.97 – 0.87 (m, 3H).
^{13}C NMR (101 MHz, $CDCl_3$) δ 161.99, 152.83, 150.13, 146.86, 129.10, 128.89, 125.06, 120.90, 120.08, 114.89, 68.56, 34.29, 31.73, 29.32, 25.84, 24.09, 22.75, 14.19.
HRMS (ESI): m/z for $C_{21}H_{29}N_2O^+$; calcd 325.2275, found 325.2278; m.p. 36.7 °C

Synthesis of 1-(3-*tert*-butylphenyl)-2-[4-(hexoxy)phenyl]diazene (3i)

A mixture of hydroxy-AB **2i** (256 mg, 1.01 mmol, 1.00 eq.), 1-bromohexane (0.17 mL, 1.2 mmol, 1.2 eq.) and K_2CO_3 (512 mg, 3.10 mmol, 3.08 eq) were dissolved in Acetone (15 mL) and stirred for 4 d at 70 °C. The color of solution was changed to yellow. After cooling the solution to room temperature, K_2CO_3 was filtered off, and the solvent was removed under reduced pressure to obtain yellowish crude product. The residue was purified by flash column chromatography (SiO_2, toluene cyclohexane 1:1) to yield an orange solid (314 mg, 92%).
^1H NMR (400 MHz, $CDCl_3$) δ 7.96 – 7.91 (m, 3H), 7.69 (ddd, *J* = 7.6, 1.9, 1.3 Hz, 1H), 7.48 (ddd, *J* = 7.8, 2.0, 1.3 Hz, 1H), 7.42 (t, *J* = 7.7 Hz, 1H), 7.04 – 6.98 (m, 2H), 4.05 (t, *J* = 6.6 Hz, 2H), 1.82 (dq, *J* = 8.1, 6.6 Hz, 2H), 1.53 – 1.45 (m, 2H), 1.40 (s, 9H), 1.36 (q, *J* = 3.6 Hz, 4H), 0.96 – 0.89 (m, 3H).

^{13}C NMR (101 MHz, $CDCl_3$) δ 161.76, 152.60, 152.31, 146.80, 128.70, 127.53, 124.83, 120.65, 118.84, 114.73, 68.41, 34.93, 31.59, 31.35, 29.18, 25.71, 22.62, 14.05.
HRMS (ESI): m/z for $C_{22}H_{31}N_2O^+$; calcd 339.2431, found 339.2437; m.p. 54.5 °C

Synthesis of 1-(4-methylphenyl)-2-[4-(hexoxy)phenyl]diazene (3j)

A mixture of hydroxy-AB **2j** (155 mg, 730 µmol, 1.00 eq.), 1-bromohexane (0.14 mL, 1.0 mmol, 1.4 eq.) and K_2CO_3 (313 mg, 2.26 mmol, 3.10 eq) were dissolved in Acetone (15 mL) and stirred for 4 d at 70 °C. The color of solution was changed to yellow. After cooling the solution to room temperature, K_2CO_3 was filtered off, and the solvent was removed under reduced pressure to obtain yellowish crude product. The residue was purified by flash column chromatography (SiO_2, toluene) to yield an orange solid (182 mg, 84%).
^{1}H NMR (400 MHz, $CDCl_3$) δ 7.91 (d, *J* = 9.0 Hz, 2H), 7.80 (d, *J* = 8.3 Hz, 2H), 7.30 (d, *J* = 7.9 Hz, 2H), 7.00 (d, *J* = 9.0 Hz, 2H), 4.04 (t, *J* = 6.6 Hz, 2H), 2.43 (s, 3H), 1.88 – 1.76 (m, 2H), 1.54 – 1.43 (m, 2H), 1.42 – 1.31 (m, 4H), 0.97 – 0.87 (m, 3H).
^{13}C NMR (101 MHz, $CDCl_3$) δ 161.84, 150.73, 146.85, 141.02, 129.86, 124.93, 122.70, 114.89, 68.55, 31.73, 29.32, 25.85, 22.75, 21.61, 14.18.
HRMS (ESI): m/z for $C_{19}H_{25}N_2O^+$; calcd 297.1962, found 297.1961; m.p. 77.0 °C

Synthesis of 1-(4-ethylphenyl)-2-[4-(hexoxy)phenyl]diazene (3k)

A mixture of hydroxy-AB **2k** (231 mg, 1.02 mmol, 1.00 eq.), 1-bromohexane (0.17 mL, 1.2 mmol, 1.2 eq.) and K_2CO_3 (502 mg, 3.04 mmol, 2.98 eq) were dissolved in Acetone (15 mL) and stirred for 4 d at 70 °C. The color of solution was changed to yellow. After cooling the solution to room temperature, K_2CO_3 was filtered off, and the solvent was removed under reduced pressure to obtain yellowish crude product. The residue was purified by flash column chromatography (SiO_2, toluene cyclohexane 1:1) to yield an orange solid (298 mg, 94%).
^{1}H NMR (400 MHz, $CDCl_3$) δ 7.96 – 7.88 (m, 2H), 7.87 – 7.78 (m, 2H), 7.36 – 7.28 (m, 2H), 7.03 – 6.97 (m, 2H), 4.04 (t, *J* = 6.5 Hz, 2H), 2.72 (q, *J* = 7.6 Hz, 2H), 1.82 (dq, *J* = 8.0, 6.6 Hz, 2H), 1.49 (p, *J* = 7.1 Hz, 2H), 1.36 (dp, *J* = 7.1, 3.2 Hz, 4H), 1.28 (t, *J* = 7.6 Hz, 3H), 0.97 – 0.87 (m, 3H).
^{13}C NMR (101 MHz, $CDCl_3$) δ 161.84, 150.90, 147.31, 146.86, 128.66, 124.94, 122.77, 114.88, 68.55, 31.72, 29.31, 28.96, 25.84, 22.75, 15.58, 14.18.
HRMS (ESI): m/z for $C_{20}H_{27}N_2O^+$; calcd 311.2118, found 311.2117; m.p. 64.6 °C

Synthesis of 1-(4-*iso*-propylphenyl)-2-[4-(hexoxy)phenyl]diazene (3l)

A mixture of hydroxy-AB **2l** (247 mg, 1.03 mmol, 1.00 eq.), 1-bromohexane (0.17 mL, 1.2 mmol, 1.2 eq.) and K_2CO_3 (499 mg, 3.02 mmol, 2.94 eq.) were dissolved in Acetone (15 mL) and stirred for 4 d at 70 °C. The color of solution was changed to yellow. After cooling the solution to room temperature, K_2CO_3 was filtered off, and the solvent was removed under reduced pressure to obtain yellowish crude product. The residue was purified by flash column chromatography (SiO_2, toluene cyclohexane 1:1) to yield an orange solid (292 mg, 88%).

^{1}H NMR (400 MHz, $CDCl_3$) δ 7.96 – 7.88 (m, 2H), 7.87 – 7.79 (m, 2H), 7.39 – 7.31 (m, 2H), 7.04 – 6.97 (m, 2H), 4.04 (t, *J* = 6.6 Hz, 2H), 2.98 (hept, *J* = 6.9 Hz, 1H), 1.92 – 1.76 (m, 2H), 1.56 – 1.44 (m, 2H), 1.36 (dt, *J* = 7.2, 2.3 Hz, 4H), 1.30 (d, *J* = 6.9 Hz, 6H), 0.97 – 0.87 (m, 3H).

^{13}C NMR (101 MHz, $CDCl_3$) δ 161.83, 151.86, 151.01, 146.88, 127.22, 124.93, 122.76, 114.88, 68.54, 34.26, 31.72, 29.31, 25.84, 24.03, 22.75, 14.18.

HRMS (ESI): m/z for $C_{21}H_{29}N_2O^+$; calcd 325.2275, found 325.2277; m.p. 92.7 °C

Synthesis of 1-(4-*tert*-butylphenyl)-2-[4-(hexoxy)phenyl]diazene (3m)

A mixture of hydroxy-AB **2m** (253 mg, 995 µmol, 1.00 eq.), 1-bromohexane (0.17 mL, 1.2 mmol, 1.2 eq.) and K_2CO_3 (496 mg, 3.00 mmol, 3.02 eq.) were dissolved in Acetone (15 mL) and stirred for 4 d at 70 °C. The color of solution was changed to yellow. After cooling the solution to room temperature, K_2CO_3 was filtered off, and the solvent was removed under reduced pressure to obtain yellowish crude product. The residue was purified by flash column chromatography (SiO_2, toluene cyclohexane 1:1) to yield an orange solid (317 mg, 94%).

^{1}H NMR (400 MHz, $CDCl_3$) δ 7.88 – 7.79 (m, 2H), 7.78 – 7.71 (m, 2H), 7.48 – 7.41 (m, 2H), 6.96 – 6.88 (m, 2H), 3.97 (t, *J* = 6.6 Hz, 2H), 1.80 – 1.69 (m, 2H), 1.41 (p, *J* = 7.5 Hz, 2H), 1.30 (s, 13H), 0.89 – 0.80 (m, 3H).

^{13}C NMR (101 MHz, $CDCl_3$) δ 161.78, 154.02, 150.69, 146.99, 126.11, 124.87, 122.40, 114.86, 68.54, 35.10, 31.72, 31.44, 29.32, 25.85, 22.75, 14.18.

HRMS (ESI): m/z for $C_{22}H_{31}N_2O^+$; calcd 339.2431, found 339.2431; m.p. 116.6 °C

Synthesis of 1-(2,6-dimethylphenyl)-2-[4-(hexoxy)phenyl]diazene (3n)

A mixture of hydroxy-AB **2n** (230 mg, 1.02 mmol, 1.00 eq.), 1-bromohexane (0.17 mL, 1.2 mmol, 1.2 eq.) and K_2CO_3 (509 mg, 3.08 mmol, 3.03 eq) were dissolved in Acetone (15 mL) and stirred for 4 d at 70 °C. The color of solution was changed to yellow. After cooling the solution to room temperature, K_2CO_3 was filtered off, and the solvent was removed under reduced pressure to obtain yellowish crude product. The residue was purified by flash column chromatography (SiO_2, toluene cyclohexane 1:1) to yield an orange solid (271 mg, 86%).

^{1}H NMR (400 MHz, $CDCl_3$) δ 7.93 – 7.85 (m, 2H), 7.11 (s, 3H), 7.05 – 6.97 (m, 2H), 4.05 (t, *J* = 6.6 Hz, 2H), 2.31 (s, 6H), 1.83 (dq, *J* = 7.9, 6.6 Hz, 2H), 1.52 – 1.44 (m, 2H), 1.36 (dp, *J* = 7.2, 3.3 Hz, 4H), 0.97 – 0.88 (m, 3H).

^{13}C NMR (101 MHz, $CDCl_3$) δ 161.96, 151.84, 147.09, 130.38, 129.13, 127.66, 124.52, 114.83, 68.57, 31.72, 29.30, 25.84, 22.76, 18.78, 14.19.

HRMS (ESI): m/z for $C_{20}H_{27}N_2O^+$; calcd 311.2118, found 311.2119; m.p. 25.1 °C

Synthesis of 1-(2,4,6-trimethylphenyl)-2-[4-(hexoxy)phenyl]diazene (3o)

A mixture of hydroxy-AB **2o** (246 mg, 1.02 mmol, 1.00 eq.), 1-bromohexane (0.17 mL, 1.2 mmol, 1.2 eq.) and K_2CO_3 (502 mg, 3.04 mmol, 2.97 eq) were dissolved in Acetone (15 mL) and stirred for 4 d at 70 °C. The color of solution was changed to yellow. After cooling the solution to room temperature, K_2CO_3 was filtered off, and the solvent was removed under reduced pressure to obtain yellowish crude product. The residue was purified by flash column chromatography (SiO_2, toluene cyclohexane 1:1) to yield an orange solid (311 mg, 94%).

^{1}H NMR (400 MHz, $CDCl_3$) δ 7.92 – 7.83 (m, 2H), 7.03 – 6.98 (m, 2H), 6.92 (s, 2H), 4.04 (t, *J* = 6.6 Hz, 2H), 2.32 (d, *J* = 6.4 Hz, 9H), 1.82 (dq, *J* = 7.9, 6.6 Hz, 2H), 1.49 (p, *J* = 7.2 Hz, 2H), 1.36 (tt, *J* = 7.1, 3.3 Hz, 4H), 0.97 – 0.87 (m, 3H).

^{13}C NMR (101 MHz, $CDCl_3$) δ 161.72, 149.19, 147.18, 137.83, 131.01, 129.94, 124.39, 114.80, 68.54, 31.72, 29.31, 25.84, 22.76, 21.20, 19.08, 14.19.

HRMS (ESI): m/z for $C_{21}H_{29}N_2O^+$; calcd 325.2275, found 325.2280; m.p. 59.5 °C

Synthesis of 1-(3,5-Dimethylphenyl)-2-[4-(hexoxy)phenyl]diazene (3p)

A mixture of hydroxy-AB **2p** (233 mg, 1.03 mmol, 1.00 eq.), 1-bromohexane (0.17 mL, 1.2 mmol, 1.2 eq.) and K_2CO_3 (497 mg, 3.01 mmol, 2.92 eq.) were dissolved in Acetone (15 mL) and stirred for 4 d at 70 °C. The color of solution was changed to

yellow. After cooling the solution to room temperature, K_2CO_3 was filtered off, and the solvent was removed under reduced pressure to obtain yellowish crude product. The residue was purified by flash column chromatography (SiO_2, toluene cyclohexane 1:1) to yield an orange solid (285 mg, 89%).

^{1}H NMR (400 MHz, $CDCl_3$) δ 7.90 (d, *J* = 9.0 Hz, 2H), 7.51 (s, 2H), 7.09 (s, 1H), 7.00 (d, *J* = 9.0 Hz, 2H), 4.04 (s, 2H), 2.41 (s, 6H), 1.80 (d, *J* = 14.4 Hz, 2H), 1.55 – 1.43 (m, 2H), 1.36 (q, *J* = 3.6 Hz, 4H), 0.98 – 0.87 (m, 3H).

^{13}C NMR (101 MHz, $CDCl_3$) δ 161.80, 153.03, 147.00, 138.83, 132.20, 124.84, 120.46, 114.84, 68.52, 31.72, 29.31, 25.84, 22.75, 21.42, 14.18.

HRMS (ESI): m/z for $C_{20}H_{27}N_2O^+$; calcd 311.2118, found 311.2119; m.p. 57.9 °C

Synthesis of 1-(2,6-difluorophenyl)-2-[4-(hexoxy)phenyl]diazene (3q)

A mixture of hydroxy-AB **2q** (260 mg, 1.11 mmol, 1.00 eq.), 1-bromohexane (0.18 mL, 1.3 mmol, 1.2 eq.) and K_2CO_3 (534 mg, 3.23 mmol, 2.91 eq) were dissolved in Acetone (15 mL) and stirred for 4 d at 70 °C. The color of solution was changed to yellow. After cooling the solution to room temperature, K_2CO_3 was filtered off, and the solvent was removed under reduced pressure to obtain yellowish crude product. The residue was purified by flash column chromatography (SiO_2, toluene cyclohexane 1:1) to yield an orange solid (311 mg, 88%).

^{1}H NMR (400 MHz, $CDCl_3$) δ 7.92 (d, *J* = 9.2 Hz, 2H), 7.33 – 7.20 (m, 1H), 7.02 (dd, *J* = 12.0, 8.9 Hz, 4H), 4.05 (t, *J* = 6.5 Hz, 2H), 1.88 – 1.77 (m, 2H), 1.53 – 1.44 (m, 2H), 1.36 (t, *J* = 7.2 Hz, 4H), 0.95 – 0.87 (m, 3H).

^{13}C NMR (101 MHz, $CDCl_3$) δ 162.72, 155.88 (dd, *J* = 257.8, 4.6 Hz), 147.73, 131.79 (t, *J* = 10.4 Hz), 129.47 (t, *J* = 10.2 Hz), 125.21, 114.88, 112.73 – 112.40 (m), 68.61, 31.71, 29.26, 25.82, 22.74, 14.17.

HRMS (ESI): m/z for $C_{18}H_{20}F_2N_2ONa^+$; calcd 341.1436, found 341.1438; m.p. 40.9 °C

Synthesis of 1-(3-methoxyphenyl)-2-[4-(hexoxy)phenyl]diazene (3r)

A mixture of hydroxy-AB **2r** (123 mg, 0.539 mmol, 1.00 eq.), 1-bromohexane (0.11 mL, 0.69 mmol, 1.3 eq.) and K_2CO_3 (418 mg, 3.00 mmol, 3.00 eq) were dissolved in Acetone (15 mL) and stirred for 4 d at 70 °C. The color of solution was changed to yellow. After cooling the solution to room temperature, K_2CO_3 was filtered off, and the solvent was removed under reduced pressure to obtain yellowish crude product. The

residue was purified by flash column chromatography (SiO_2, toluene cyclohexane 1:1) to yield an orange solid (138 mg, 82%).

^{1}H NMR (400 MHz, $CDCl_3$) δ 7.98 – 7.93 (m, 2H), 7.54 (ddd, *J* = 7.8, 1.7, 1.0 Hz, 1H), 7.45 (dd, *J* = 2.6, 1.7 Hz, 1H), 7.41 (t, *J* = 8.0 Hz, 1H), 7.04 – 6.98 (m, 3H), 4.05 (t, *J* = 6.6 Hz, 2H), 3.90 (s, 3H), 1.82 (dq, *J* = 8.2, 6.6 Hz, 2H), 1.55 – 1.43 (m, 2H), 1.36 (tt, *J* = 7.2, 3.3 Hz, 4H), 1.00 – 0.87 (m, 3H).

^{13}C NMR (101 MHz, $CDCl_3$) δ 162.25, 160.48, 153.79, 146.63, 129.90, 125.29, 117.43, 116.84, 114.96, 105.76, 68.61, 55.63, 31.72, 29.30, 25.84, 22.75, 14.18.

HRMS (ESI): m/z for $C_{19}H_{24}N_2O_2Na^+$; calcd 335.1730, found 335.1730; 47.0 °C

3. NMR spectra

1H-NMR of 4-phenyldiazenylphenol (3a)

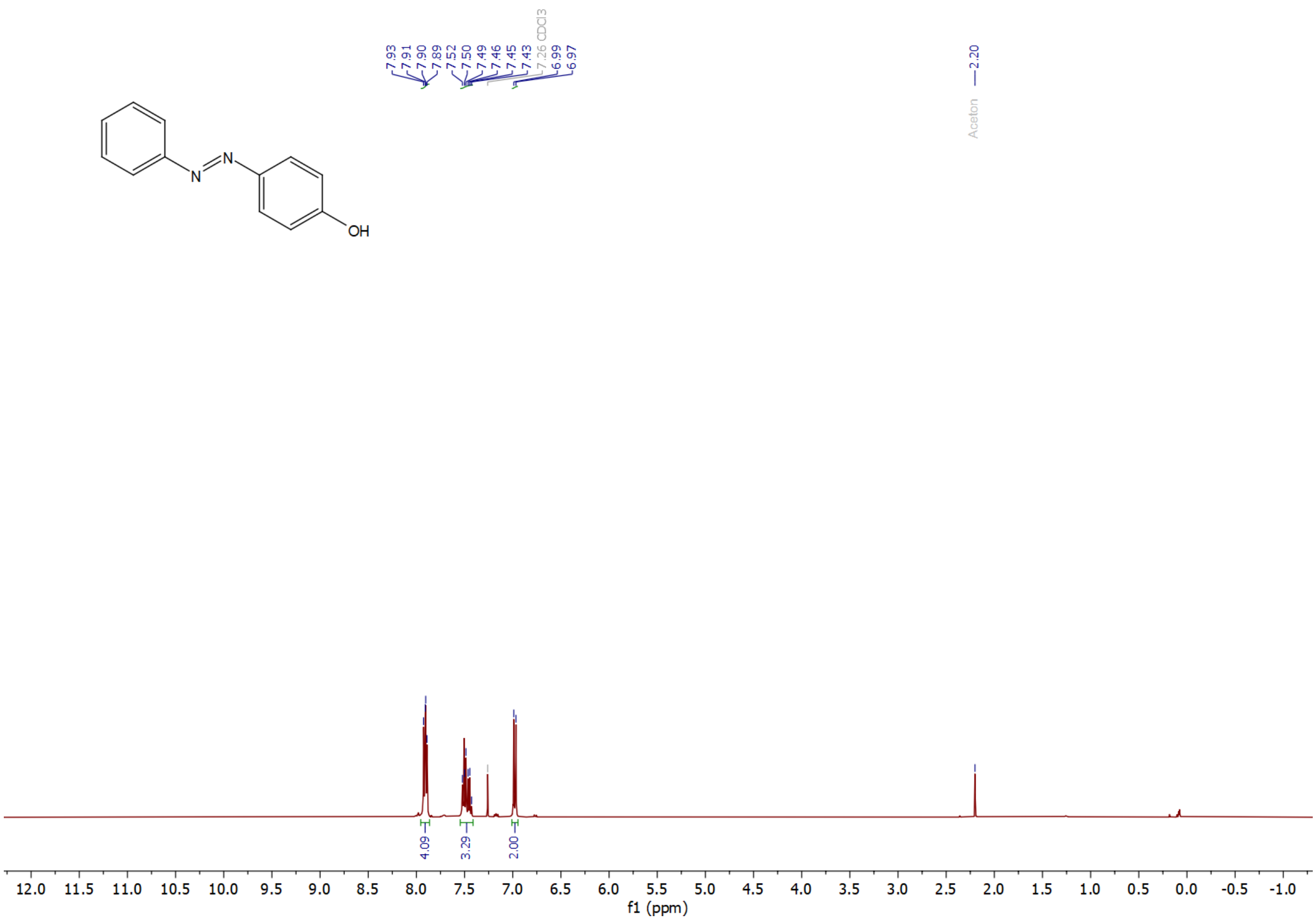

1H-NMR of 4-(2-methylphenyl)diazenylphenol (3b)

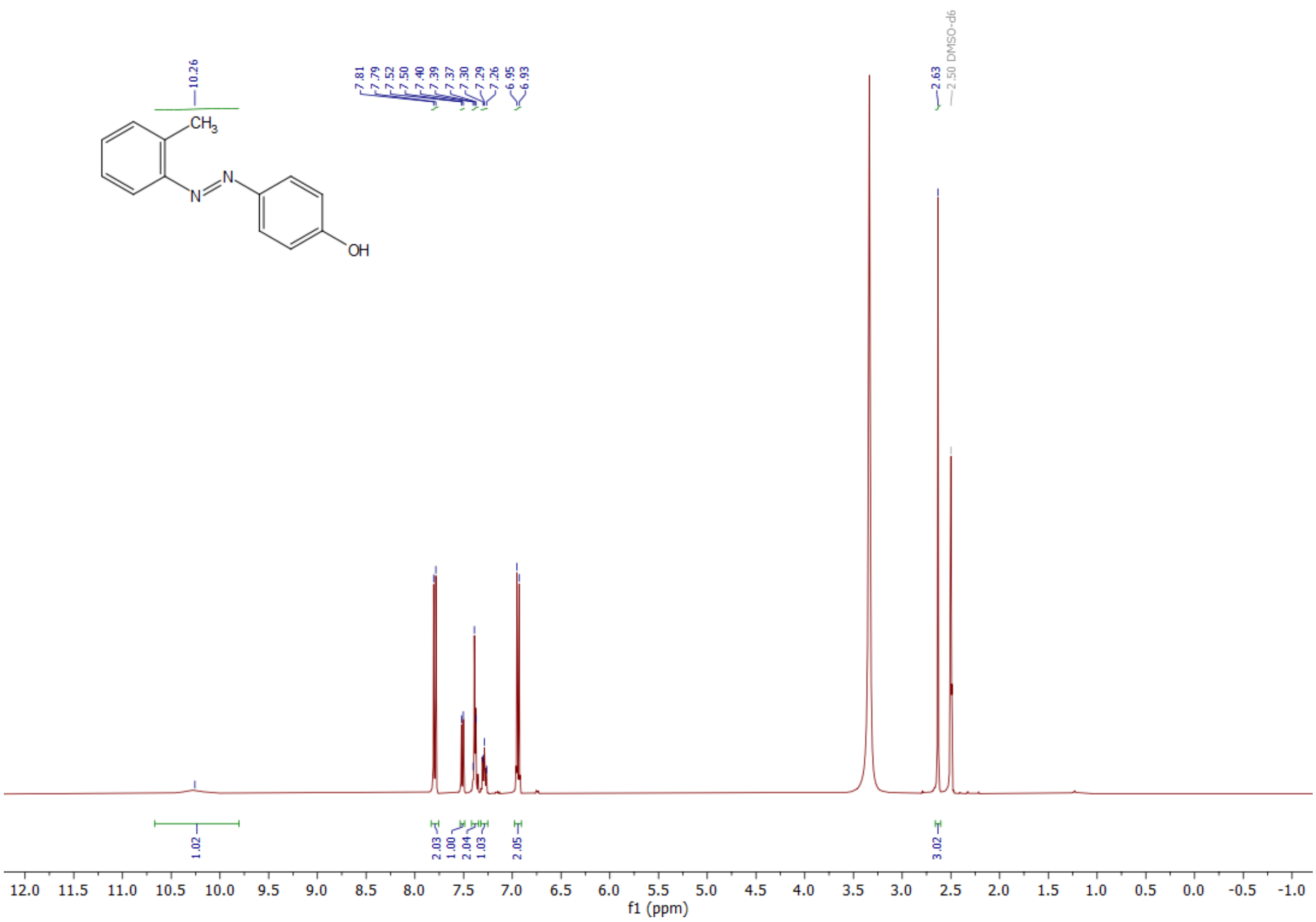

^{13}C-NMR of 4-(2-methylphenyl)diazenylphenol (3b)

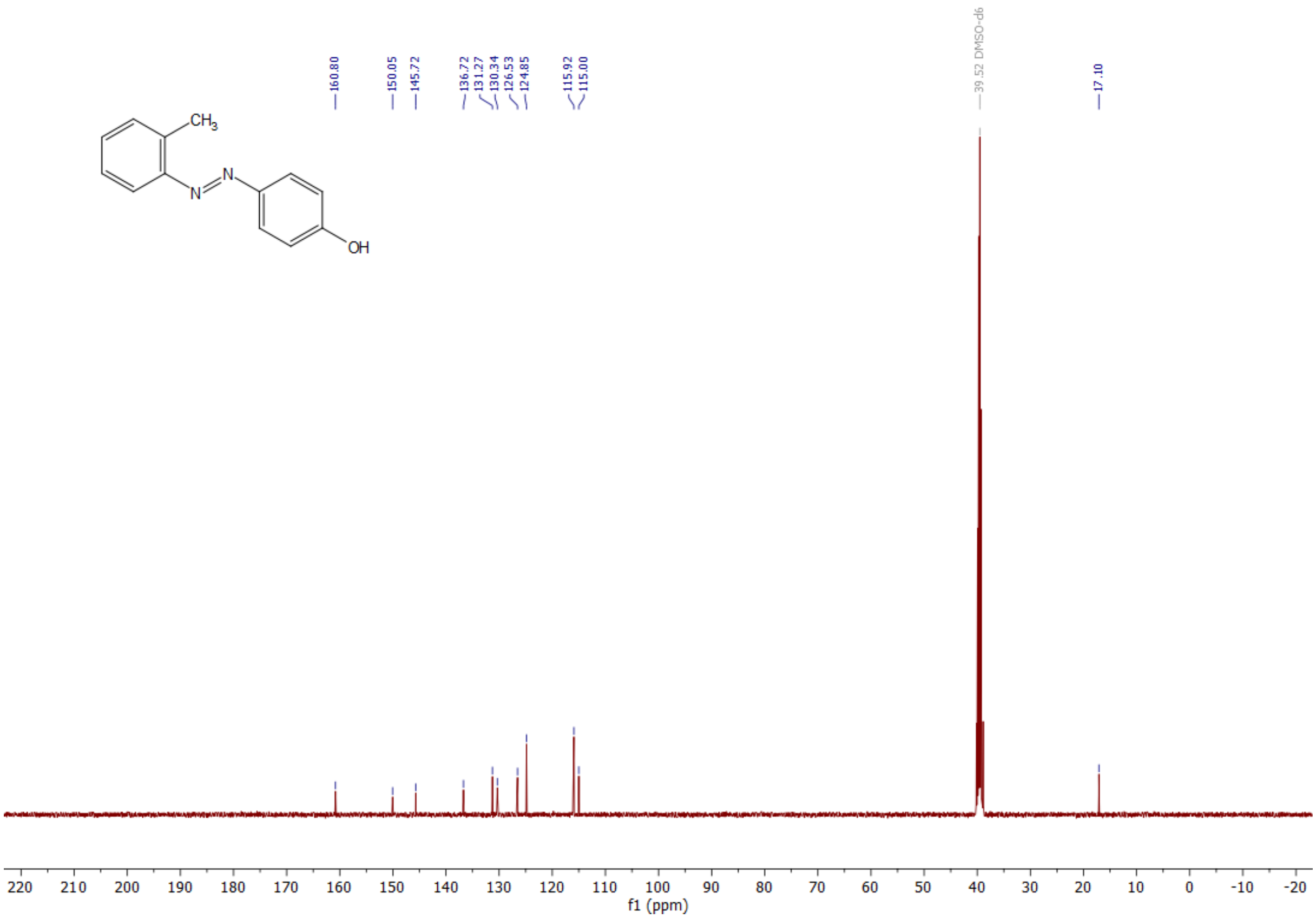

^{1}H-NMR of 4-(2-ethylphenyl)diazenylphenol (3c)

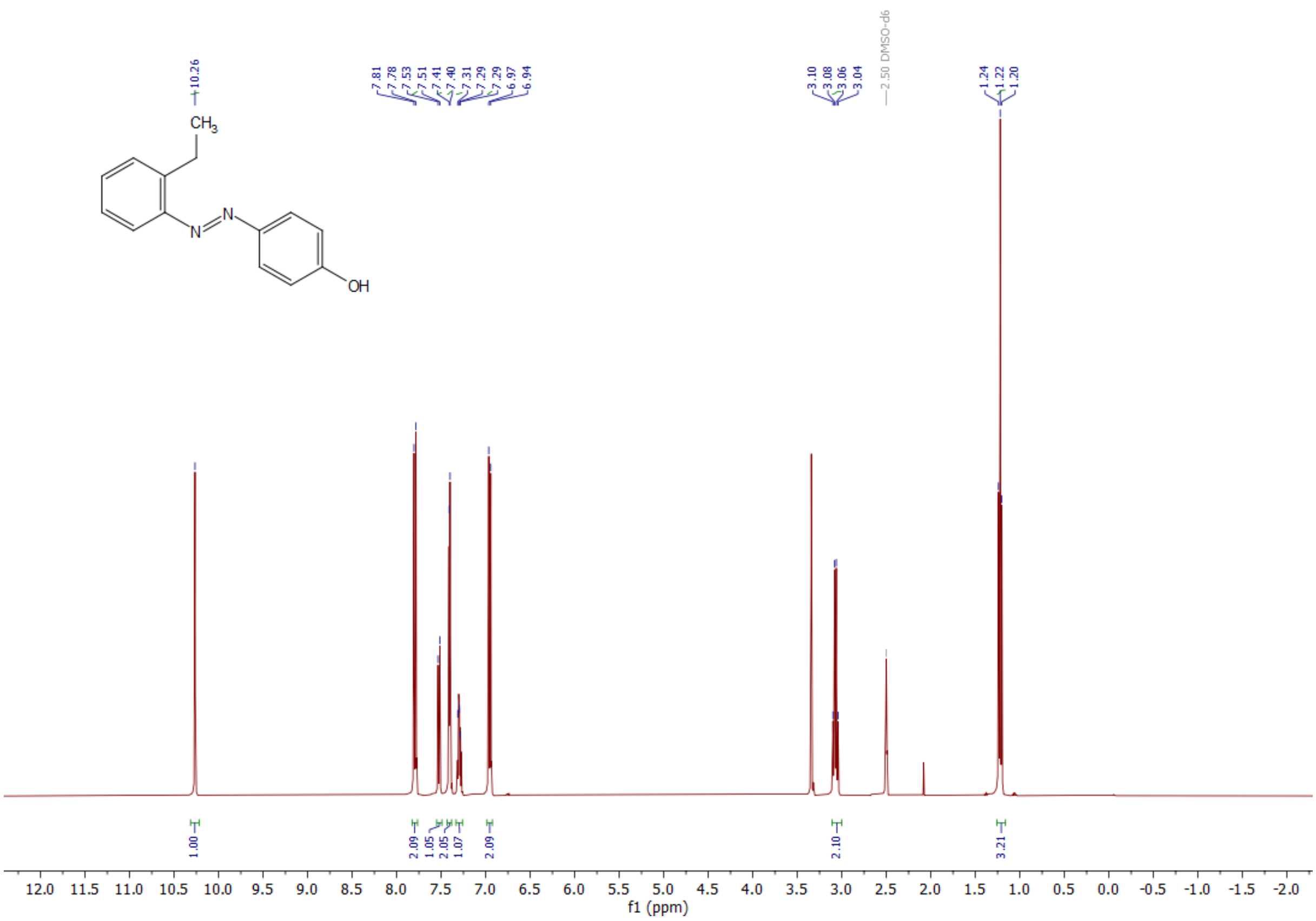

^{13}C-NMR of 4-(2-ethylphenyl)diazenylphenol (3c)

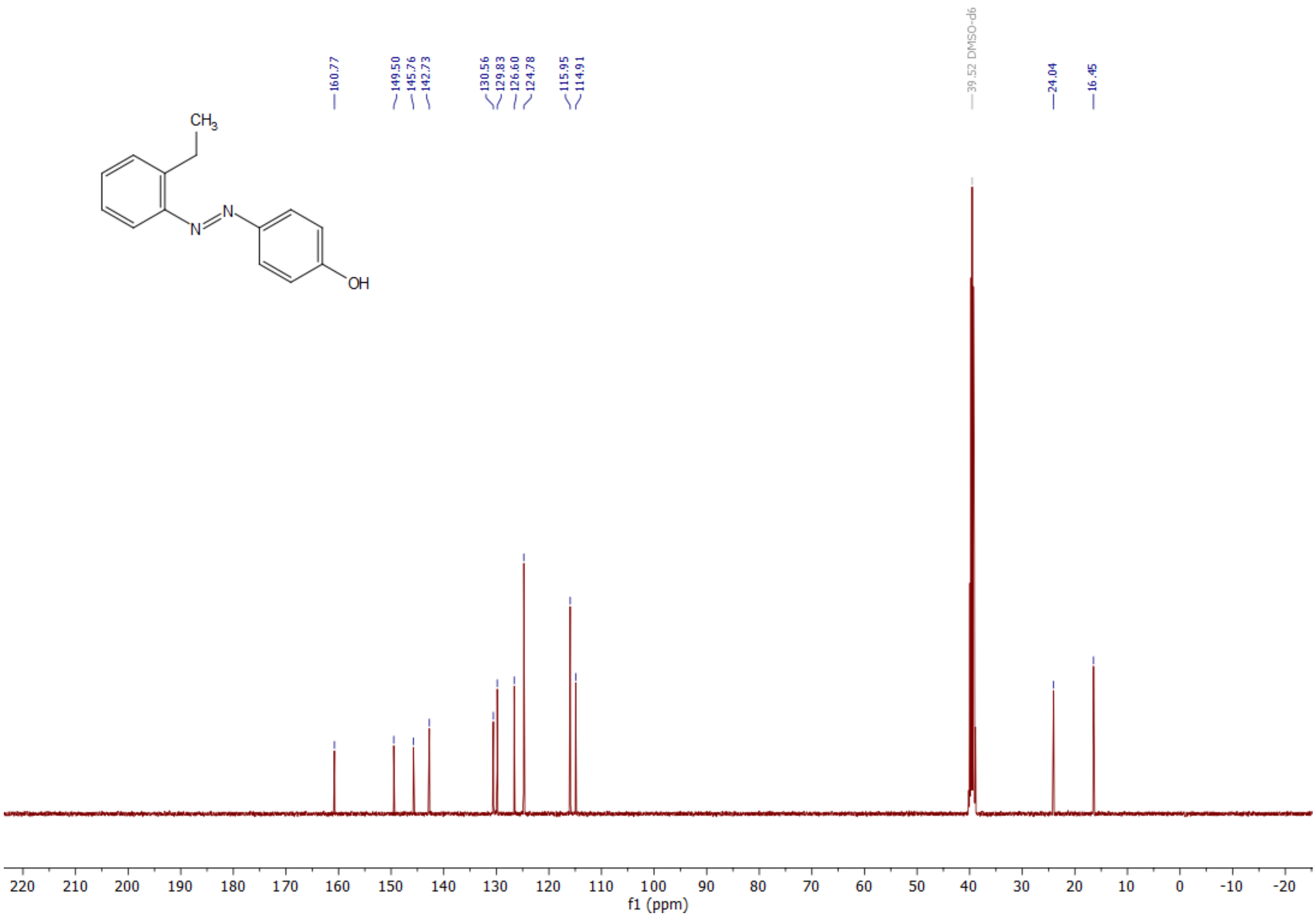

^{1}H-NMR of 4-(2-*iso*-propylphenyl)diazenylphenol (3d)

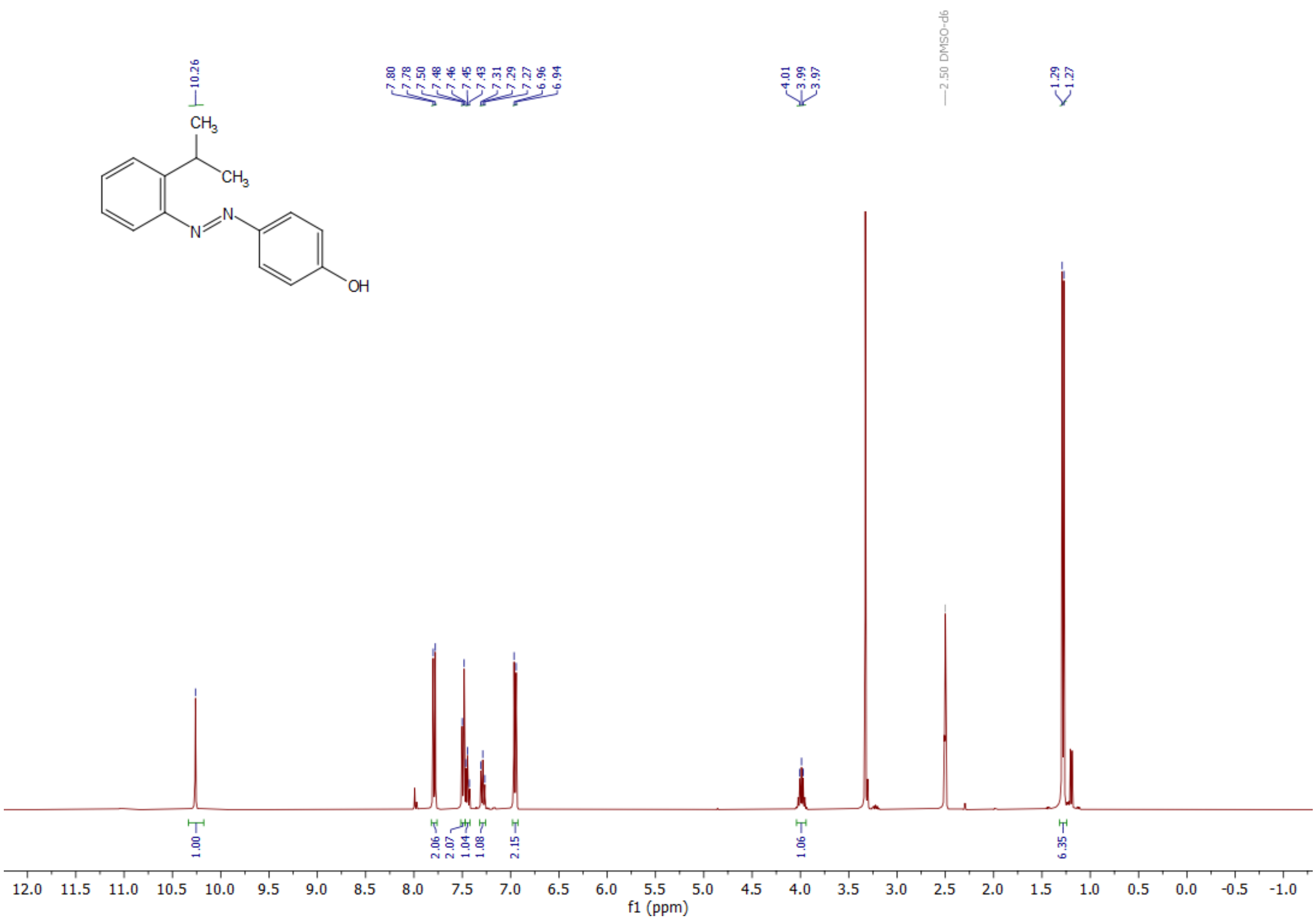

^{13}C-NMR of 4-(2-*iso*-propylphenyl)diazenylphenol (3d)

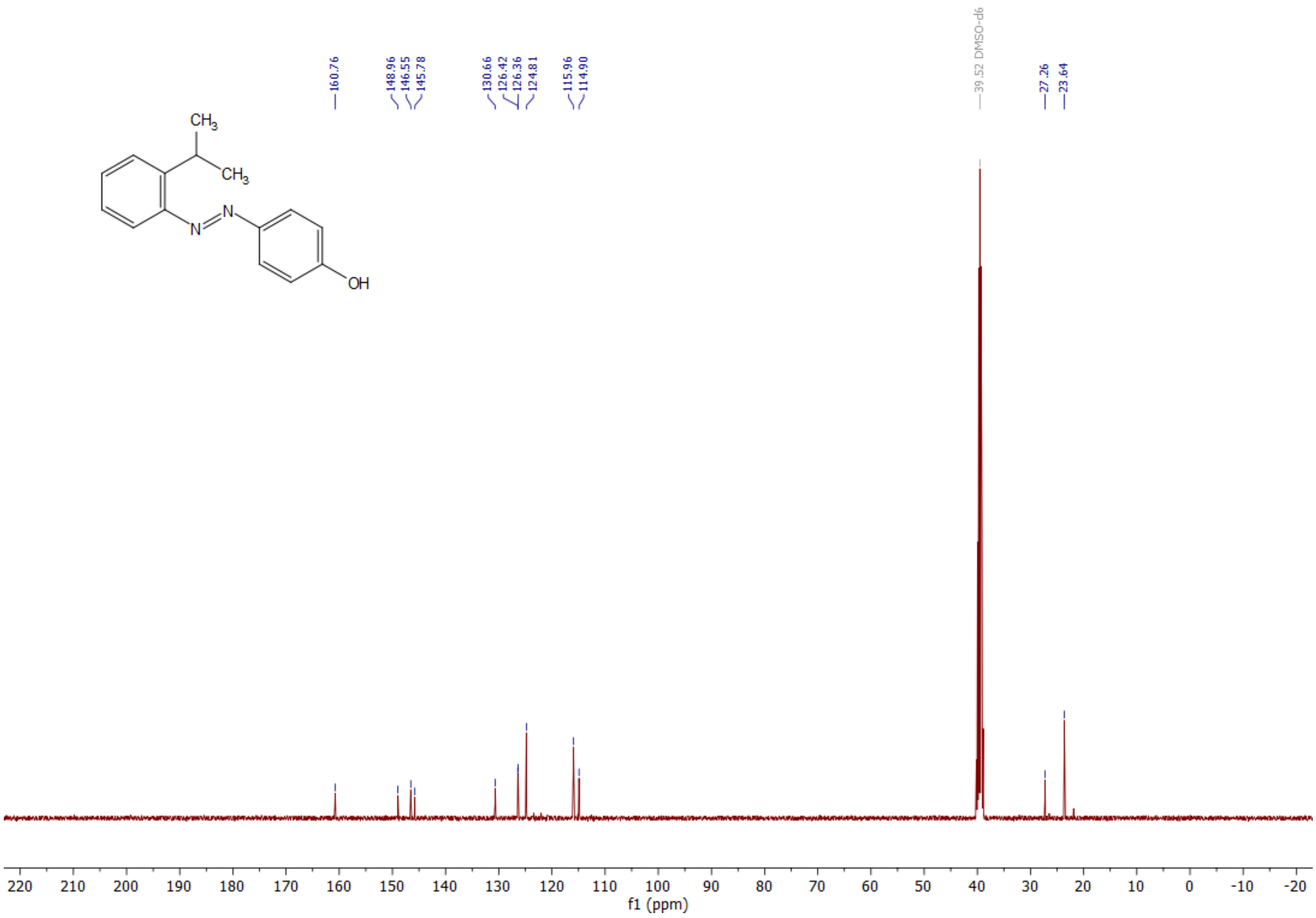

^{1}H-NMR of 4-(2-*tert*-butylphenyl)diazenylphenol (3e)

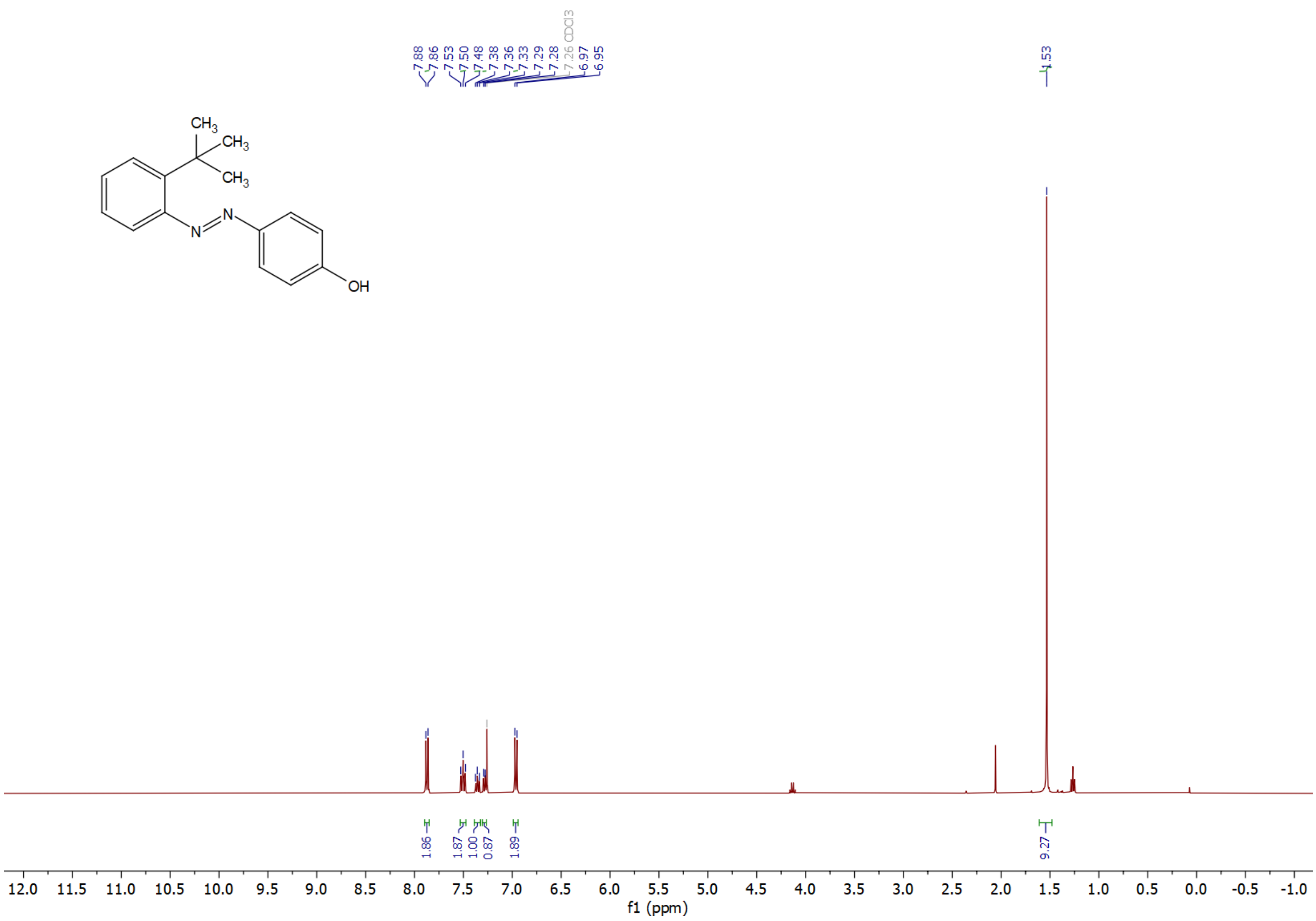

^{13}C-NMR of 4-(2-*tert*-butylphenyl)diazenylphenol (3e)

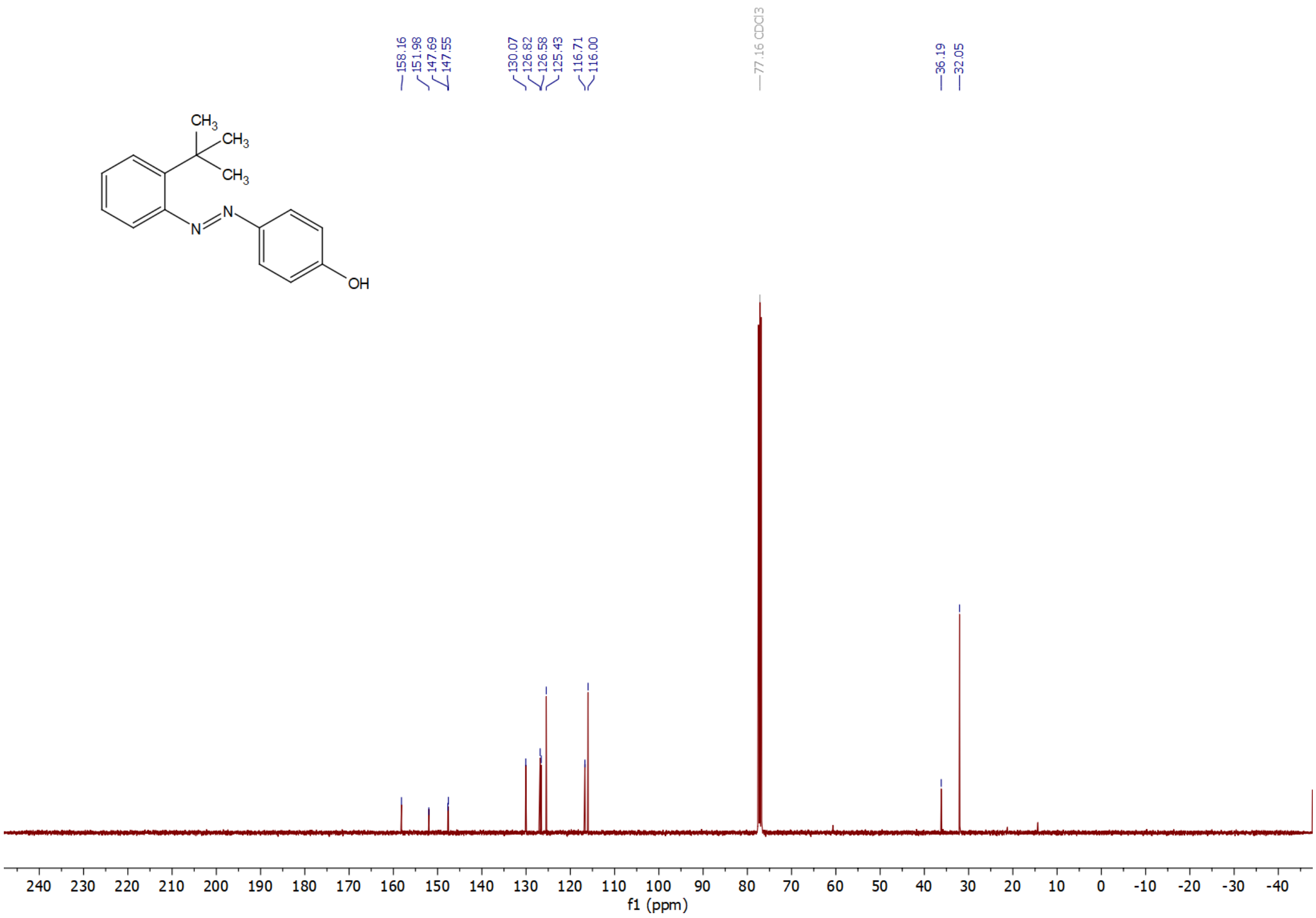

^{1}H-NMR of 4-(3-methylphenyl)diazenylphenol (3f)

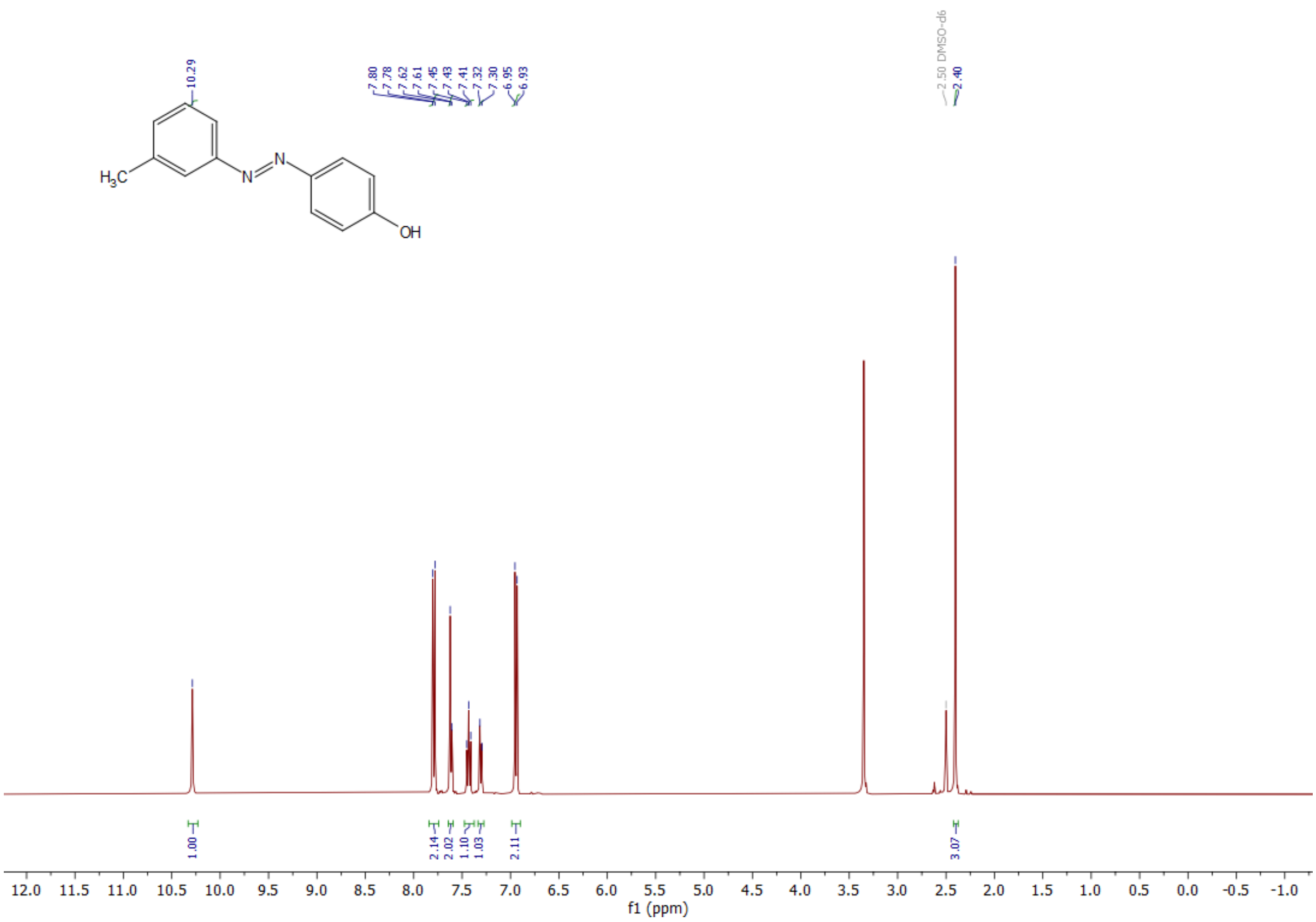

^{13}C-NMR of 4-(3-methylphenyl)diazenylphenol (3f)

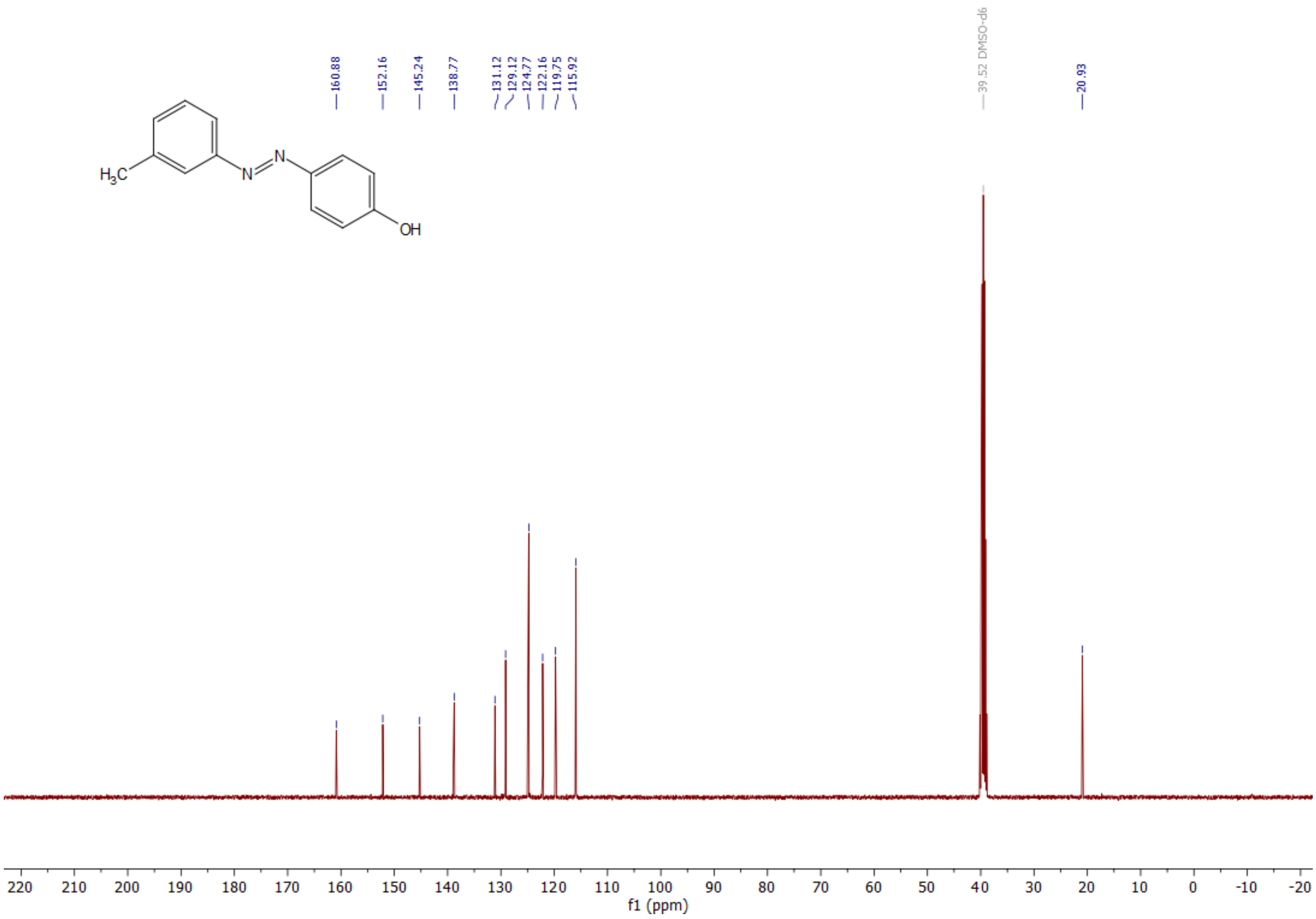

^{1}H-NMR of 4-(3-ethylphenyl)diazenylphenol (3g)

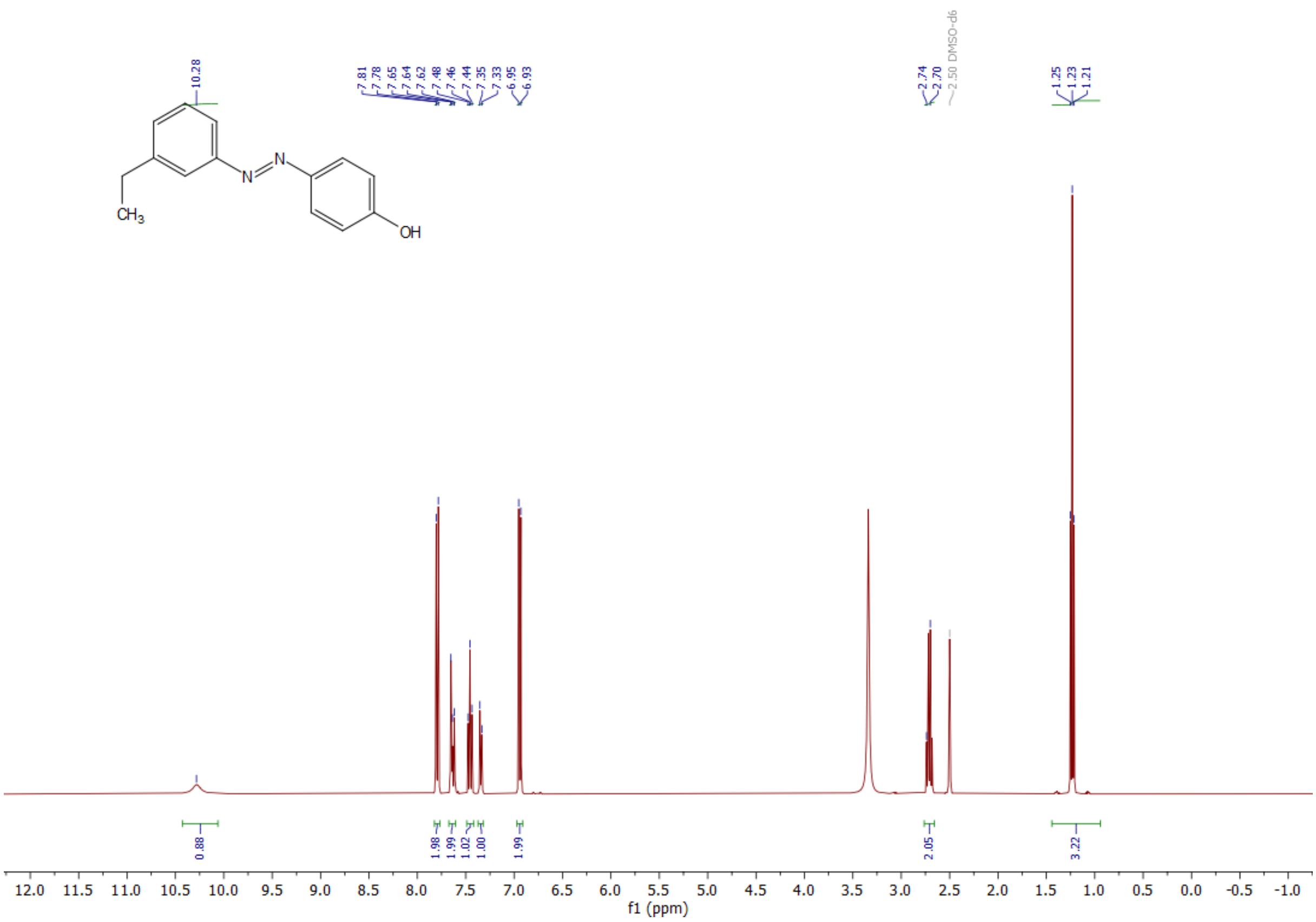

^{13}C-NMR of 4-(3-ethylphenyl)diazenylphenol (3g)

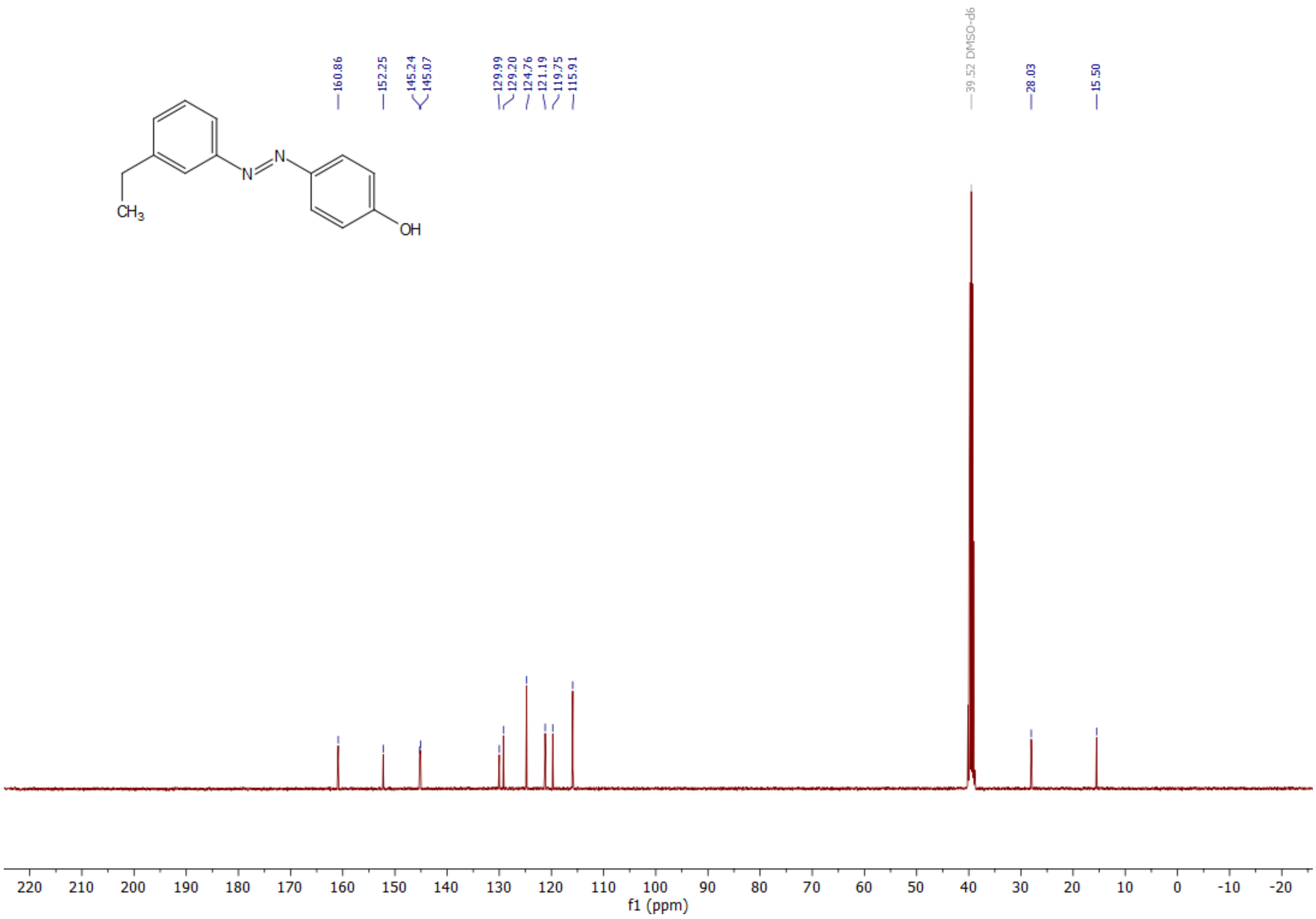

^{1}H-NMR of 4-(3-*iso*-propylphenyl)diazenylphenol (3h)

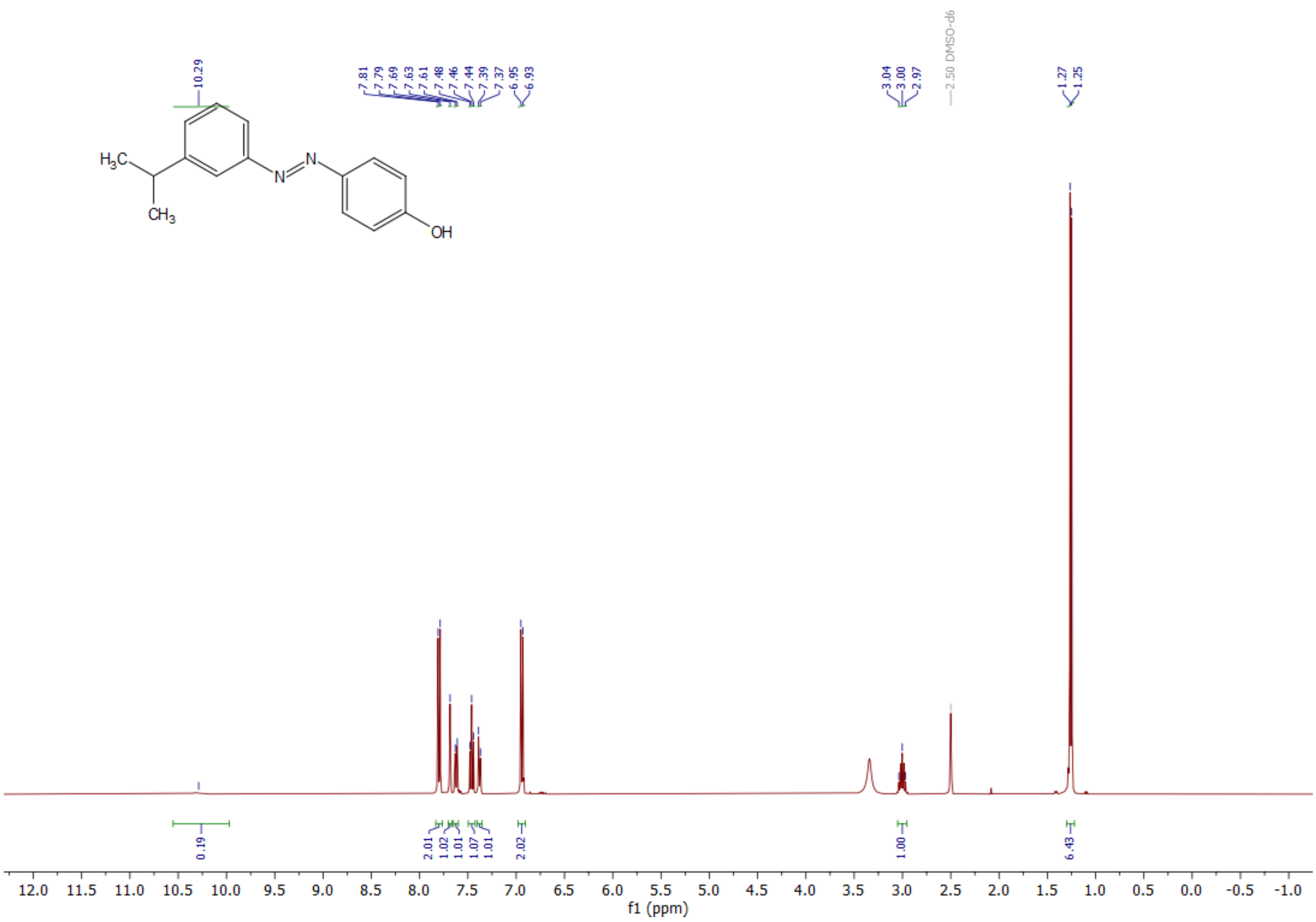

^{13}C-NMR of 4-(3-*iso*-propylphenyl)diazenylphenol (3h)

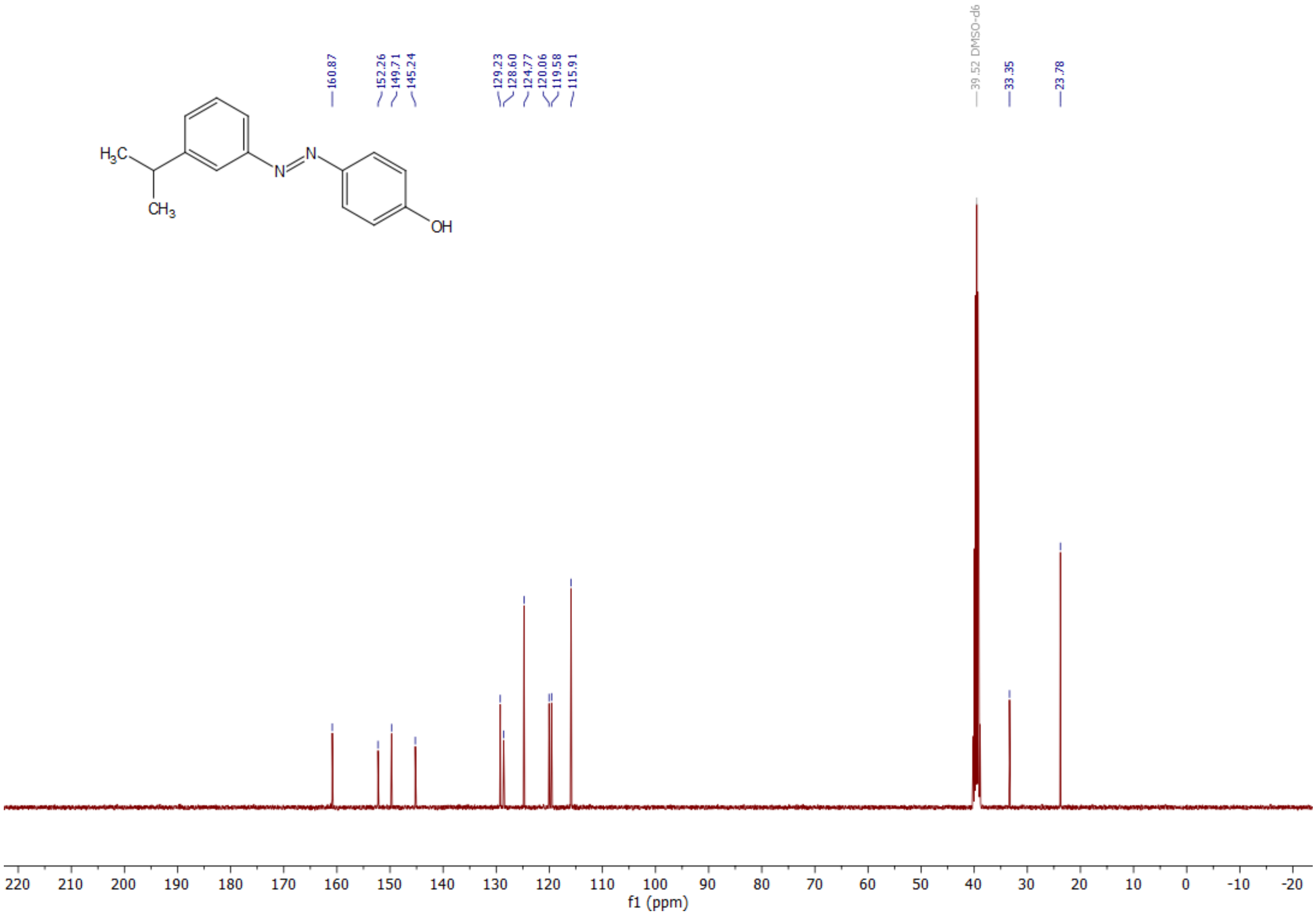

^{1}H-NMR of 4-(3-*tert*-butylphenyl)diazenylphenol (3i)

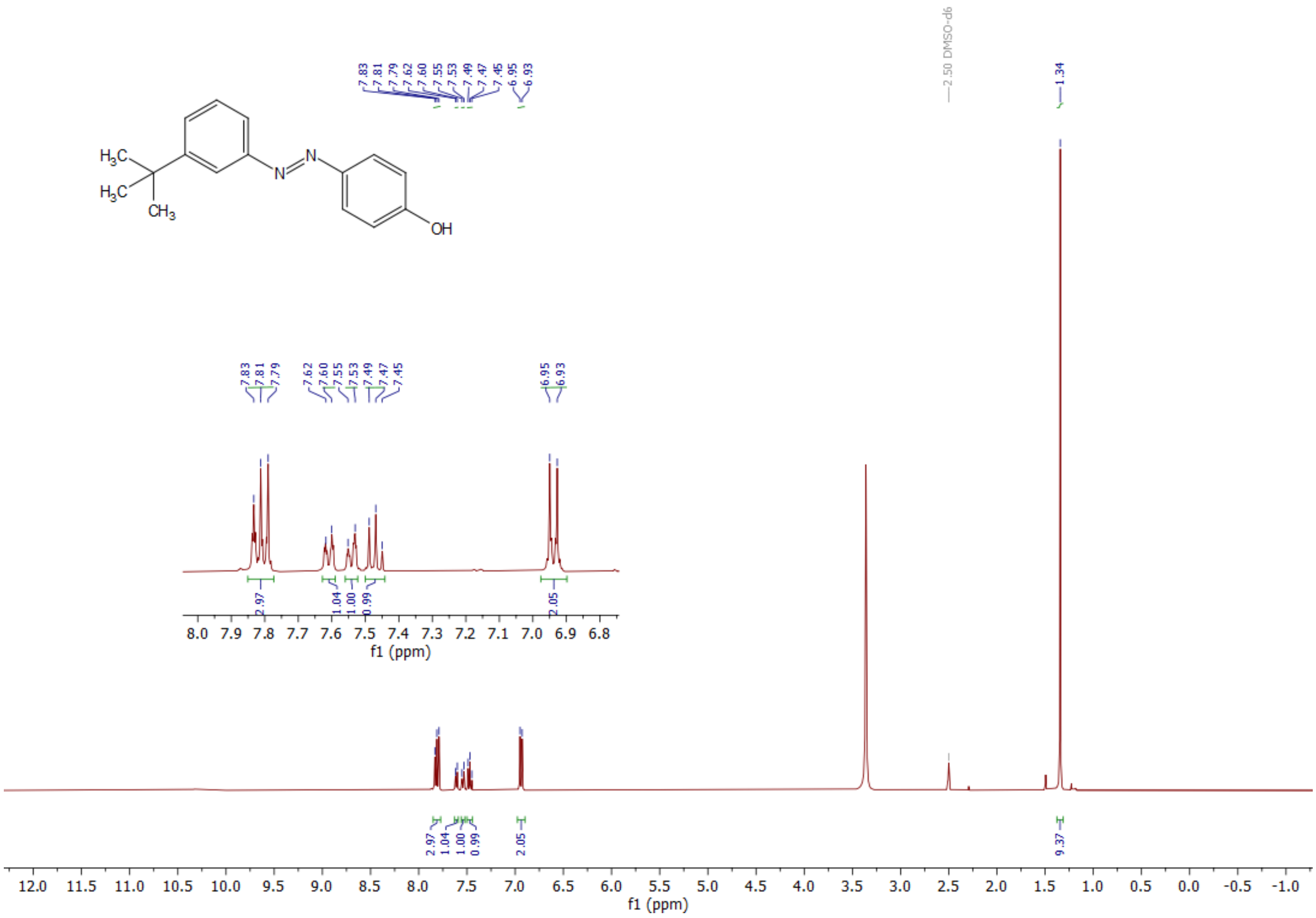

^{13}C-NMR of 4-(3-*tert*-butylphenyl)diazenylphenol (3i)

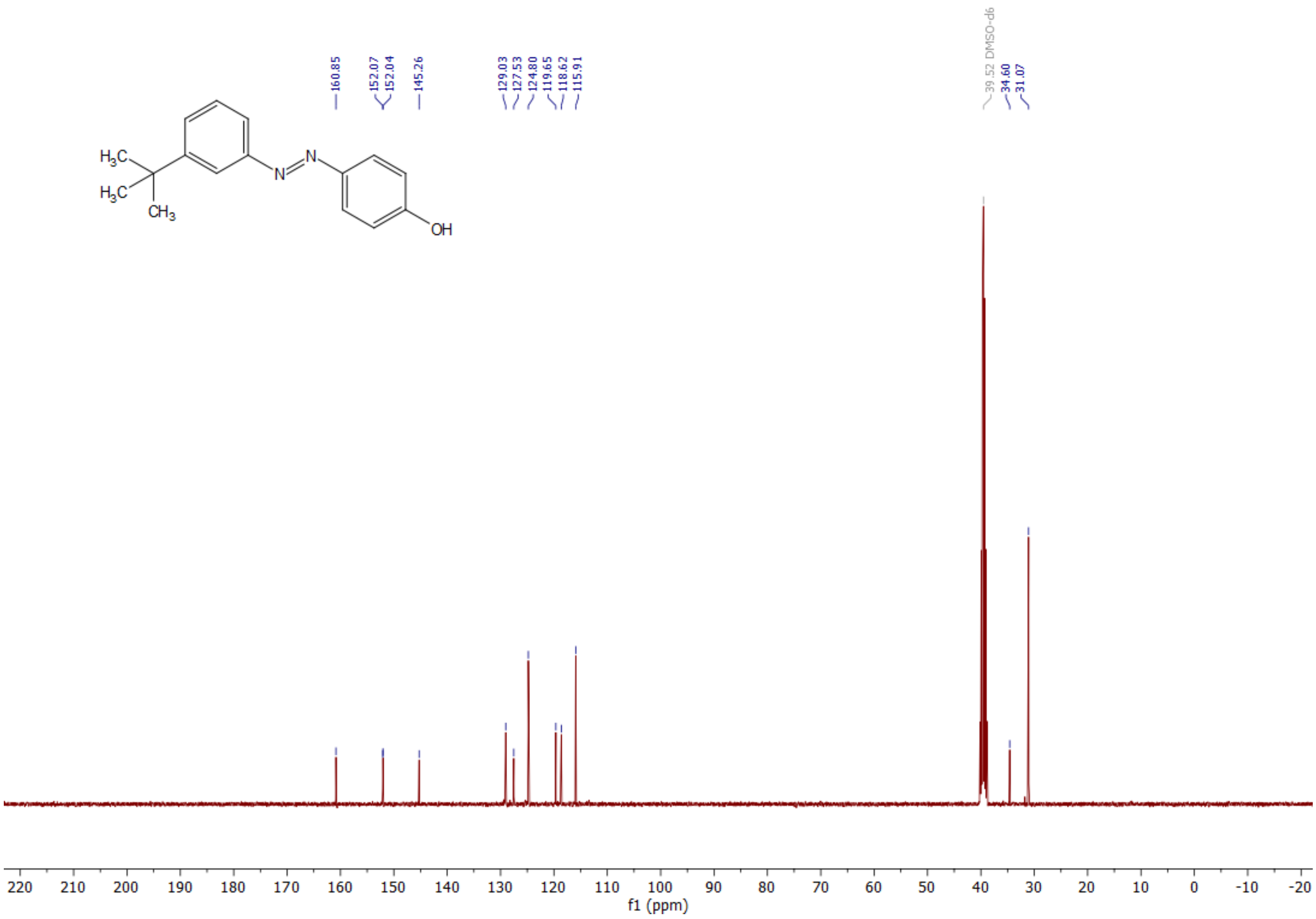

^{1}H-NMR of 4-(4-methylphenyl)diazenylphenol (3j)

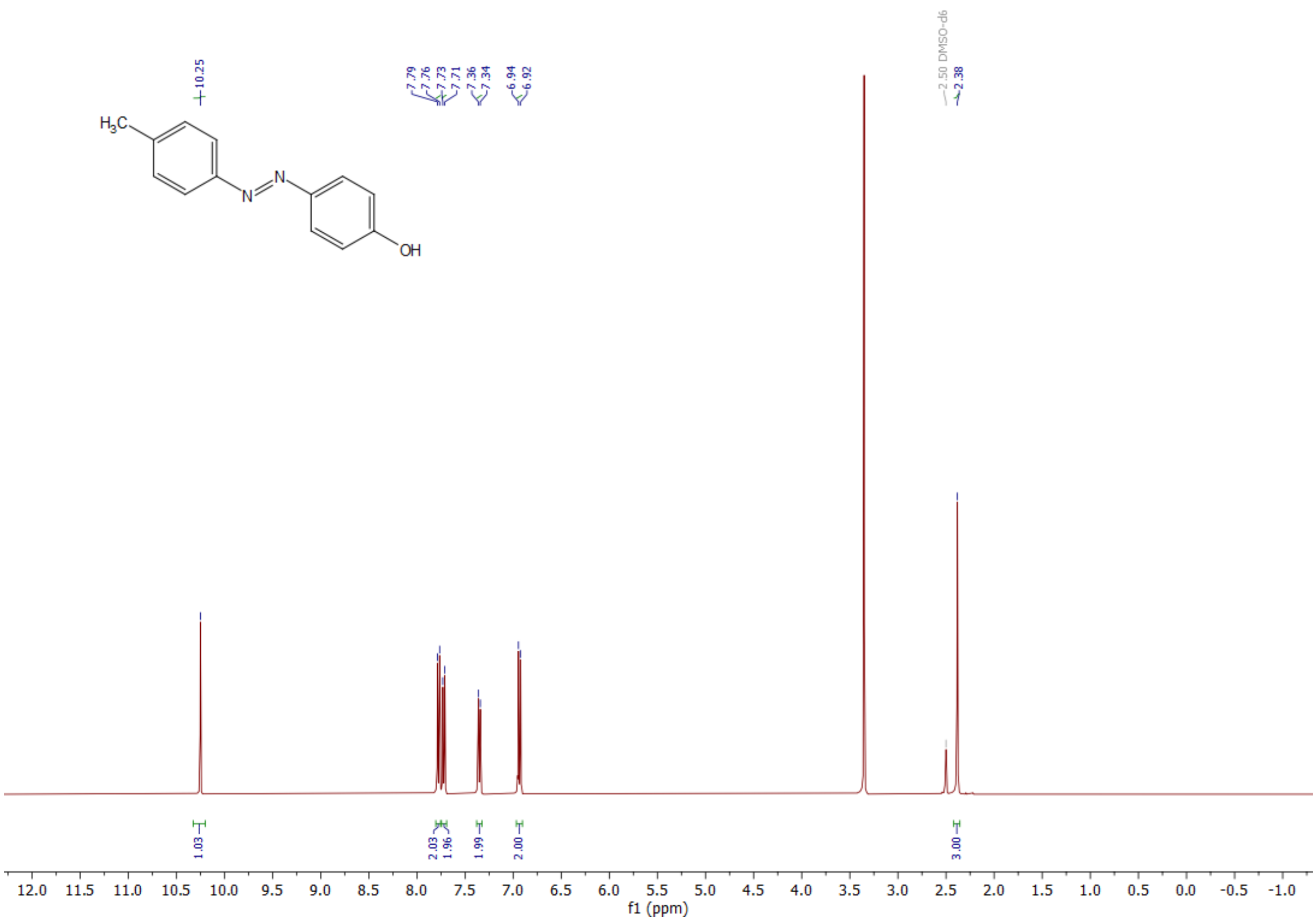

^{13}C-NMR of 4-(4-methylphenyl)diazenylphenol (3j)

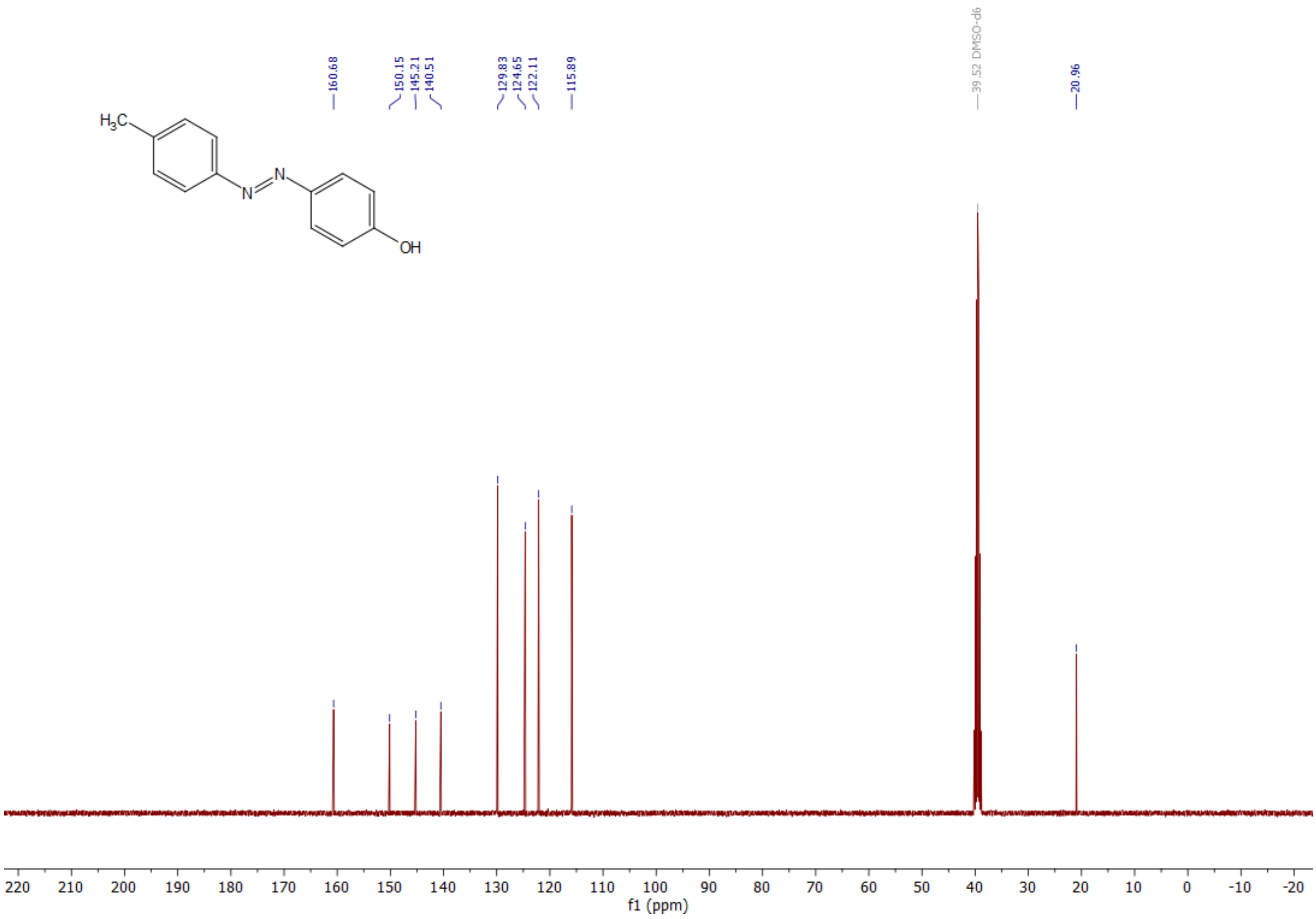

^{1}H-NMR of 4-(4-ethylphenyl)diazenylphenol (3k)

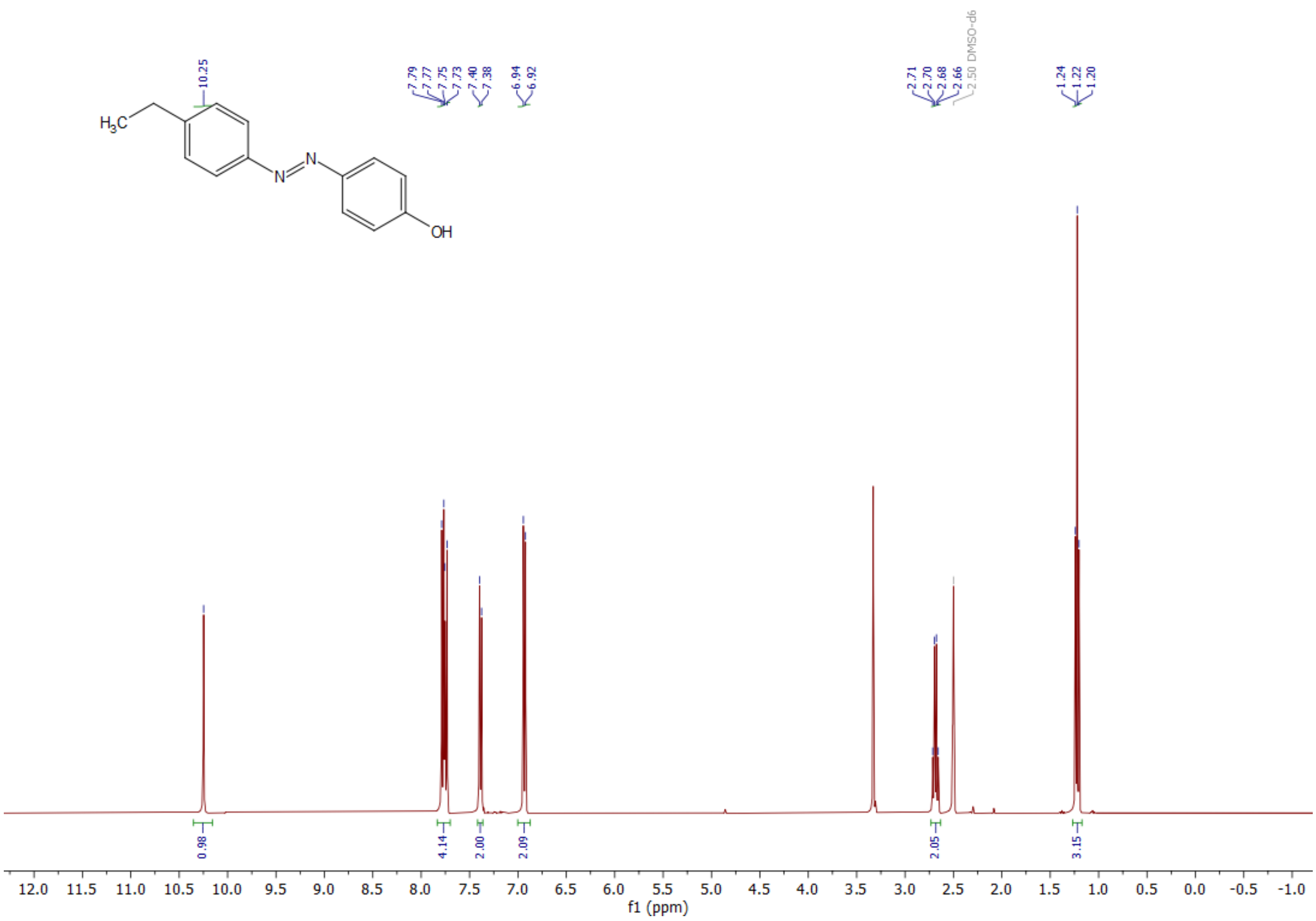

^{13}C-NMR of 4-(4-ethylphenyl)diazenylphenol (3k)

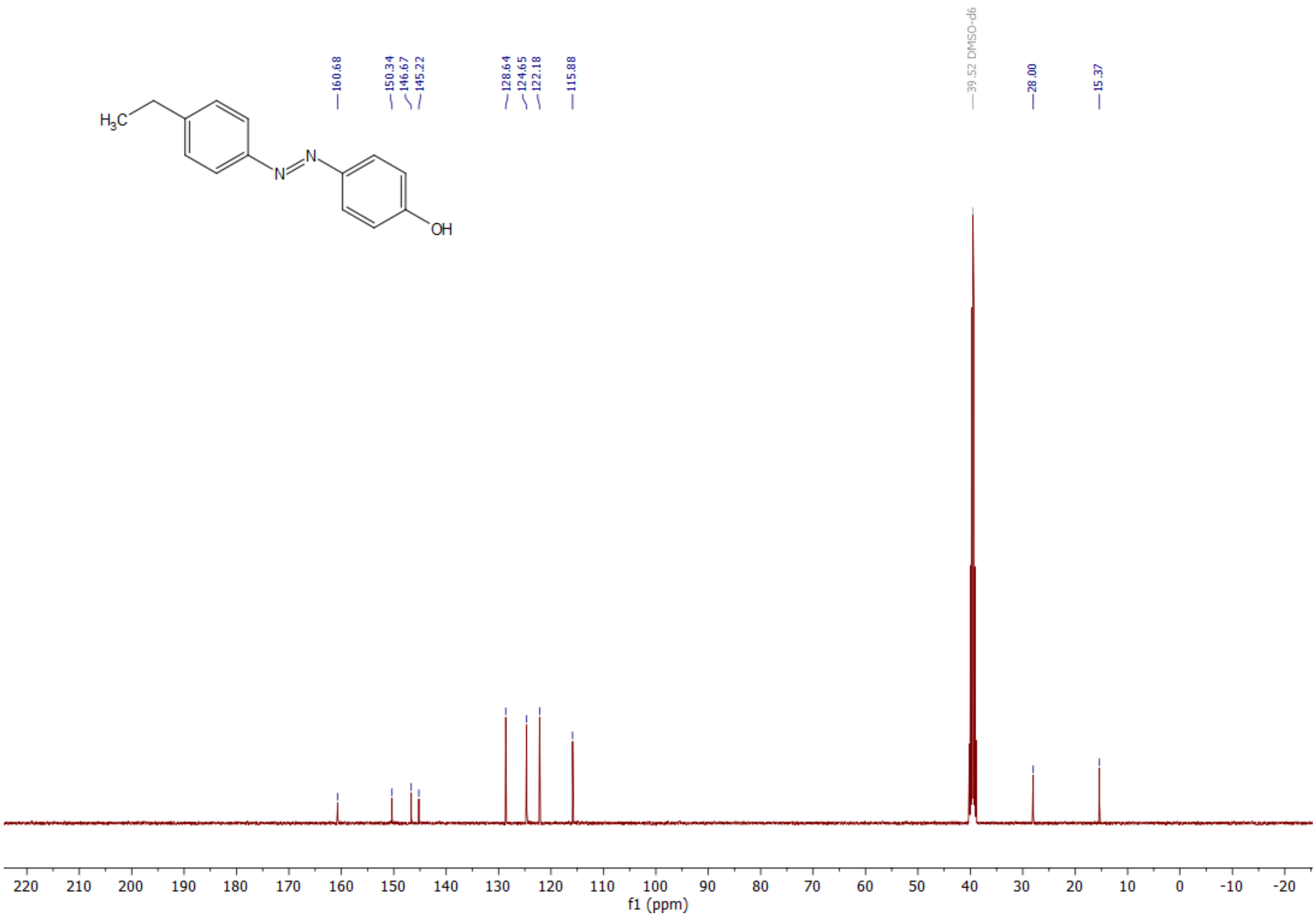

^{1}H-NMR of 4-(4-*iso*-propylphenyl)diazenylphenol (3l)

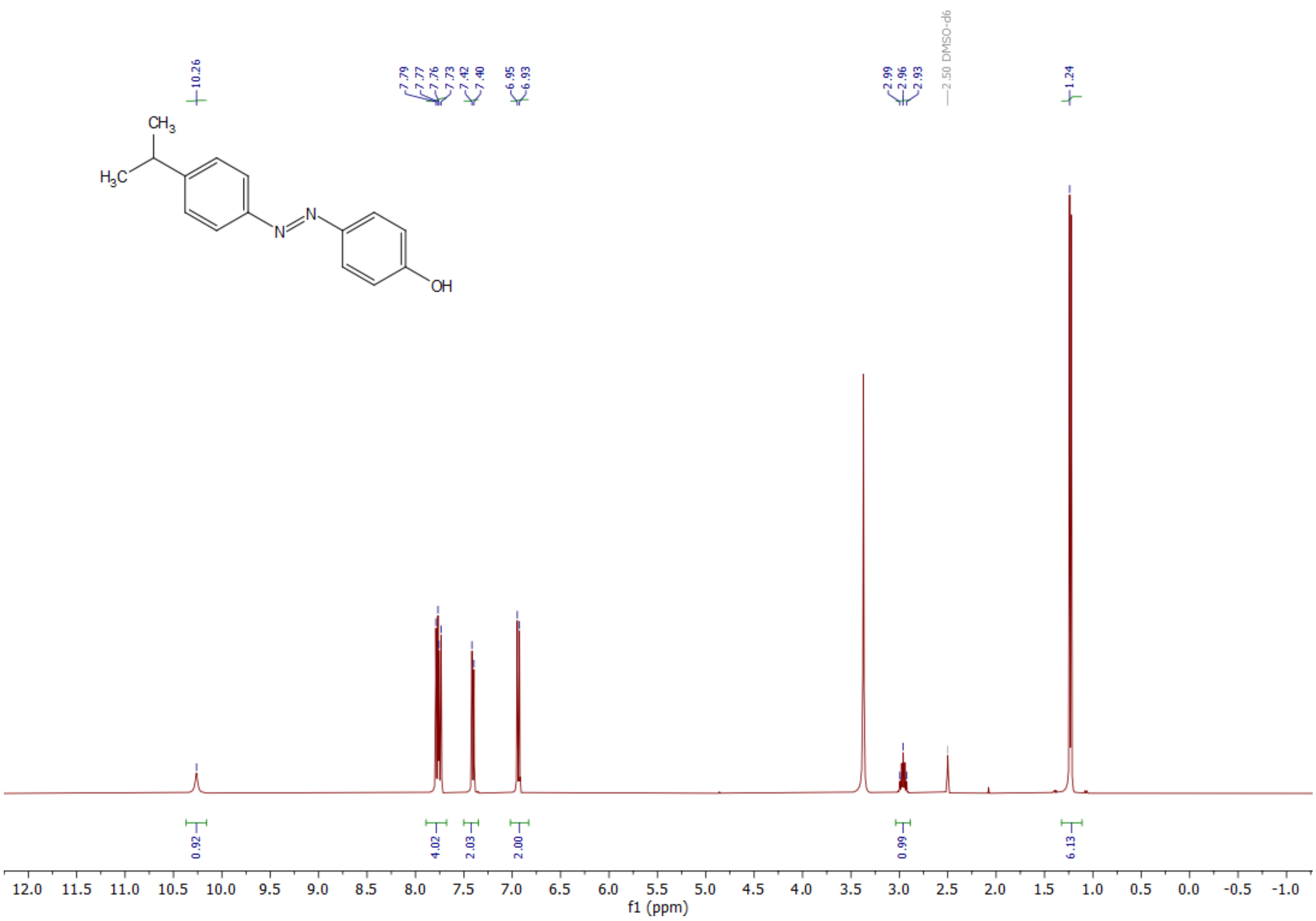

^{13}C-NMR of 4-(4-*iso*-propylphenyl)diazenylphenol (3l)

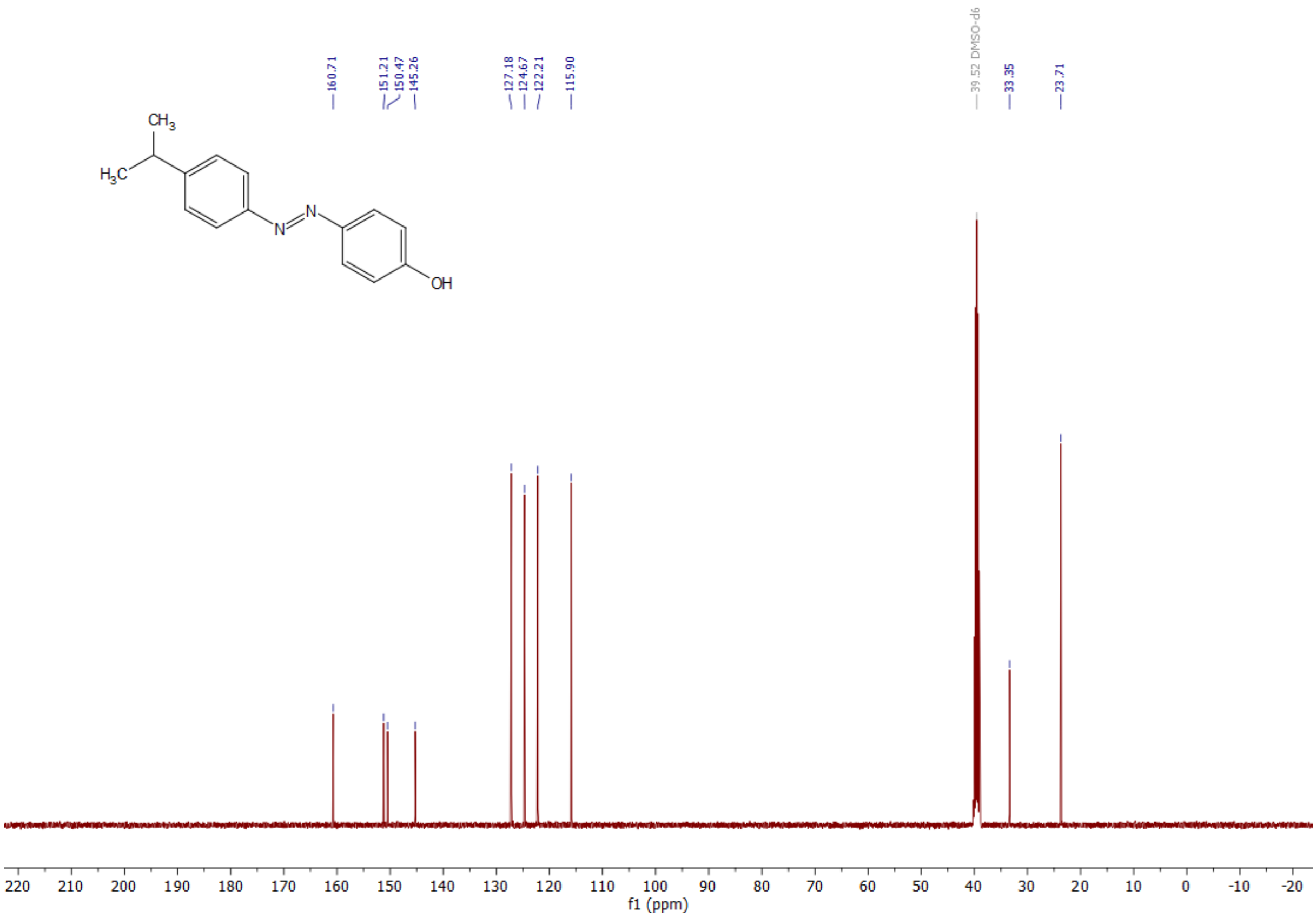

^{1}H-NMR of 4-(4-*tert*-butylphenyl)diazenylphenol (3m)

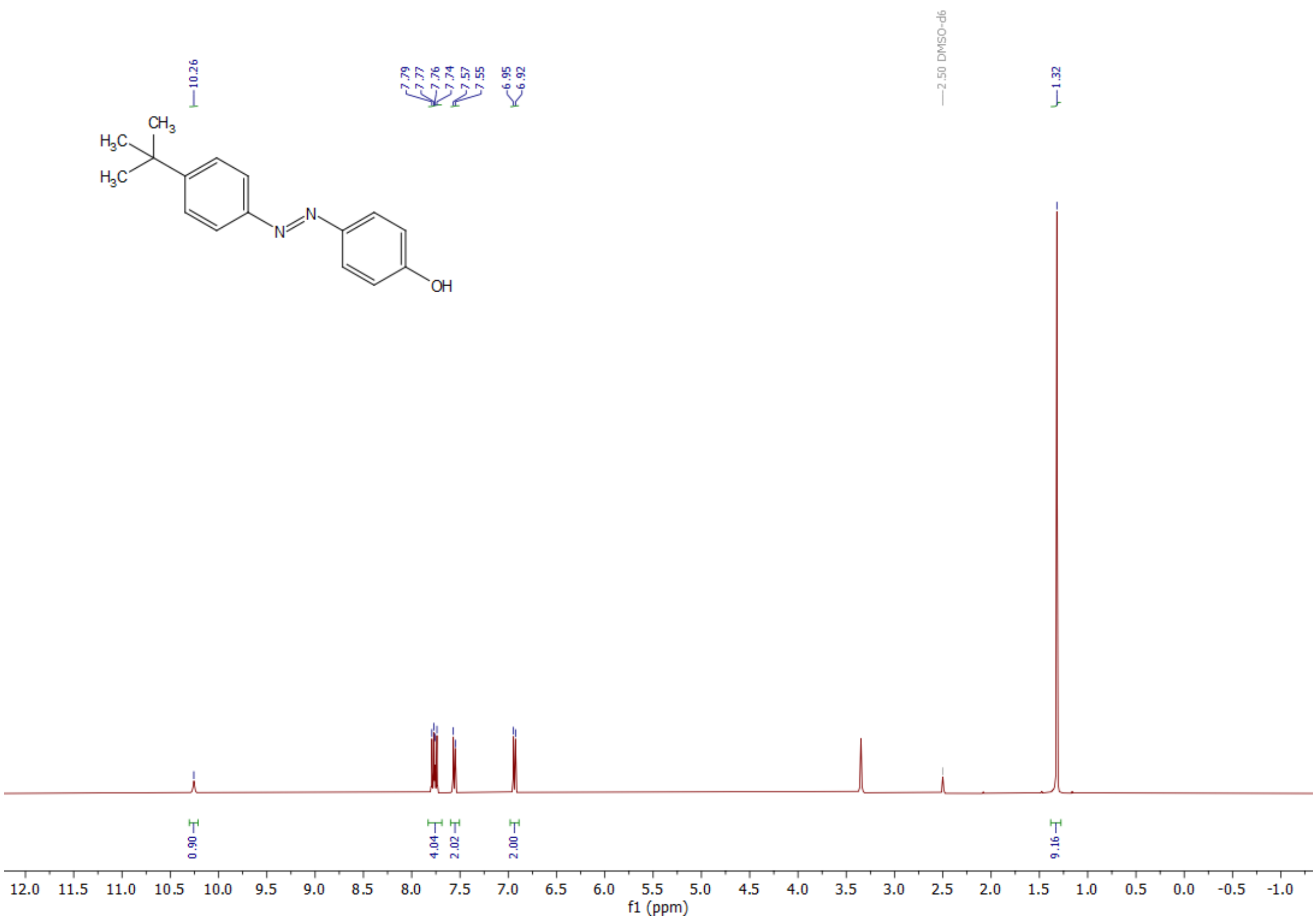

^{13}C-NMR of 4-(4-*tert*-butylphenyl)diazenylphenol (3m)

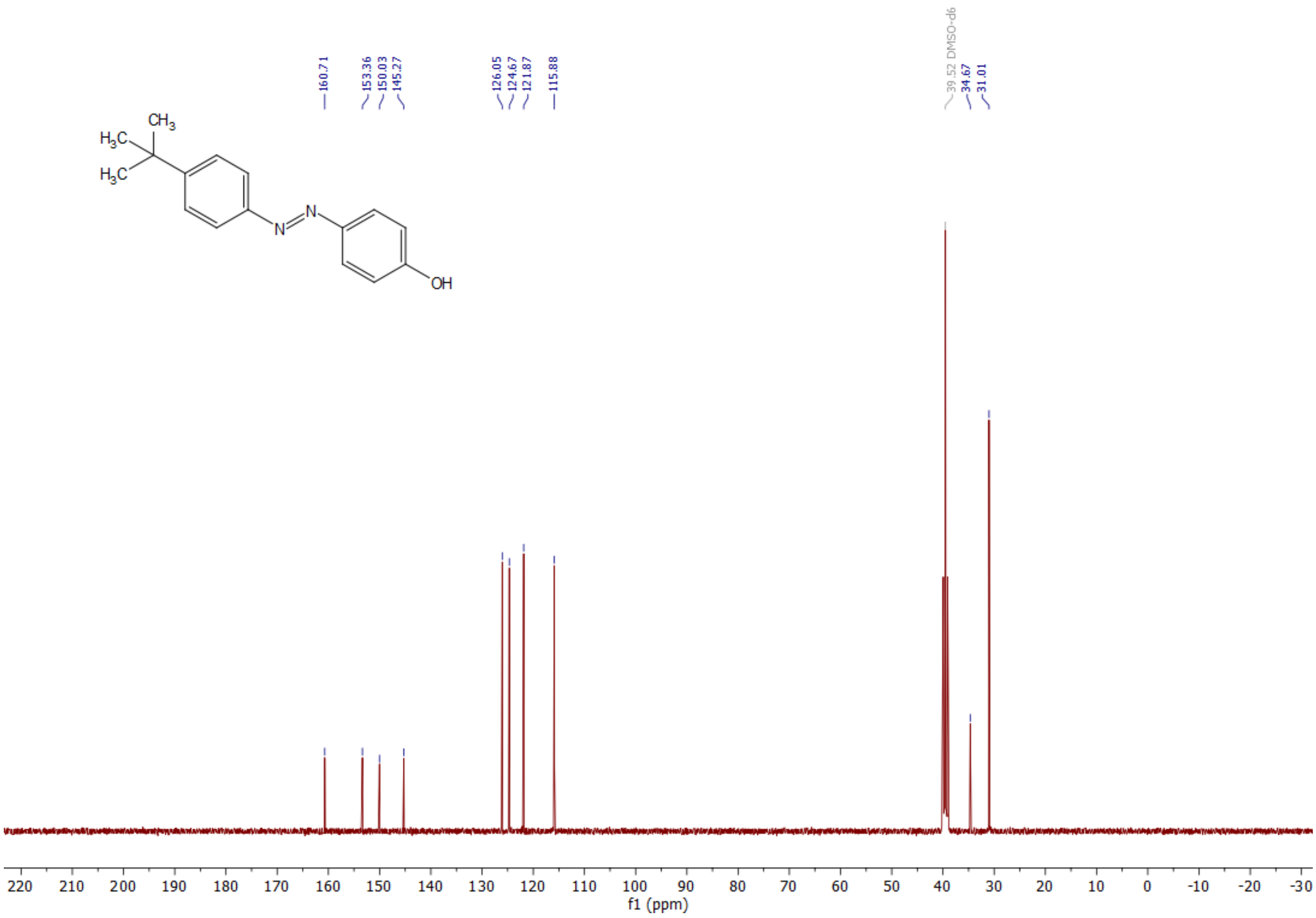

^{1}H-NMR of 4-(2,6-dimethylphenyl)diazenylphenol (3n)

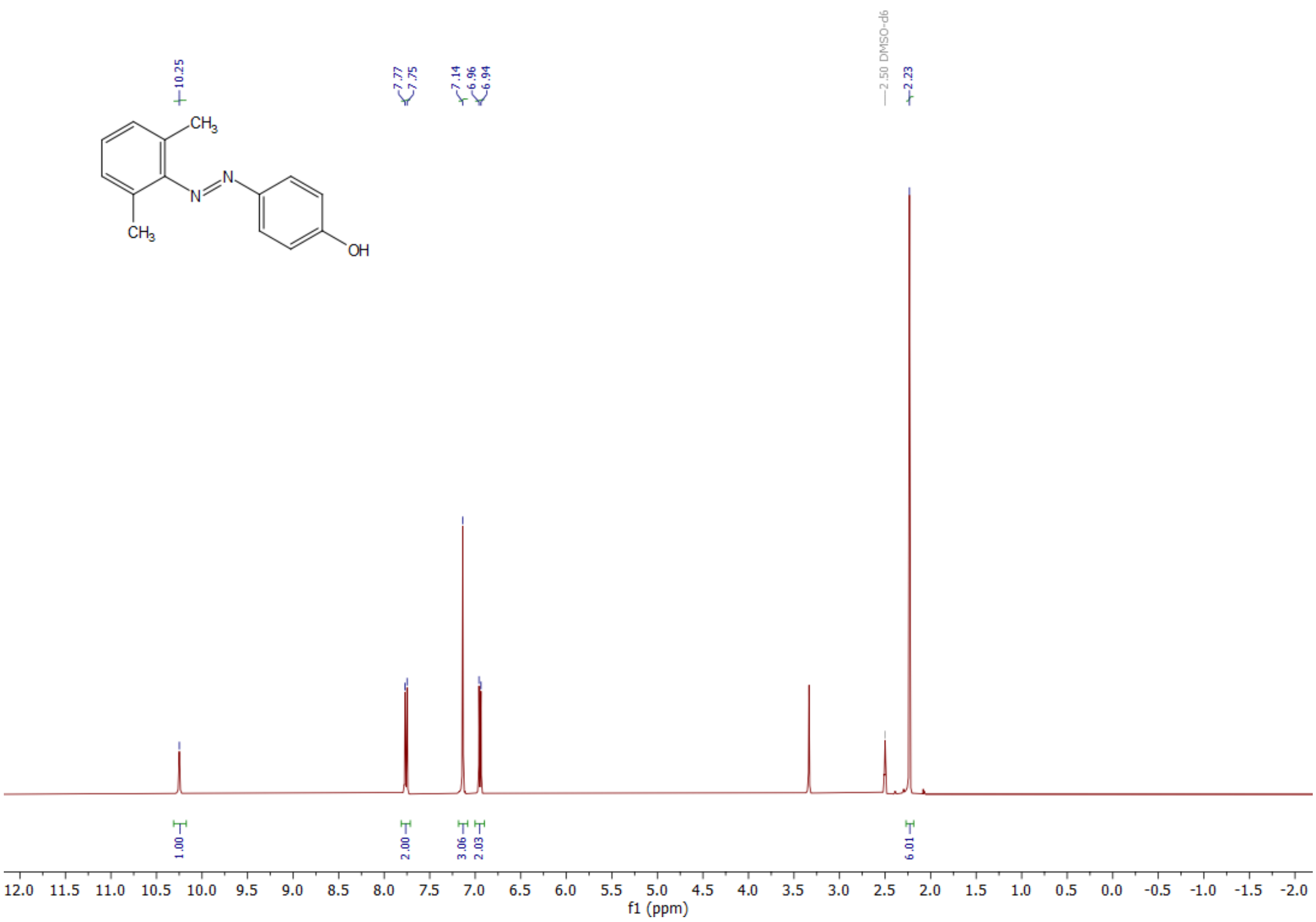

^{13}C-NMR of 4-(2,6-dimethylphenyl)diazenylphenol (3n)

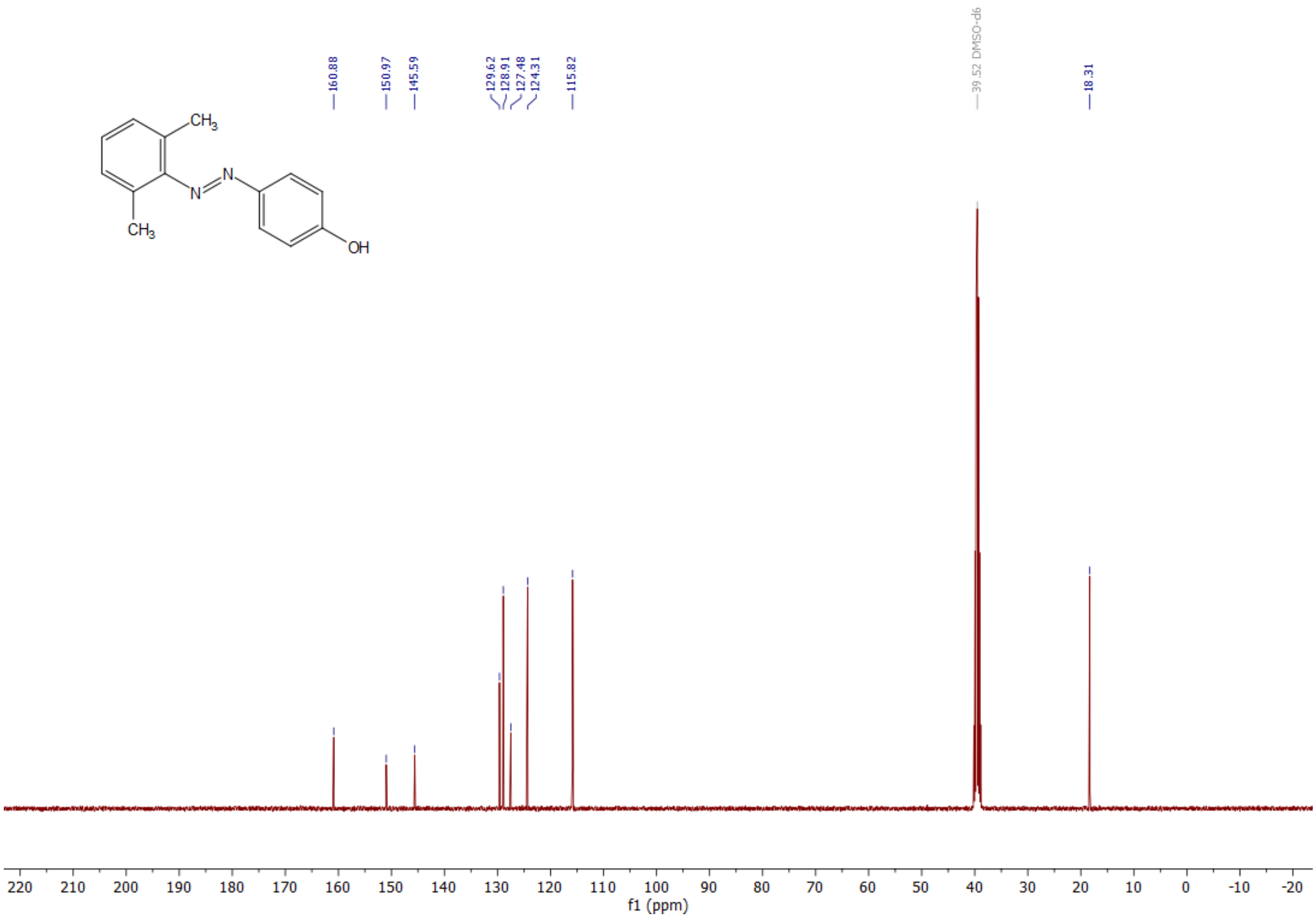

^{1}H-NMR of 4-(2,4,6-trimethylphenyl)diazenylphenol (3o)

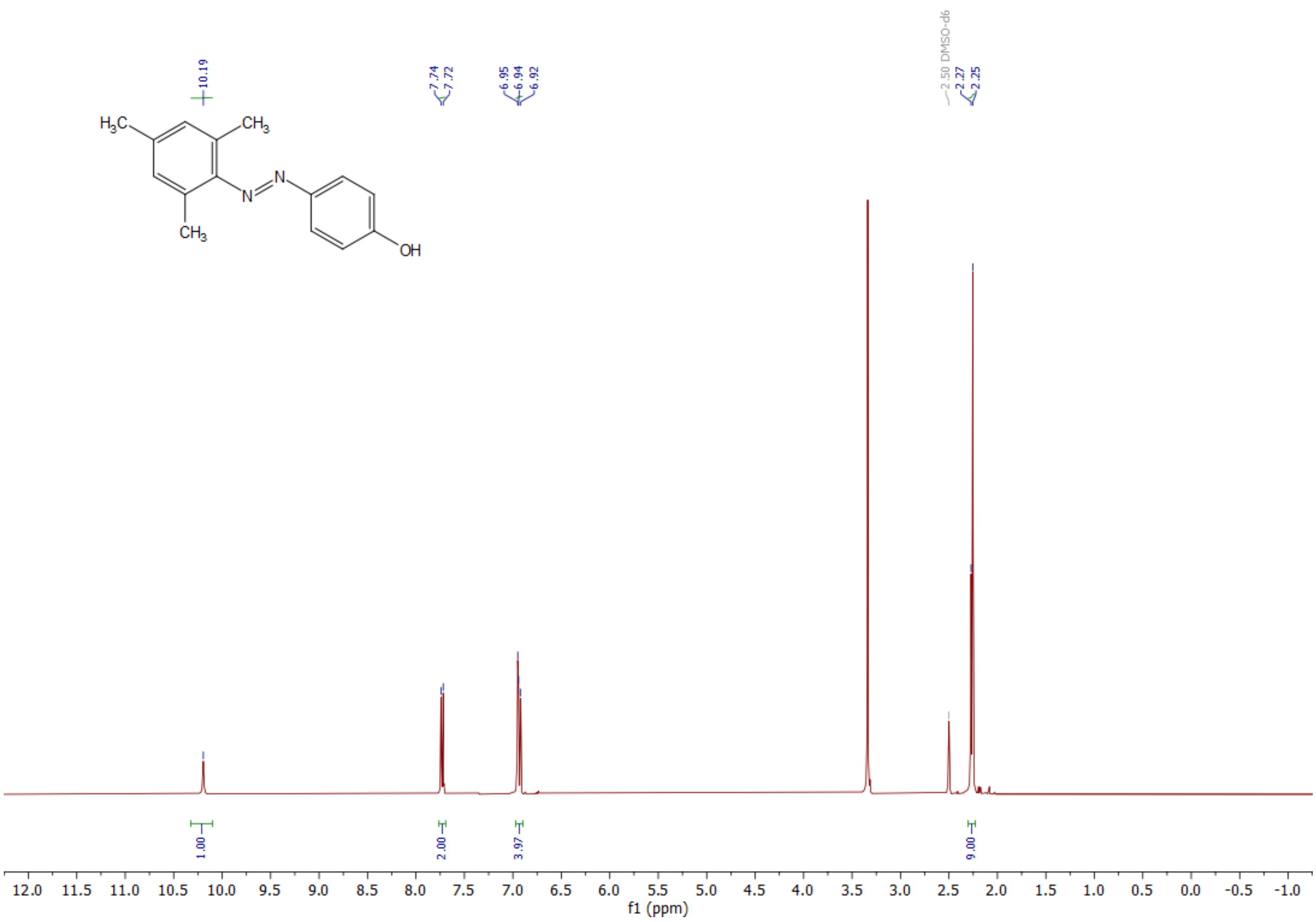

^{13}C-NMR of 4-(2,4,6-trimethylphenyl)diazenylphenol (3o)

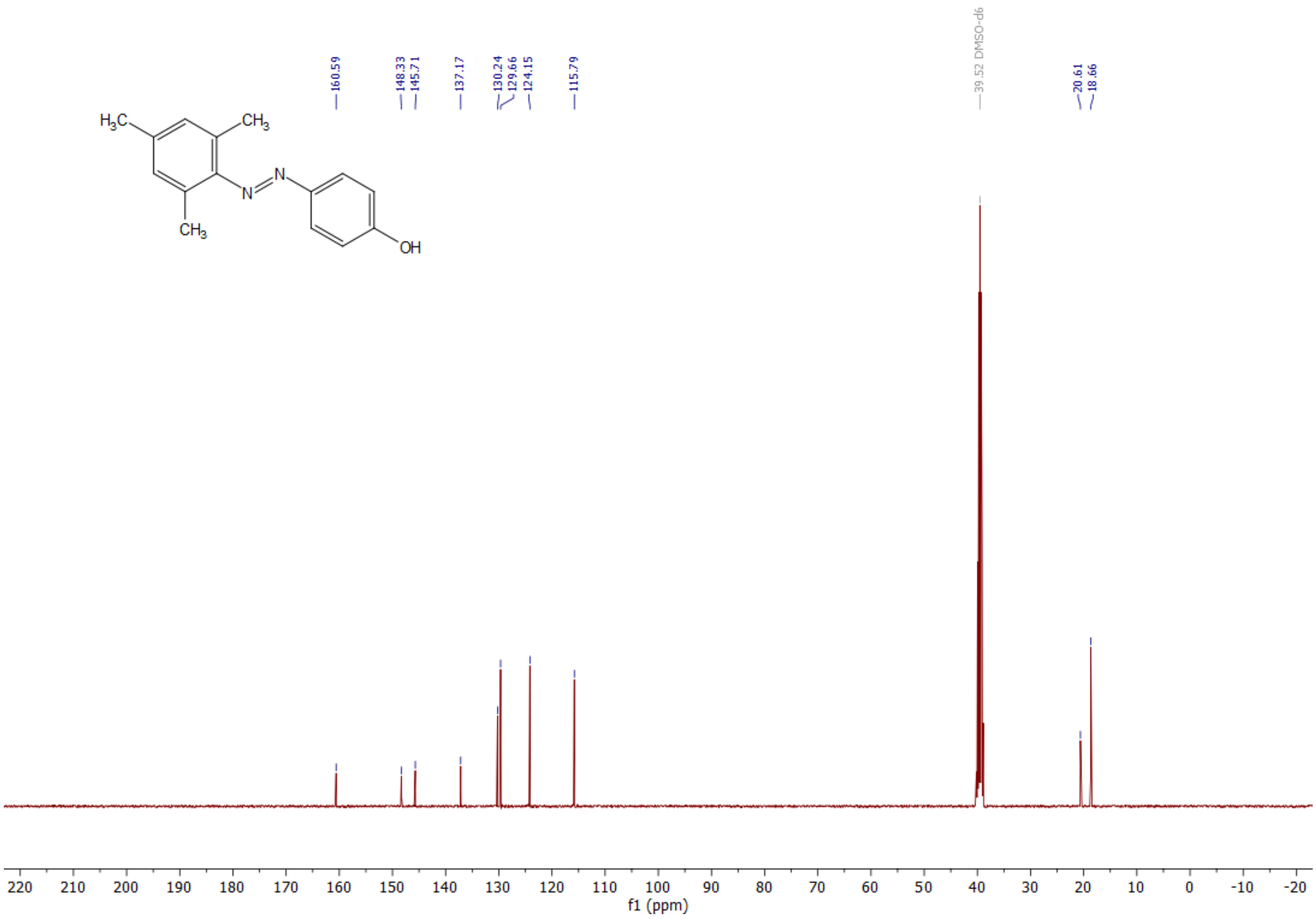

^{1}H-NMR of 4-(3,5-dimethylphenyl)diazenylphenol (3p)

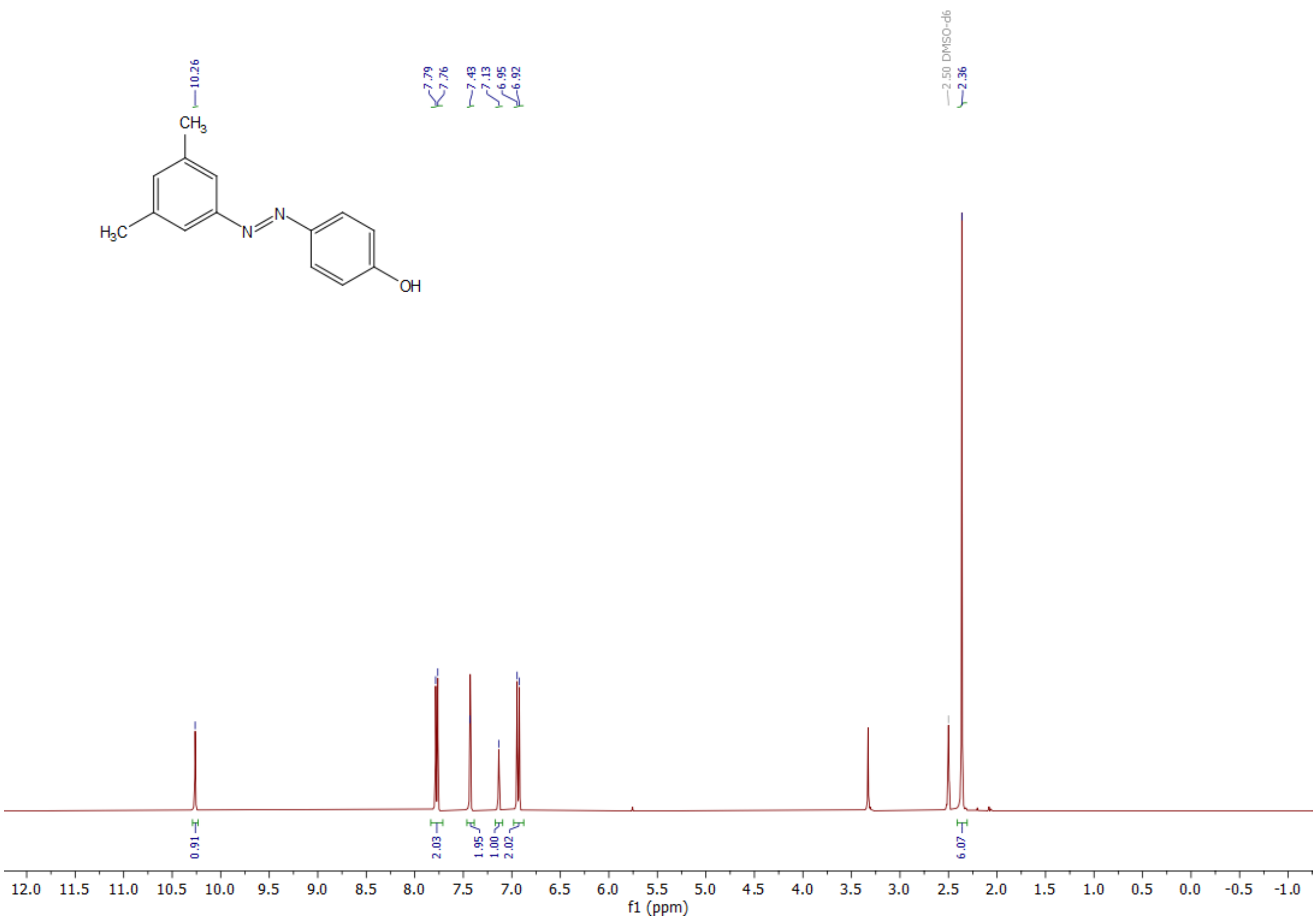

^{13}C-NMR of 4-(3,5-dimethylphenyl)diazenylphenol (3p)

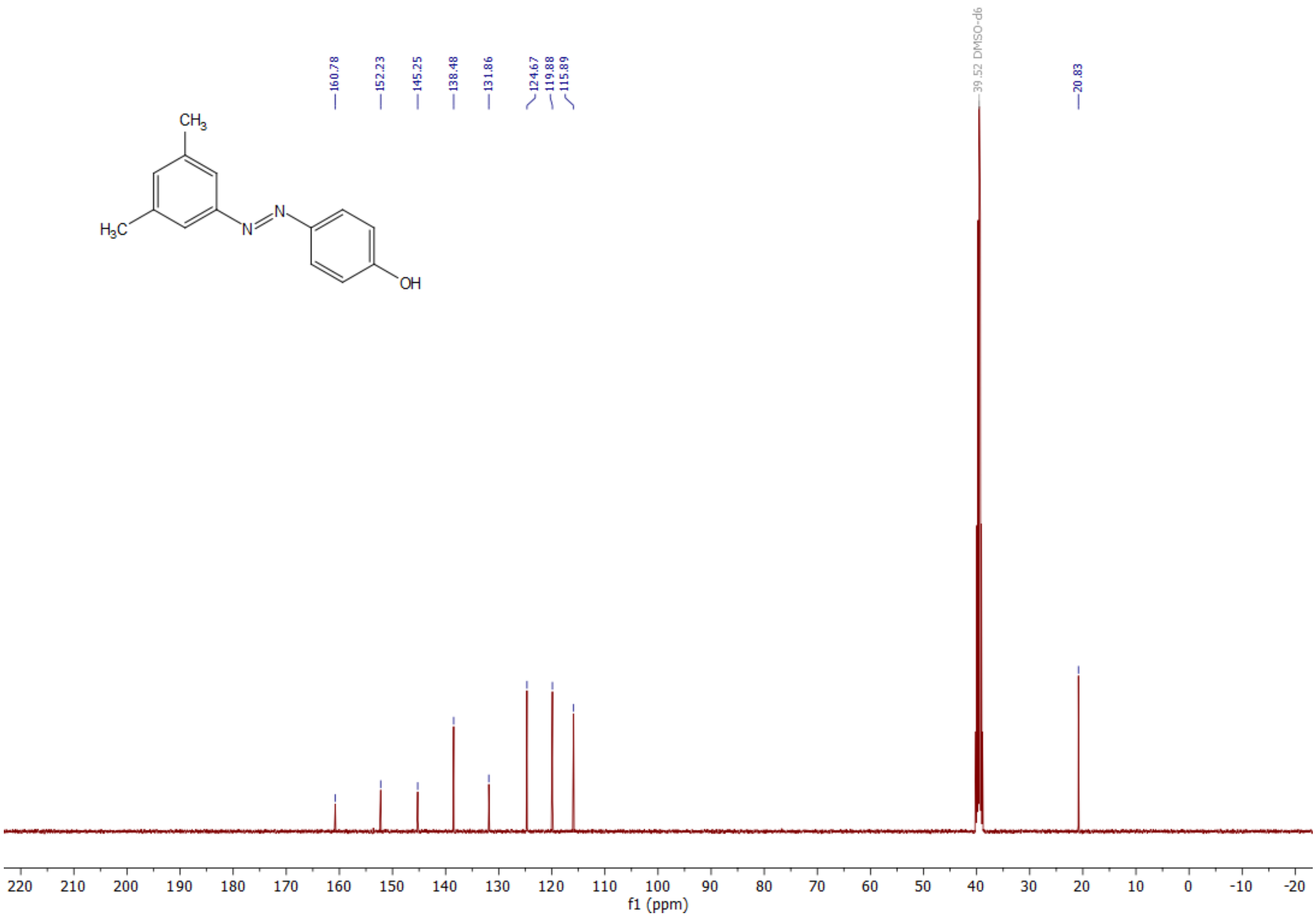

^{1}H-NMR of 4-(2,6-difluorophenyl)diazenylphenol (3q)

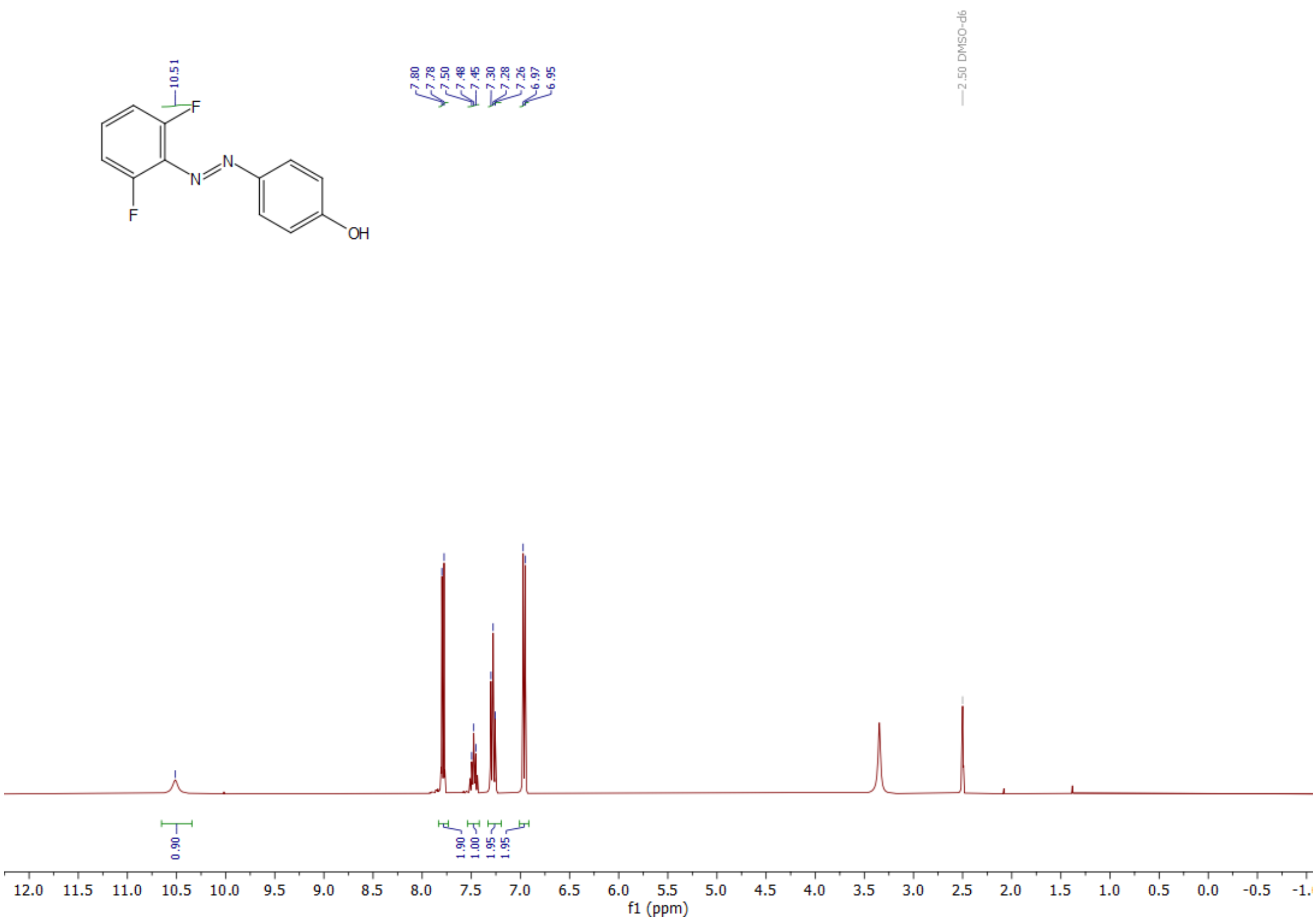

¹H-NMR of 4-(2,6- difluorophenyl)diazenylphenol (3q)

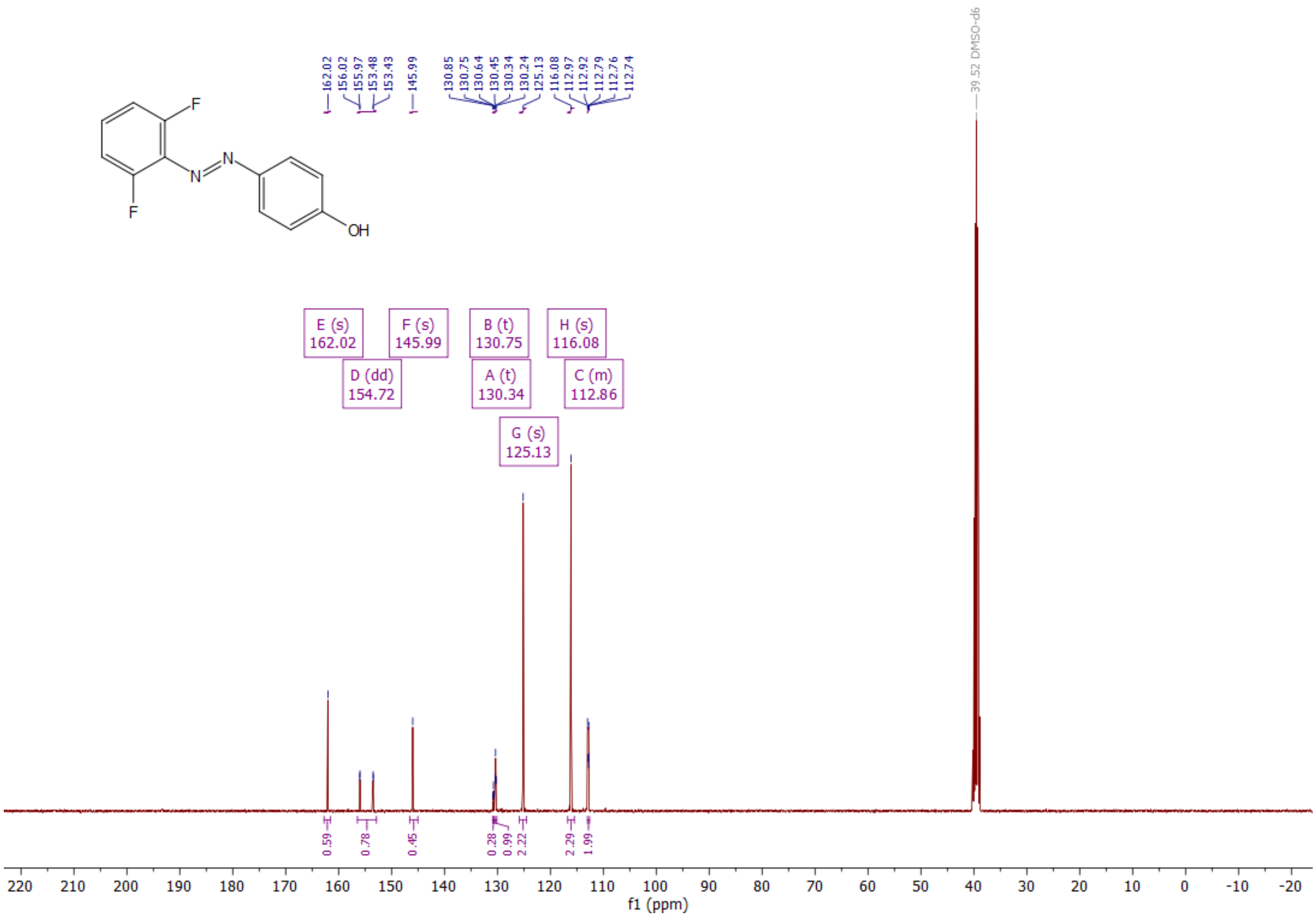

¹H-NMR of 4-(3-methoxyphenyl)diazenylphenol (3r)

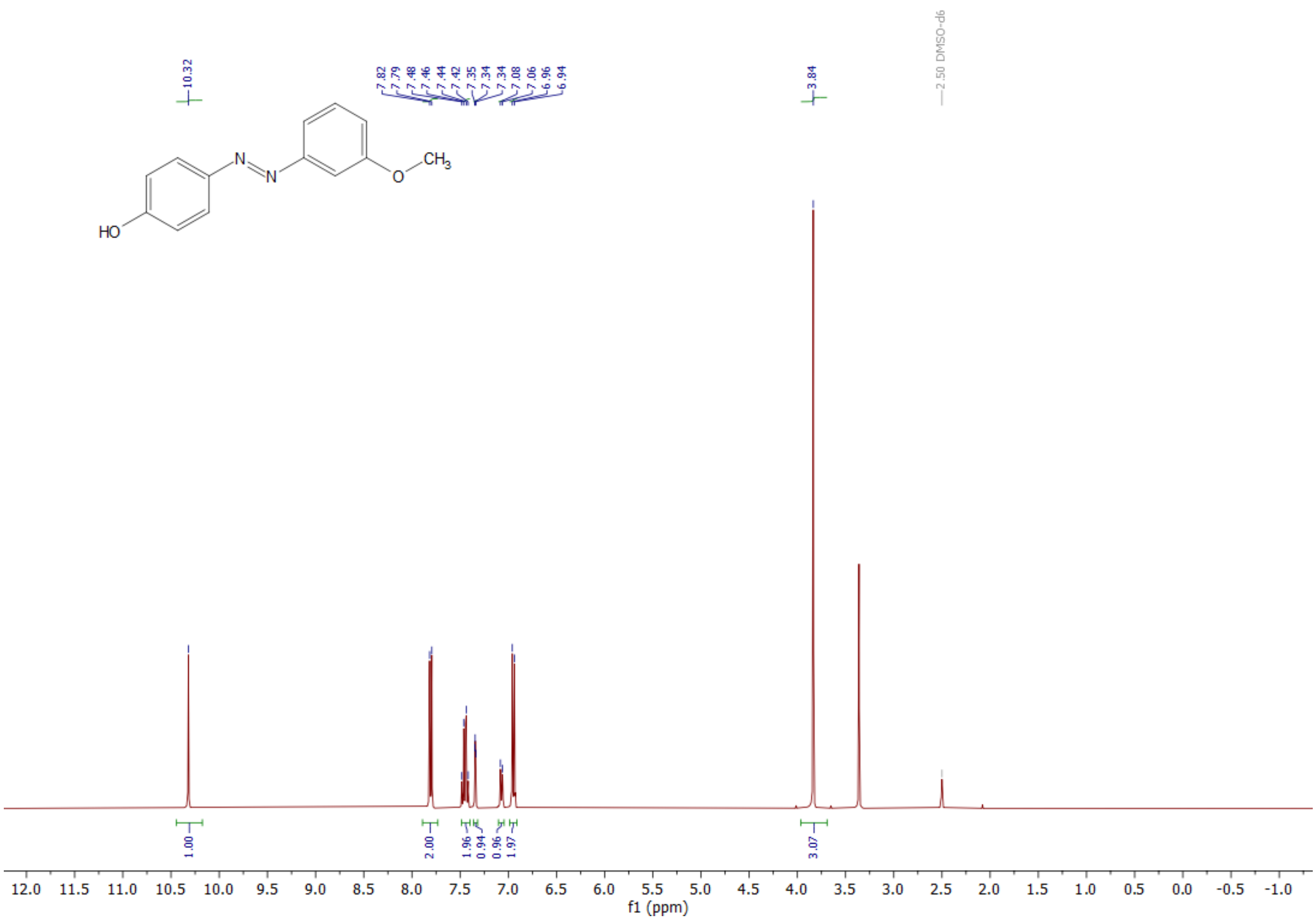

^{13}C-NMR of 4-(3-methoxyphenyl)diazenylphenol (3r)

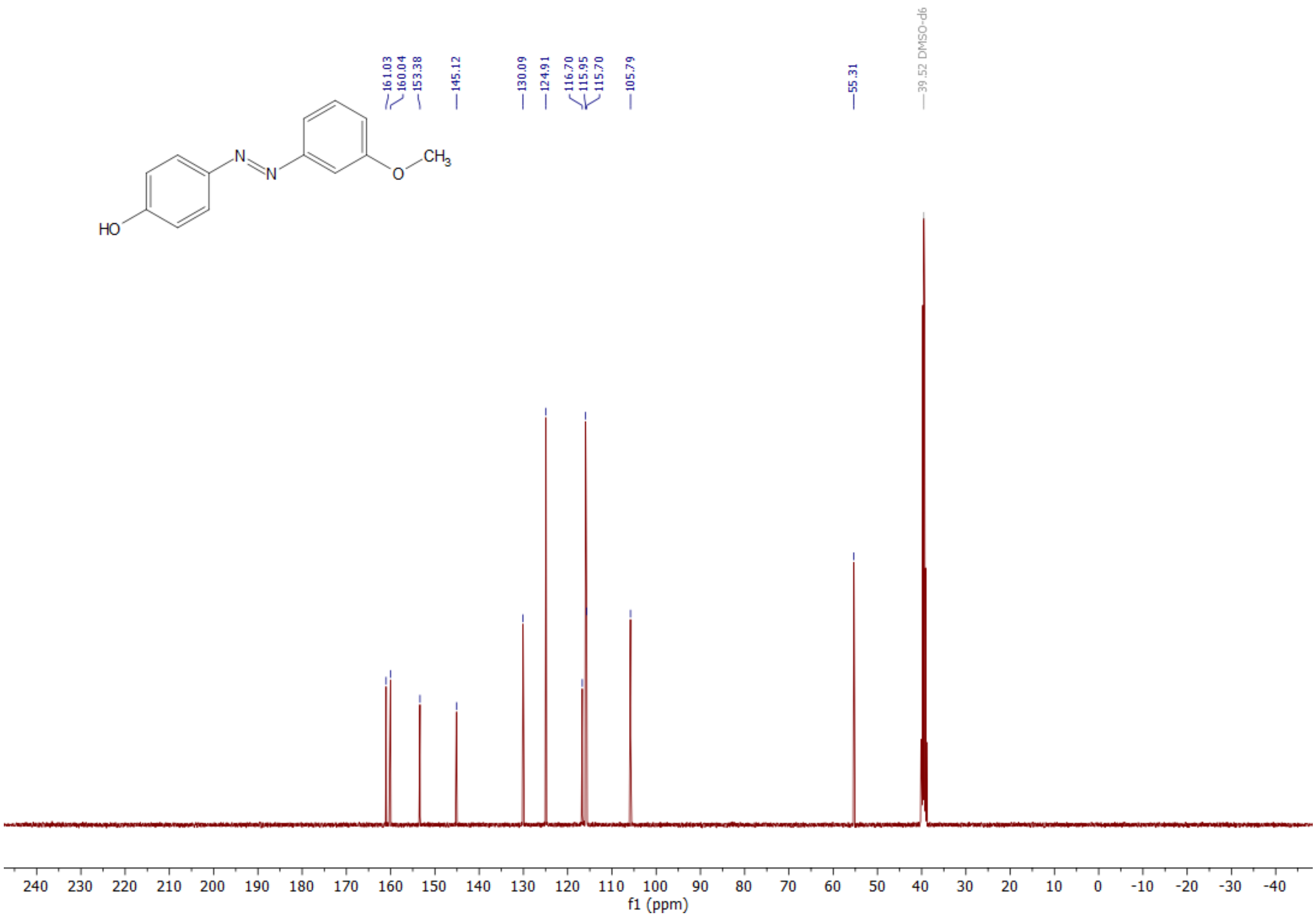

^{1}H-NMR of 1-(phenyl)-2-[4-(hexoxy)phenyl]diazene (4a)

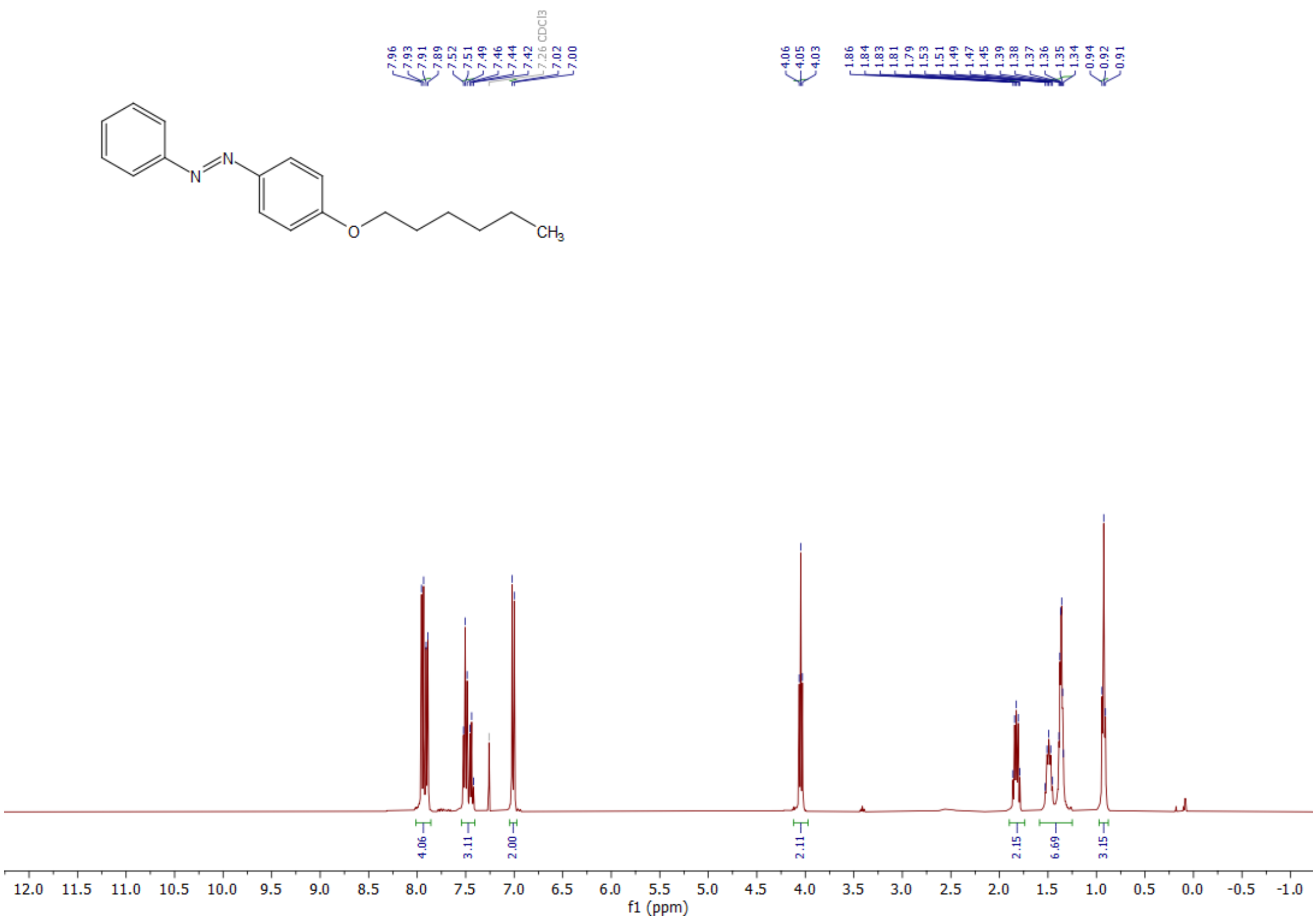

^{1}H-NMR of 1-(2-Methylphenyl)-2-[4-(hexoxy)phenyl]diazene (4b)

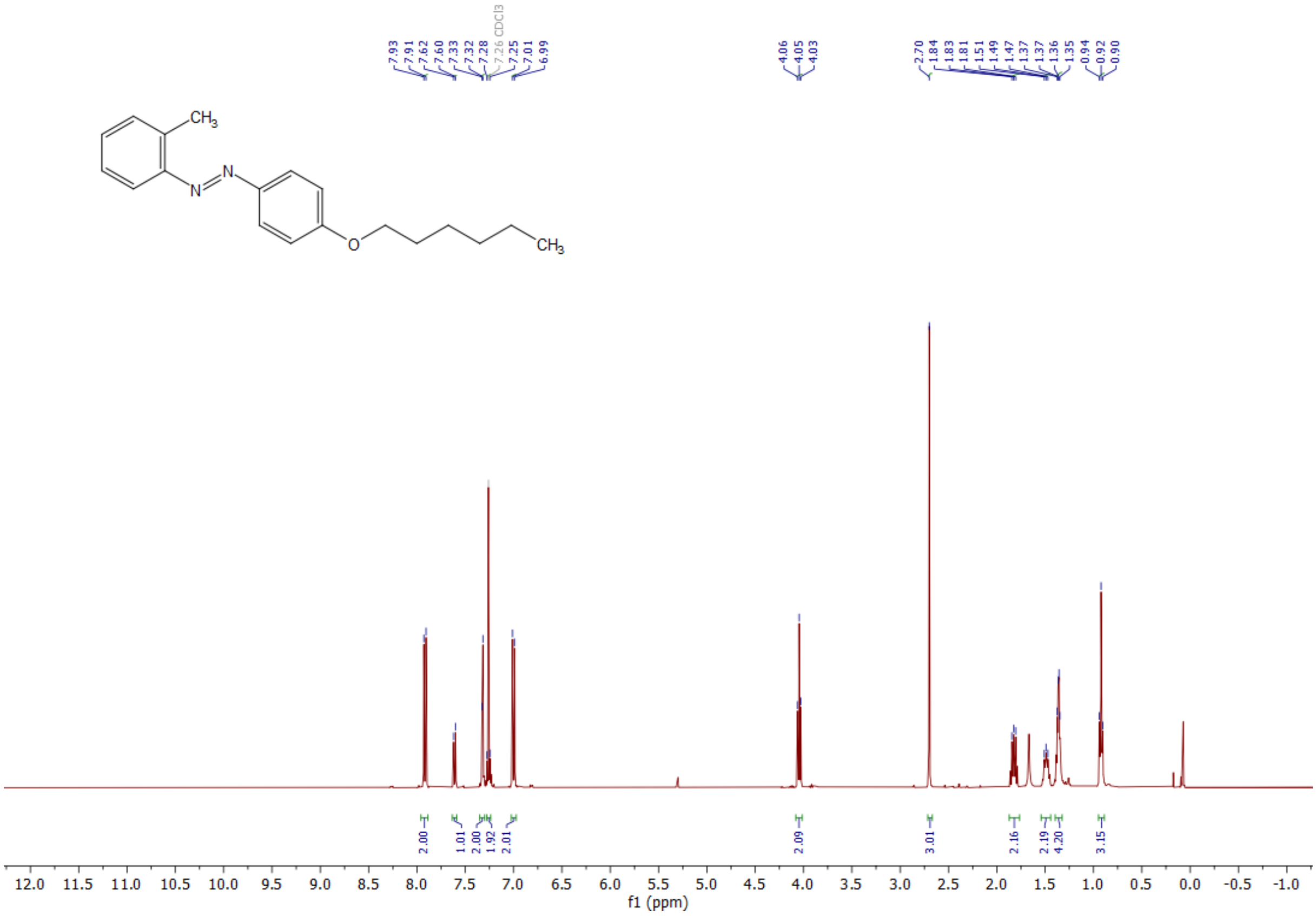

^{13}C-NMR of 1-(2-Methylphenyl)-2-[4-(hexoxy)phenyl]diazene (4b)

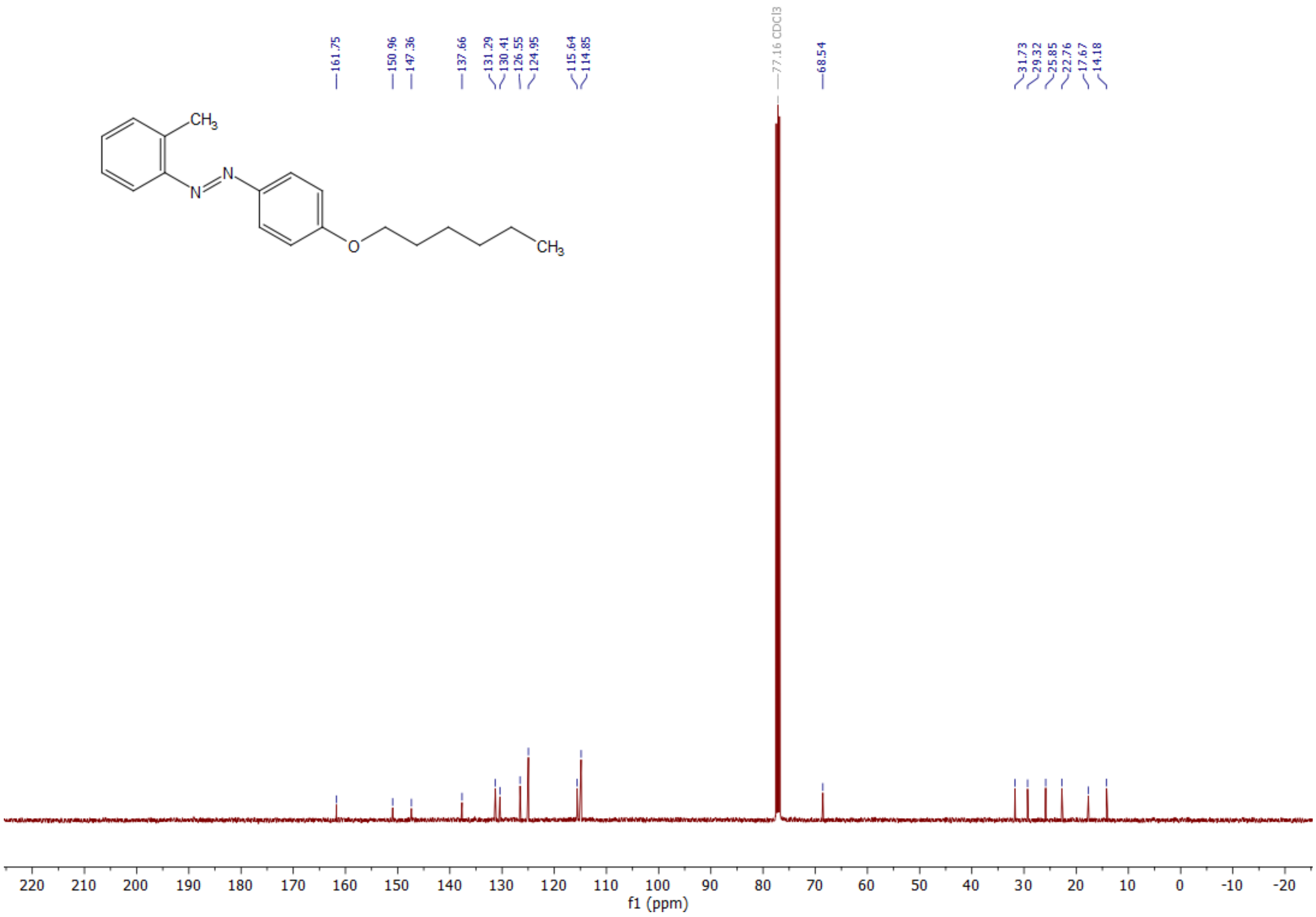

^{1}H-NMR of 1-(2-Ethylphenyl)-2-[4-(hexoxy)phenyl]diazene (4c)

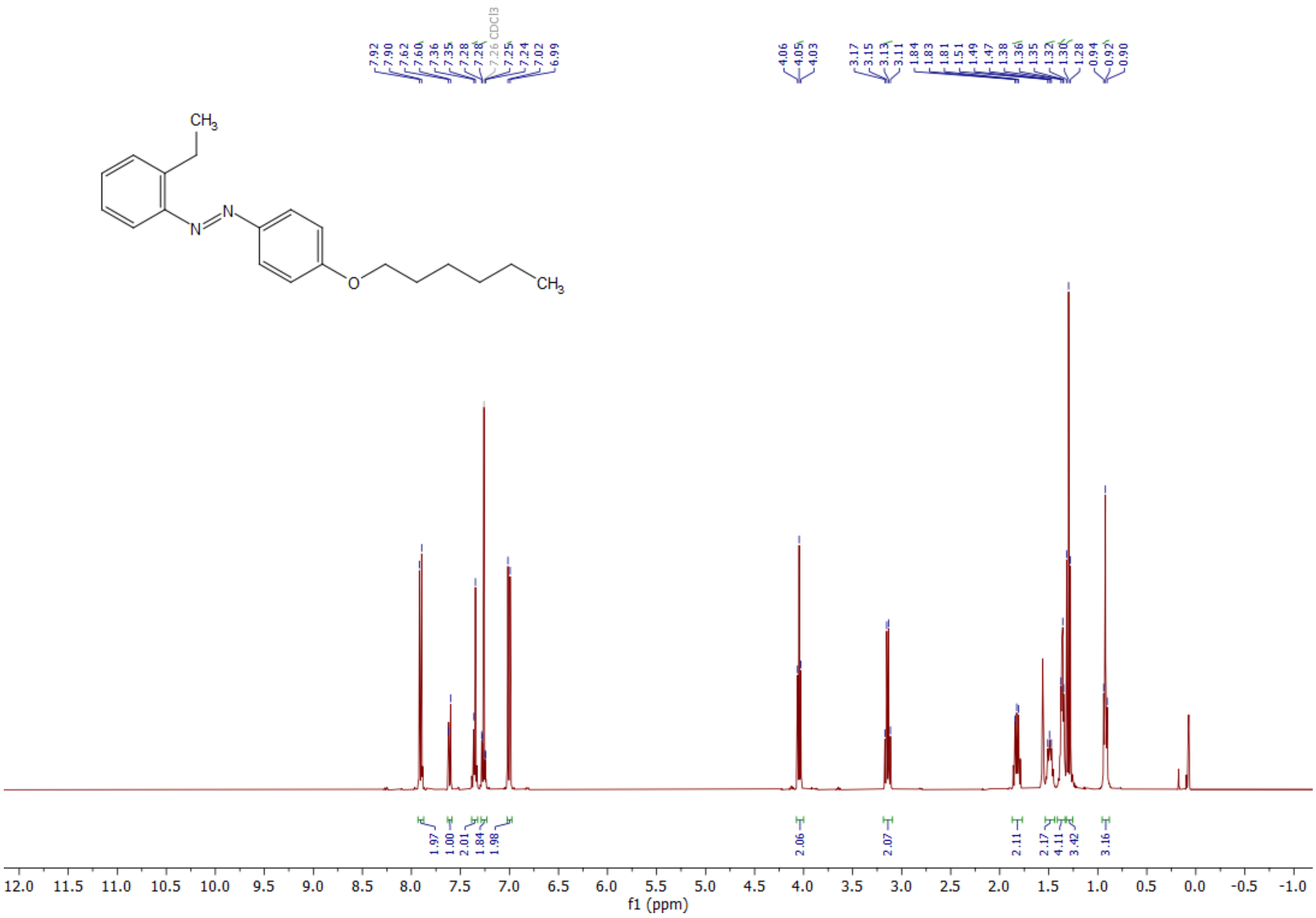

^{13}C-NMR of 1-(2-Ethylphenyl)-2-[4-(hexoxy)phenyl]diazene (4c)

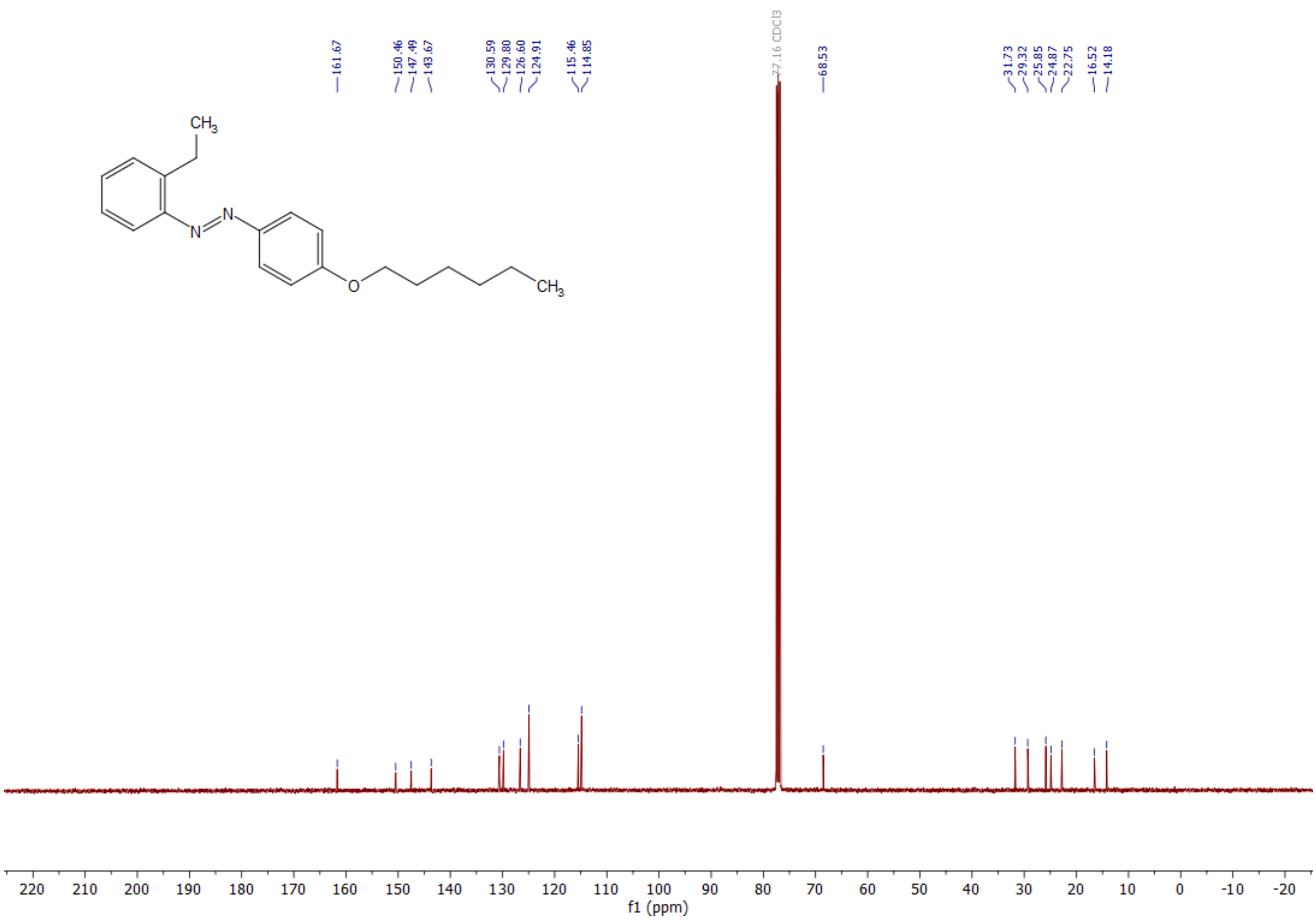

^{1}H-NMR of 1-(2-*iso*-propylphenyl)-2-[4-(hexoxy)phenyl]diazene (4d)

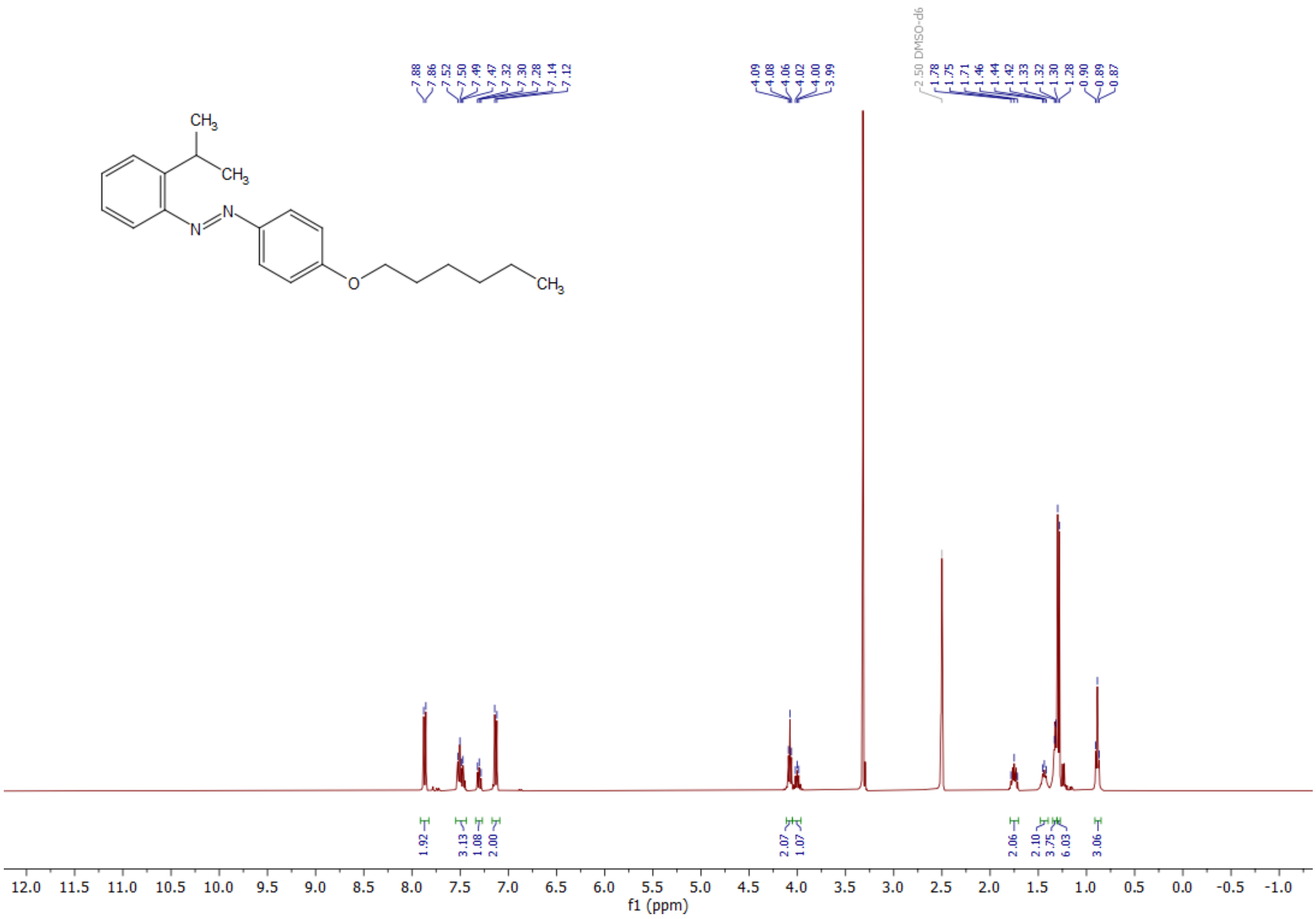

^{13}C-NMR of 1-(2-*iso*-propylphenyl)-2-[4-(hexoxy)phenyl]diazene (4d)

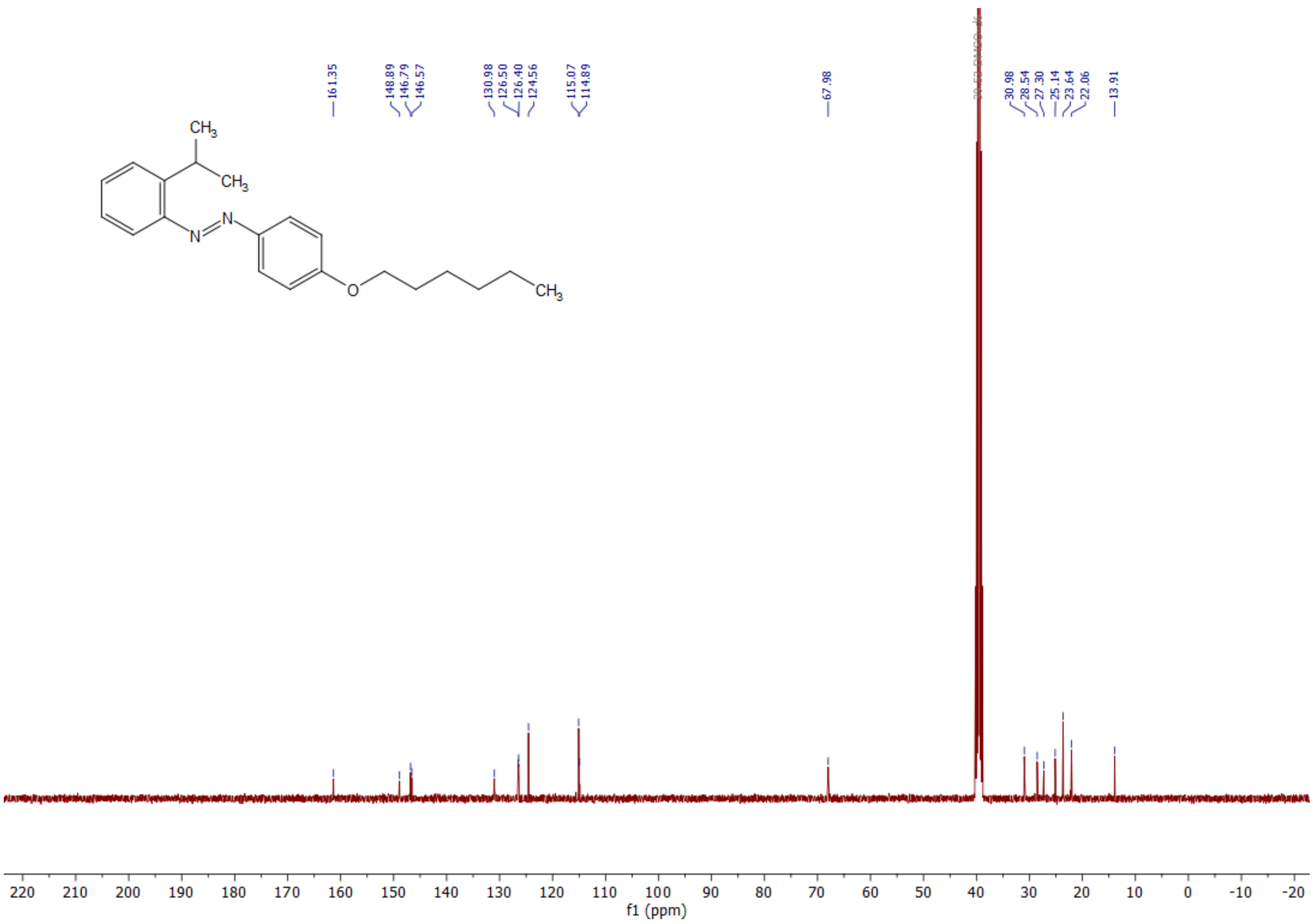

^{1}H-NMR of 1-(2-*tert*-butylphenyl)-2-[4-(hexoxy)phenyl]diazene (4e)

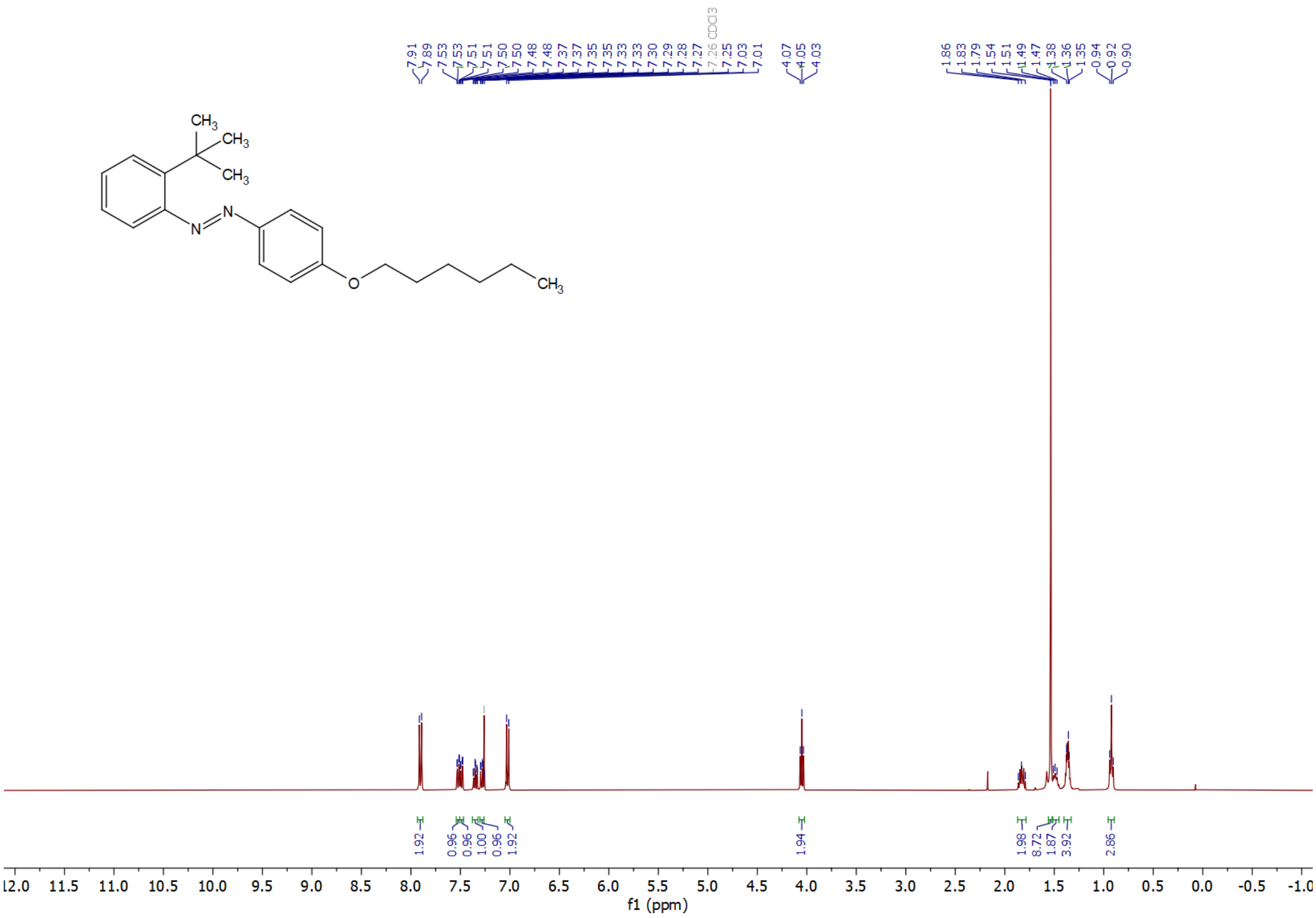

^{13}C-NMR of 1-(2-*tert*-butylphenyl)-2-[4-(hexoxy)phenyl]diazene (4e)

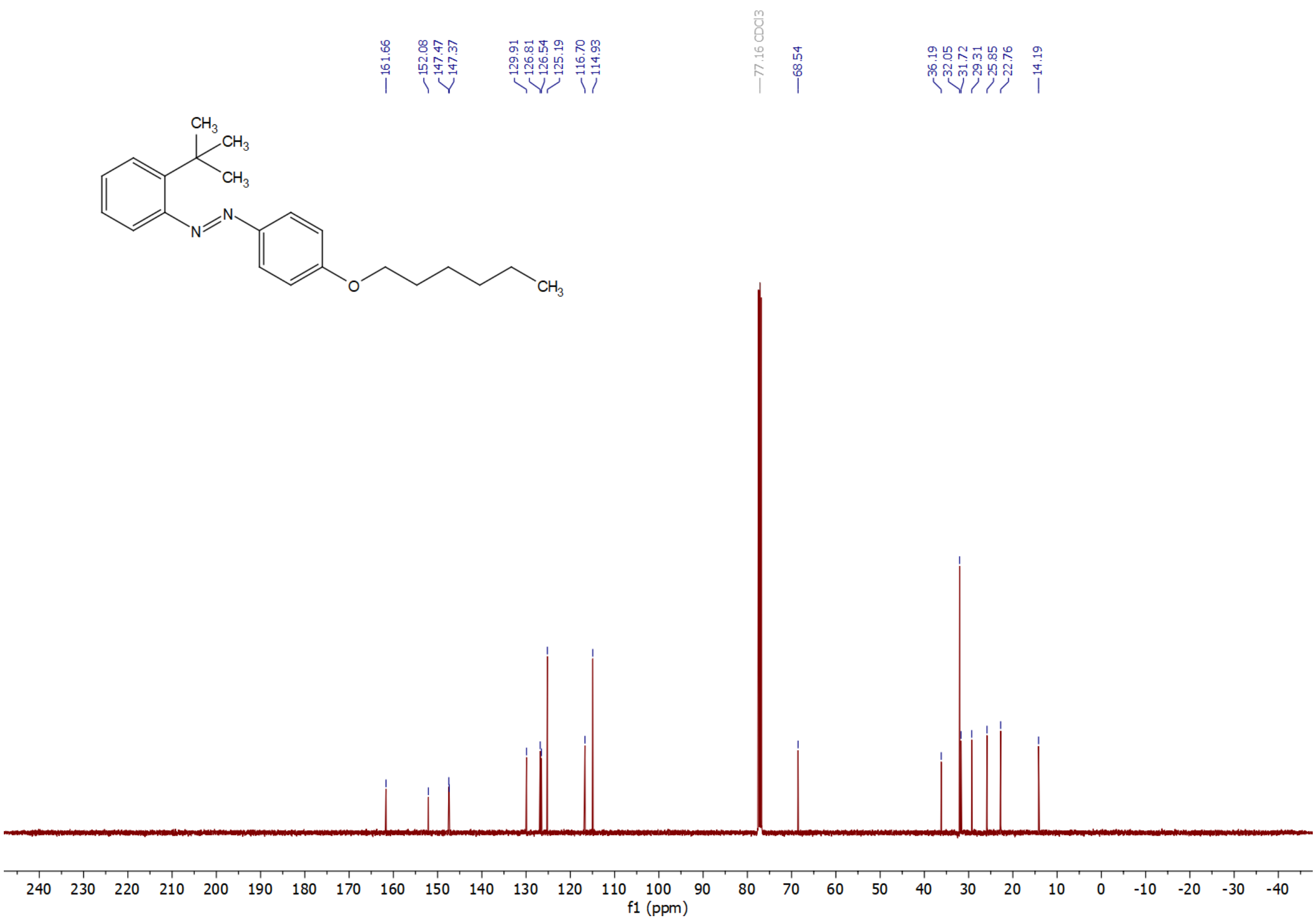

^{1}H-NMR of 1-(3-Methylphenyl)-2-[4-(hexoxy)phenyl]diazene (4f)

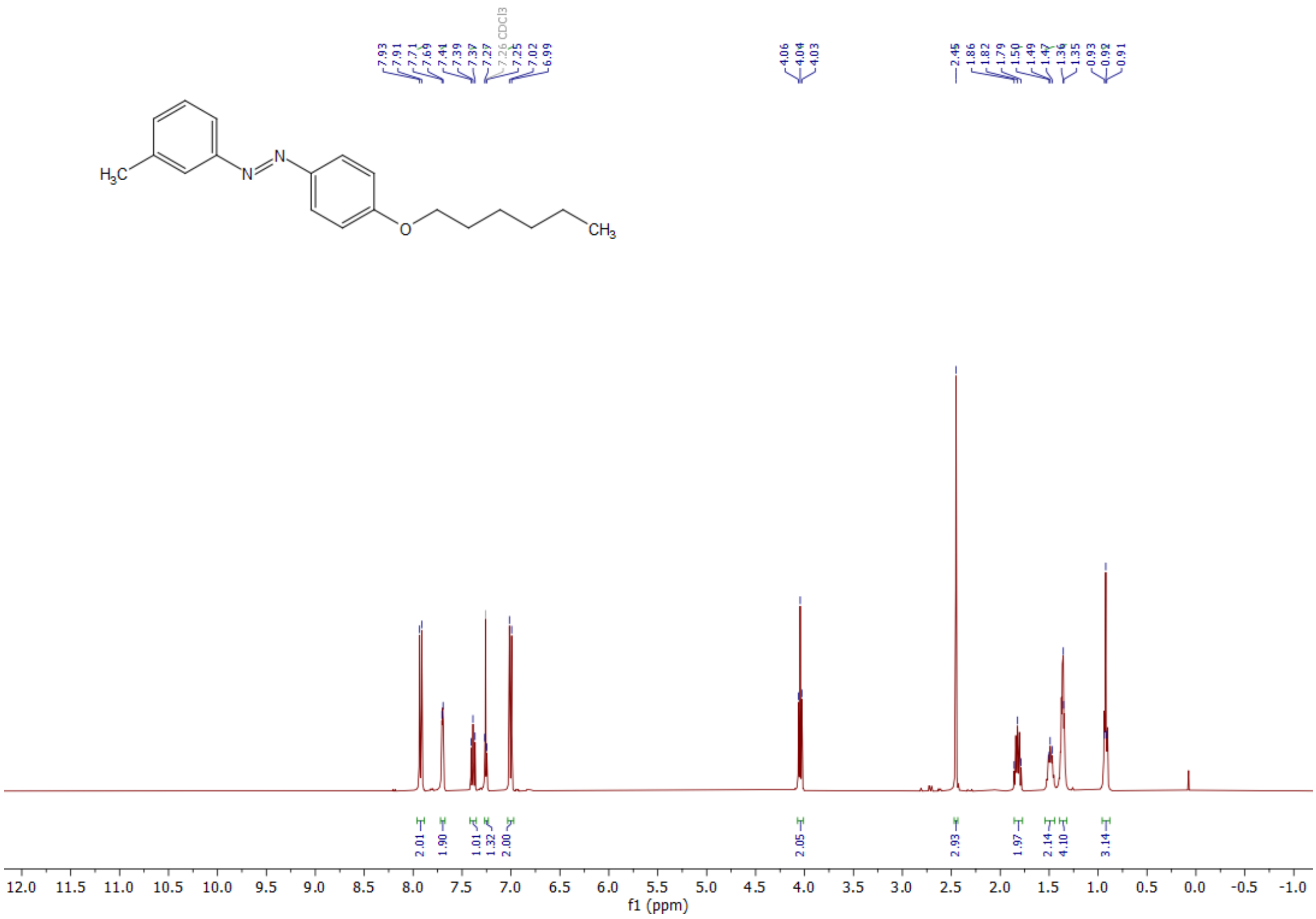

^{13}C-NMR of 1-(3-Methylphenyl)-2-[4-(hexoxy)phenyl]diazene (4f)

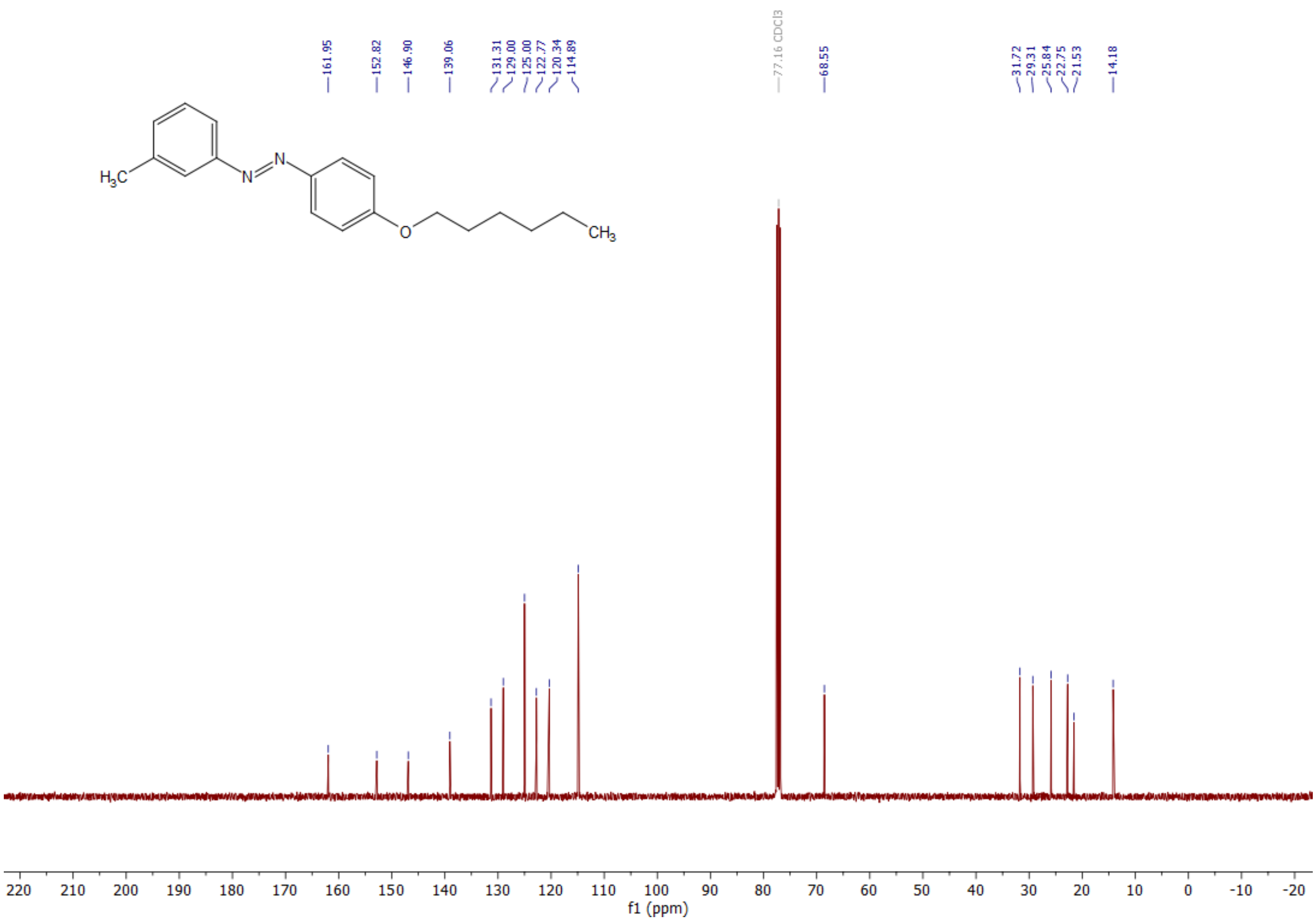

^{1}H-NMR of 1-(3-Ethylphenyl)-2-[4-(hexoxy)phenyl]diazene (4g)

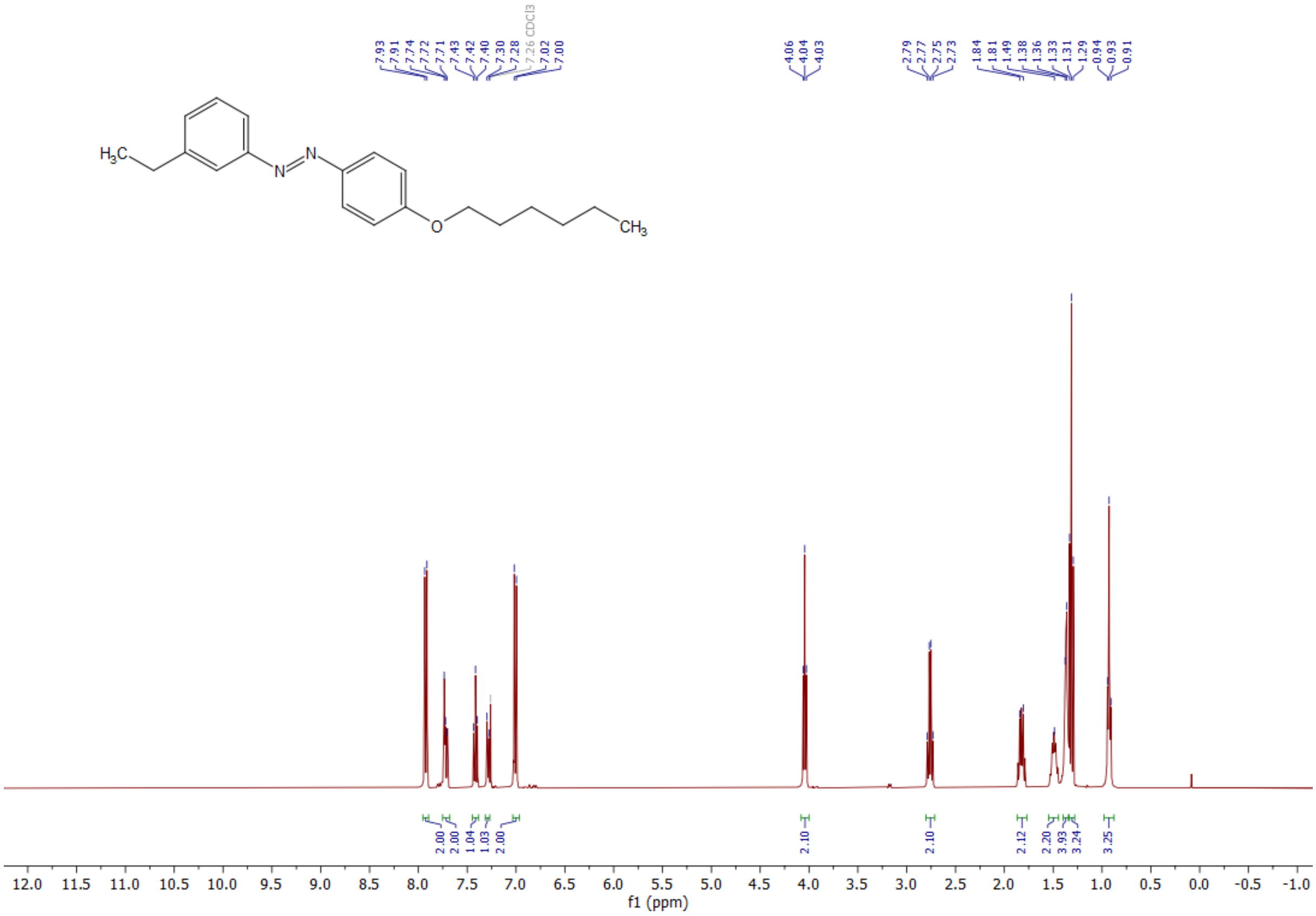

^{13}C-NMR of 1-(3-Ethylphenyl)-2-[4-(hexoxy)phenyl]diazene (4g)

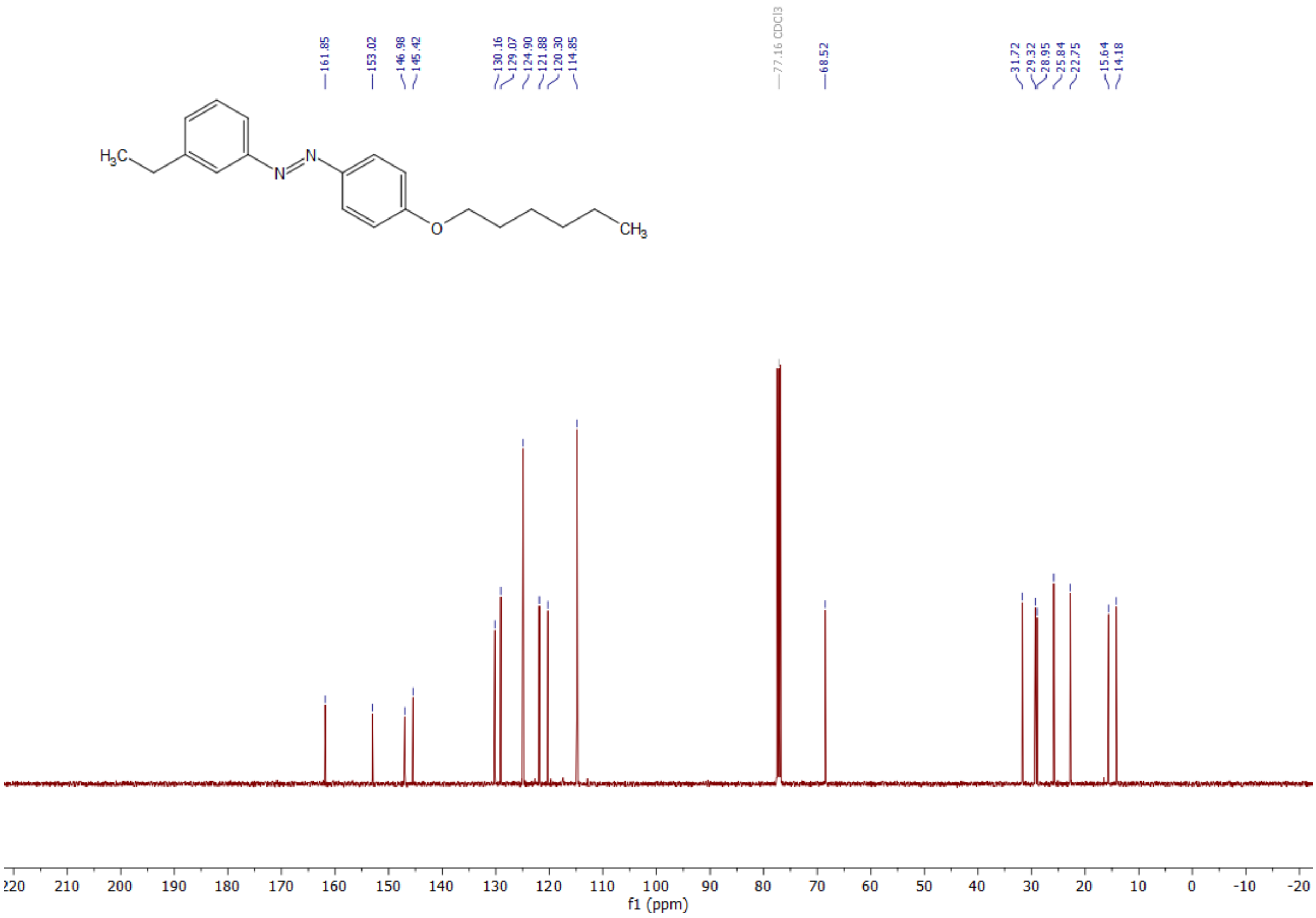

^{1}H-NMR of 1-(3-*iso*-propylphenyl)-2-[4-(hexoxy)phenyl]diazene (4h)

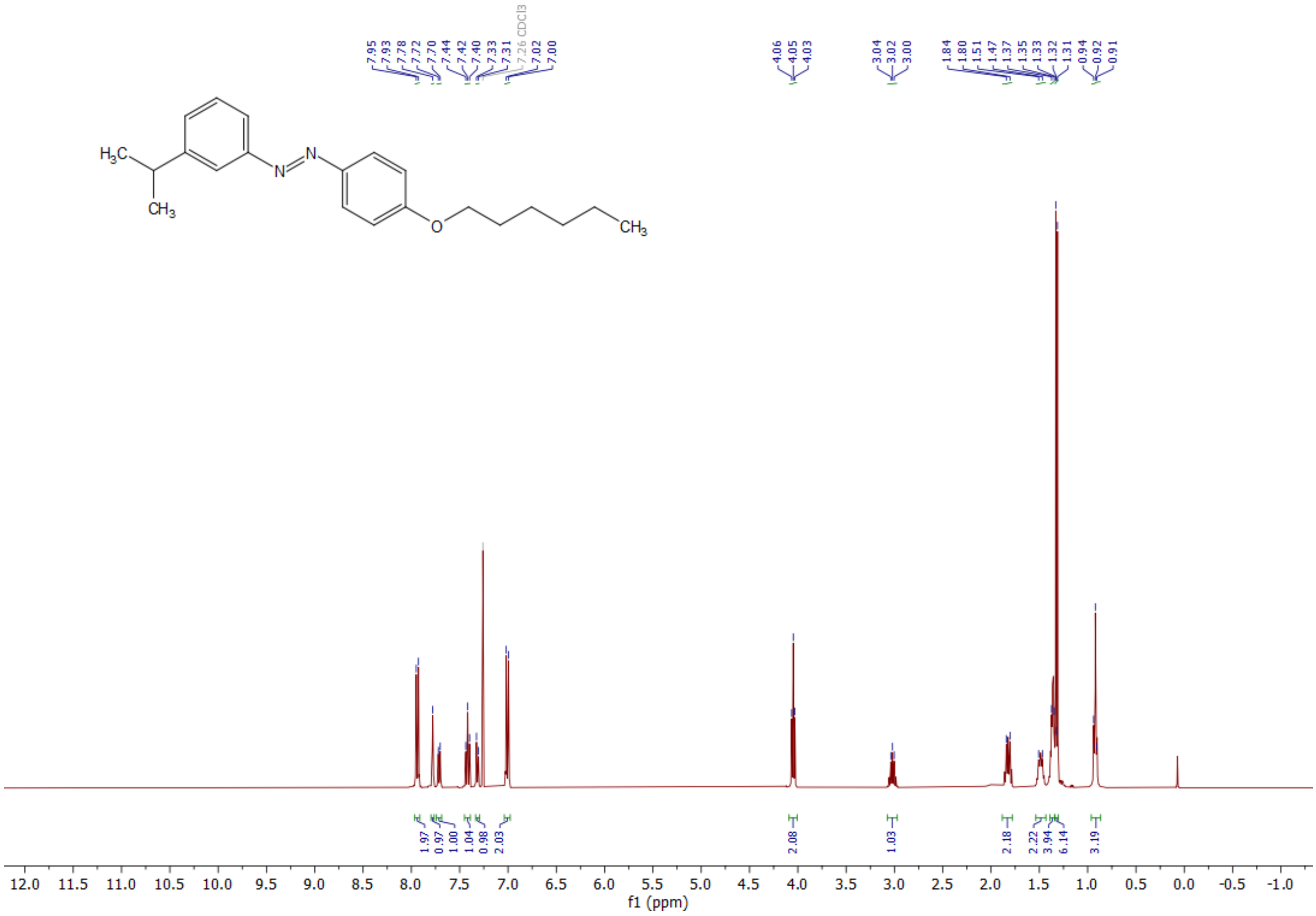

^{13}C-NMR of 1-(3-*iso*-propylphenyl)-2-[4-(hexoxy)phenyl]diazene (4h)

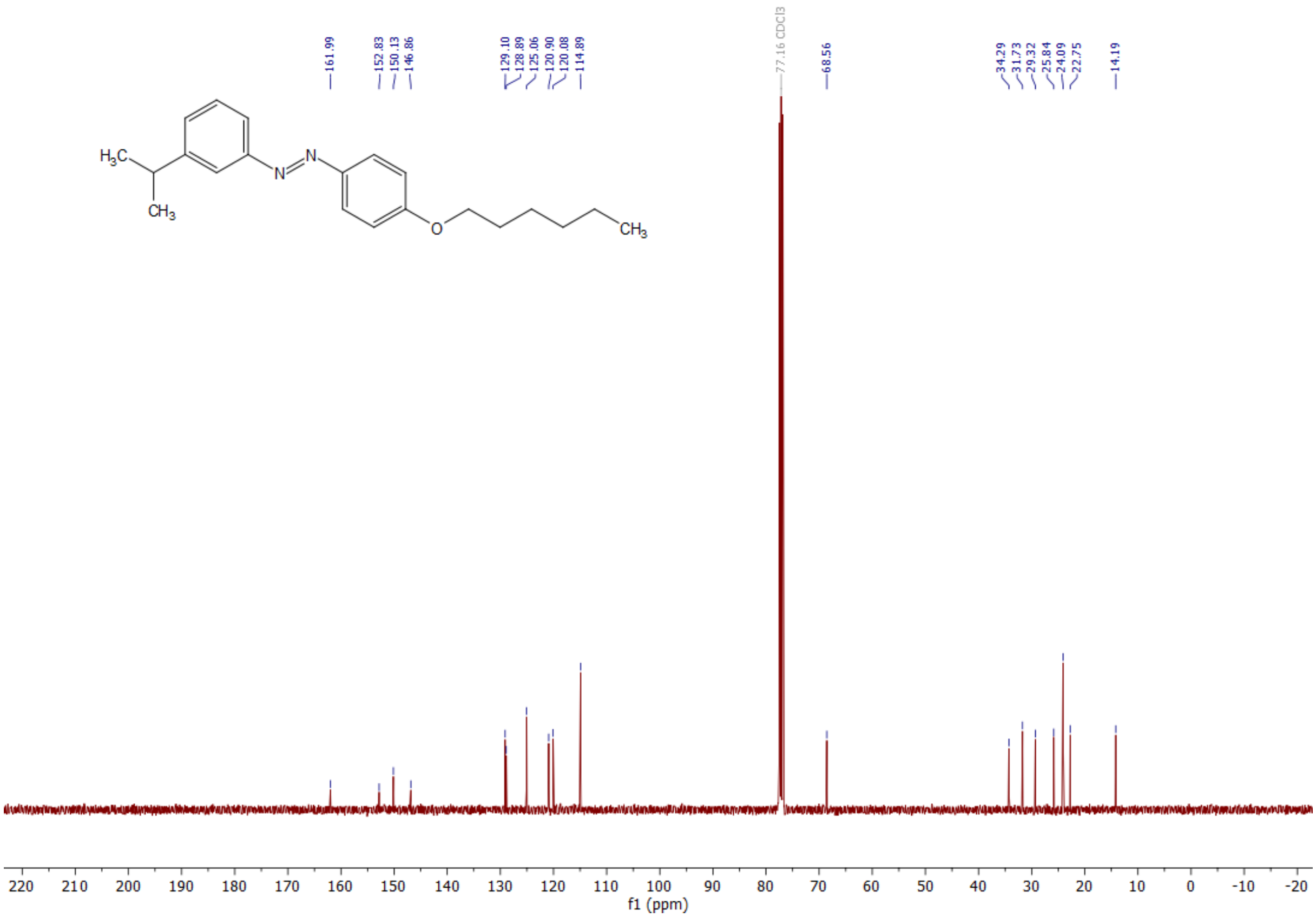

^{1}H-NMR of 1-(3-*tert*-butylphenyl)-2-[4-(hexoxy)phenyl]diazene (4i)

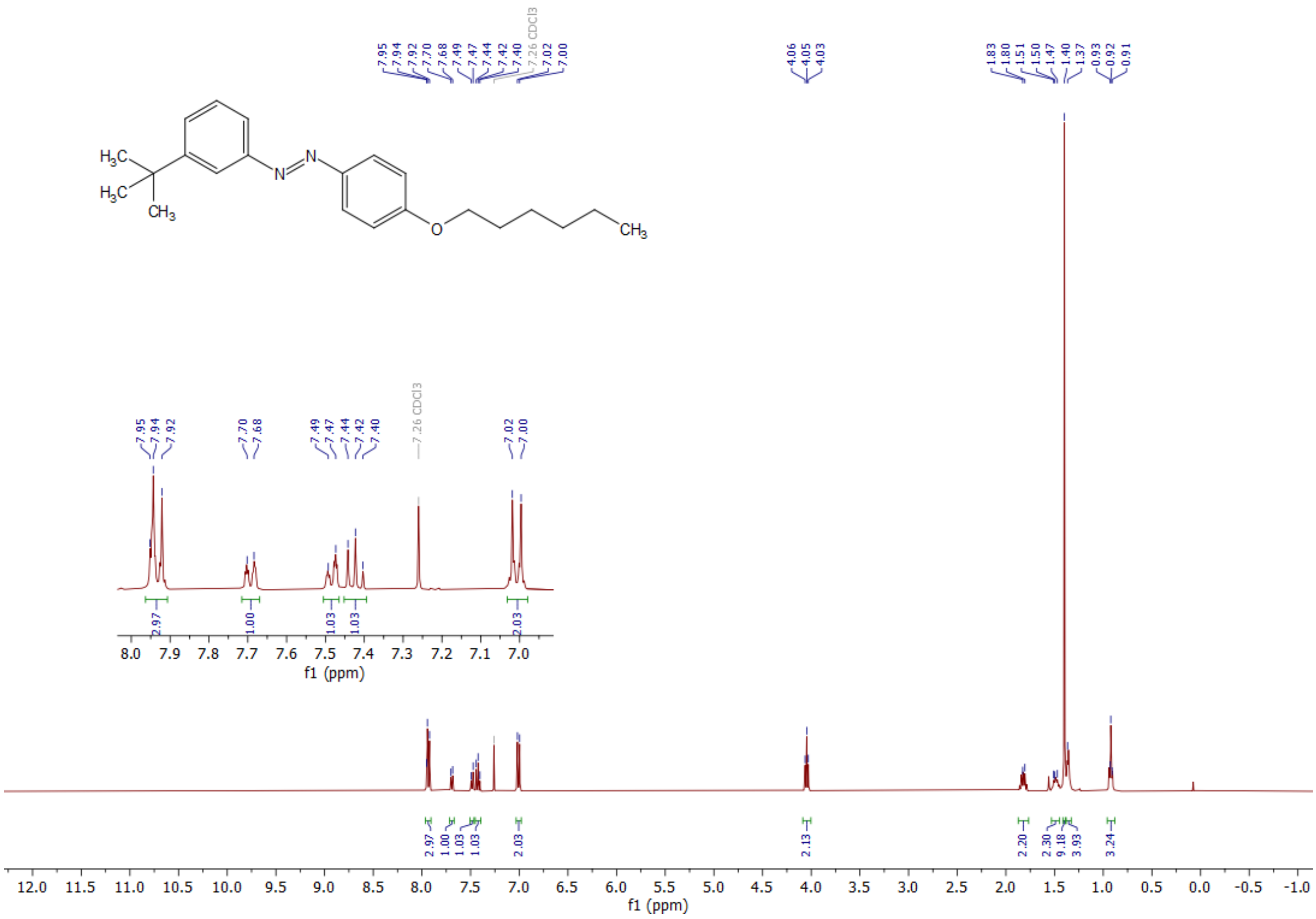

^{13}C-NMR of 1-(3-*tert*-butylphenyl)-2-[4-(hexoxy)phenyl]diazene (4i)

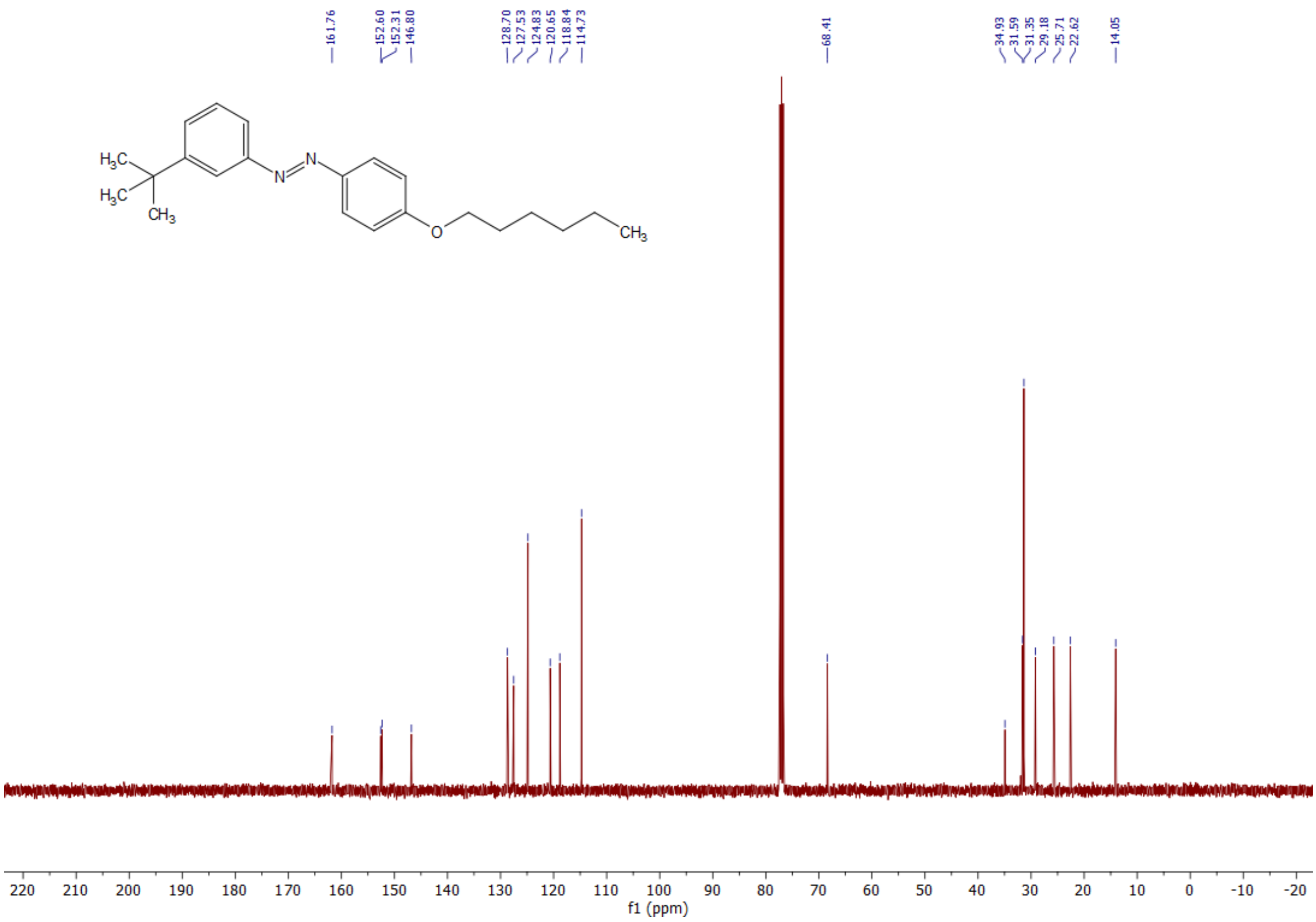

^{1}H-NMR of 1-(4-Methylphenyl)-2-[4-(hexoxy)phenyl]diazene (4j)

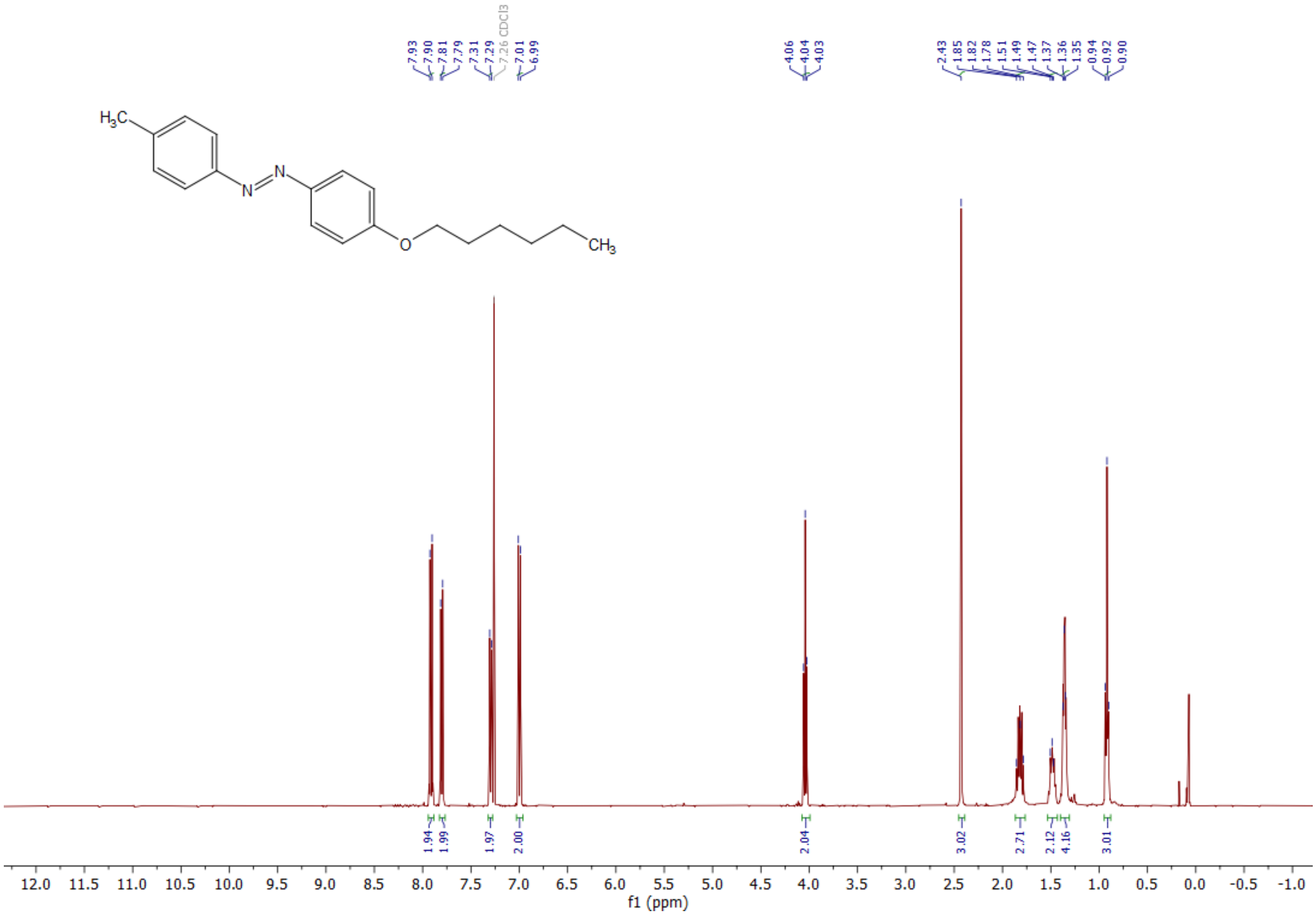

^{13}C-NMR of 1-(4-Methylphenyl)-2-[4-(hexoxy)phenyl]diazene (4j)

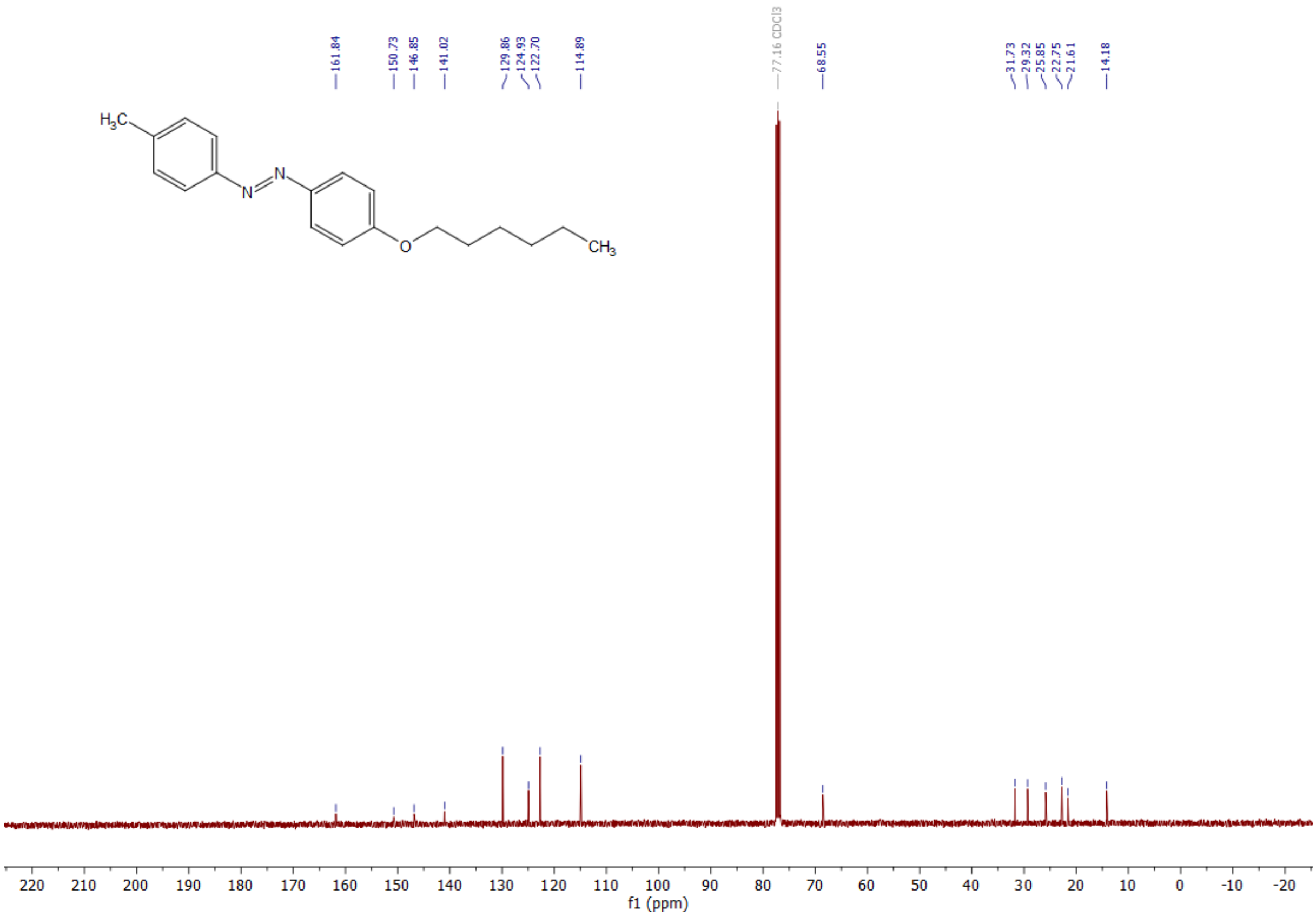

^{1}H-NMR of 1-(4-Ethylphenyl)-2-[4-(hexoxy)phenyl]diazene (4k)

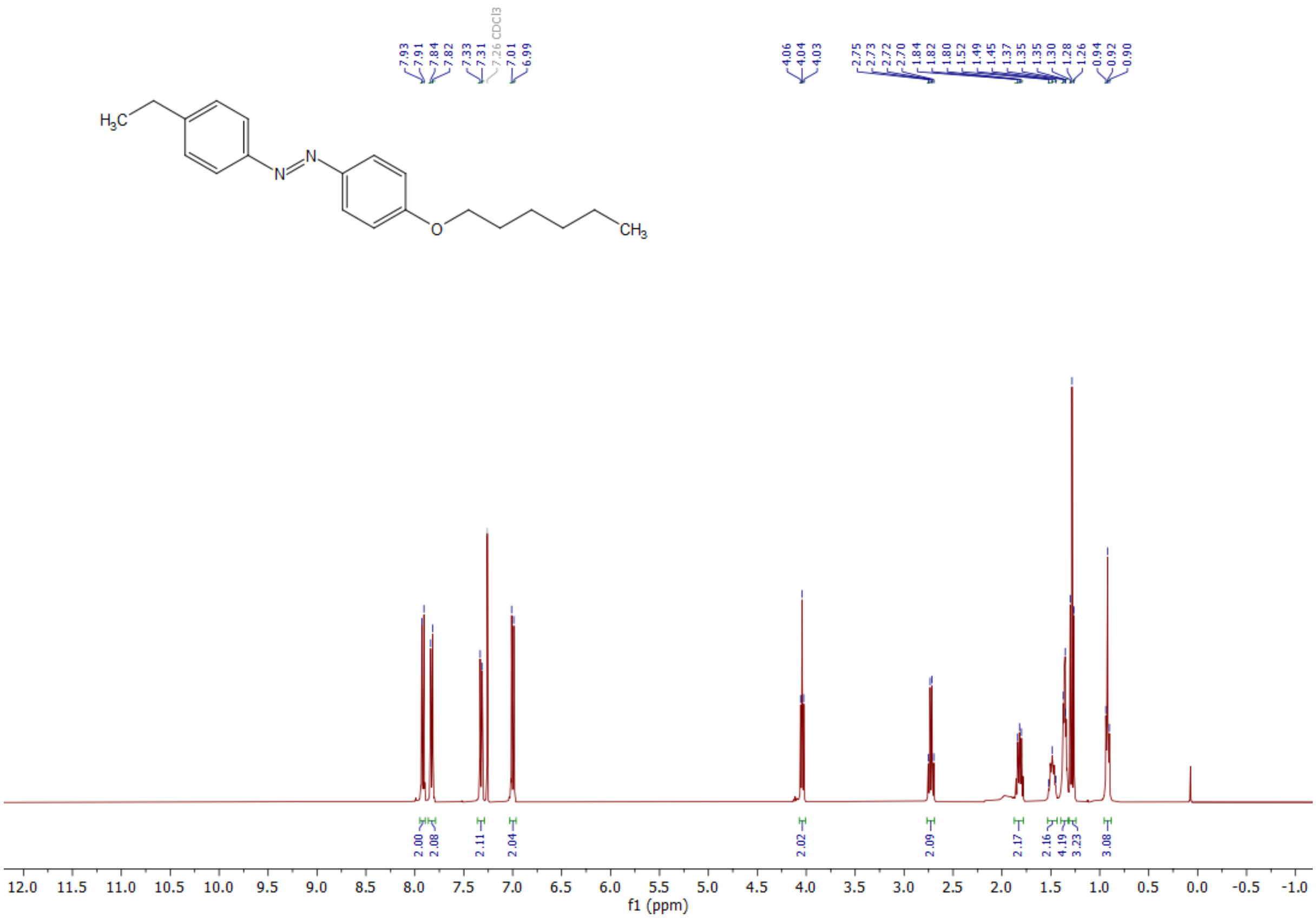

^{13}C-NMR of 1-(4-Ethylphenyl)-2-[4-(hexoxy)phenyl]diazene (4k)

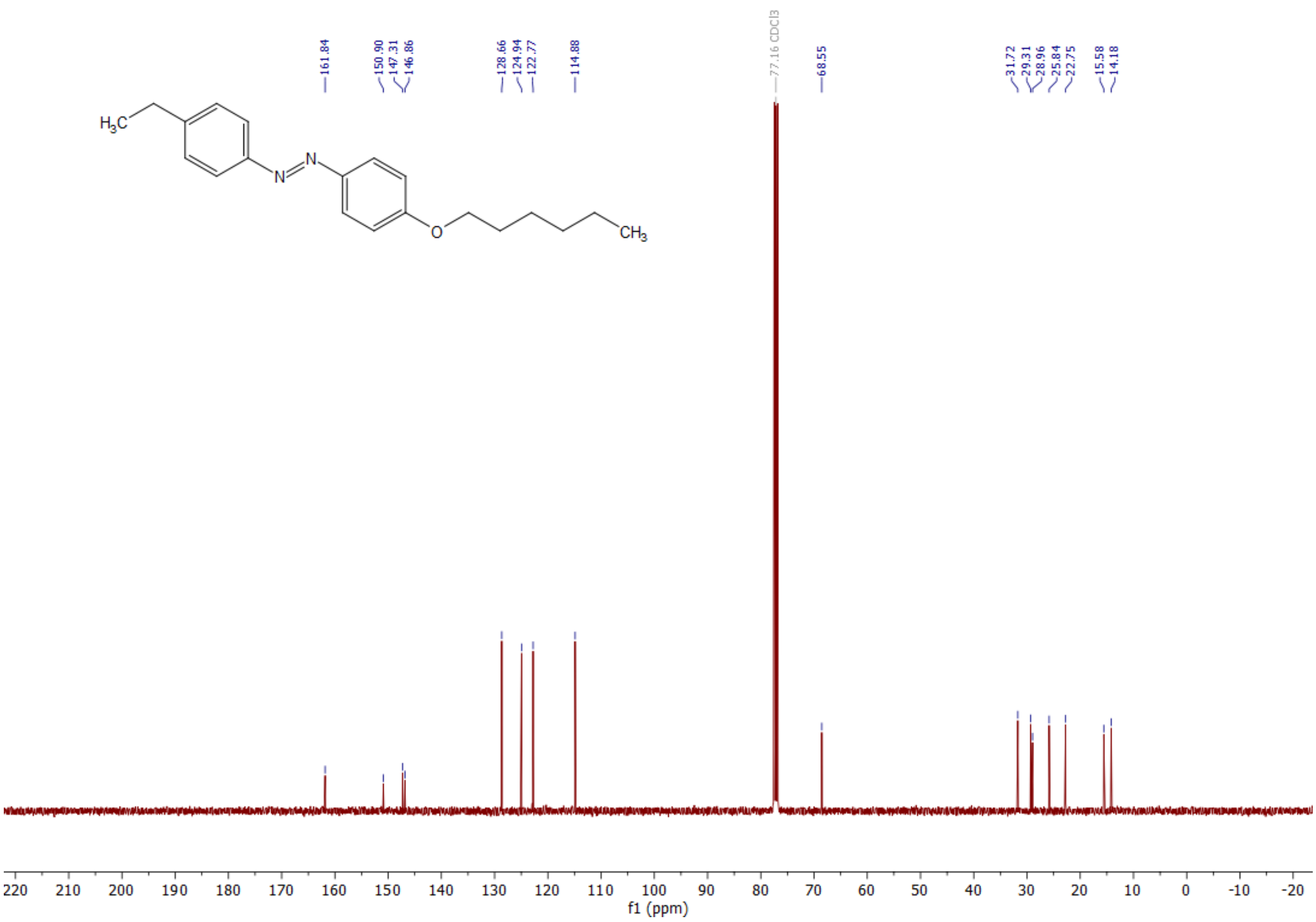

^{1}H-NMR of 1-(4-*iso*-propylphenyl)-2-[4-(hexoxy)phenyl]diazene (4l)

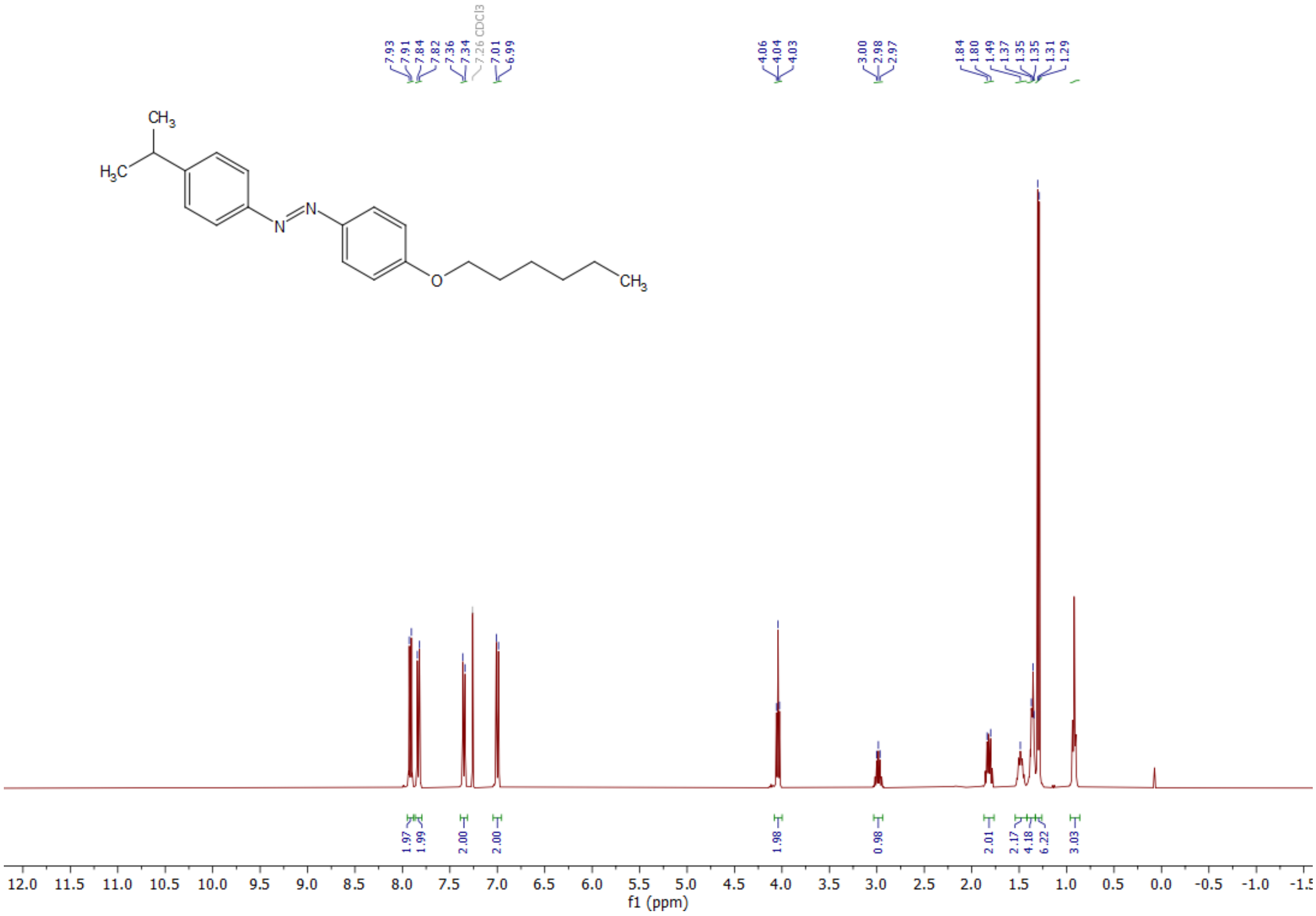

^{13}C-NMR of 1-(4-*iso*-propylphenyl)-2-[4-(hexoxy)phenyl]diazene (4l)

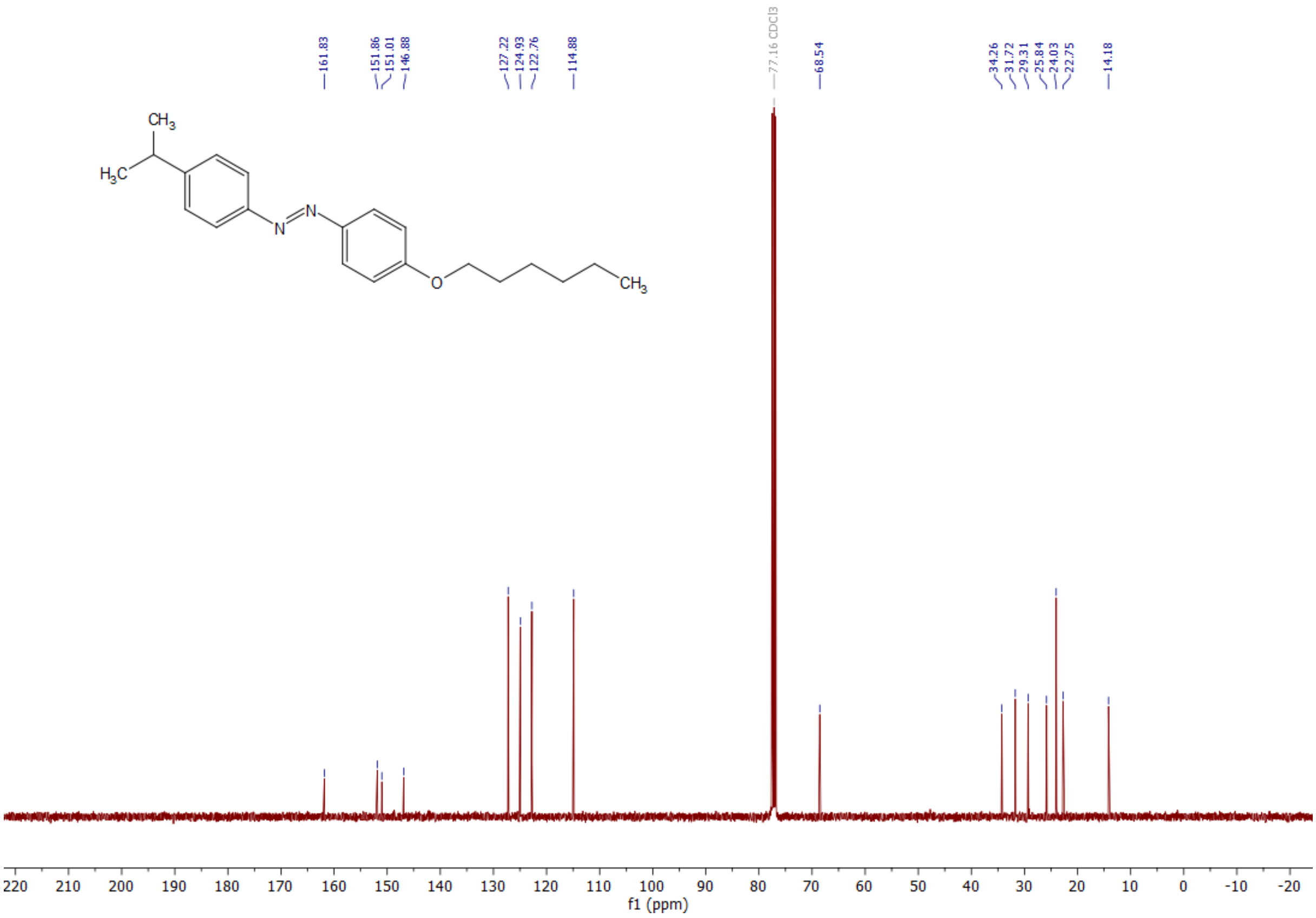

^{1}H-NMR of 1-(4-*tert*-butylphenyl)-2-[4-(hexoxy)phenyl]diazene (4m)

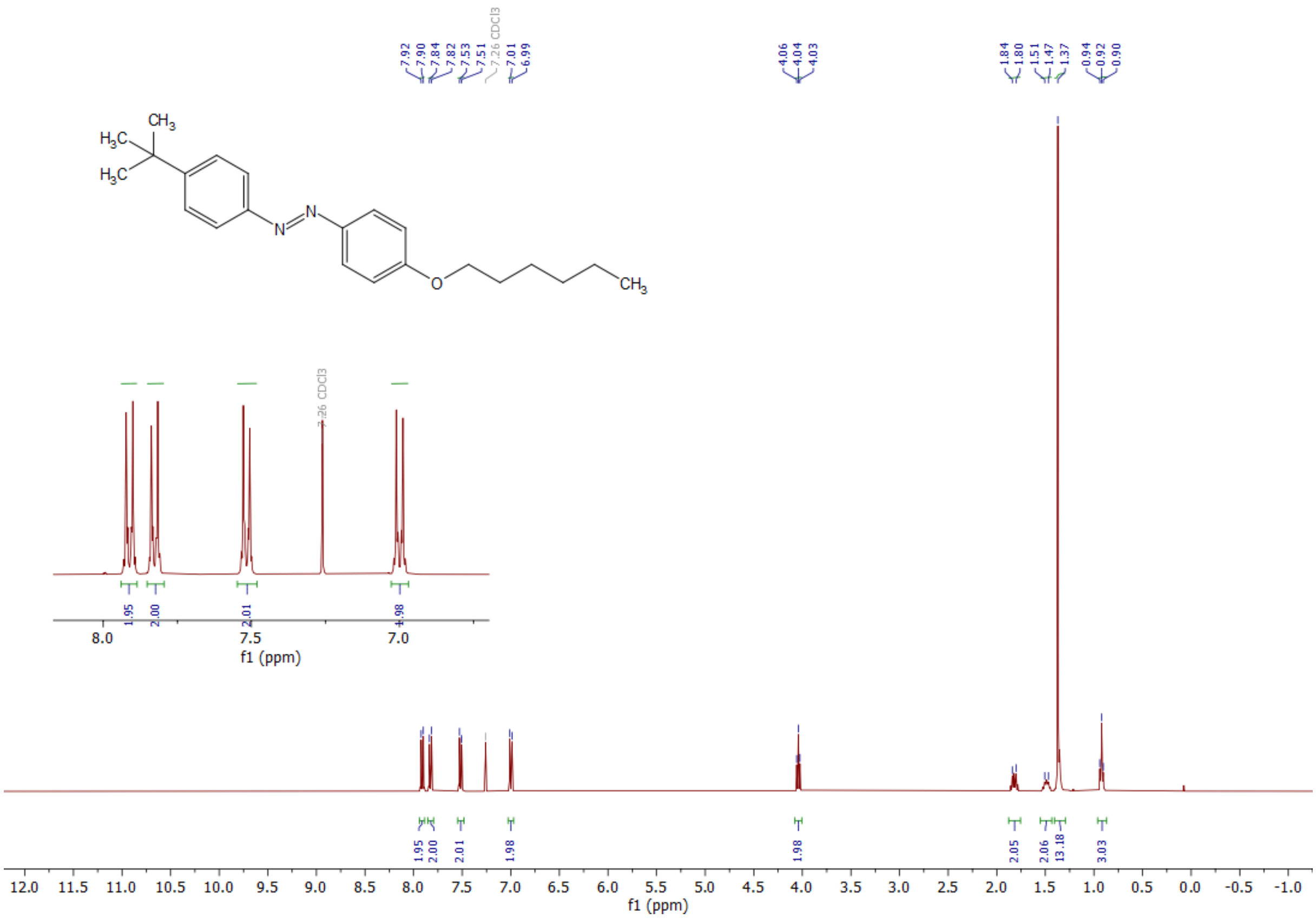

^{13}C-NMR of 1-(4-*tert*-butylphenyl)-2-[4-(hexoxy)phenyl]diazene (4m)

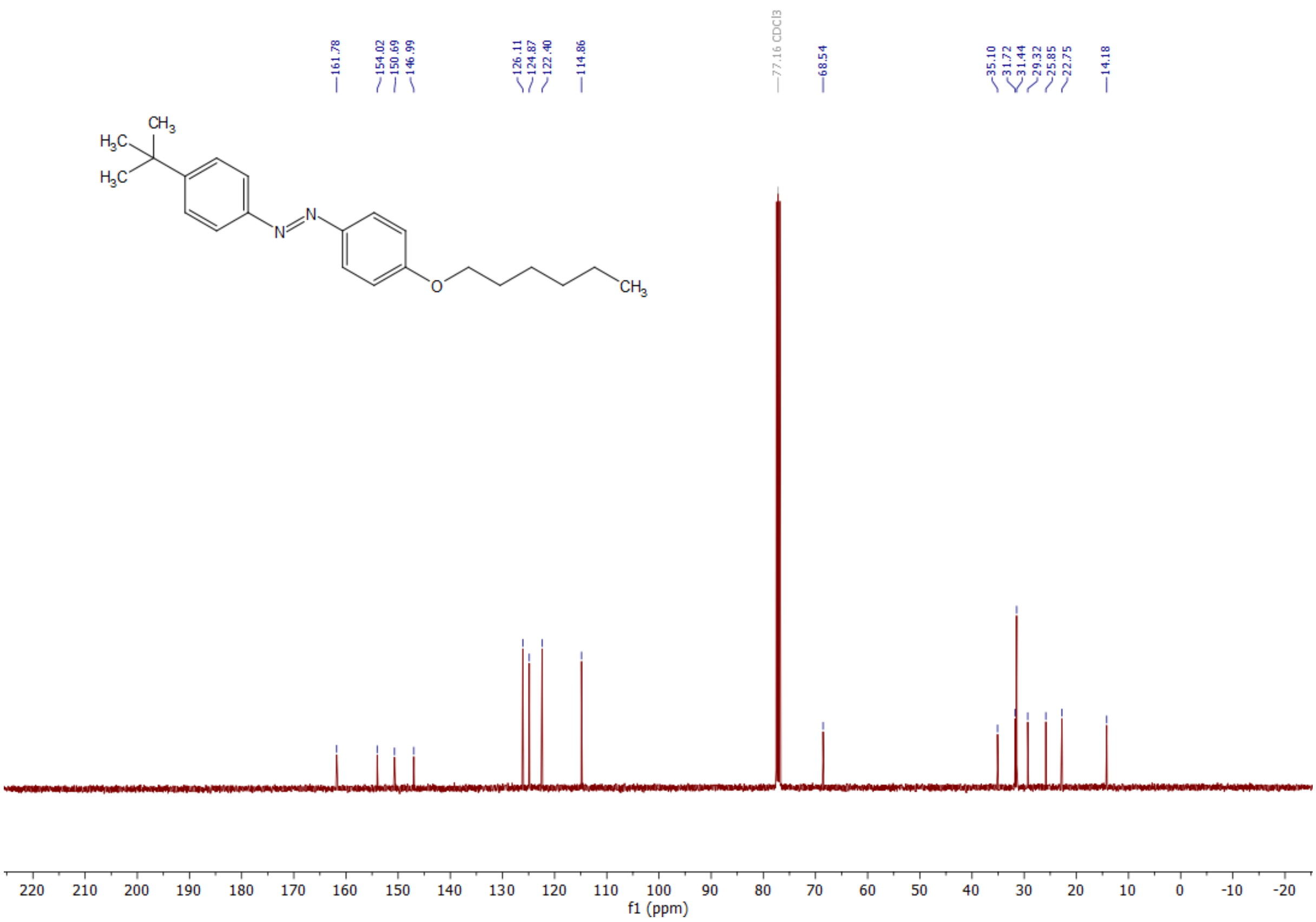

^{1}H-NMR of 1-(2,6-Dimethylphenyl)-2-[4-(hexoxy)phenyl]diazene (4n)

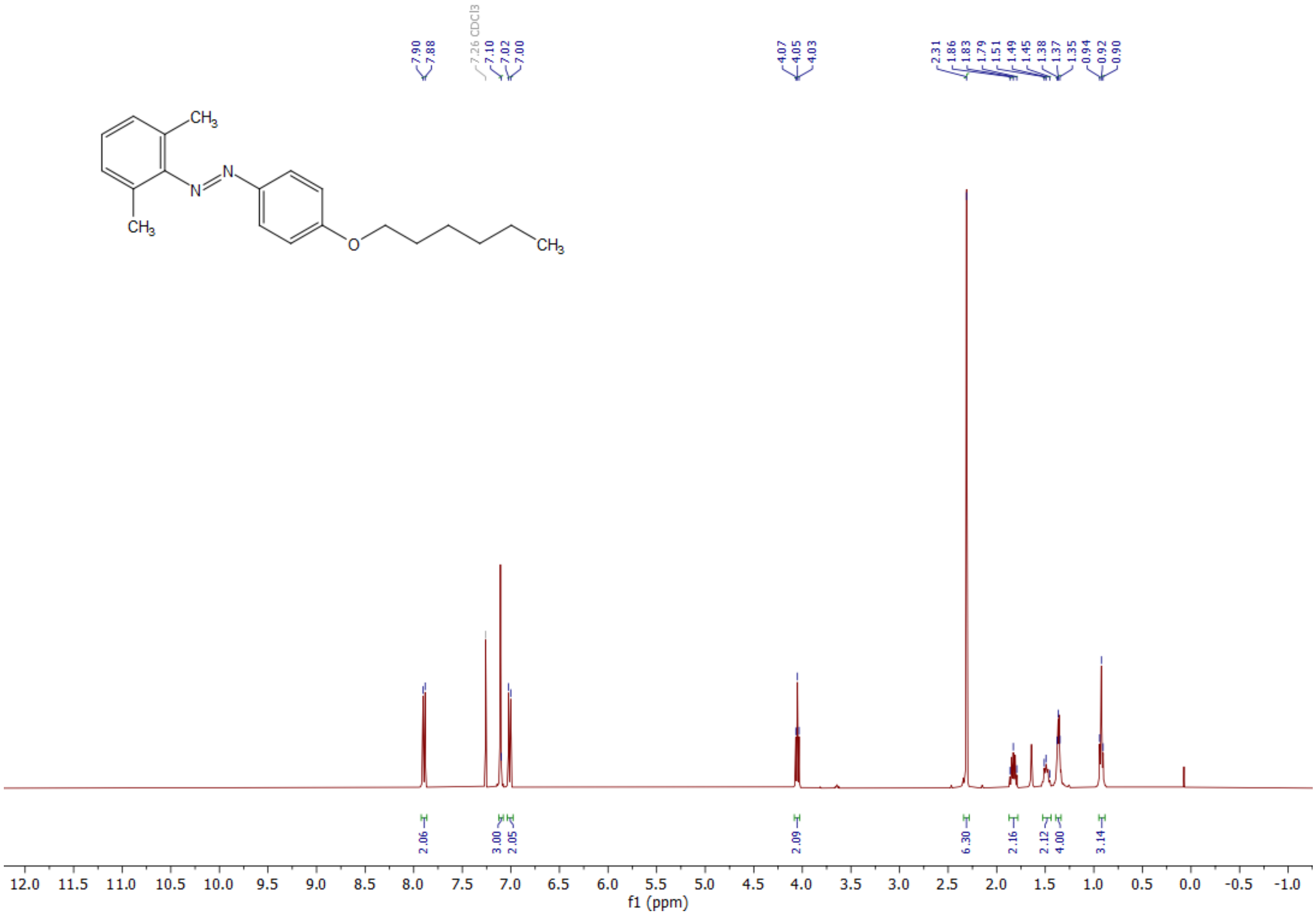

^{1}H-NMR of 1-(2,6-Dimethylphenyl)-2-[4-(hexoxy)phenyl]diazene (4n)

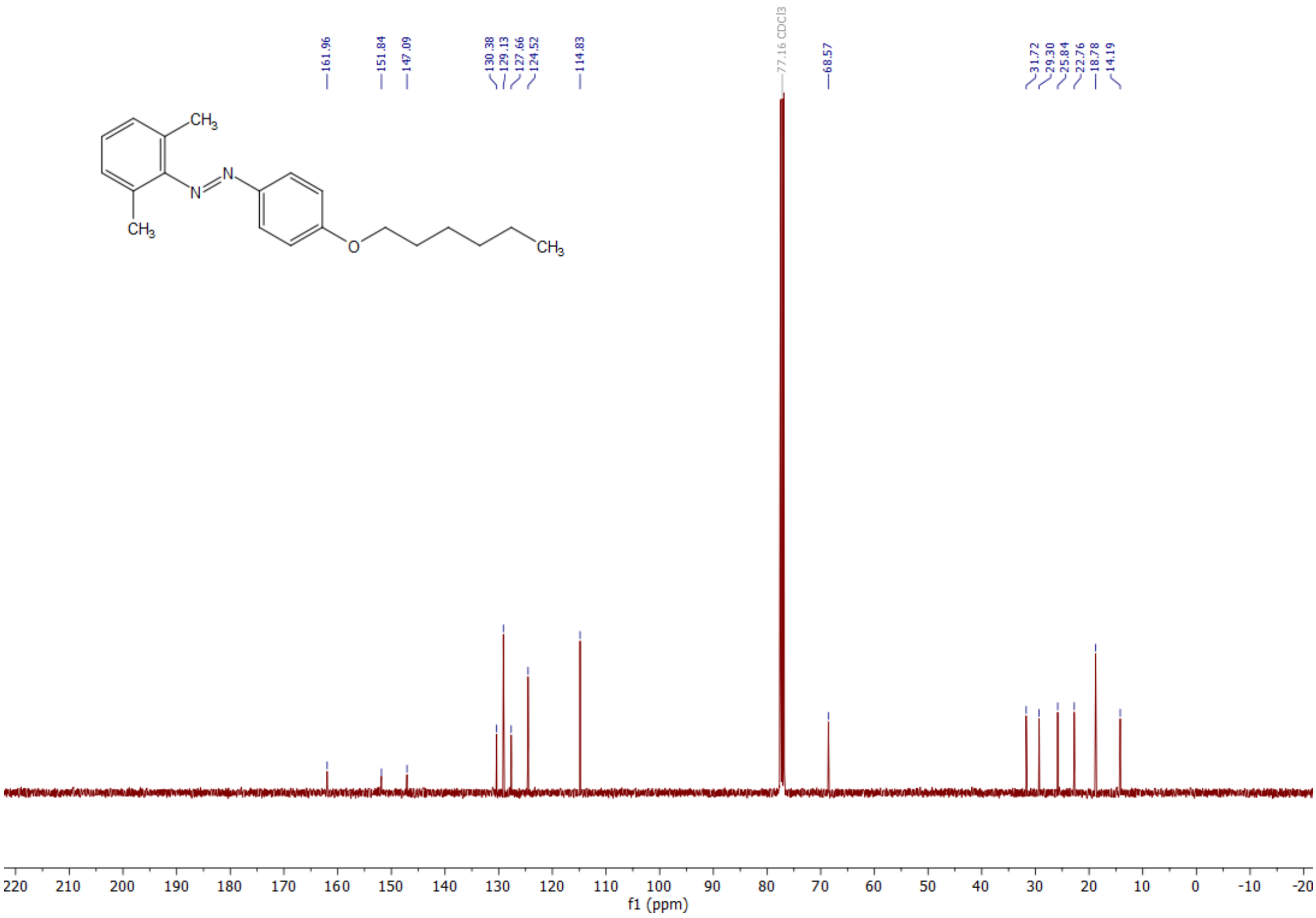

^{1}H-NMR of 1-(2,4,6-Trimethylphenyl)-2-[4-(hexoxy)phenyl]diazene (4o)

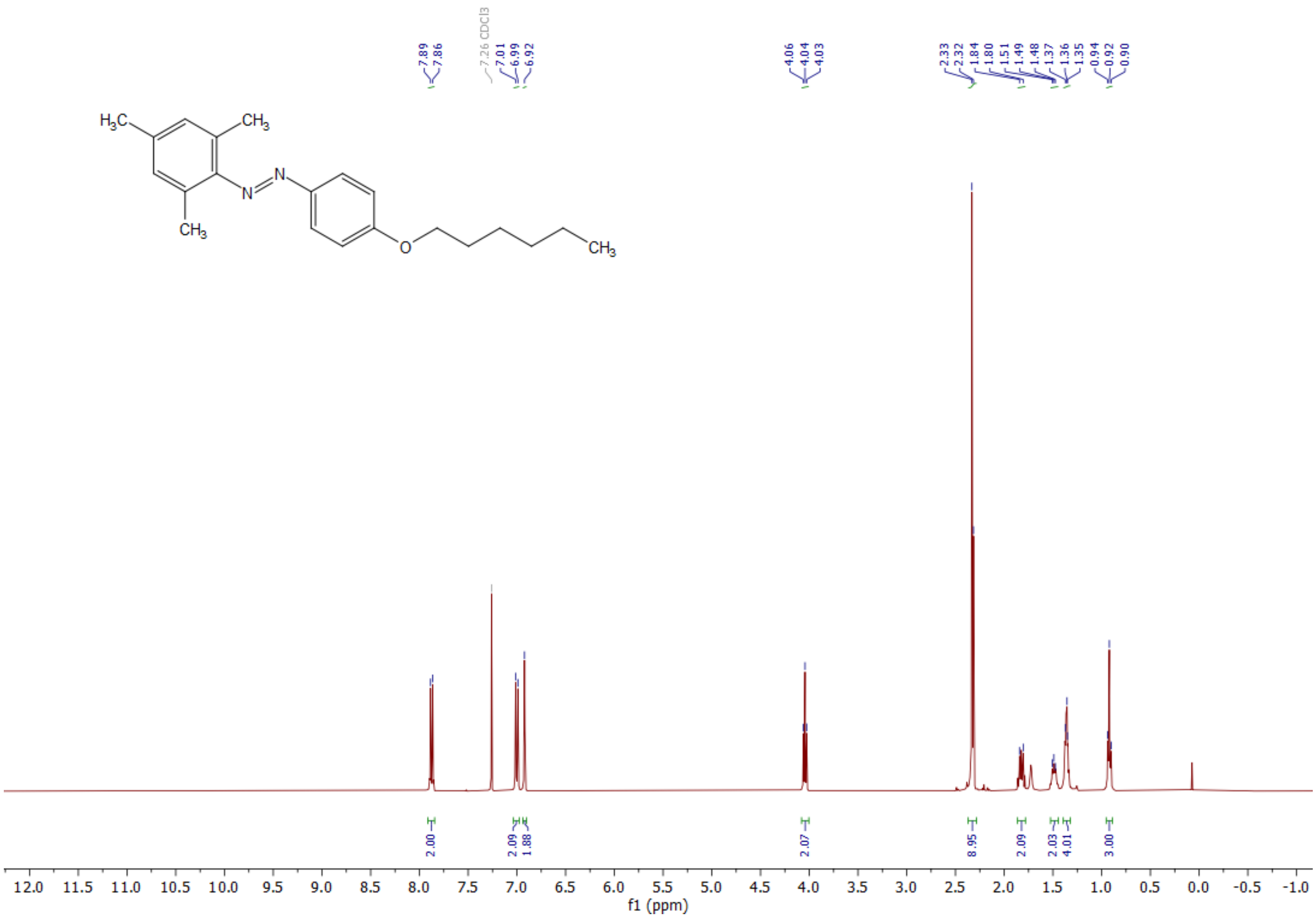

^{13}C-NMR of 1-(2,4,6-Trimethylphenyl)-2-[4-(hexoxy)phenyl]diazene (4o)

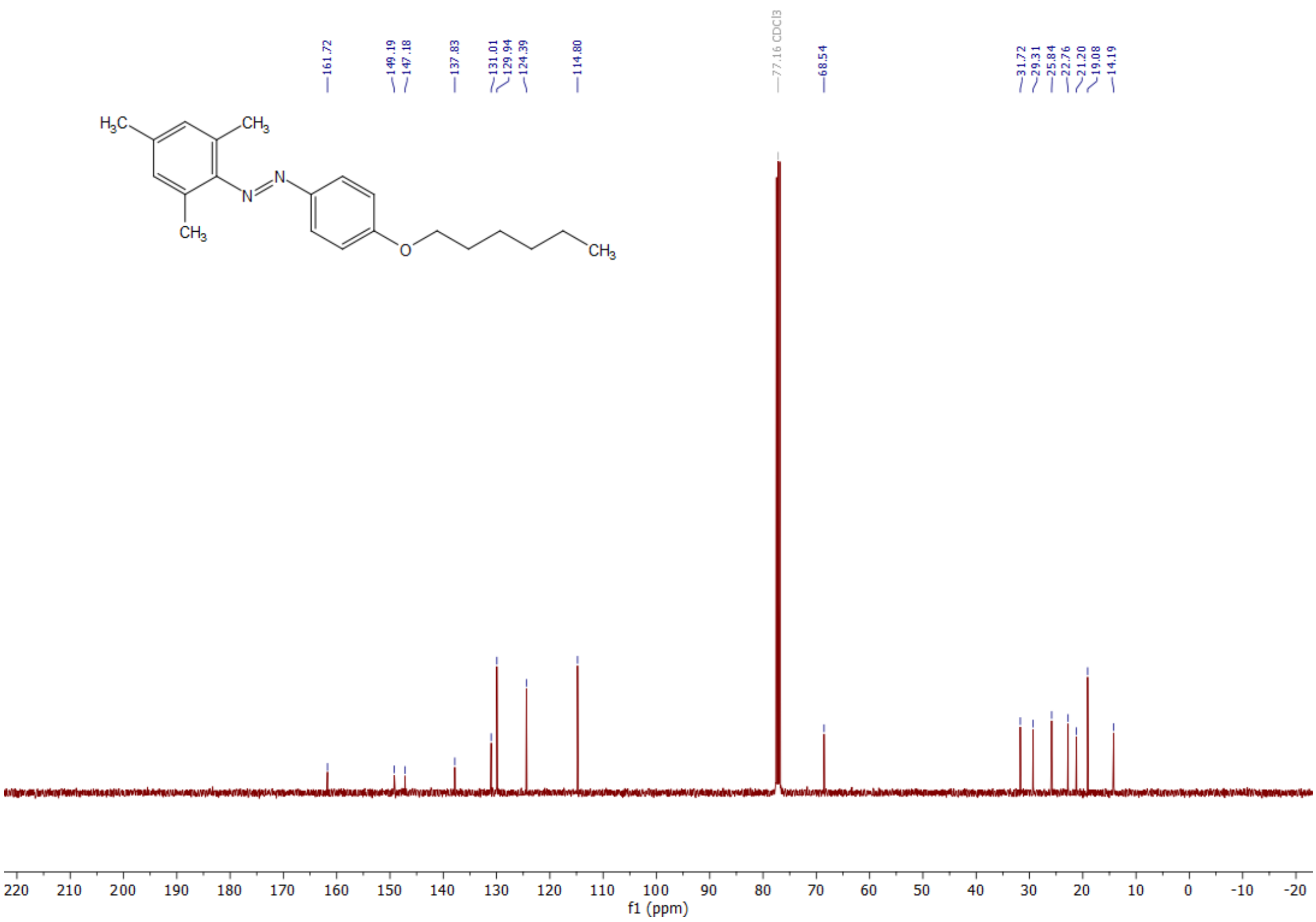

^{1}H-NMR of 1-(3,5-Dimethylphenyl)-2-[4-(hexoxy)phenyl]diazene (4p)

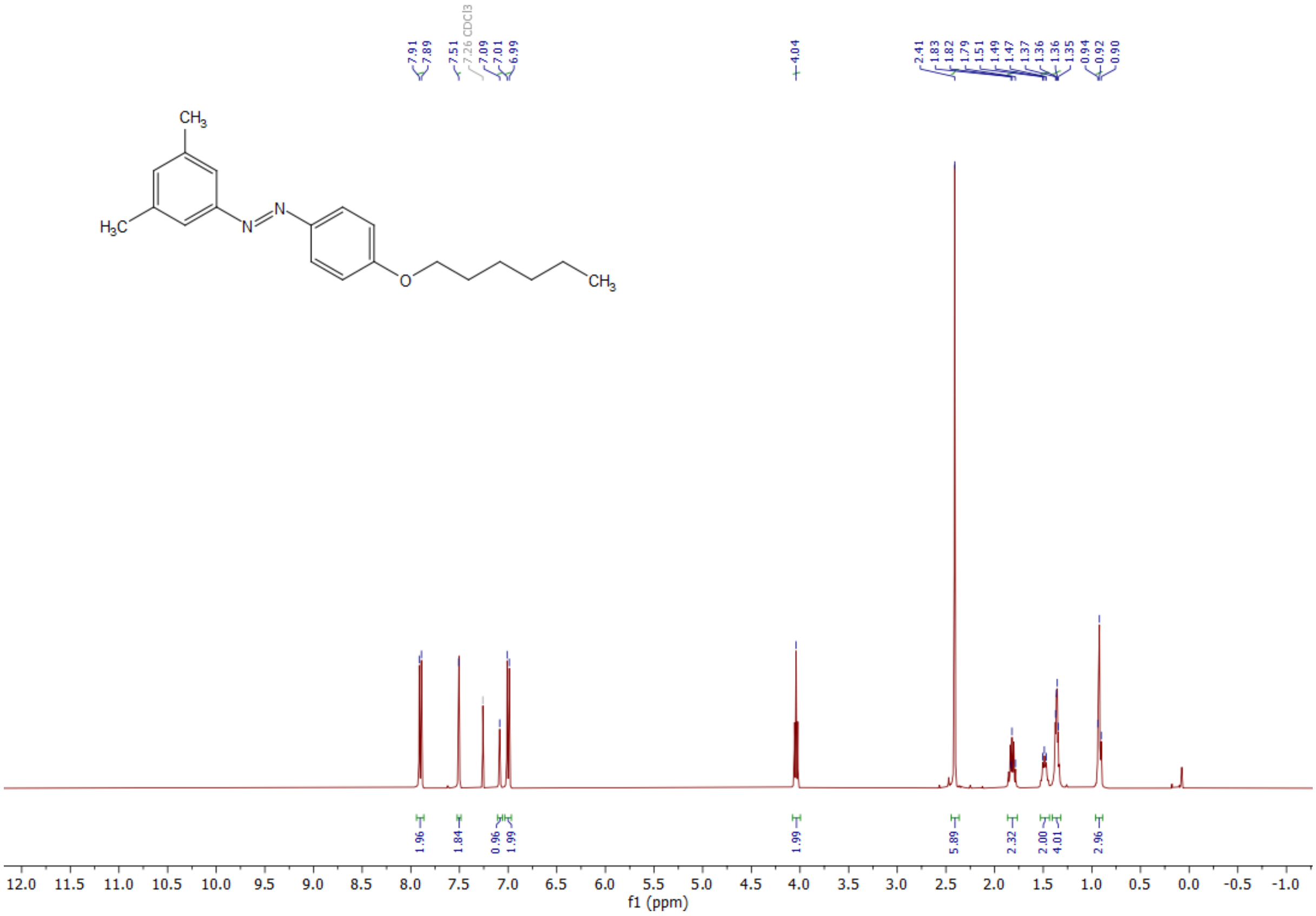

^{13}C-NMR of 1-(3,5-Dimethylphenyl)-2-[4-(hexoxy)phenyl]diazene (4p)

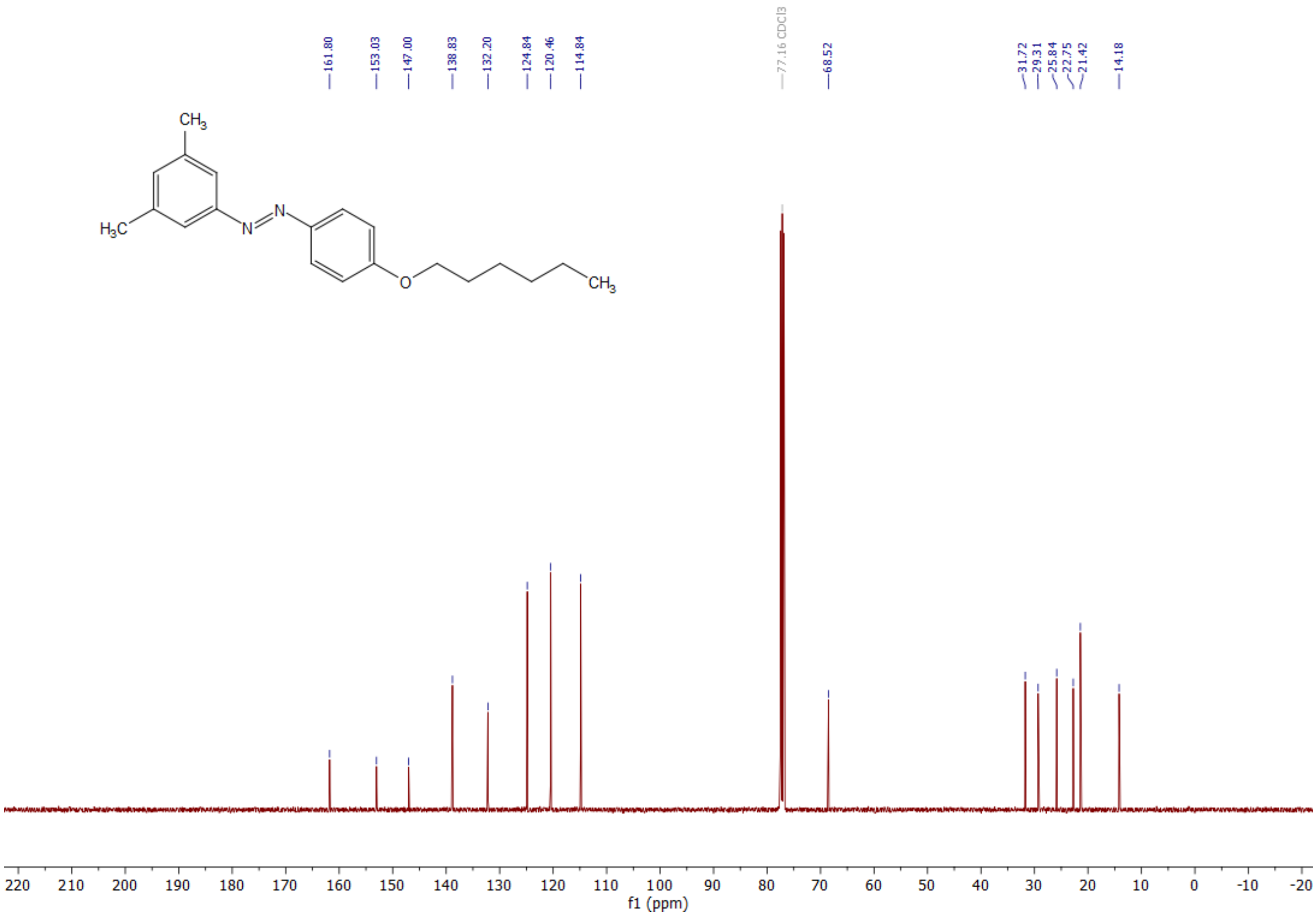

^{1}H-NMR of 1-(2,6-Difluorophenyl)-2-[4-(hexoxy)phenyl]diazene (4q)

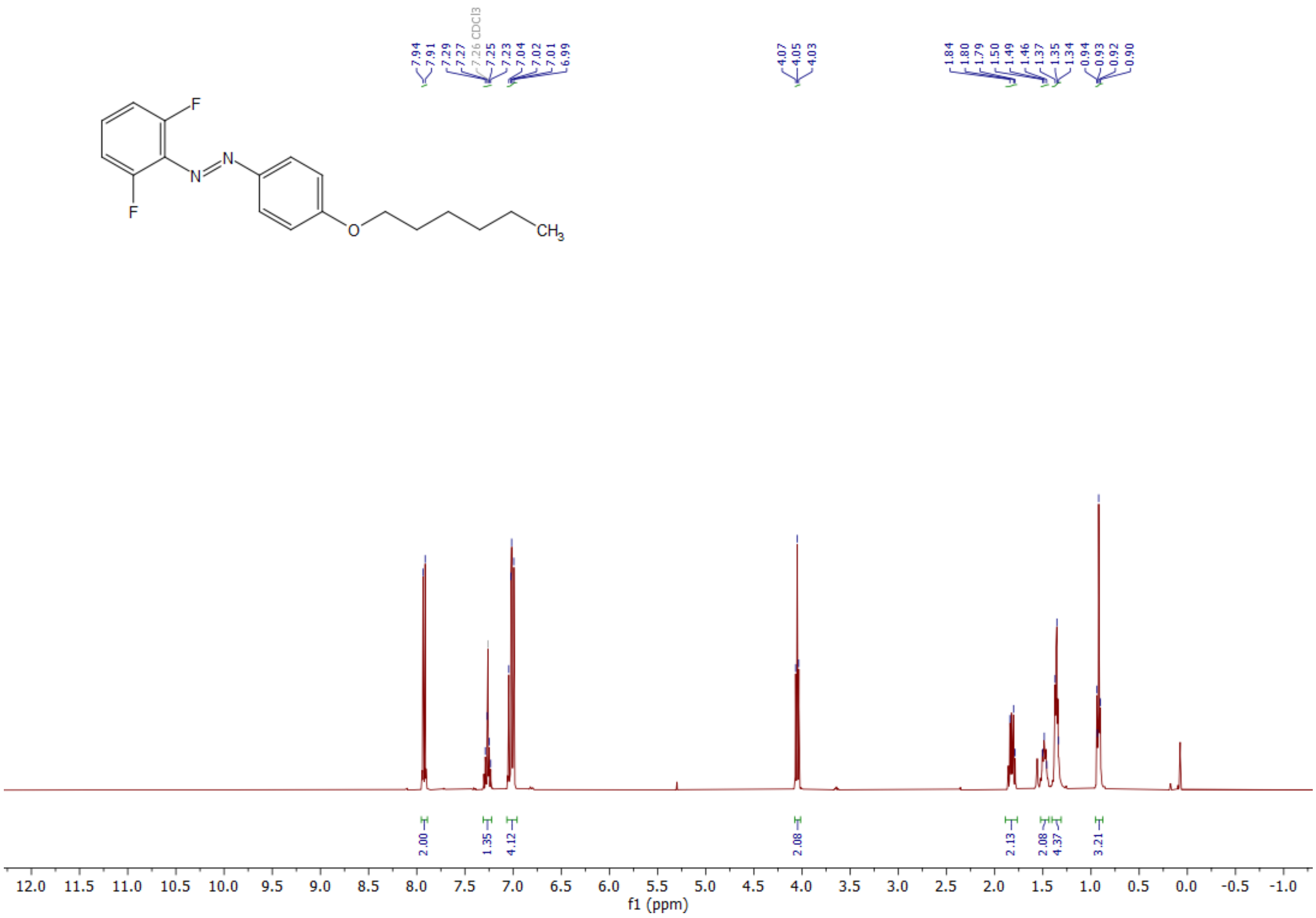

^{13}C-NMR of 1-(2,6-Difluorophenyl)-2-[4-(hexoxy)phenyl]diazene (4q)

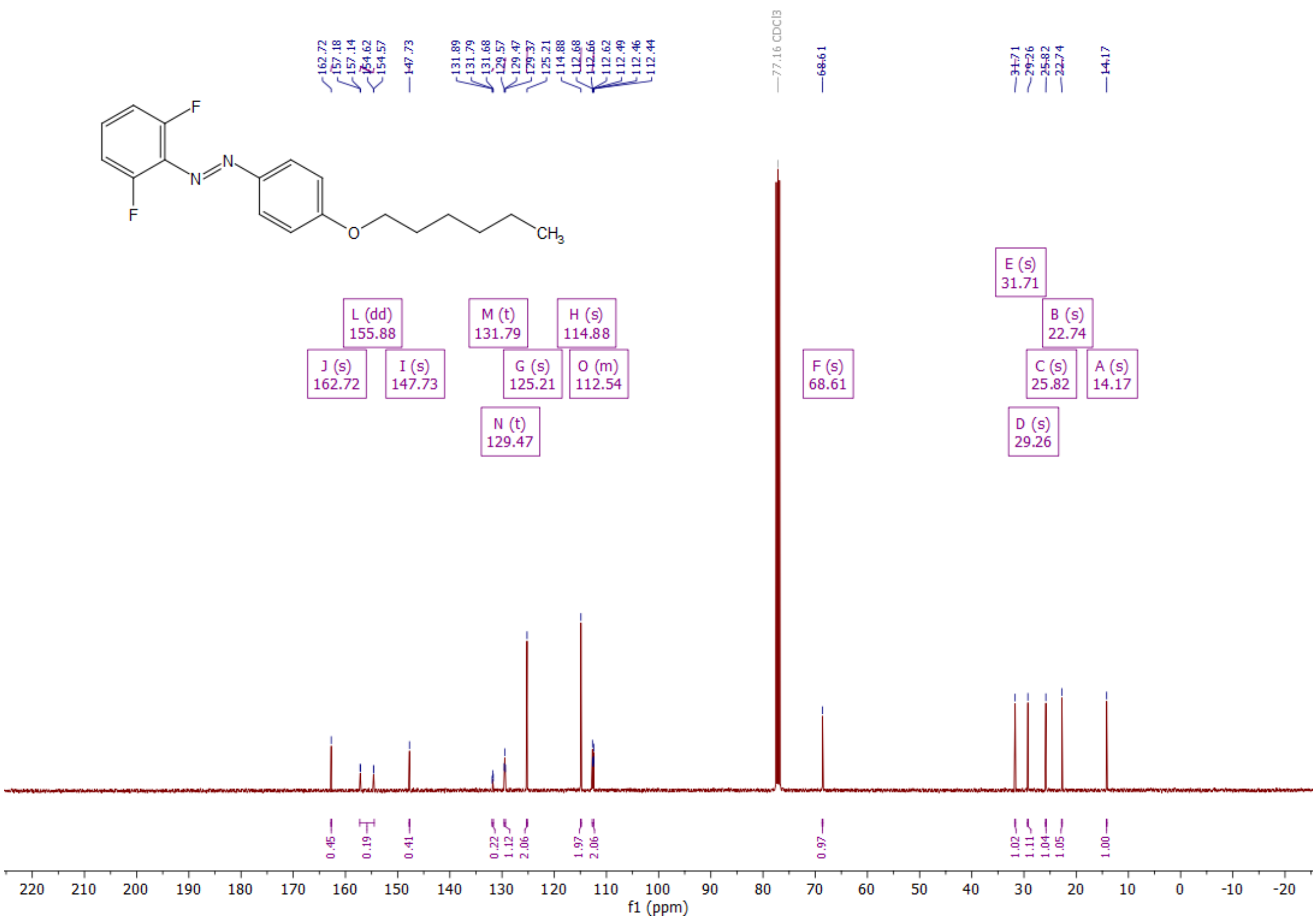

^{1}H-NMR of 1-(3-Methoxyphenyl)-2-[4-(hexoxy)phenyl]diazene (4r)

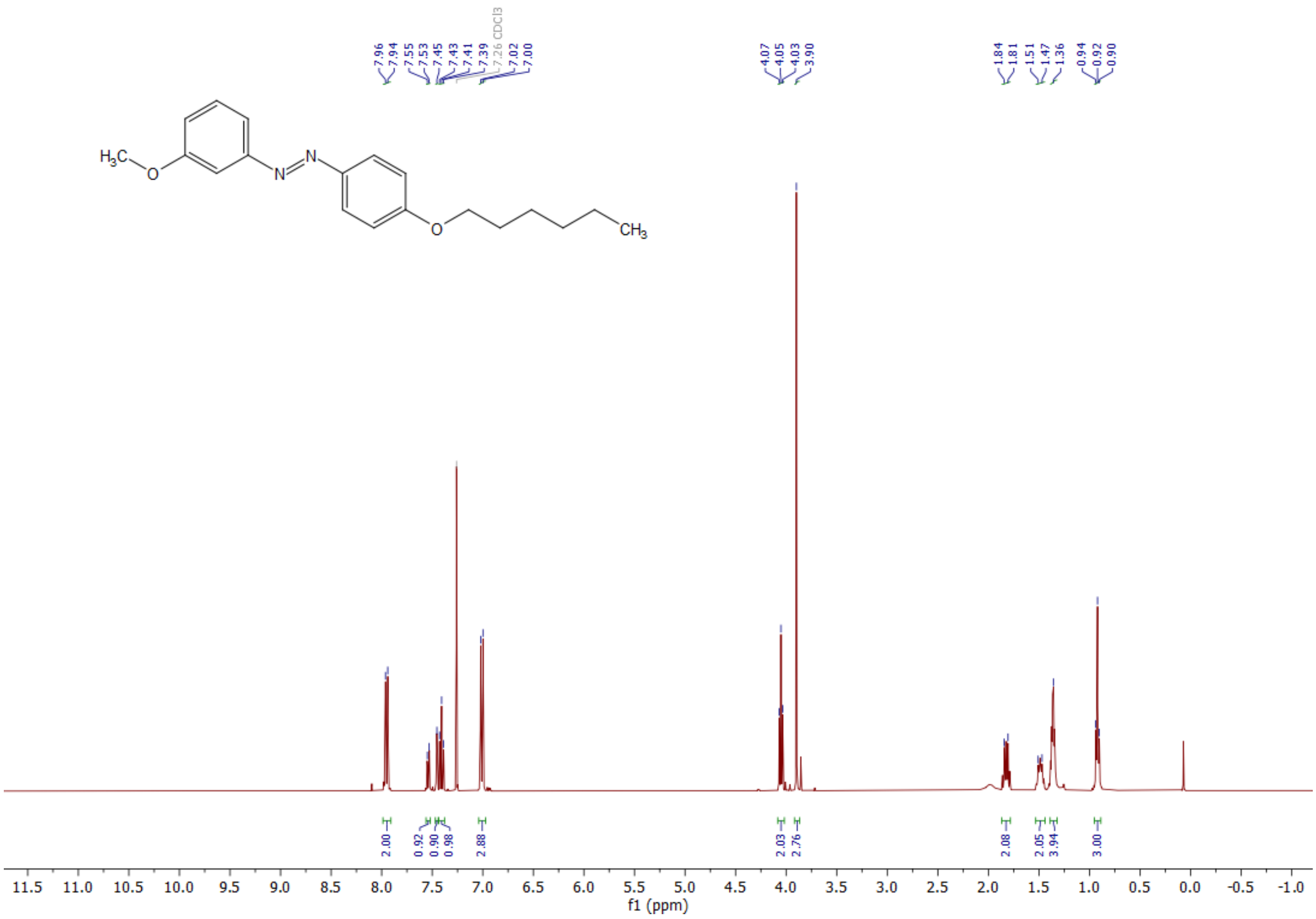

^{13}C-NMR of 1-(3-Methoxyphenyl)-2-[4-(hexoxy)phenyl]diazene (4r)

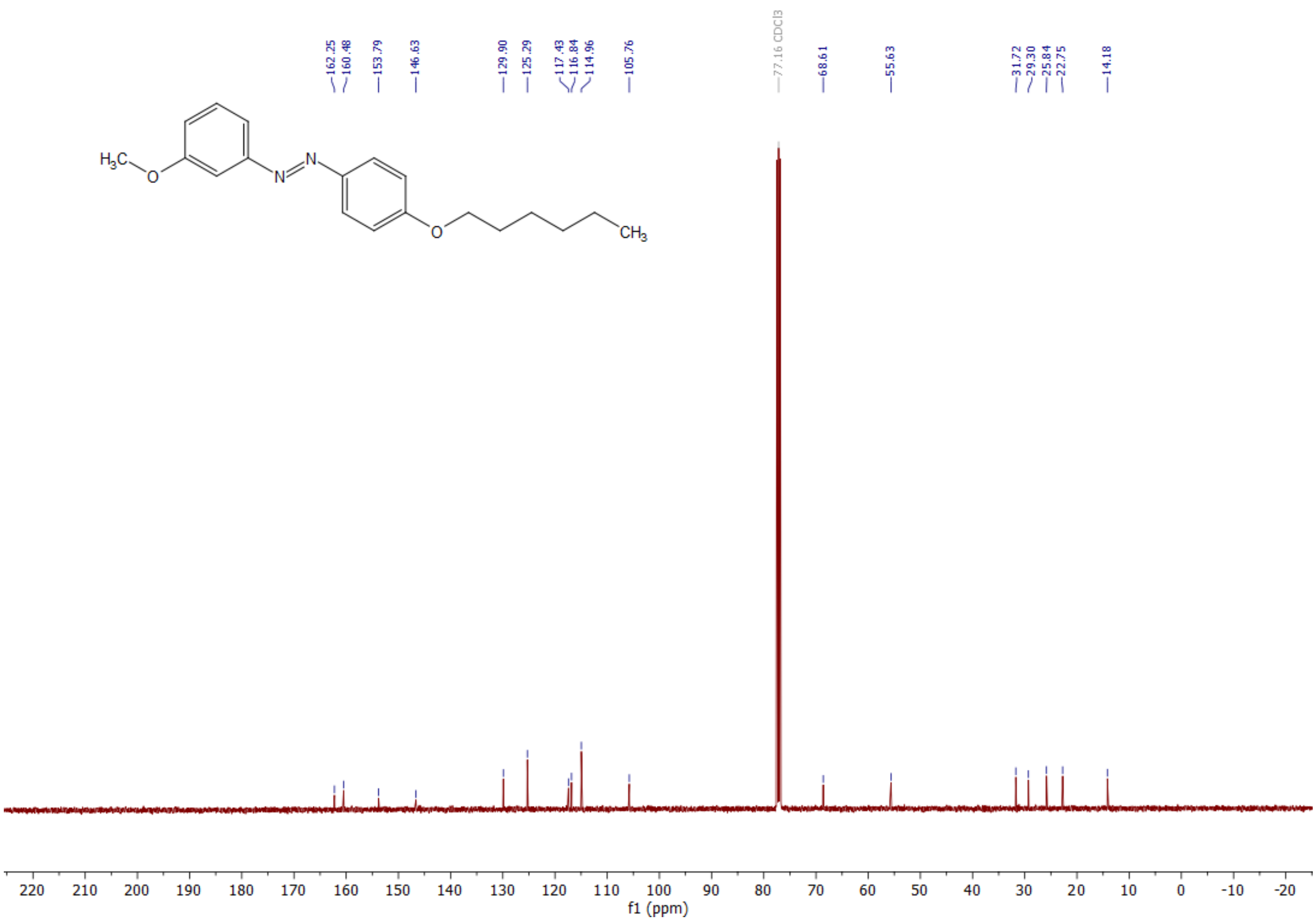

4. Irradiation experiments

UV-Vis samples were irradiated in a custom in-house built box using high power LEDs by Thorlabs powered with T-Cube™ LED Driver with Trigger Mode with max. 1200 mA. After specified irradiation times, the samples were immediately placed in the UV-Vis spectrometer and measured. The neat compounds were irradiated on a glass slide with a standard size according to DIN ISO 8037/1. For the use of thin films, the compounds were thermally melted between two glass plates, which allowed a better observation of the photoliquefication. To assess the photoliquefication of the neat compounds, the respective *p*-OHex-AB´s were applied as thin layer of powder on a glass slide and irradiated at room temperature with a wavelength of 365 nm. To exclude thermal effects, the LEDs were placed at a distance of approx. 5 cm above the sample. To determine the degree of photoisomerization, the content of the (*E*)- and (*Z*)-isomers was determined by HPLC analysis.

Table S1: LEDs used for irradiation experiments with specifications.

λ_{max} / nm	$\Delta\lambda$FWHM / nm	LED Output Power / mW	Product name
300	20	32	M300L4
325	12	35	M325L5
340	10	69.2	M340L5
365	9	1290	M365L3
385	11	1780	M385L3
405	12.5	1300	M405L4
530	35	480	M530L4
660	20	1050	M660L4

a)

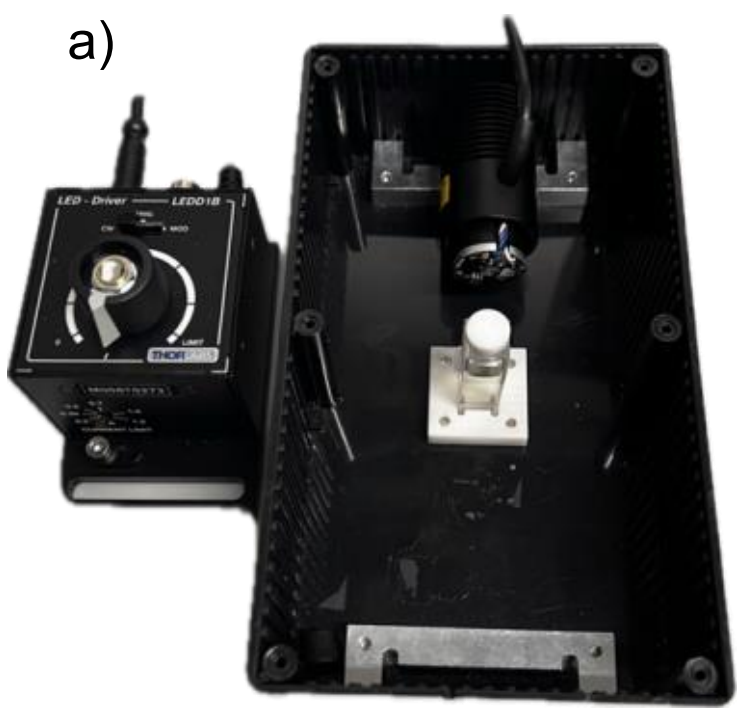

b)

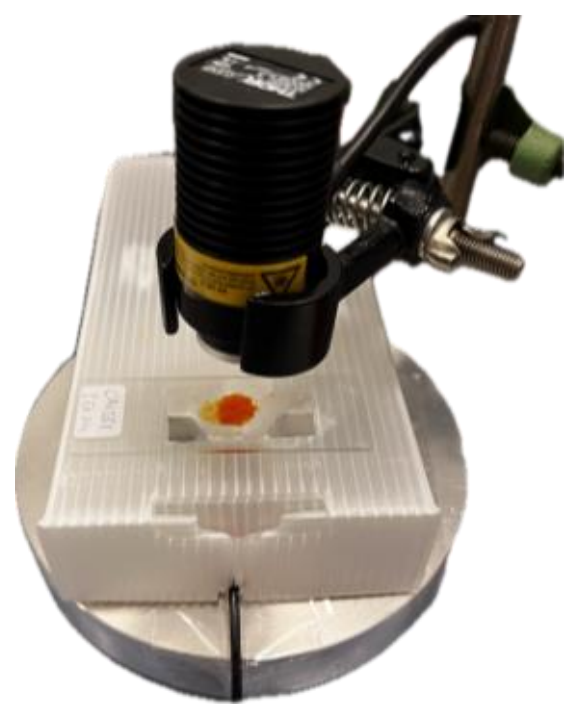

Figure S2: a) In-house build LED irradiation Box for UV-Vis cuvettes and b) Irradiation of neat compounds on a glass slide.

5. UV-Vis spectroscopy

The UV-Vis absorption spectra in solution were recorded using a SPECORD® 200 PLUS UV-Vis spectrophotometer equipped with two automatic eightfold cell changers and a Peltier thermostat system for temperature control manufactured by Analytik Jena. The spectrophotometer system was operated by the software Aspect UV from Analytik Jena. The samples were measured in QS High Precision Cells made of Quartz Suprasil® by Hellma Analytics with a light path of 10 mm. The sample volume was 3 mL and the sample concentration 5 x 10^{-5} M. As solvent UV-Vis grated acetonitrile (ACN) and water was used from VWR chemicals was used.

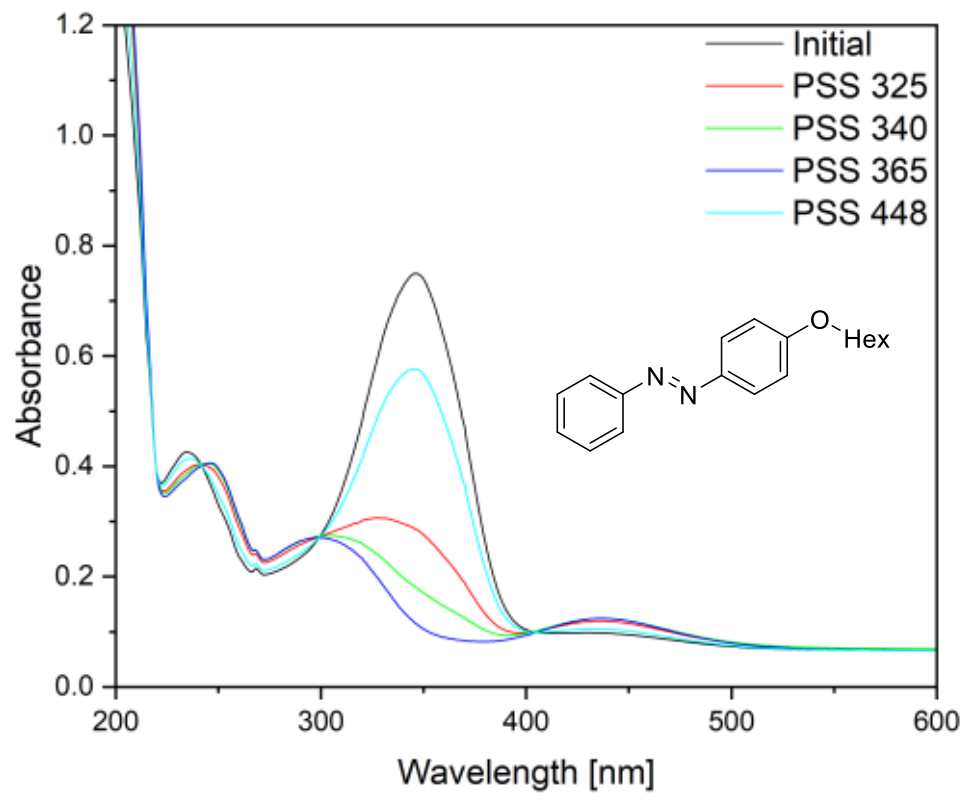

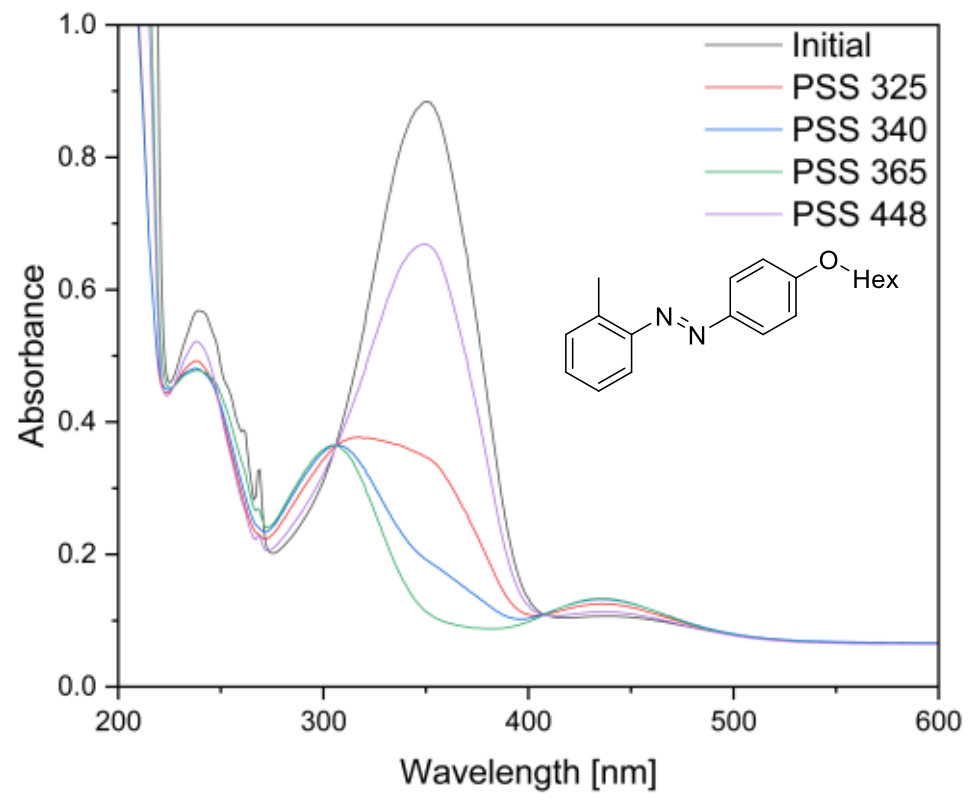

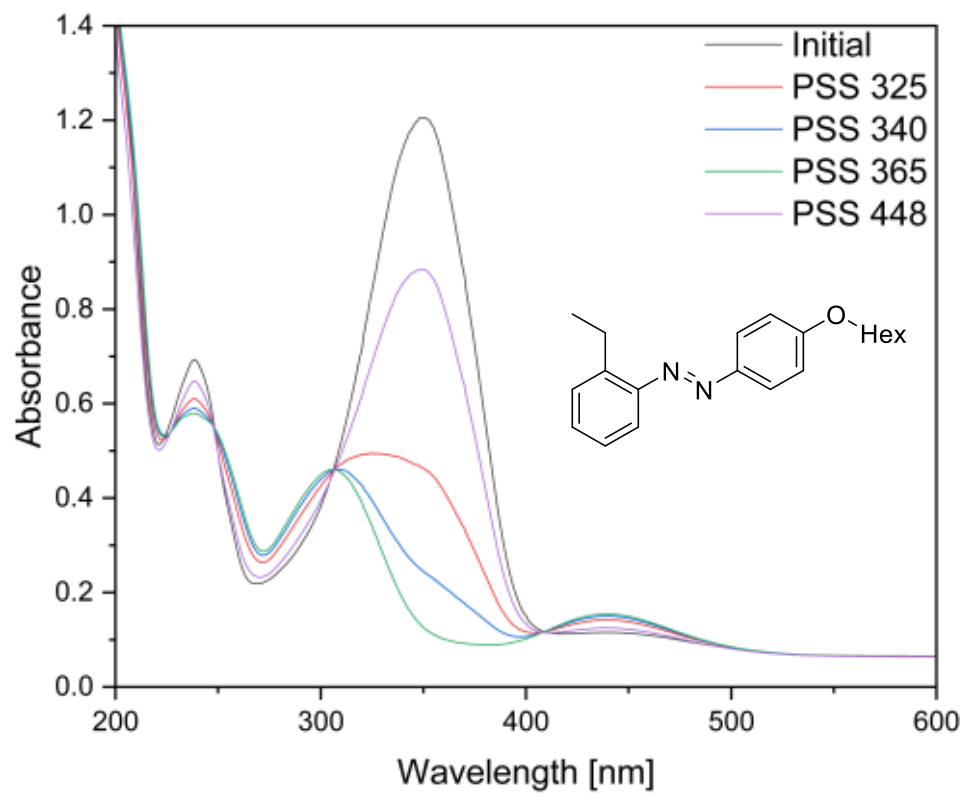

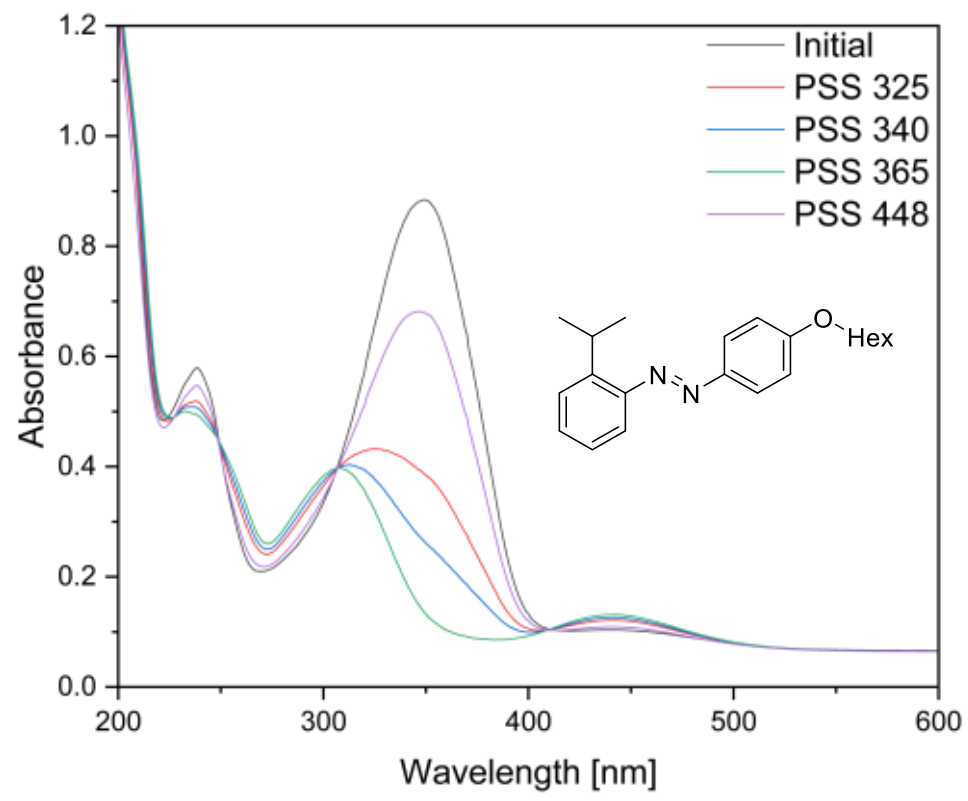

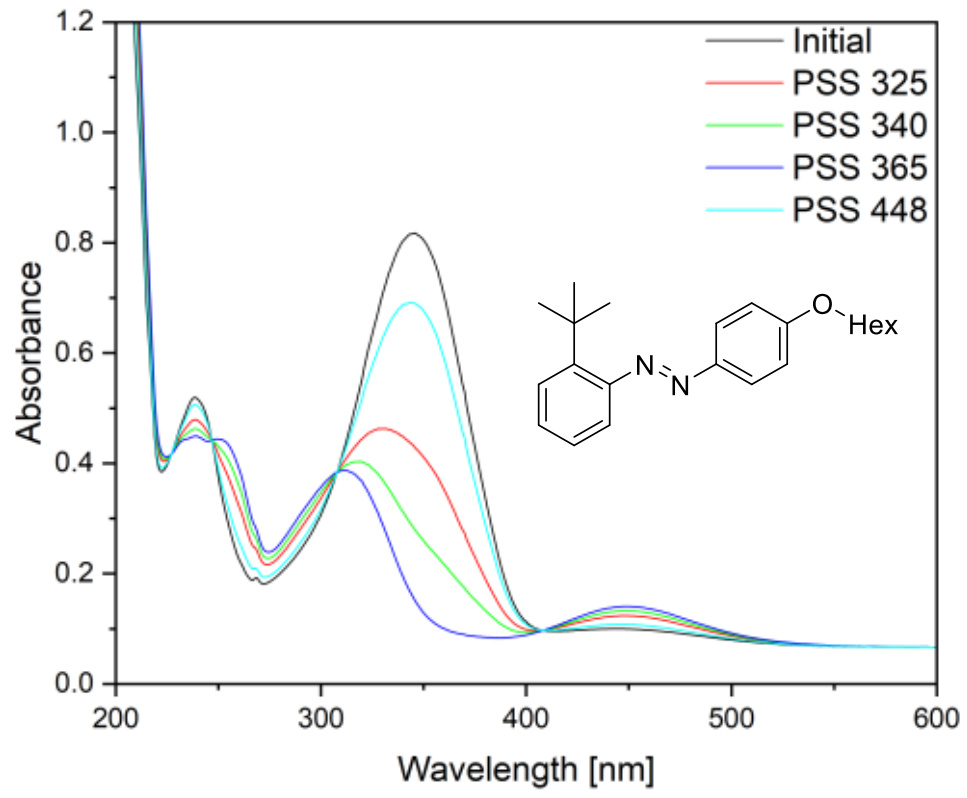
Initial
PSS 325
PSS 340
PSS 365
PSS 448
Absorbance
Wavelength [nm]
N
N
O
Hex

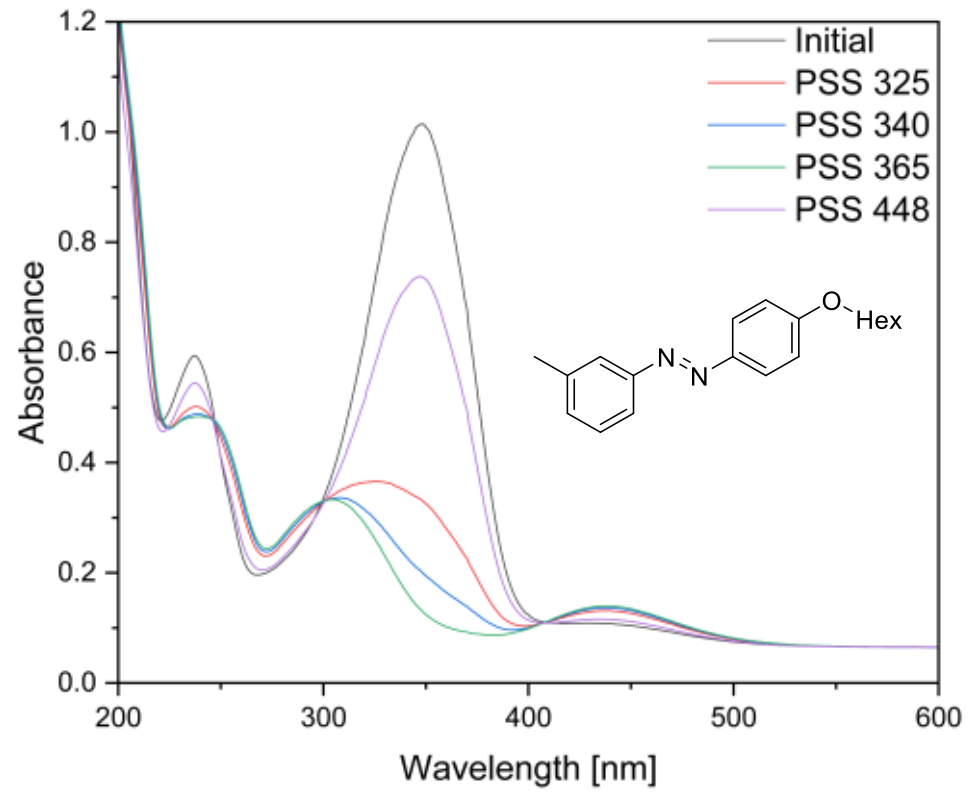
Initial
PSS 325
PSS 340
PSS 365
PSS 448
Absorbance
Wavelength [nm]
N
N
O
Hex

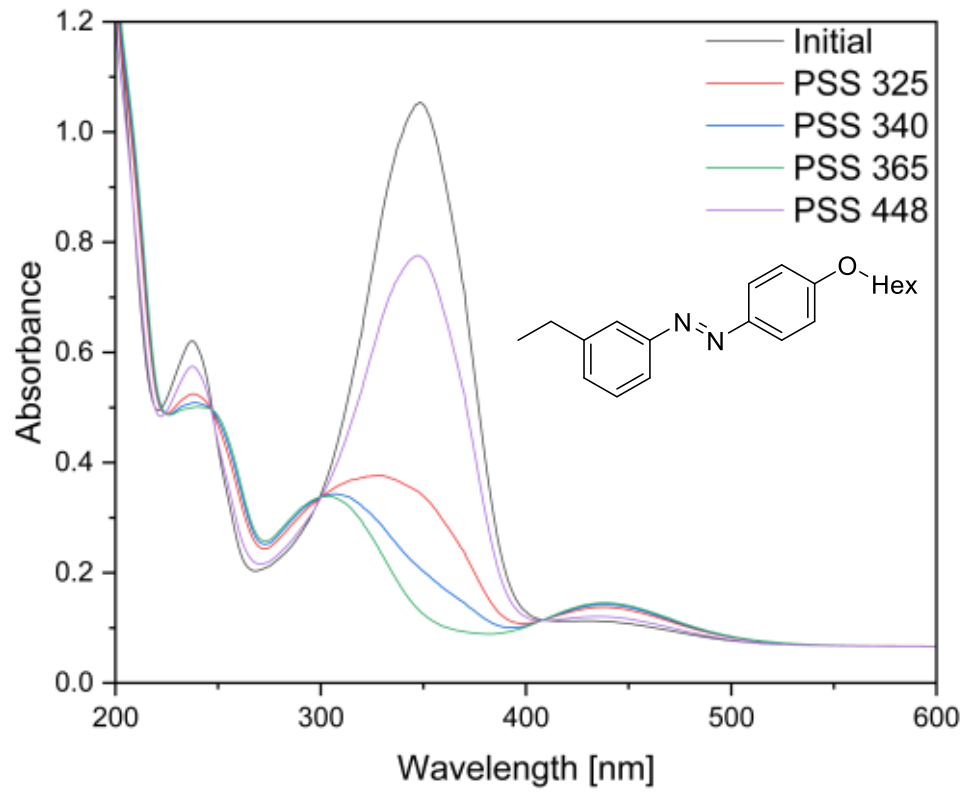
Initial
PSS 325
PSS 340
PSS 365
PSS 448
Absorbance
Wavelength [nm]
N
N
O
Hex

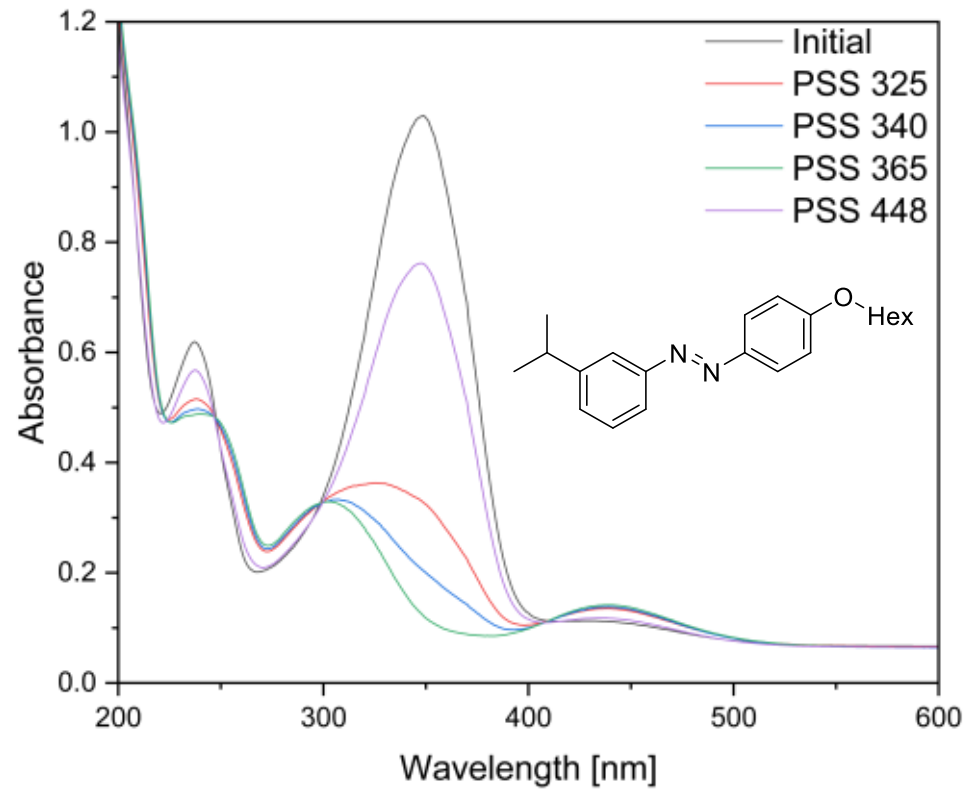
Initial
PSS 325
PSS 340
PSS 365
PSS 448
Absorbance
Wavelength [nm]
N
N
O
Hex

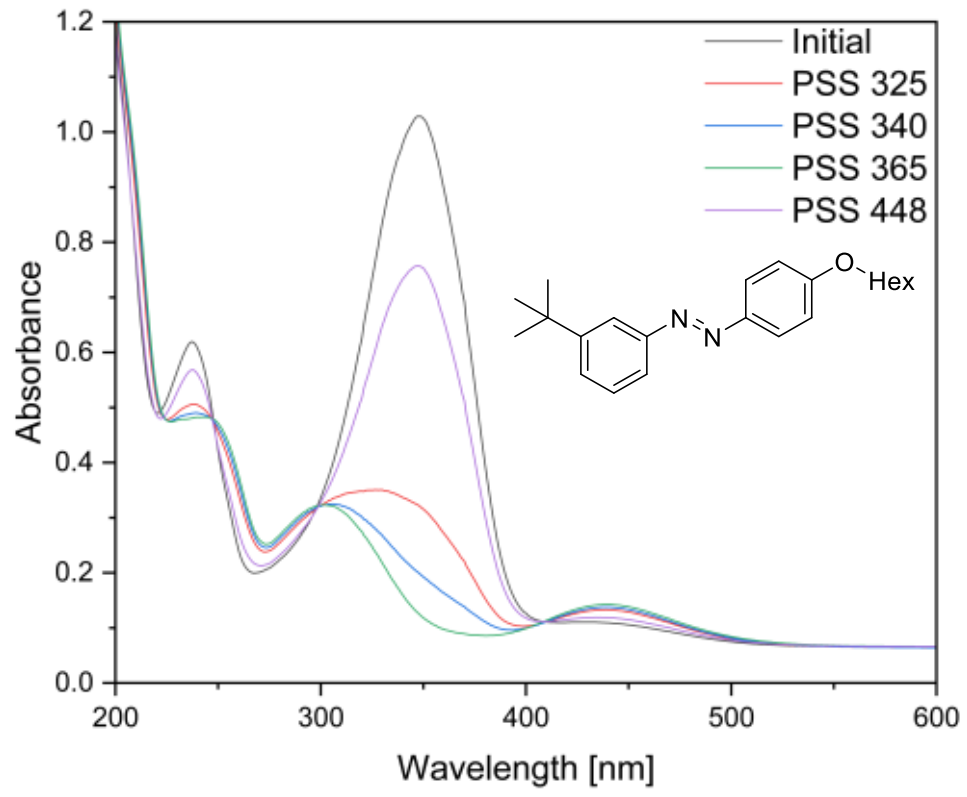
Initial
PSS 325
PSS 340
PSS 365
PSS 448
Absorbance
Wavelength [nm]
N
N
O
Hex

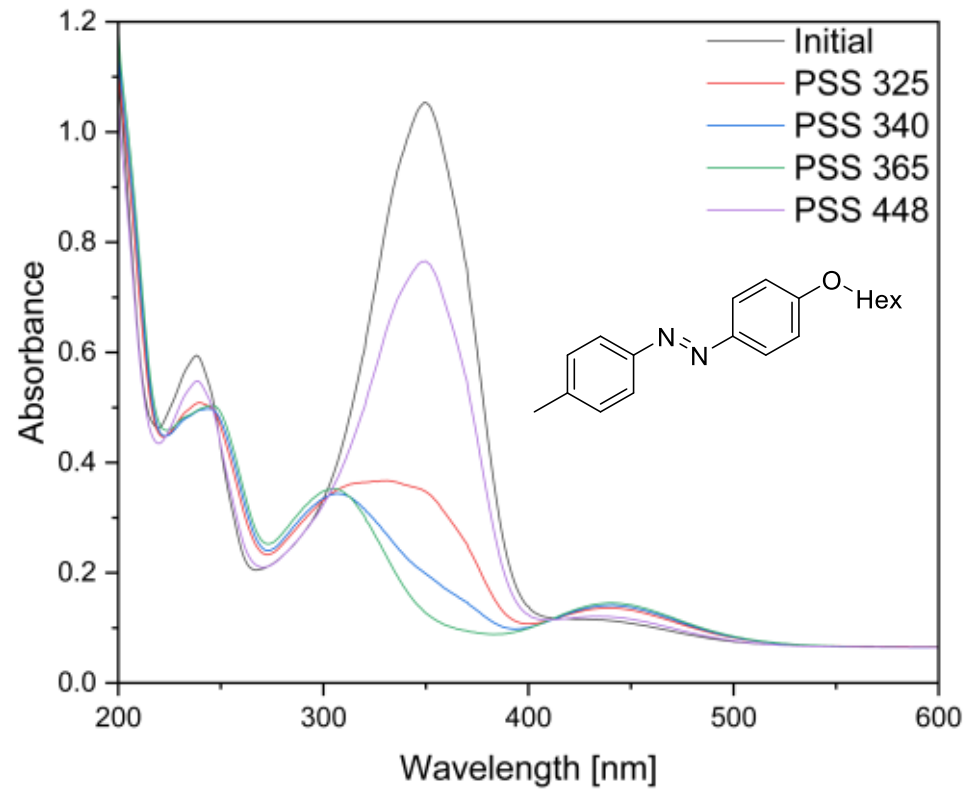
Initial
PSS 325
PSS 340
PSS 365
PSS 448
Absorbance
Wavelength [nm]
N
N
O
Hex

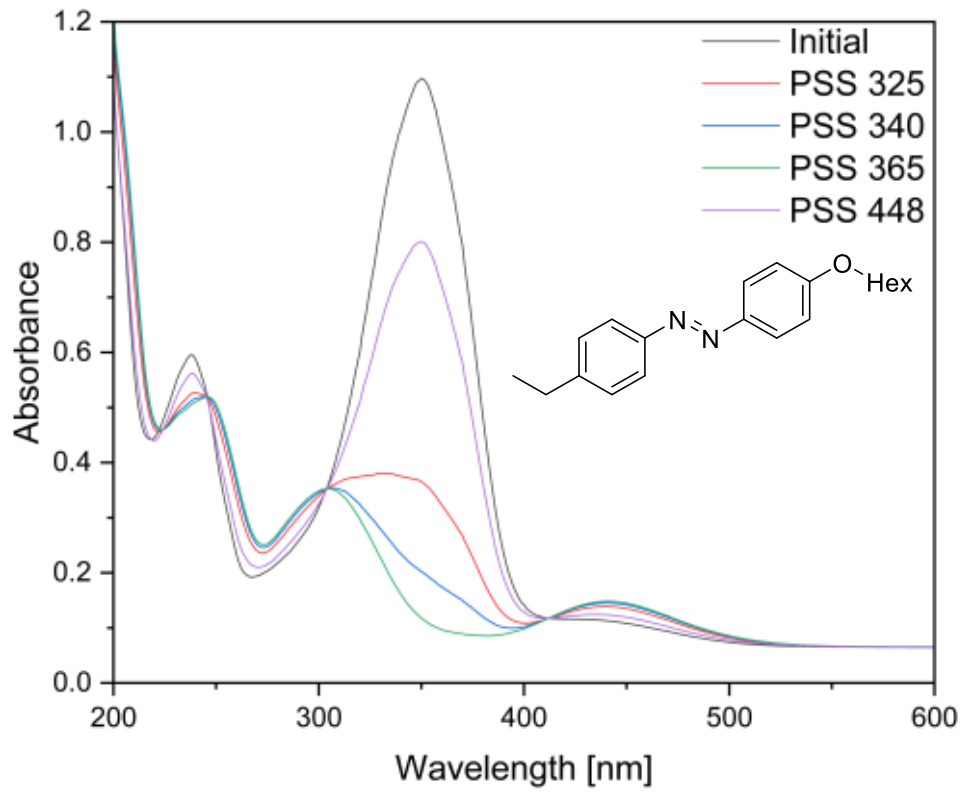
Initial
PSS 325
PSS 340
PSS 365
PSS 448
N N O Hex
Absorbance
Wavelength [nm]

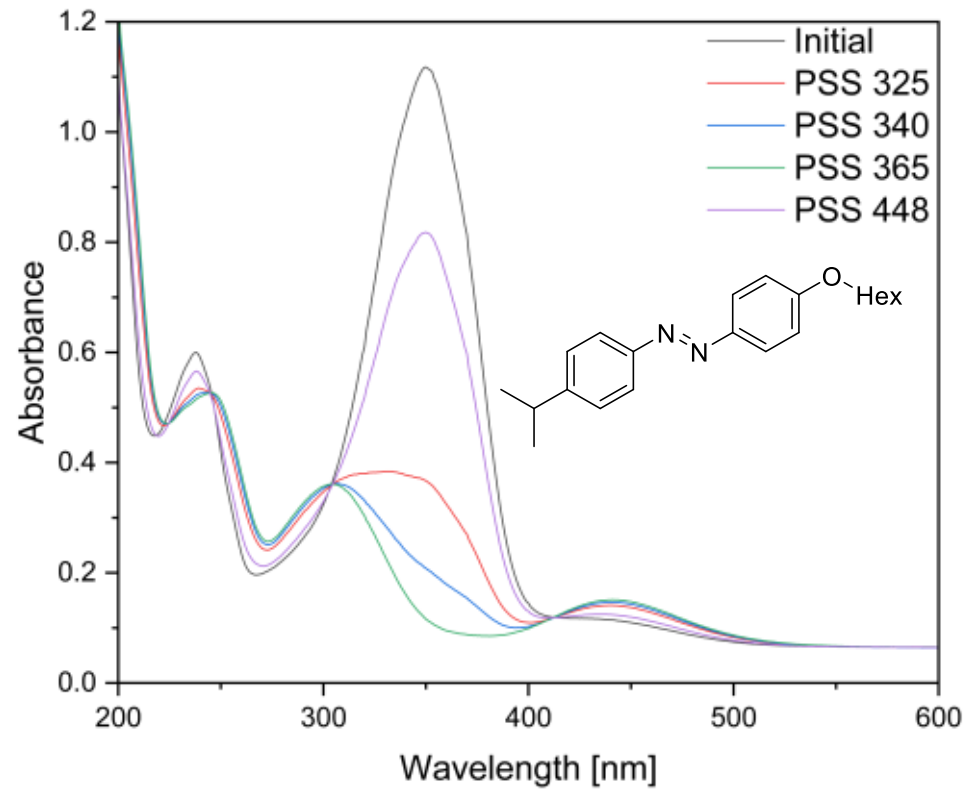
Initial
PSS 325
PSS 340
PSS 365
PSS 448
N N O Hex
Absorbance
Wavelength [nm]

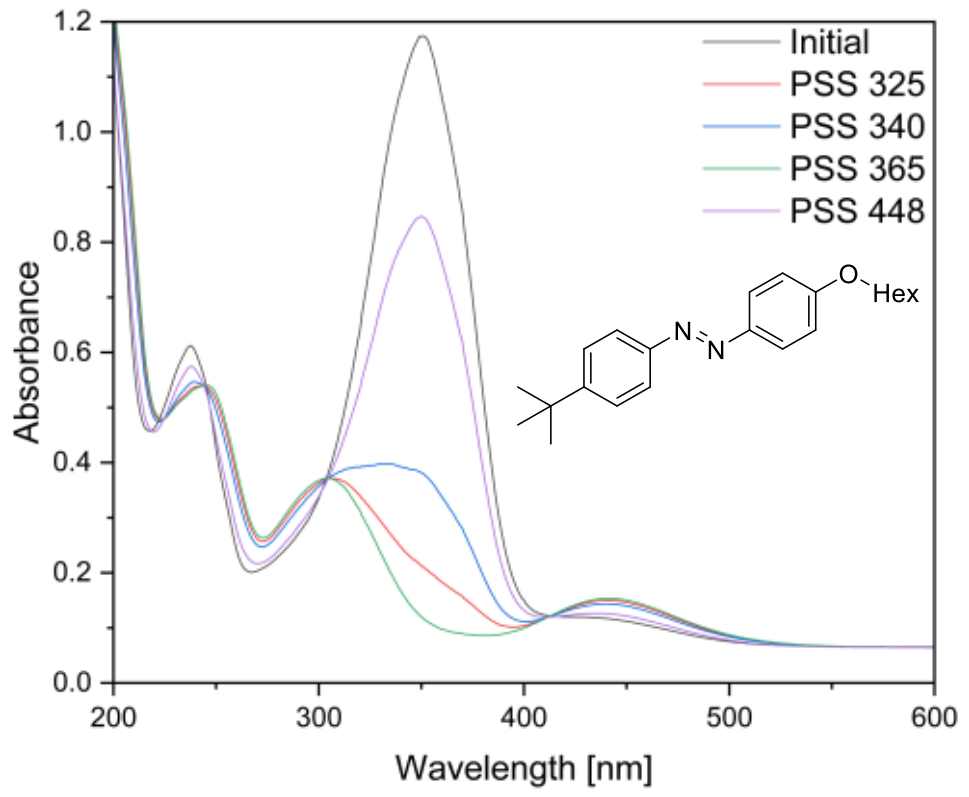
Initial
PSS 325
PSS 340
PSS 365
PSS 448
N N O Hex
Absorbance
Wavelength [nm]

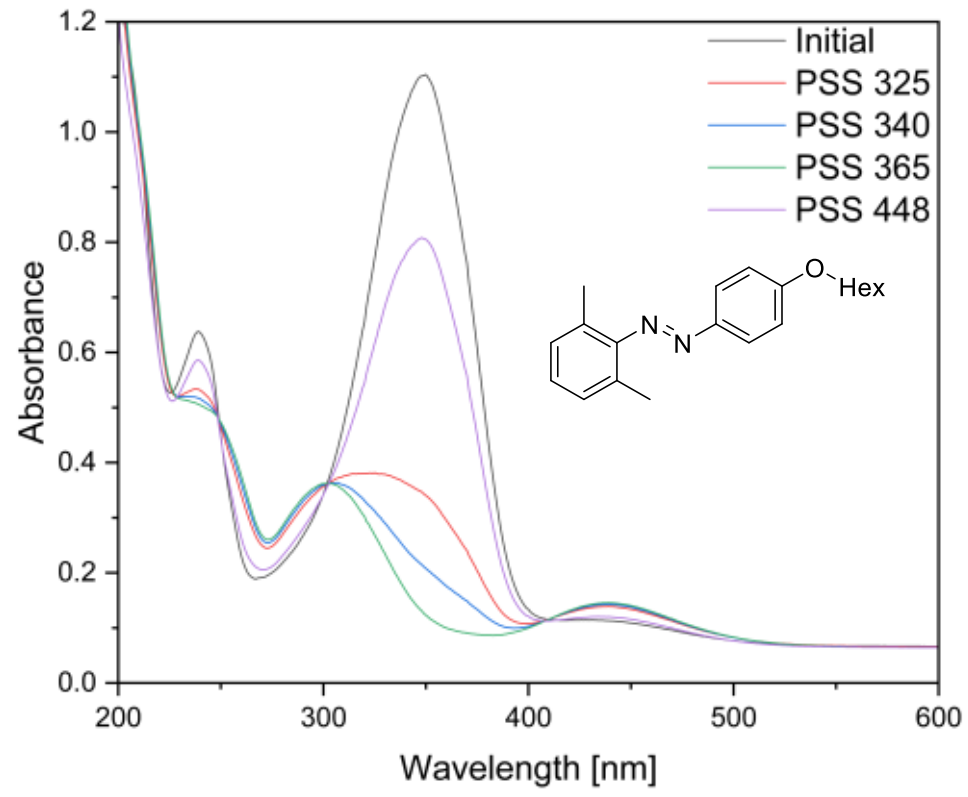
Initial
PSS 325
PSS 340
PSS 365
PSS 448
N N O Hex
Absorbance
Wavelength [nm]

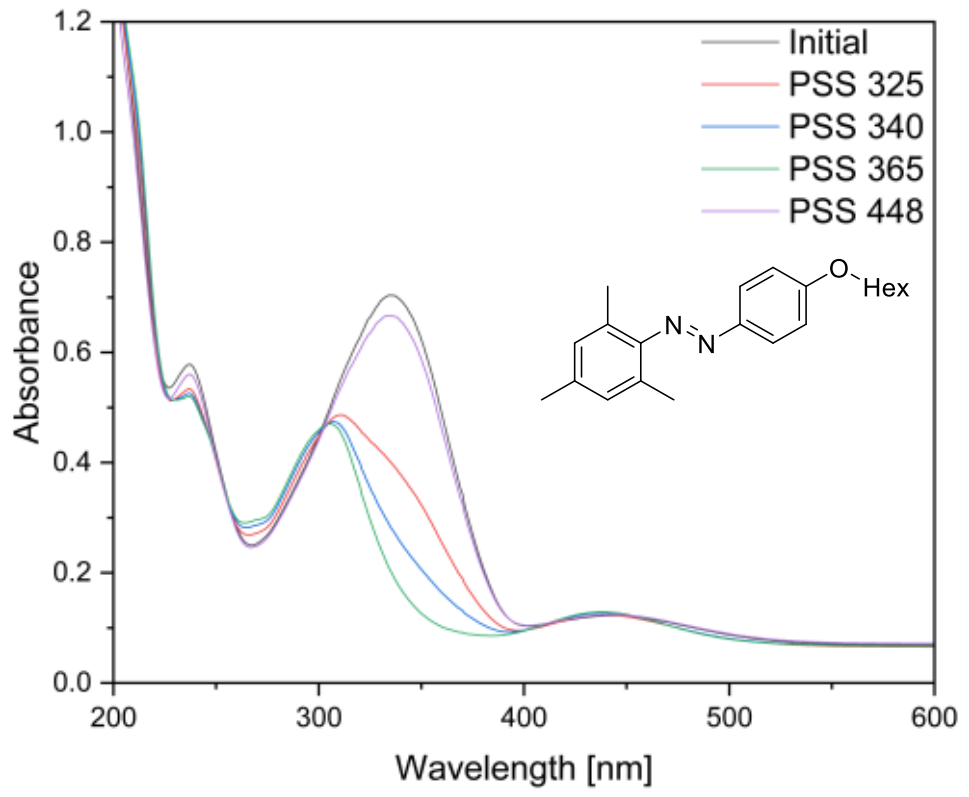
Initial
PSS 325
PSS 340
PSS 365
PSS 448
N N O Hex
Absorbance
Wavelength [nm]

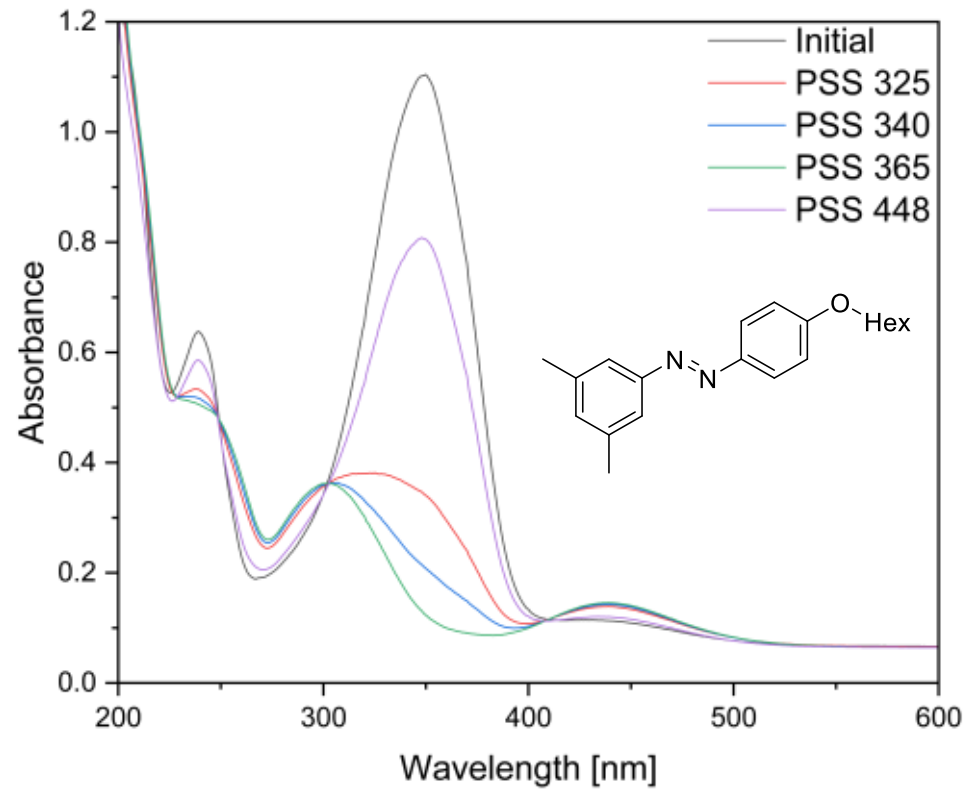
Initial
PSS 325
PSS 340
PSS 365
PSS 448
N N O Hex
Absorbance
Wavelength [nm]

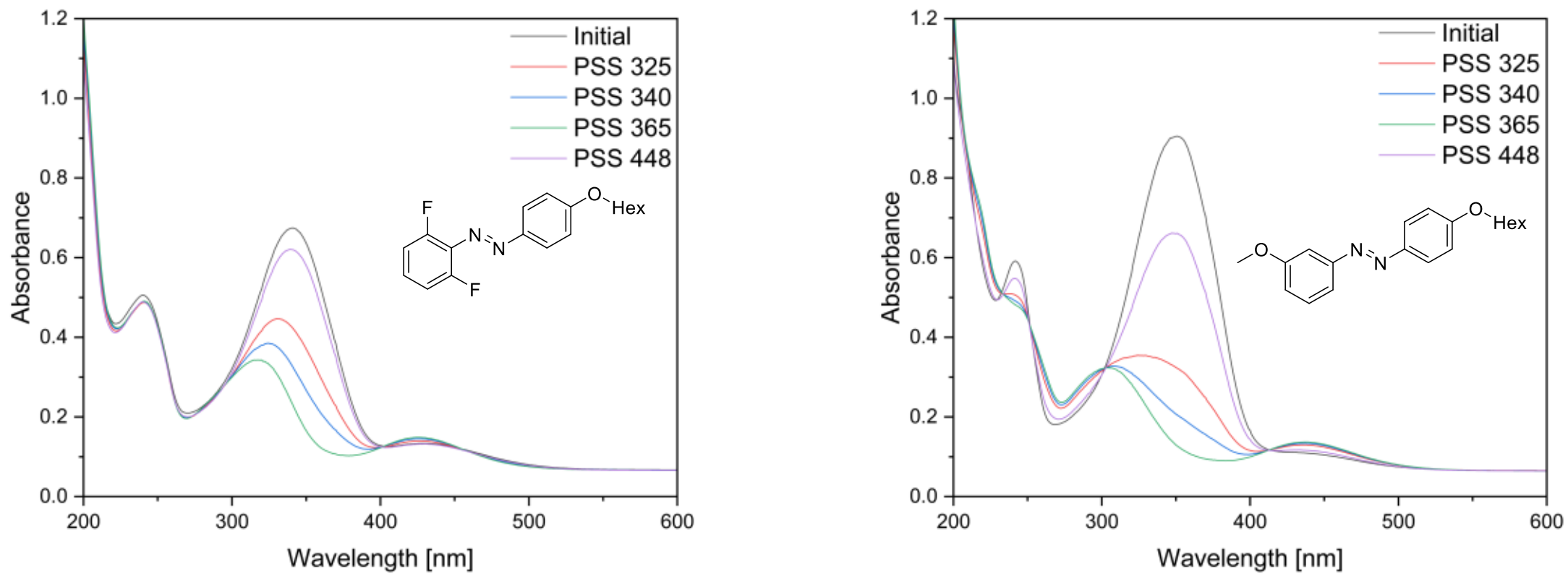

Figure S3: a) UV-Vis absorption spectra of substituted *p*-OHex-AB 4a-4r in ACN from the initial state and after irradiation with 325 nm, 340 nm, 365 nm and 448 nm.

6. Half-life in solution *via* UV-Vis spectroscopy

For determination of the half-life all samples were irradiated with 365 nm for 5-10 min. before each measurement. UV-Vis absorption spectra were recorded from 200 – 600 nm with a scan speed of 20 nm/s. The thermal isomerization was monitored in 60 min intervals at 20 °C for 60 h. The half-life of the compounds **4n**, **4o**, **4q** was measured at 40°C. All compounds were measured three-fold at concentrations of $5 \cdot 10^{-5}$ mol/L in acetonitrile. For data analysis, Origin Pro 2023 by Origin Lab Corporation was used. The absorbances *A* at the absorption maxima were plotted against the time *t* elapsed after the start of the measurements. Rate constants *k* and half-lives $t_{1/2}$ were determined after fitting the data with an Exponential Decay Function (ExpDec1, Origin) according to the literature.

$$y = y_0 + A_1 \cdot e^{-x/t_1}$$

Equation S1: Exponential Decay 1 function of Origin.

The exponential time constant t_1 was obtained by fitting the change in absorbance at absorption maximum vs. time. The time constant t_1 was further used to calculate the rate constant *k* by Equation S2 and the half-life $t_{1/2}$ by Equation S3.

$$k = 1 / t_1$$

Equation S2: Calculation of rate constant *k*.

$$t_{1/2} = \ln 2 \cdot t_1$$

Equation S3: Calculation of half-life $t_{1/2}$.

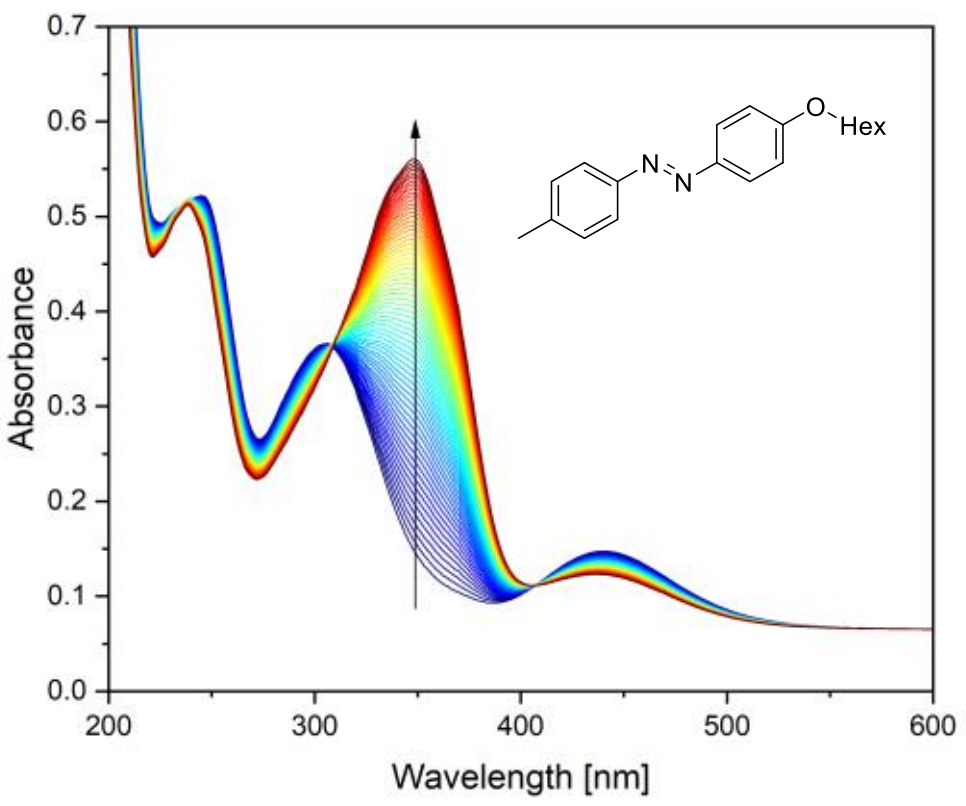

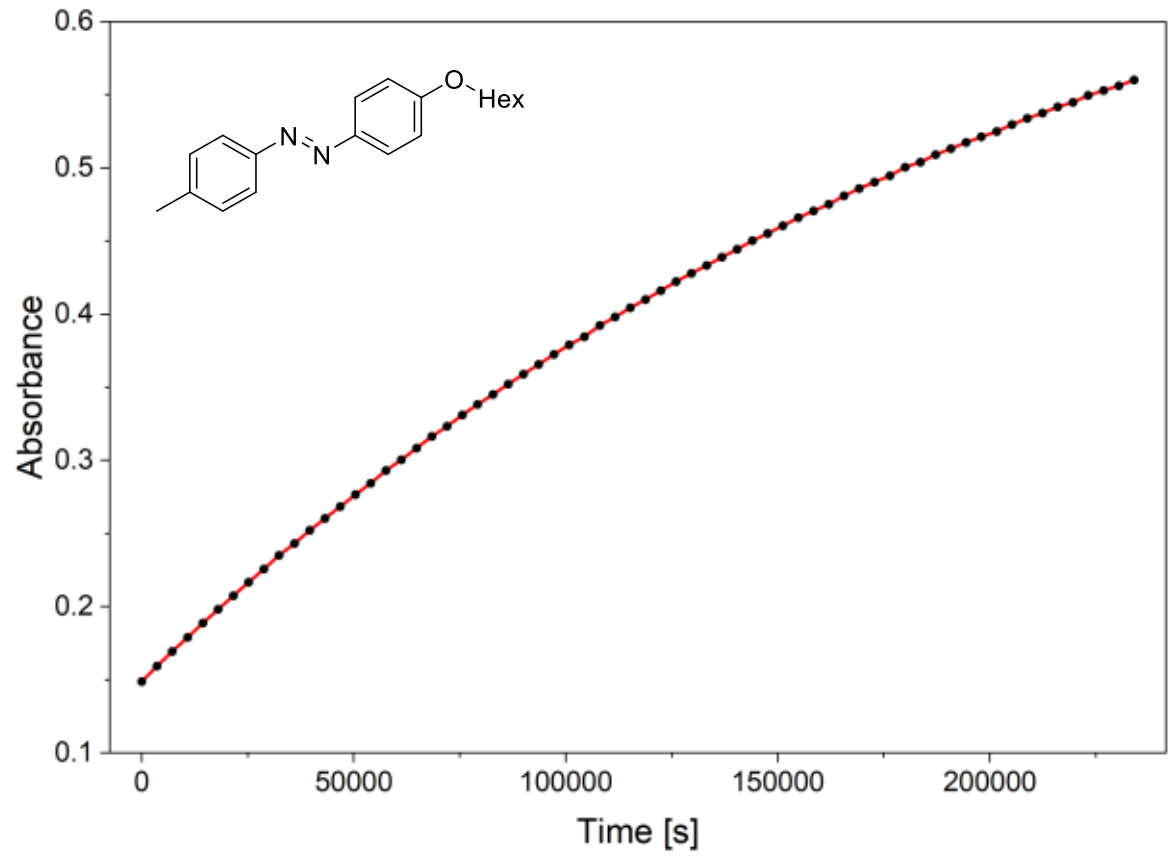

Figure S4: a) Example of UV-Vis absorption spectra during thermal relaxation at 20 °C in ACN and b) change of the absorption maxima at 350 nm.

Table S2: Kinetic constants for *p*-OHex-ABs in ACN with 5 x 10^{-5} M at 20 °C and 40 °C.

Compound	k_1 / 10^{-6} s^{-1}	k_2 / 10^{-6} s^{-1}	k_3 / 10^{-6} s^{-1}	$k_\varnothing$ / 10^{-6} s^{-1}	σ *k* / 10^{-7} s^{-1}
**4a**: R = H	2.537	2.587	2.602	2.575	0.28
**4b**: R = 2-Me	1.539	1.566	1.689	1.598	0.65
**4c**: R = 2-Et	2.168	2.170	2.246	2.195	0.36
**4d**: R = 2-*i*Pr	1.988	1.956	1.967	1.970	0.13
**4e**: R = 2-*t*Bu	2.419	2.261	2.189	2.290	0.96
**4f**: R = 3-Me	1.890	1.803	1.992	1.895	0.77
**4g**: R = 3-Et	1.823	1.815	1.784	1.807	0.17
**4h**: R = 3-*i*Pr	2.459	2.404	2.376	2.413	0.34
**4i**: R = 3-*t*Bu	1.562	1.560	1.570	1.564	0.04
**4j**: R = 4-Me	4.468	4.364	4.595	4.476	0.94
**4k**: R = 4-Et	4.062	4.003	4.046	4.037	0.25
**4l**: R = 4-*i*Pr	3.390	3.405	3.394	3.396	0.06
**4m**: R = 4-*t*Bu	3.462	3.456	3.417	3.445	0.20
**4n**: R = 2,6-Me[a]	0.278	0.251	0.272	0.267	0.01
**4o**: R = 2,4,6-Me[a]	0.185	0.190	0.198	0.191	0.05
**4p**: R = 3,5-Me	2.453	2.428	2.544	2.475	0.50
**4q**: R = 2,6-F[a]	1.197	1.320	1.244	1.254	0.51
**4r**: R = 3-OMe	1.480	1.482	1.532	1.498	0.24

[a] measured at 40°C

Table S3: Half-lives at 20 °C or 40 °C for *p*-alkoxy-ABs in ACN 5 x 10^-5^ M.

Compound	1. $t_{1/2}$ / h	2. $t_{1/2}$ / h	3. $t_{1/2}$ / h	⌀ $t_{1/2}$ / h	σ $t_{1/2}$ / h
**4a**: R = H	75.89	74.43	74.00	74.77	0.81
**4b**: R = 2-Me	125.13	122.93	114.02	120.69	4.80
**4c**: R = 2-Et	88.80	88.71	85.73	87.75	1.43
**4d**: R = 2-*i*Pr	96.87	98.42	97.88	97.72	0.64
**4e**: R = 2-*t*Bu	79.60	85.16	87.96	84.24	3.48
**4f**: R = 3-Me	101.87	106.80	96.68	101.78	4.14
**4g**: R = 3-Et	105.63	106.11	107.91	106.55	0.98
**4h**: R = 3-*i*Pr	78.30	80.10	81.02	79.80	1.13
**4i**: R = 3-*t*Bu	123.26	123.39	122.66	123.11	0.32
**4j**: R = 4-Me	43.10	44.12	41.90	43.04	0.91
**4k**: R = 4-Et	47.40	48.10	47.59	47.70	0.30
**4l**: R = 4-*i*Pr	56.79	56.55	56.74	56.69	0.10
**4m**: R = 4-*t*Bu	55.61	55.71	56.35	55.89	0.33
**4n**: R = 2,6-Me[a]	692.59	767.10	707.87	722.52	32.13
**4o**: R = 2,4,6-Me[a]	1040.76	1013.37	972.43	1008.85	28.08
**4p**: R = 3,5-Me	78.49	79.31	75.69	77.83	1.55
**4q**: R = 2,6-F[a]	160.85	145.86	154.78	153.83	6.16
**4r**: R = 3-OMe	130.08	129.93	125.66	128.56	2.05

[a] measured at 40°C

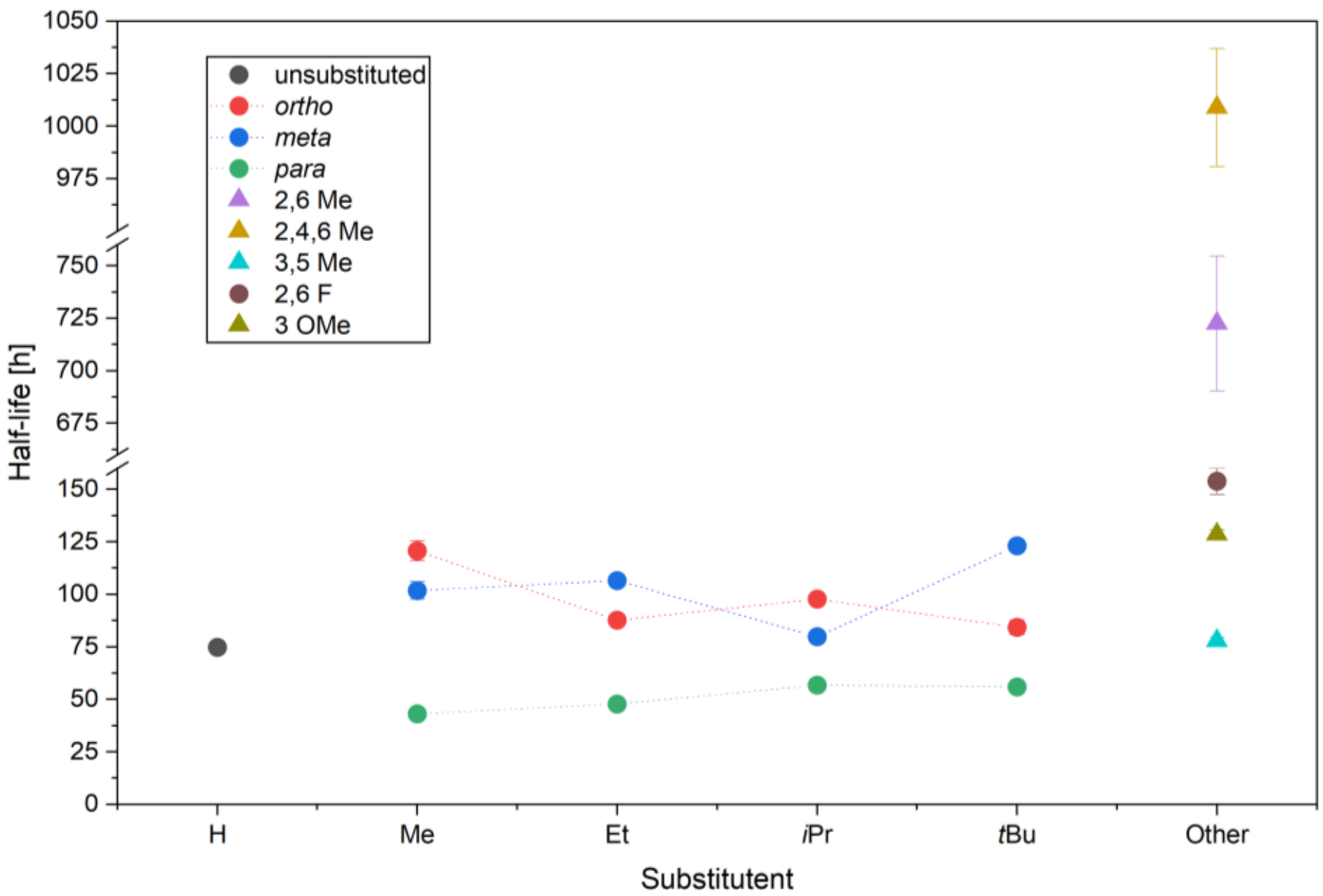

Figure S5: Thermal half-life of substituted *p*-OHex-AB 4a-4r in ACN at 20 °C and 40 °C.

7. Determination of PSS in solution and neat *via* HPLC

The PSS isomer concentrations were determined with a Shimadzu LCMS-2020 system using a 150 x 4 mm Eurospher 100^{-5} C18 column with acetonitrile/water (90:10) as isocratic eluent (1.5 mL/min). Detection was carried out using a Shimadzu SPD-M20A diode array detector at the previously determined isosbestic wavelengths. Therefore, a UV-Vis absorption spectra was measured each compound in acetonitrile (see Figure S3) and acetonitrile/water (90:10). The change from pure acetonitrile to acetonitrile/water (90:10) in the separation had only a negligible effect on the isosbestic wavelengths.

Table S4: Isosbestic points of *p*-OHex-ABs in ACN:H_2O (90:10) for determination of the composition of (*E*)- and (*Z*)-isomers *via* HPLC.

Compound	Isosbestic point / nm
**4a**: R = H	299
**4b**: R = 2-Me	307
**4c**: R = 2-Et	308
**4d**: R = 2-*i*Pr	307
**4e**: R = 2-*t*Bu	307
**4f**: R = 3-Me	301
**4g**: R = 3-Et	300
**4h**: R = 3-*i*Pr	300
**4i**: R = 3-*t*Bu	301
**4j**: R = 4-Me	305
**4k**: R = 4-Et	304
**4l**: R = 4-*i*Pr	304
**4m**: R = 4-*t*Bu	304
**4n**: R = 2,6-Me	300
**4o**: R = 2,4,6-Me	303
**4p**: R = 3,5-Me	303
**4q**: R = 2,6-F	295
**4r**: R = 3-OMe	303

By integration of the signals of the respective isomers in the PSS for 365 nm in the chromatogram at the wavelength of the isosbestic point, the composition of the isomers was determined. The samples were dissolved in 2 mL glass vials in acetonitrile with a concentration of 5 · 10^{-4} mol/L, from which aliquots of 10 µL were injected for analysis. For all determined isomer ratios, an error of ±1% is assumed. Each dissolved sample was irradiated with 365 nm for 5 - 10 min.

a)

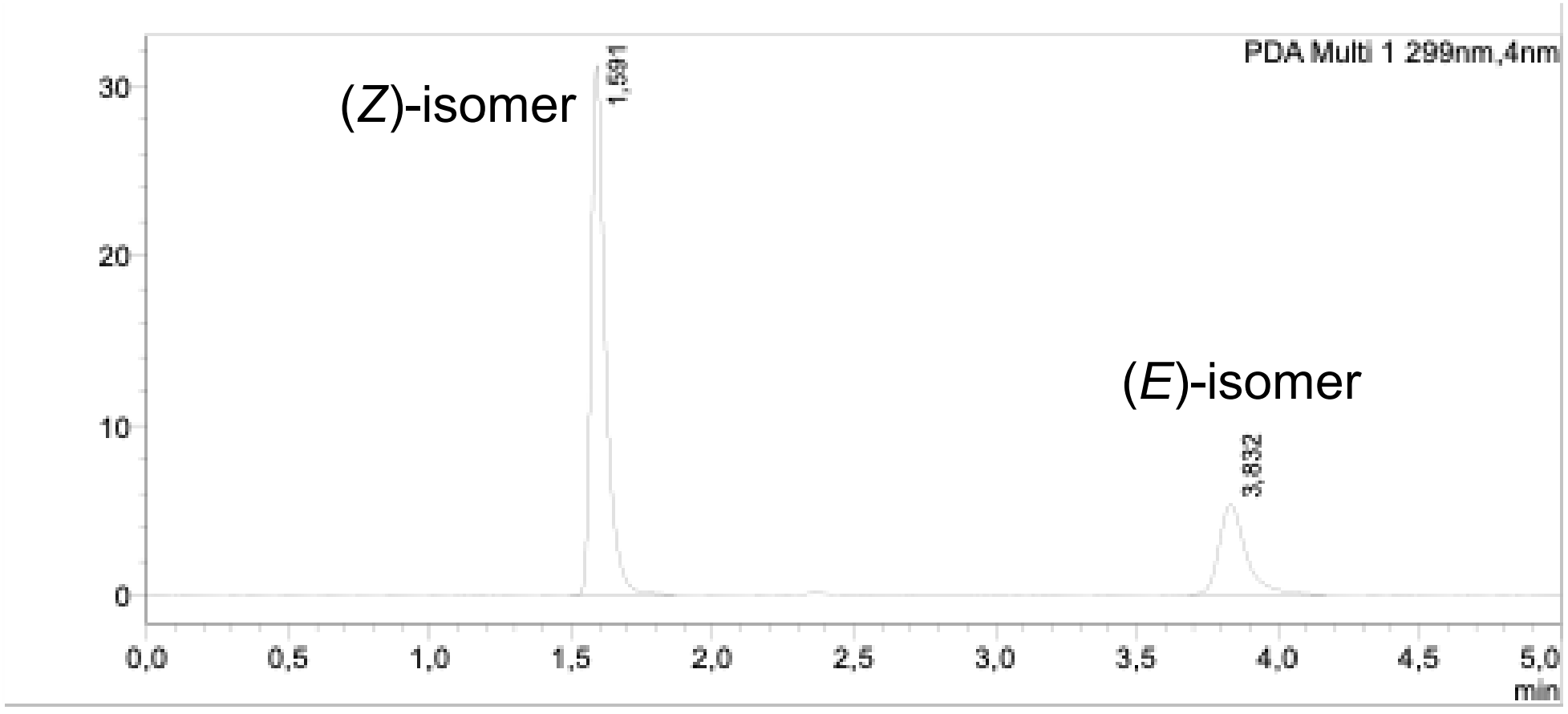

Peak Table

PDA Ch1 299nm

Peak#	Ret. Time	Area	Height	Conc.
1	1,591	109622	30596	76,364
2	3,832	33931	5211	23,636
Total		143553	35807	

b)

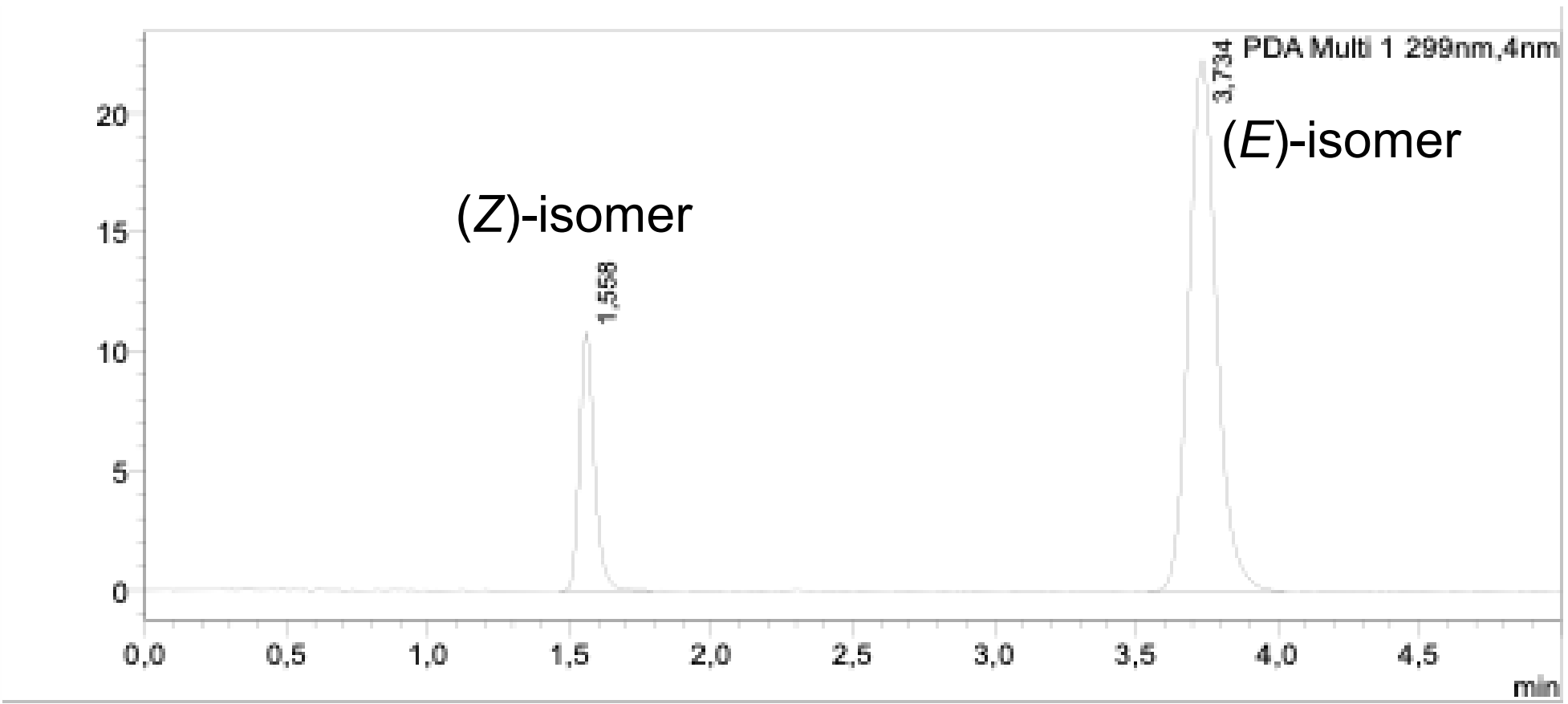

Peak Table

PDA Ch1 299nm

Peak#	Ret. Time	Area	Height	Conc.
1	1,558	38700	10605	20,385
2	3,734	151147	22117	79,615
Total		189847	32722	

Figure S6: Example of chromatograms of the PSSs of *p*-OHex-AB, detected at the isosbestic wavelength of 299 nm a) after irradiation with 340 nm for 5 min and b) after irradiation with 448 nm for 5 min.

Table S5: Threefold determination of the content of (*Z*)-isomer at PSS at 365 nm in ACN *via* HPLC.

Compound	Run 1	Run 2	Run 3	Mean	Δ
**3a**: R = H	95.1	95.0	94.6	94.9	0.2
**3b**: R = 2-Me	93.2	93.7	93.7	93.5	0.2
**3c**: R = 2-Et	95.3	95.1	94.8	95.1	0.2
**3d**: R = 2-*i*Pr	96.4	95.8	95.7	96.0	0.3
**3e**: R = 2-*t*Bu	94.5	94.3	95.3	94.7	0.4
**3f**: R = 3-Me	94.3	94.2	94.7	94.4	0.2
**3g**: R = 3-Et	96.0	96.2	95.3	95.8	0.4
**3h**: R = 3-*i*Pr	95.3	95.5	94.9	95.2	0.2
**3i**: R = 3-*t*Bu	94.4	94.4	95.3	94.7	0.4
**3j**: R = 4-Me	96.4	97.2	96.7	96.8	0.3
**3k**: R = 4-Et	96.7	96.5	96.1	96.4	0.2
**3l**: R = 4-*i*Pr	95.9	95.5	94.9	95.4	0.4
**3m**: R = 4-*t*Bu	96.8	96.0	96.3	96.4	0.3
**3n**: R = 2,6-Me	92.3	91.7	92.2	92.1	0.3
**3o**: R = 2,4,6-Me	93.9	93.2	93.5	93.5	0.3
**3p**: R = 3,5-Me	95.1	94.7	94.3	94.7	0.3
**3q**: R = 2,6-F	93.2	93.5	93.7	93.5	0.2
**3r**: R = 3-OMe	95.1	95.7	95.4	95.4	0.2

To determine the content of the (*E*)- and (*Z*)-isomer at PSS_{365} of the neat substituted *p*-OHex-AB´s, the solid compound was placed as a fine powder in a thin layer on a glass slide (see Figure S2 a) and then irradiated with a LED at 365 nm with a distance of approx. 5 cm at rt. Each sample was irradiated until they had fully converted into the liquid phase. Irradiation continued until no further changes in composition were observed.

Table S6: Determination of the highest content of (*Z*)-isomer at PSS_{365} nm of neat *p*-Hexoxy-ABs *via* HPLC.

Compound	PSS_{365}
**4a**: R = H	93.3
**4b**: R = 2-Me	91.8
**4c**: R = 2-Et	88.3
**4d**: R = 2-*i*Pr	92.8
**4e**: R = 2-*t*Bu	83.3
**4f**: R = 3-Me	93.2
**4g**: R = 3-Et	92.7
**4h**: R = 3-*i*Pr	92.8
**4i**: R = 3-*t*Bu	92.7
**4j**: R = 4-Me	94.0
**4k**: R = 4-Et	86.3
**4l**: R = 4-*i*Pro	90.0
**4m**: R = 4-*t*Bu	96.3
**4n**: R = 2,6-ME	88.9
**4o**: R = 2,4,6-Me	85.1
**4p**: R = 3,5-Me	91.5
**4q**: R = 2,6-F	91.7
**4r**: R = 3-OMe	87.3

8. DSC measurements

DSC measurements were performed on a Netzsch DSC 300 Caliris® at a heating and cooling rate of 10 °C/min under N_2 atmosphere. Approximately 10 mg of each

compound was irradiated with 365 nm. The composition of the (*E*)- and (*Z*)-isomers were determined *via* HPLC. The compounds were irradiated until no further change was observed. Subsequently, 2-3 mg of the photoliquified compound was transferred into a differential scanning calorimetry (DSC) pan. For each compound a twofold measurement was performed. The isomerization enthalpy was measured after irradiation with 365 nm of the neat compound. The content of the (*Z*)-isomer was measured *via* HPLC before each measurement. For each measurement two cooling and heating cycles were performed. In the first cycle the glass transition temperature (T_{glass}) and the isomerization enthalpy ($\Delta H_{iso.}$) and temperature ($T_{iso.}$) of the photoliquified compound were determined. In the second cycle the melting enthalpy ($\Delta H_{melt.}$) and temperature ($T_{melt.}$) and the crystallization enthalpy ($\Delta H_{cry.}$) and crystallization temperature ($T_{cry.}$) were determined. For all determined temperatures the onset temperature was used.

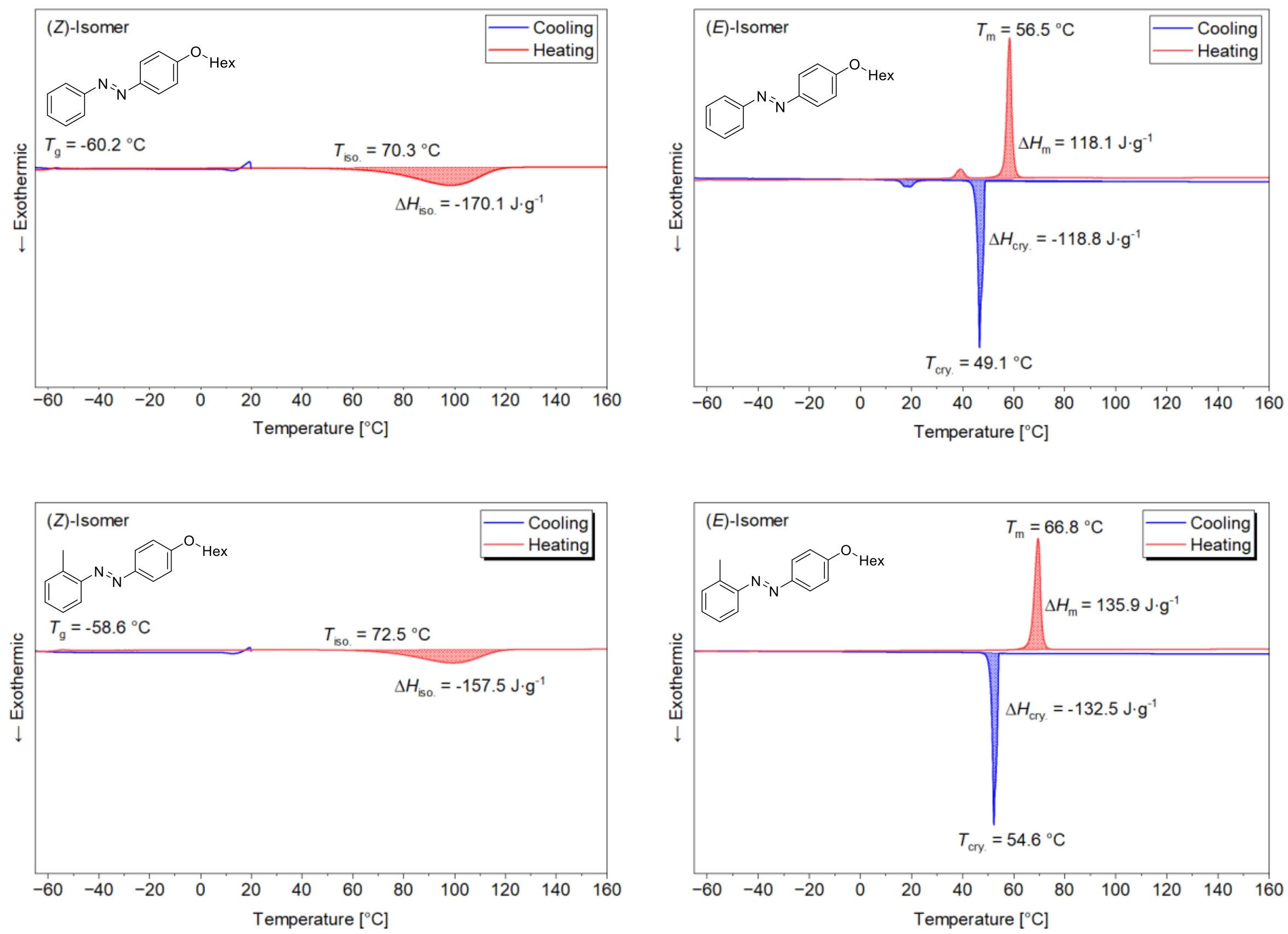

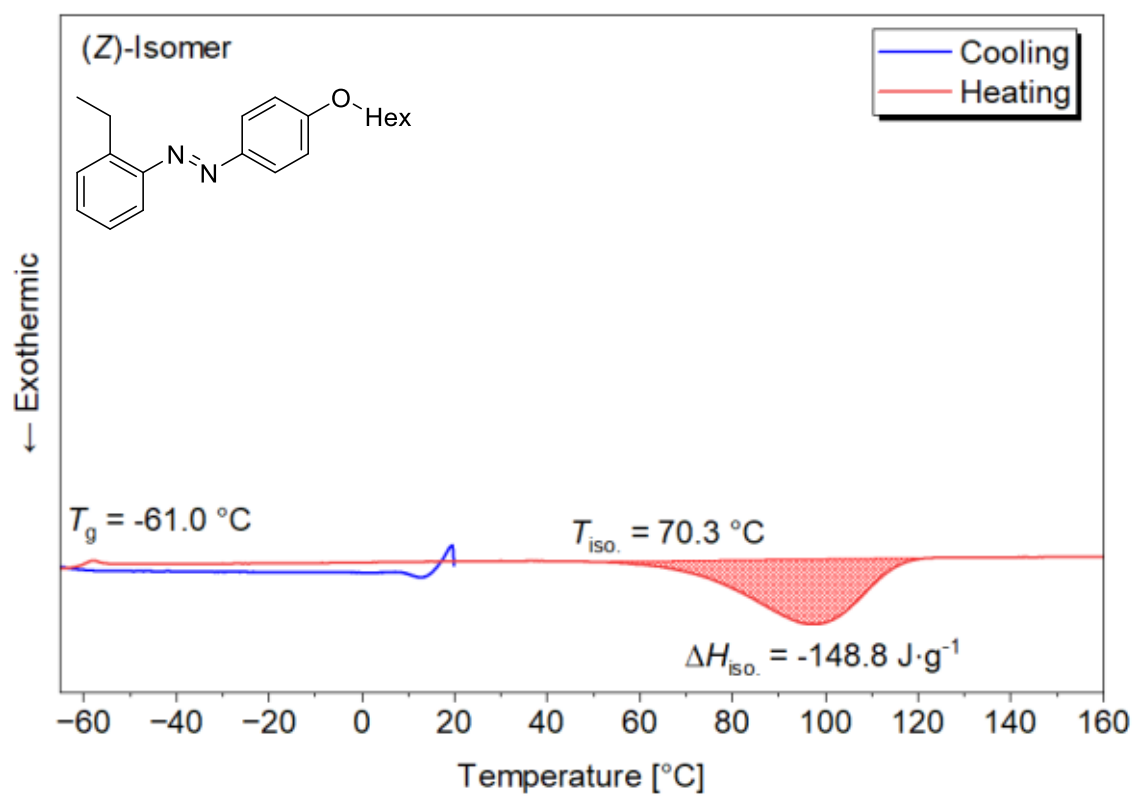
(Z)-Isomer
Hex
Cooling
Heating
Tg = -61.0 °C
Tiso. = 70.3 °C
ΔHiso. = -148.8 J·g-1
← Exothermic
Temperature [°C]

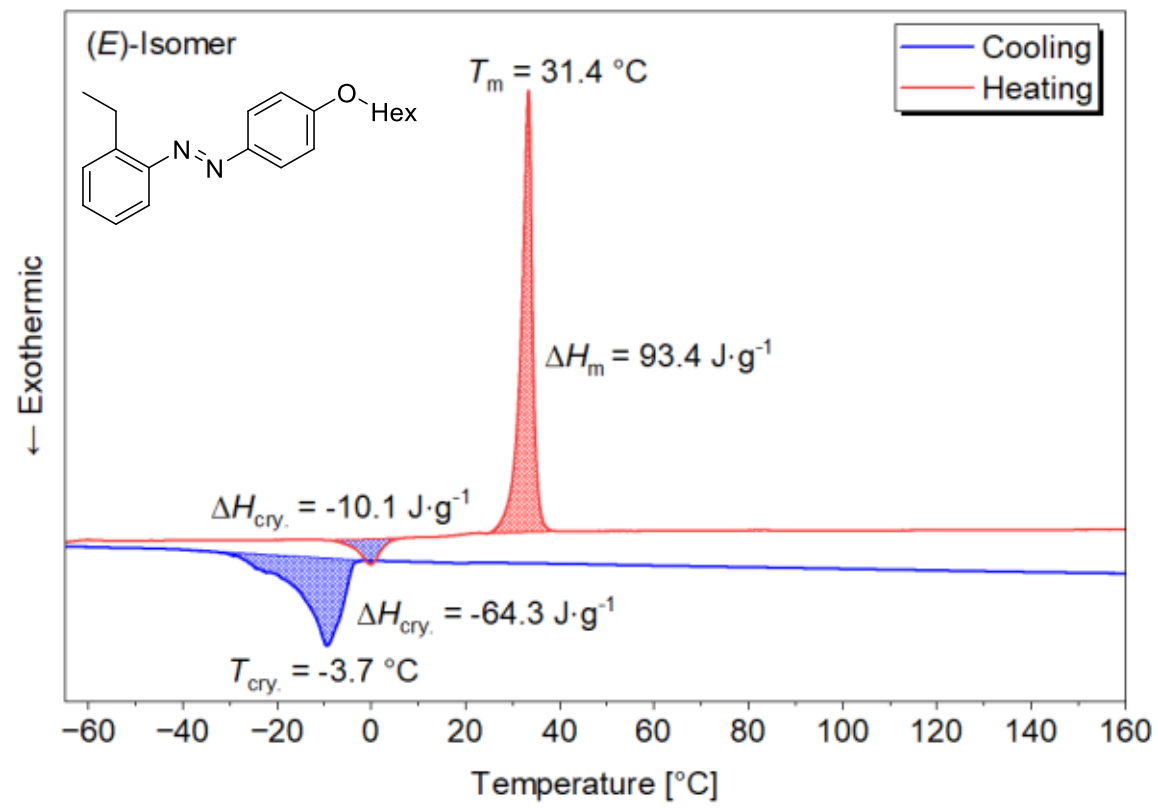
(E)-Isomer
Hex
Cooling
Heating
Tm = 31.4 °C
ΔHm = 93.4 J·g-1
ΔHcry. = -10.1 J·g-1
ΔHcry. = -64.3 J·g-1
Tcry. = -3.7 °C
← Exothermic
Temperature [°C]

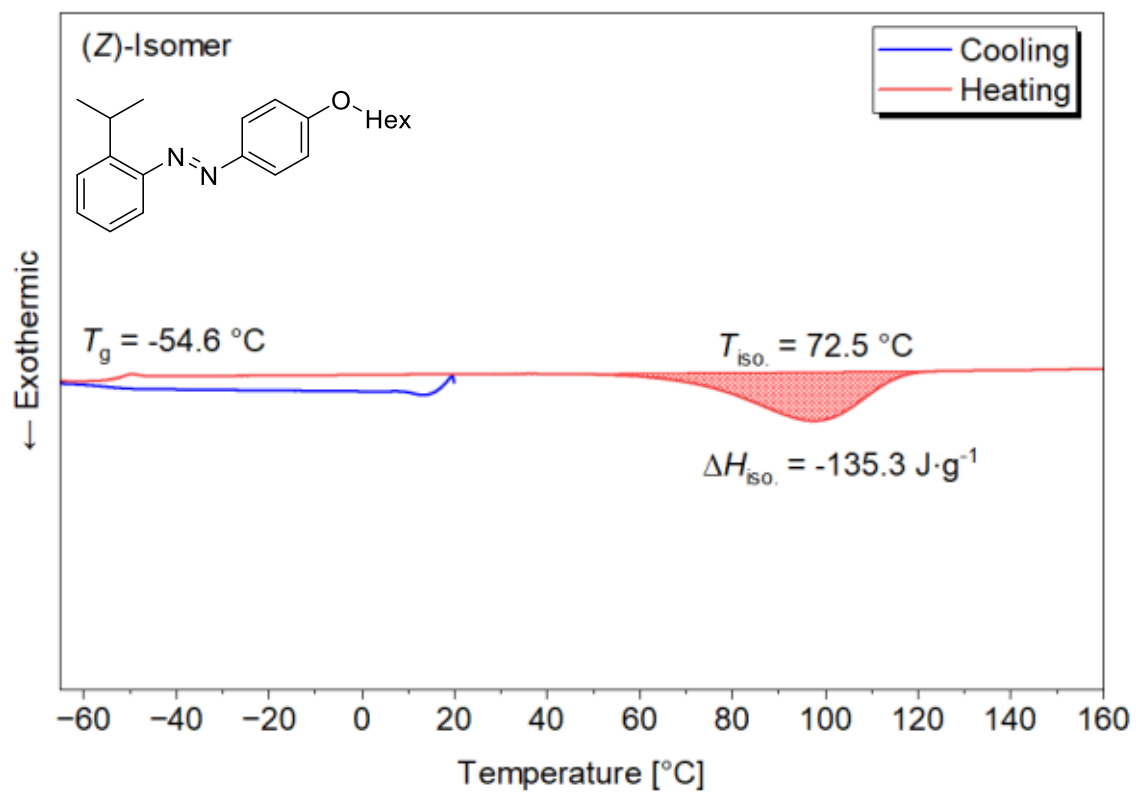
(Z)-Isomer
Hex
Cooling
Heating
Tg = -54.6 °C
Tiso. = 72.5 °C
ΔHiso. = -135.3 J·g-1
← Exothermic
Temperature [°C]

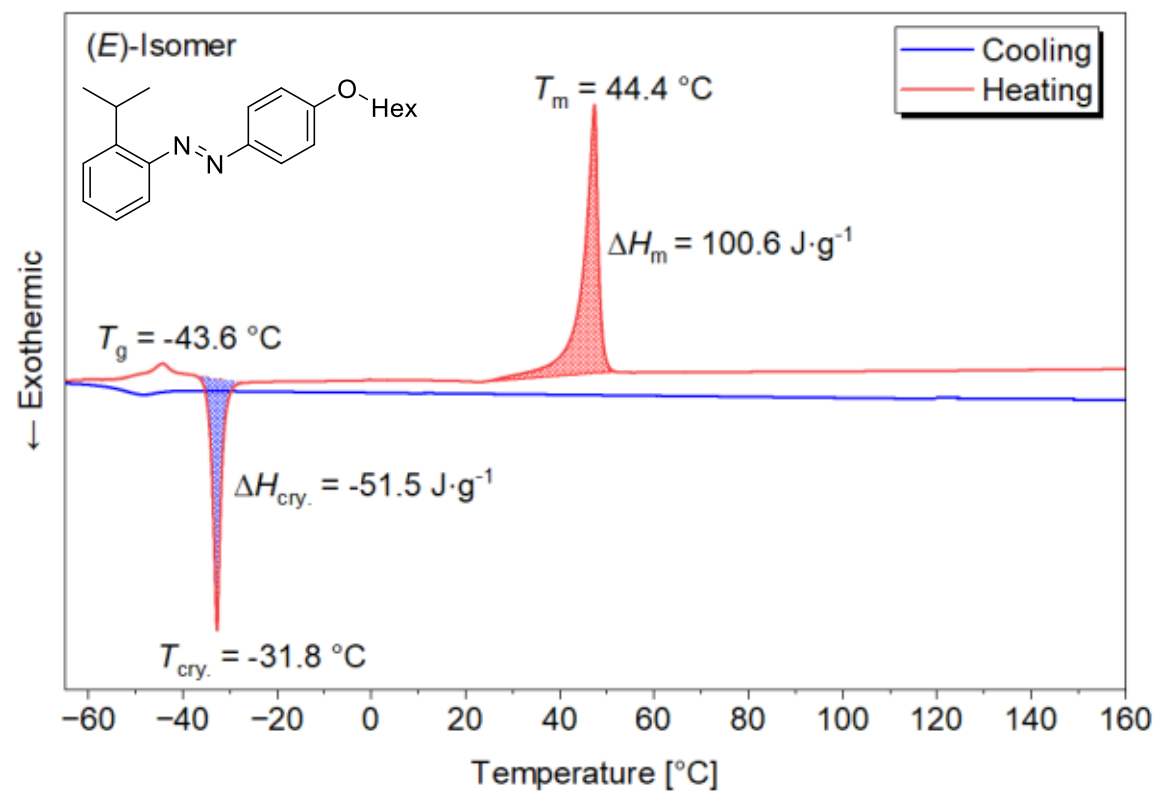
(E)-Isomer
Hex
Cooling
Heating
Tm = 44.4 °C
ΔHm = 100.6 J·g-1
Tg = -43.6 °C
ΔHcry. = -51.5 J·g-1
Tcry. = -31.8 °C
← Exothermic
Temperature [°C]

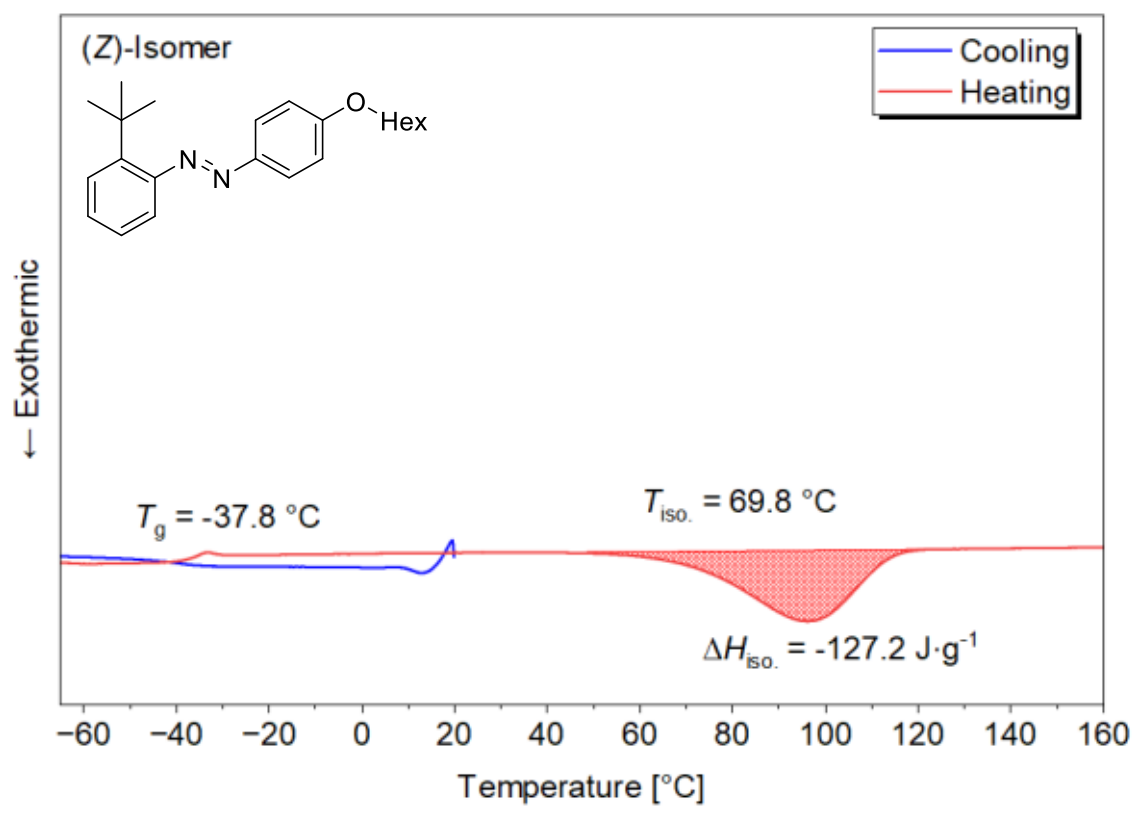
(Z)-Isomer
Hex
Cooling
Heating
Tg = -37.8 °C
Tiso. = 69.8 °C
ΔHiso. = -127.2 J·g-1
← Exothermic
Temperature [°C]

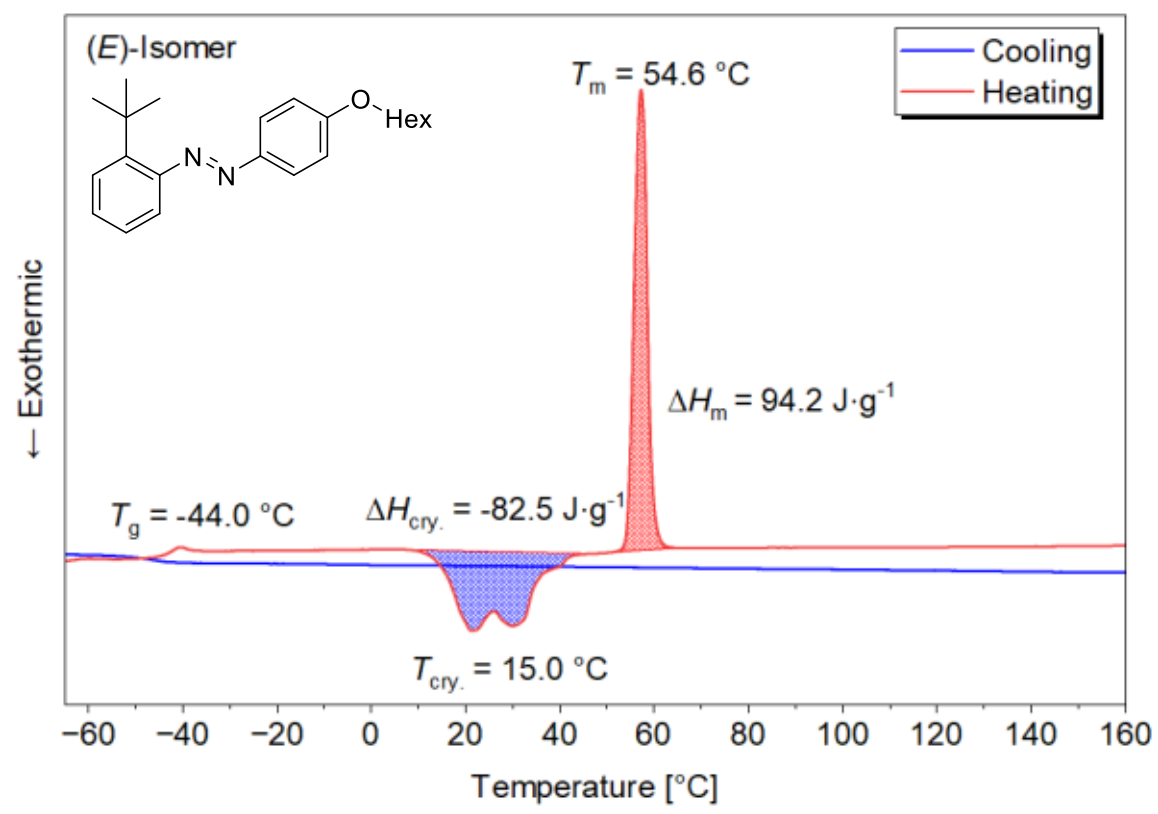
(E)-Isomer
Hex
Cooling
Heating
Tm = 54.6 °C
ΔHm = 94.2 J·g-1
Tg = -44.0 °C
ΔHcry. = -82.5 J·g-1
Tcry. = 15.0 °C
← Exothermic
Temperature [°C]

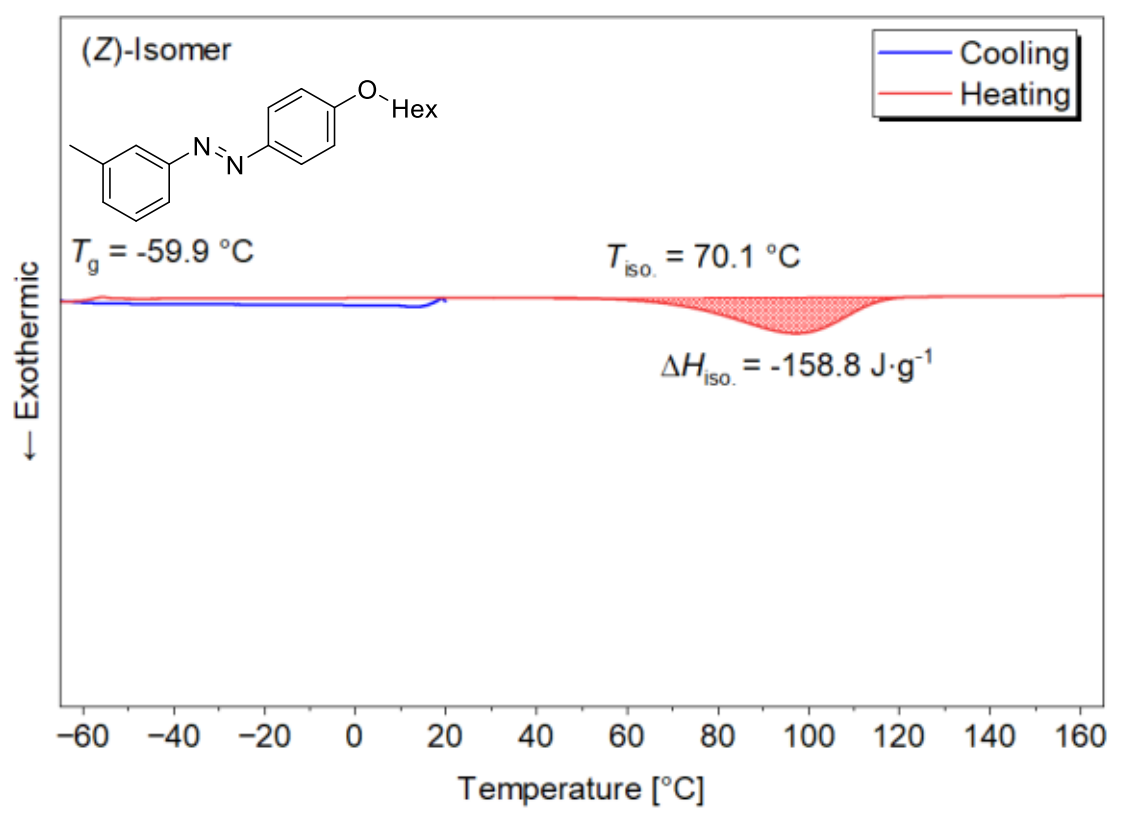
(Z)-Isomer
Hex
Cooling
Heating
Tg = -59.9 °C
Tiso. = 70.1 °C
ΔHiso. = -158.8 J·g-1
← Exothermic
Temperature [°C]

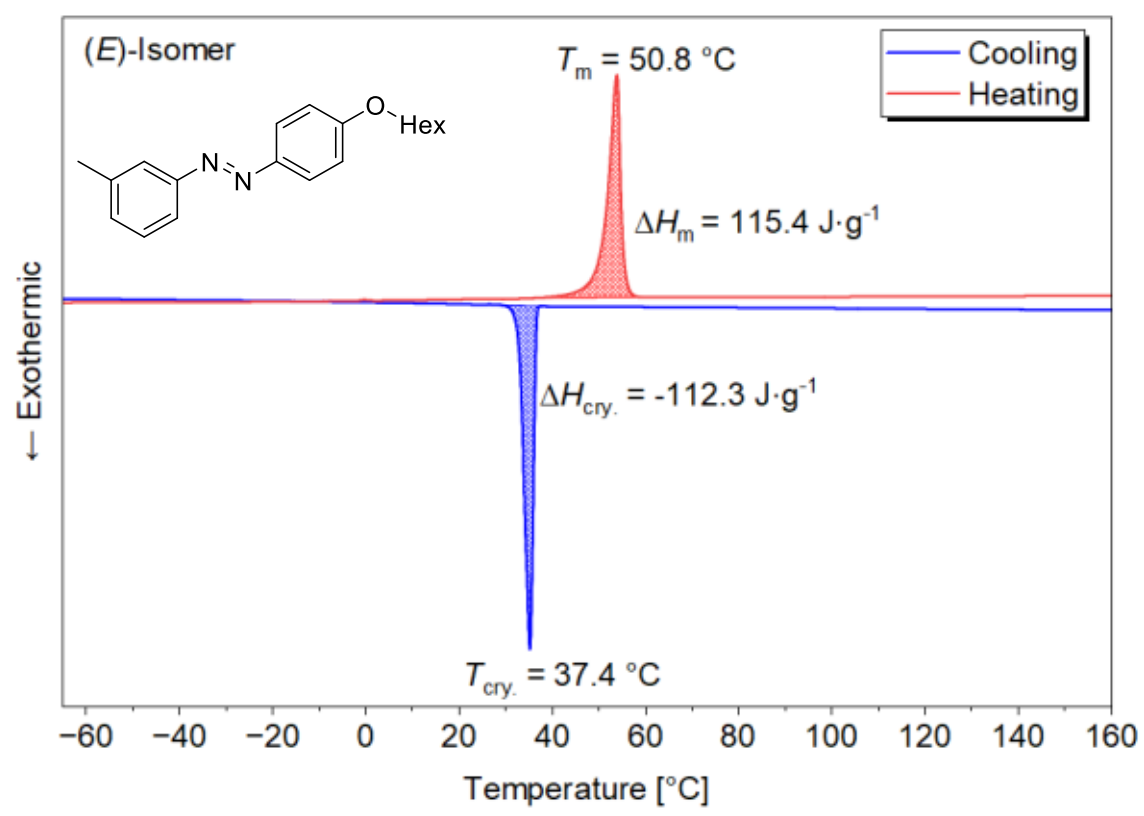
(E)-Isomer
Hex
Cooling
Heating
Tm = 50.8 °C
ΔHm = 115.4 J·g-1
ΔHcry. = -112.3 J·g-1
Tcry. = 37.4 °C
← Exothermic
Temperature [°C]

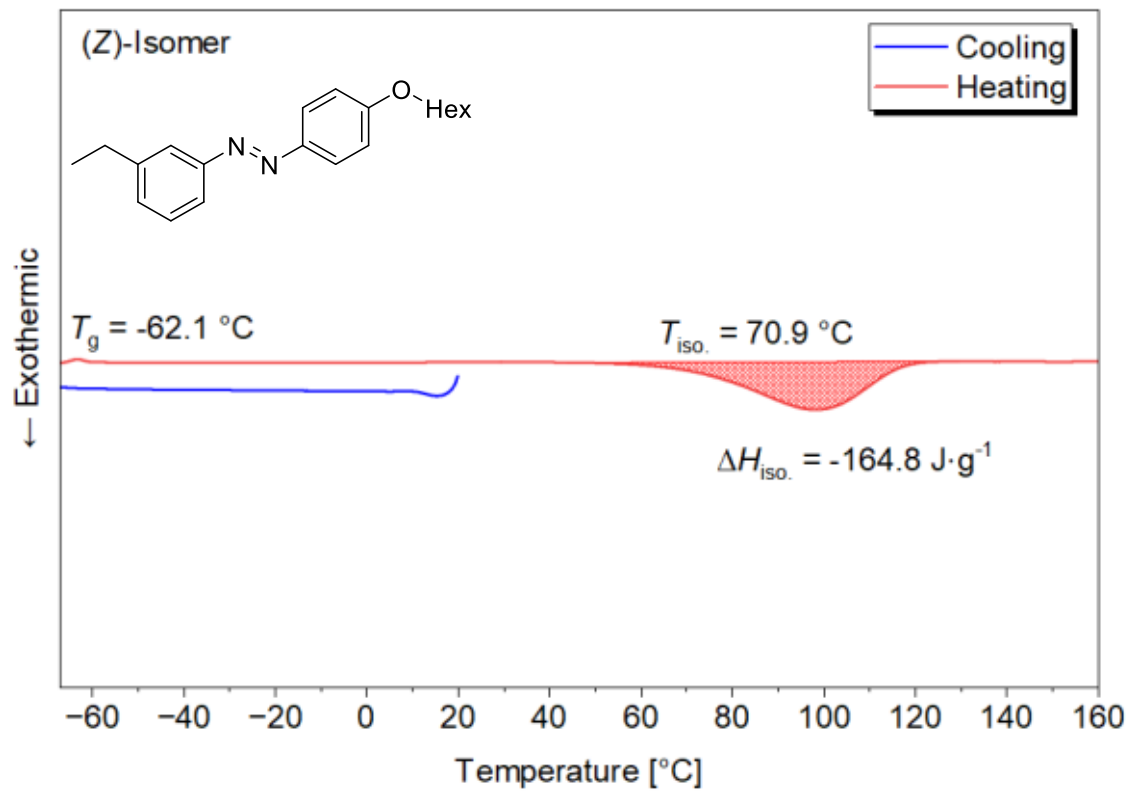
(Z)-Isomer
Cooling
Heating
O
Hex
N
N
← Exothermic
Tg = -62.1 °C
Tiso. = 70.9 °C
ΔHiso. = -164.8 J·g-1
−60 −40 −20 0 20 40 60 80 100 120 140 160
Temperature [°C]

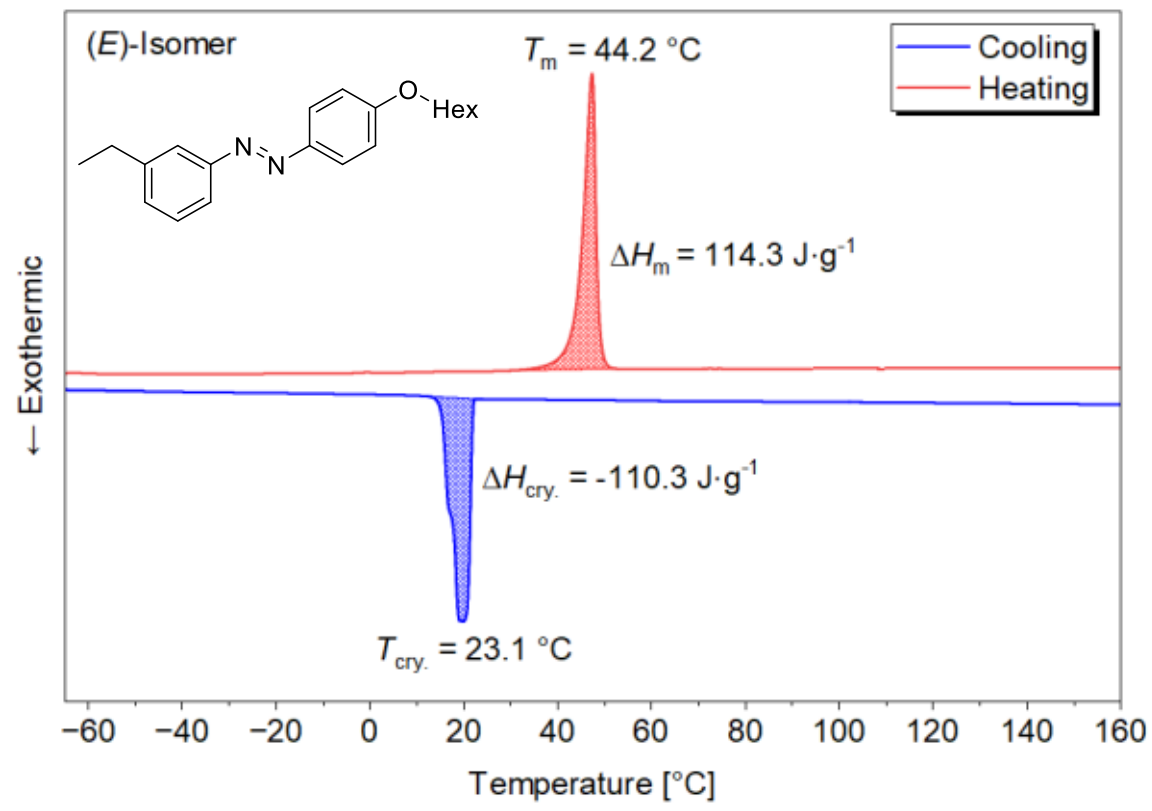
(E)-Isomer
Cooling
Heating
Tm = 44.2 °C
O
Hex
N
N
ΔHm = 114.3 J·g-1
← Exothermic
ΔHcry. = -110.3 J·g-1
Tcry. = 23.1 °C
−60 −40 −20 0 20 40 60 80 100 120 140 160
Temperature [°C]

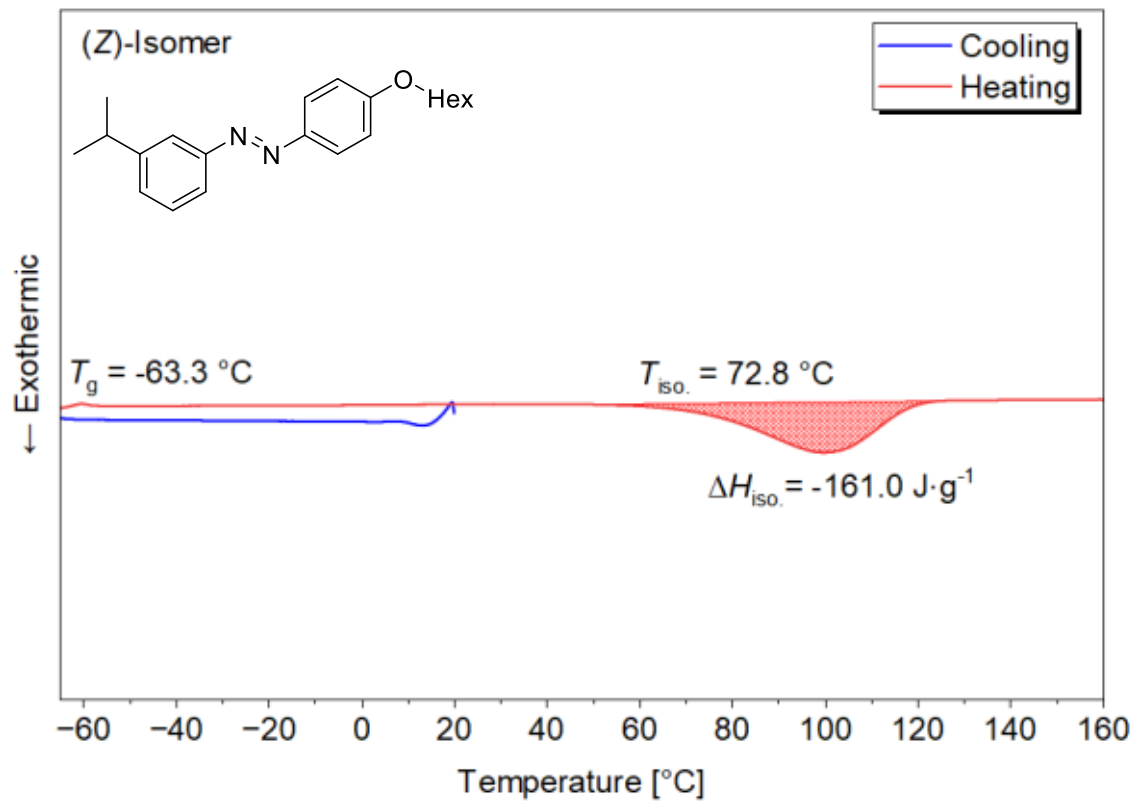
(Z)-Isomer
Cooling
Heating
O
Hex
N
N
← Exothermic
Tg = -63.3 °C
Tiso. = 72.8 °C
ΔHiso. = -161.0 J·g-1
−60 −40 −20 0 20 40 60 80 100 120 140 160
Temperature [°C]

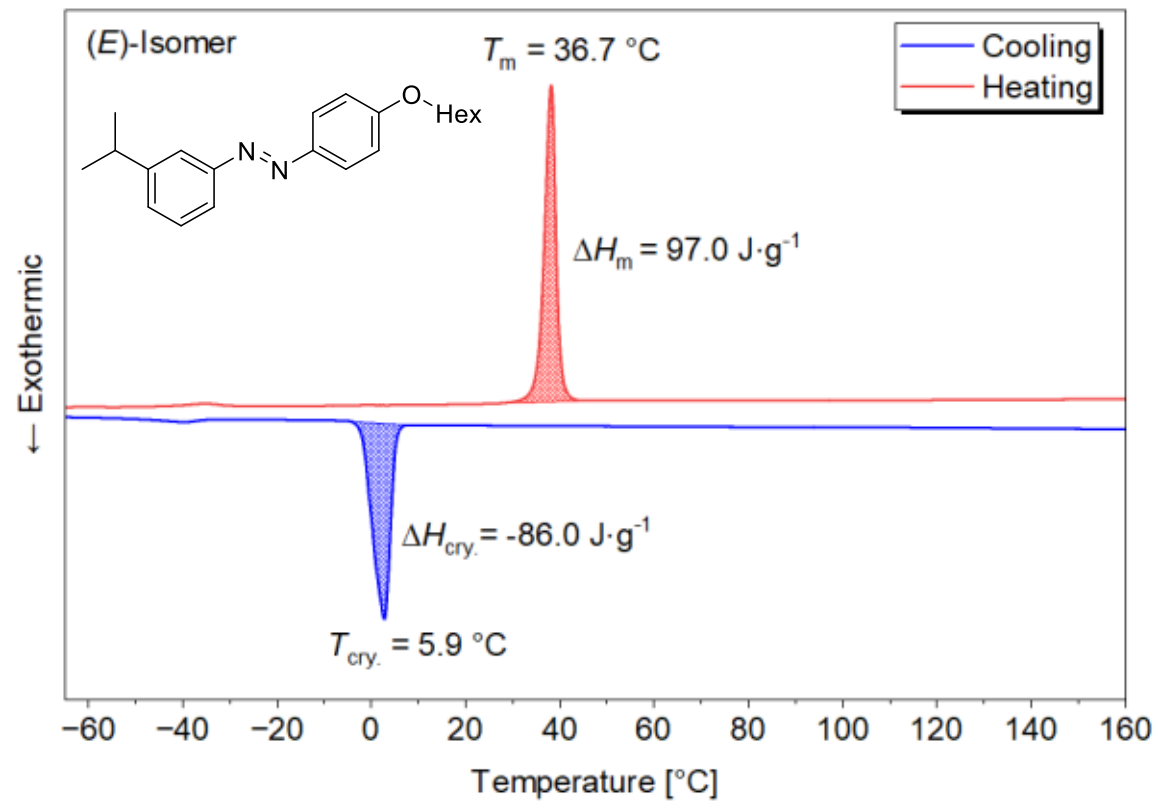
(E)-Isomer
Cooling
Heating
Tm = 36.7 °C
O
Hex
N
N
ΔHm = 97.0 J·g-1
← Exothermic
ΔHcry. = -86.0 J·g-1
Tcry. = 5.9 °C
−60 −40 −20 0 20 40 60 80 100 120 140 160
Temperature [°C]

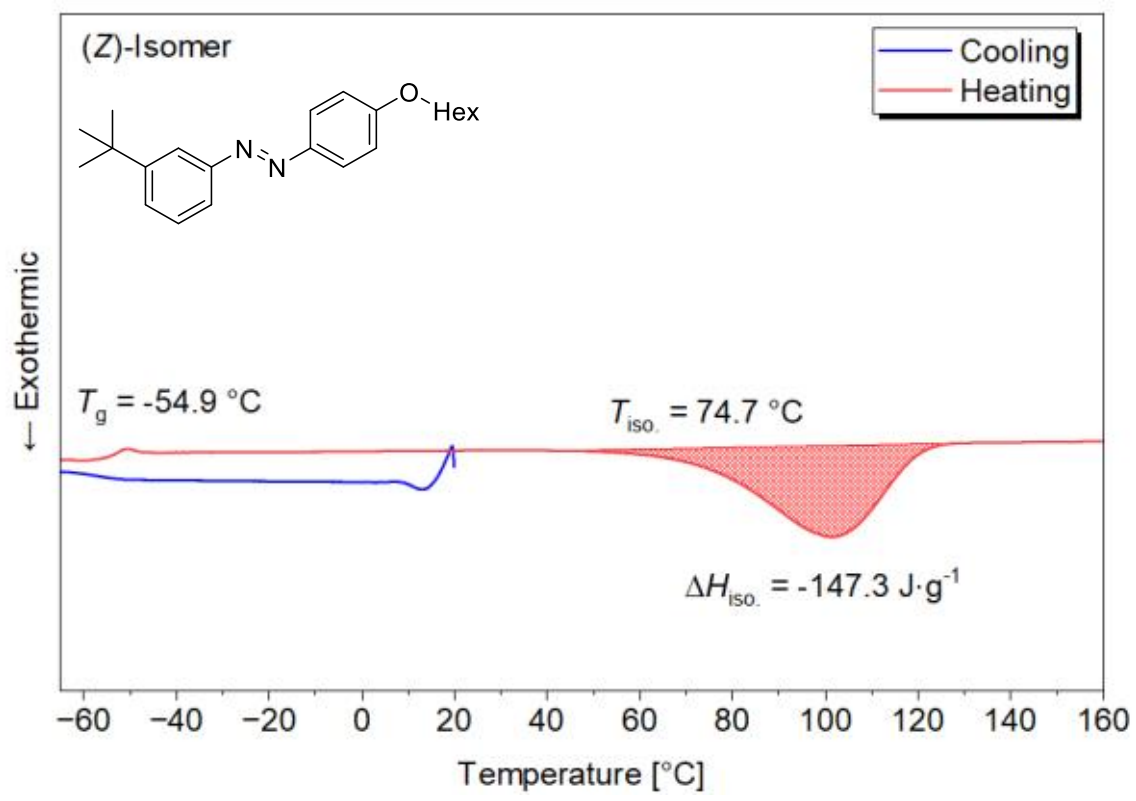
(Z)-Isomer
Cooling
Heating
O
Hex
N
N
← Exothermic
Tg = -54.9 °C
Tiso. = 74.7 °C
ΔHiso. = -147.3 J·g-1
−60 −40 −20 0 20 40 60 80 100 120 140 160
Temperature [°C]

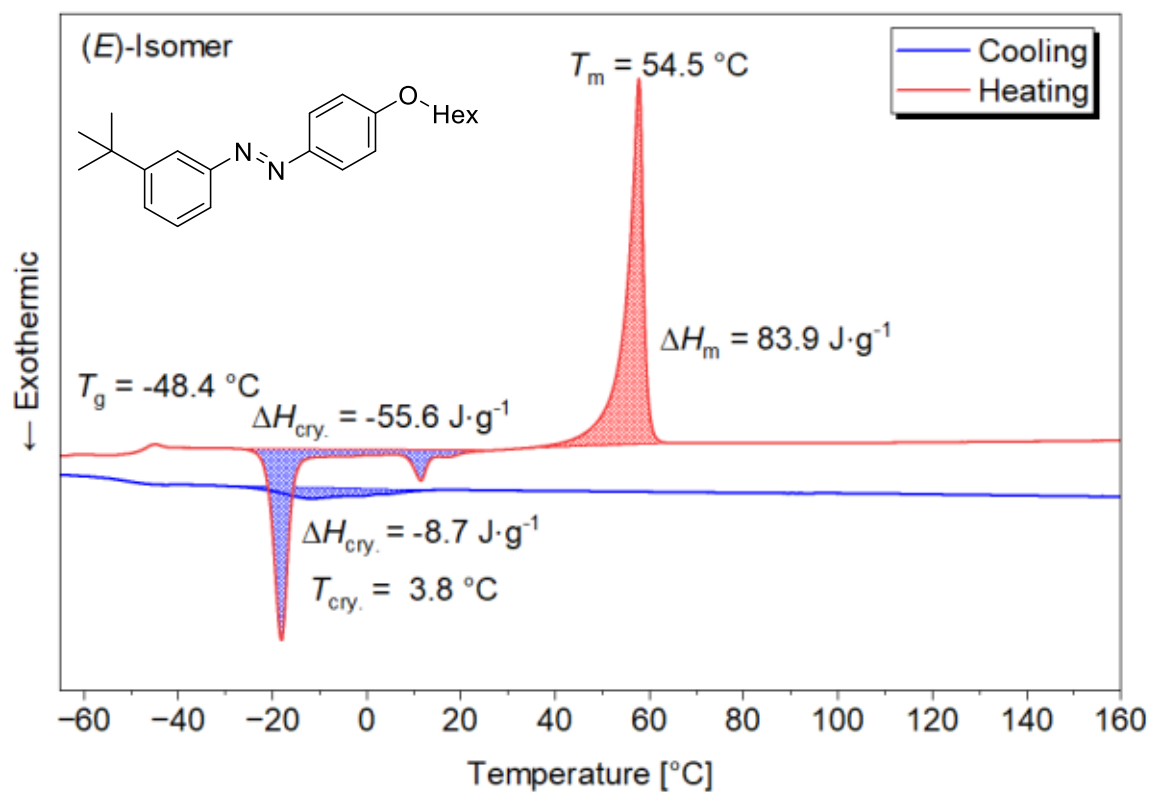
(E)-Isomer
Cooling
Heating
Tm = 54.5 °C
O
Hex
N
N
ΔHm = 83.9 J·g-1
← Exothermic
Tg = -48.4 °C
ΔHcry. = -55.6 J·g-1
ΔHcry. = -8.7 J·g-1
Tcry. = 3.8 °C
−60 −40 −20 0 20 40 60 80 100 120 140 160
Temperature [°C]

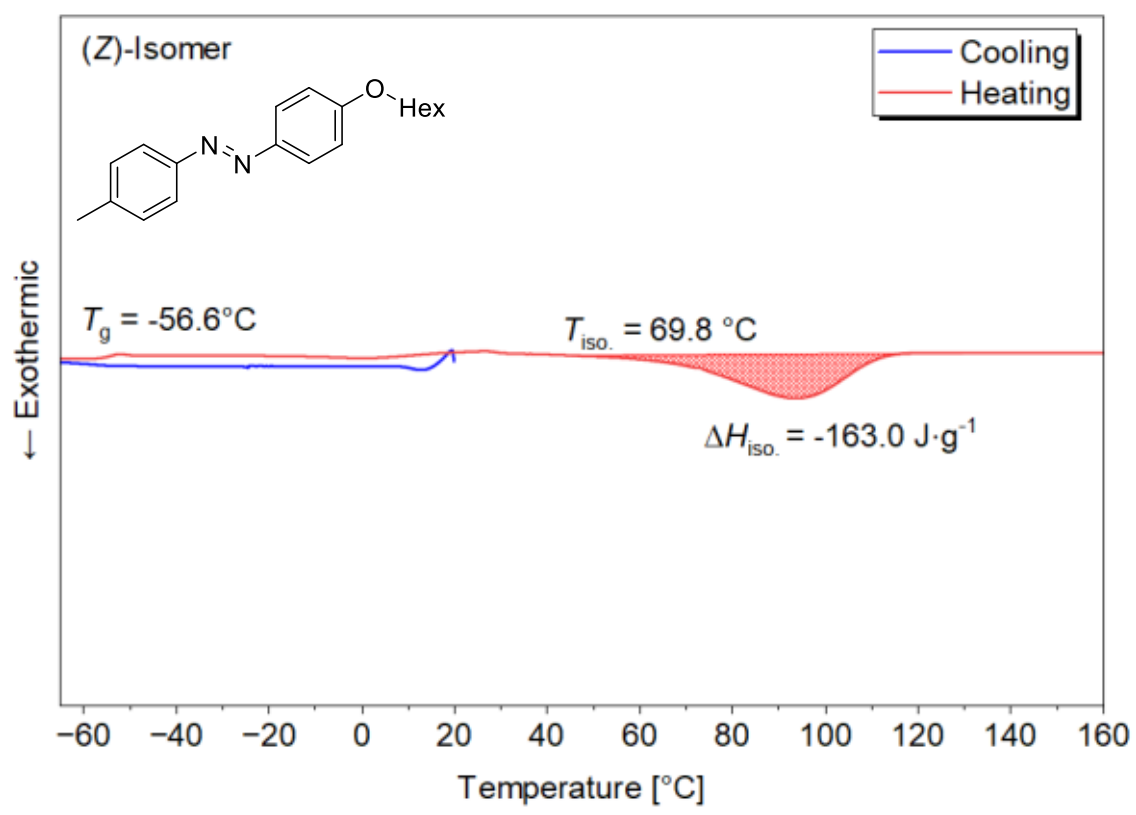
(Z)-Isomer
Cooling
Heating
O
Hex
N
N
← Exothermic
Tg = -56.6°C
Tiso. = 69.8 °C
ΔHiso. = -163.0 J·g-1
−60 −40 −20 0 20 40 60 80 100 120 140 160
Temperature [°C]

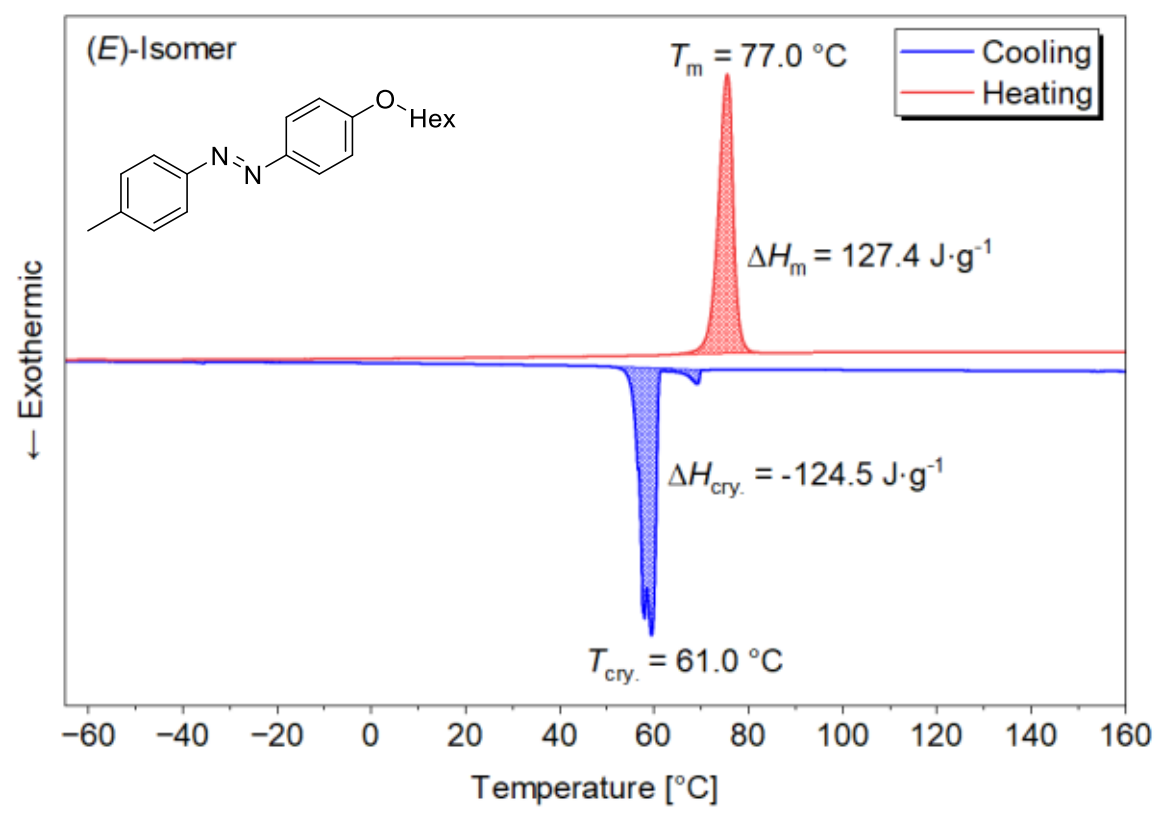
(E)-Isomer
Cooling
Heating
Tm = 77.0 °C
O
Hex
N
N
ΔHm = 127.4 J·g-1
← Exothermic
ΔHcry. = -124.5 J·g-1
Tcry. = 61.0 °C
−60 −40 −20 0 20 40 60 80 100 120 140 160
Temperature [°C]

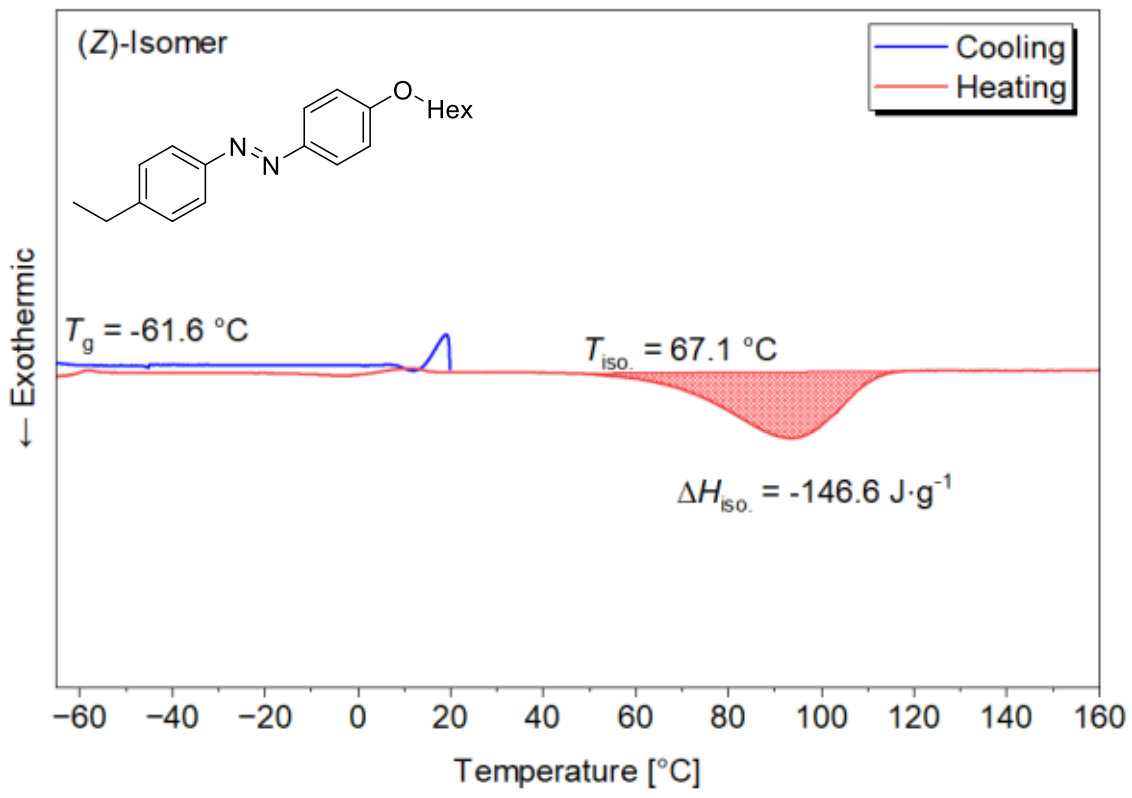
(Z)-Isomer
Cooling
Heating
Hex
T_g = -61.6 °C
$T_{iso.}$ = 67.1 °C
$\Delta H_{iso.}$ = -146.6 J·g^{-1}
← Exothermic
Temperature [°C]

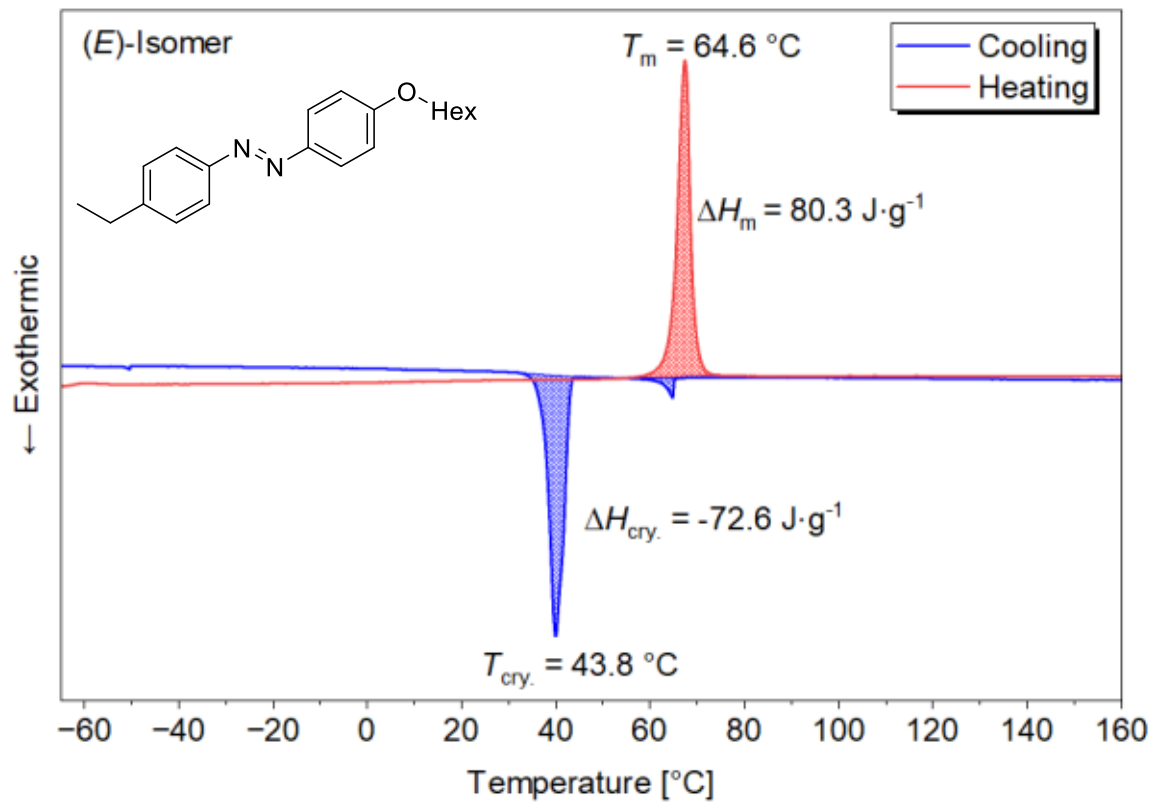
(E)-Isomer
Cooling
Heating
Hex
T_m = 64.6 °C
ΔH_m = 80.3 J·g^{-1}
$\Delta H_{cry.}$ = -72.6 J·g^{-1}
$T_{cry.}$ = 43.8 °C
← Exothermic
Temperature [°C]

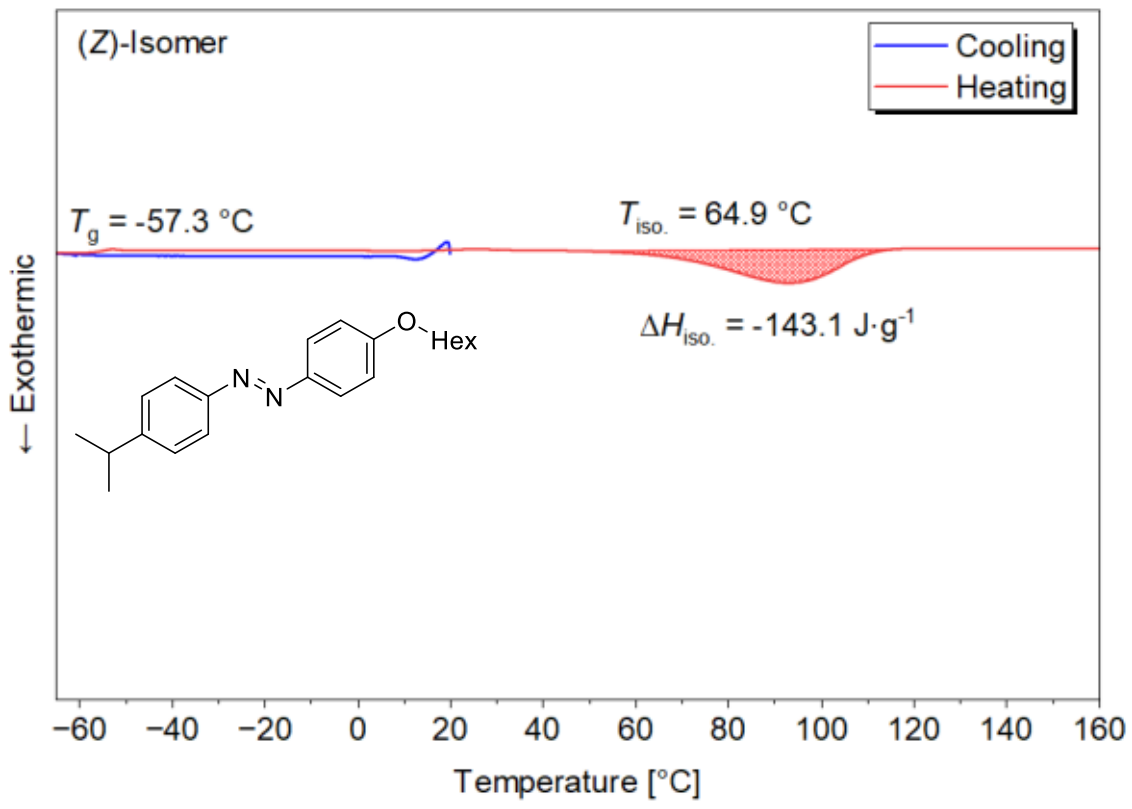
(Z)-Isomer
Cooling
Heating
T_g = -57.3 °C
$T_{iso.}$ = 64.9 °C
$\Delta H_{iso.}$ = -143.1 J·g^{-1}
Hex
← Exothermic
Temperature [°C]

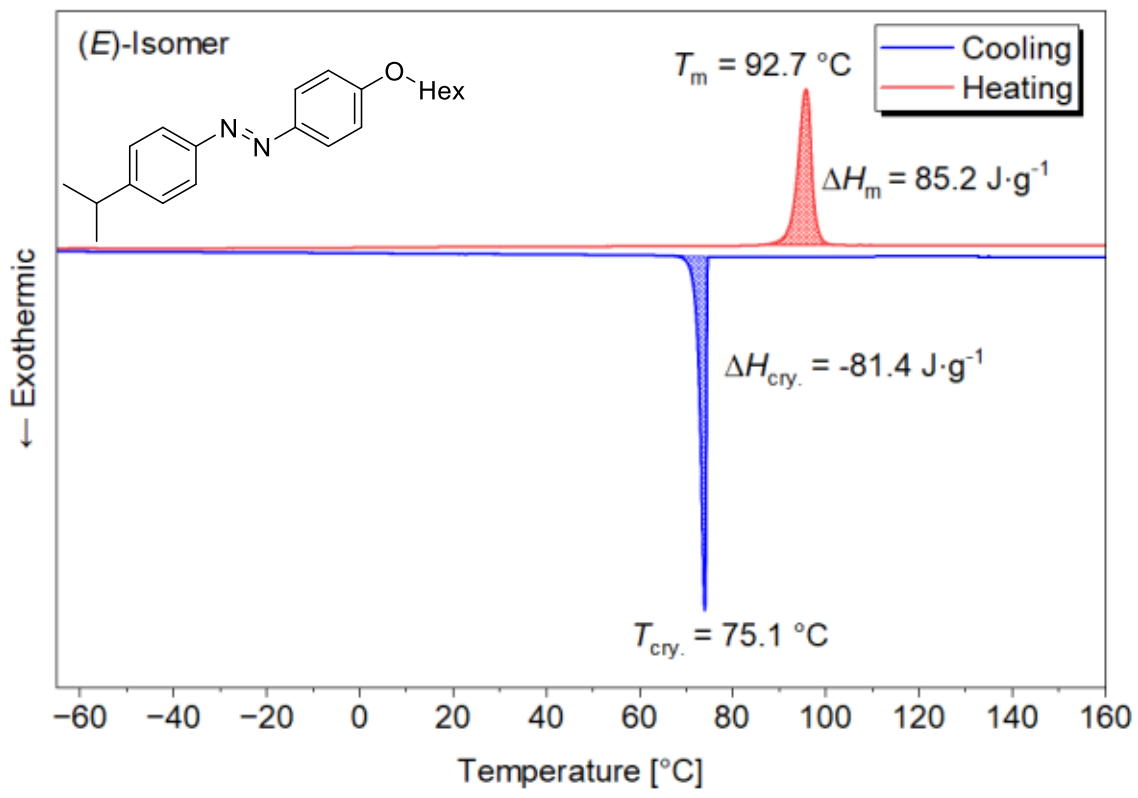
(E)-Isomer
Cooling
Heating
Hex
T_m = 92.7 °C
ΔH_m = 85.2 J·g^{-1}
$\Delta H_{cry.}$ = -81.4 J·g^{-1}
$T_{cry.}$ = 75.1 °C
← Exothermic
Temperature [°C]

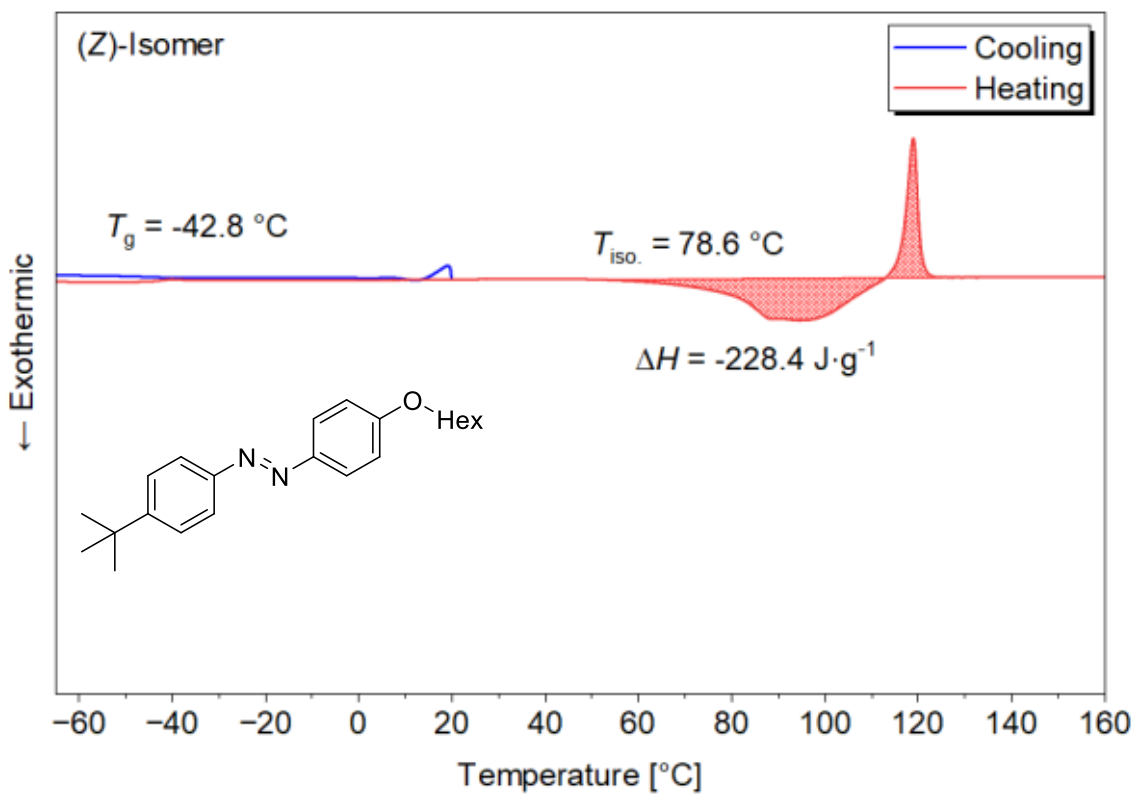
(Z)-Isomer
Cooling
Heating
T_g = -42.8 °C
$T_{iso.}$ = 78.6 °C
ΔH = -228.4 J·g^{-1}
Hex
← Exothermic
Temperature [°C]

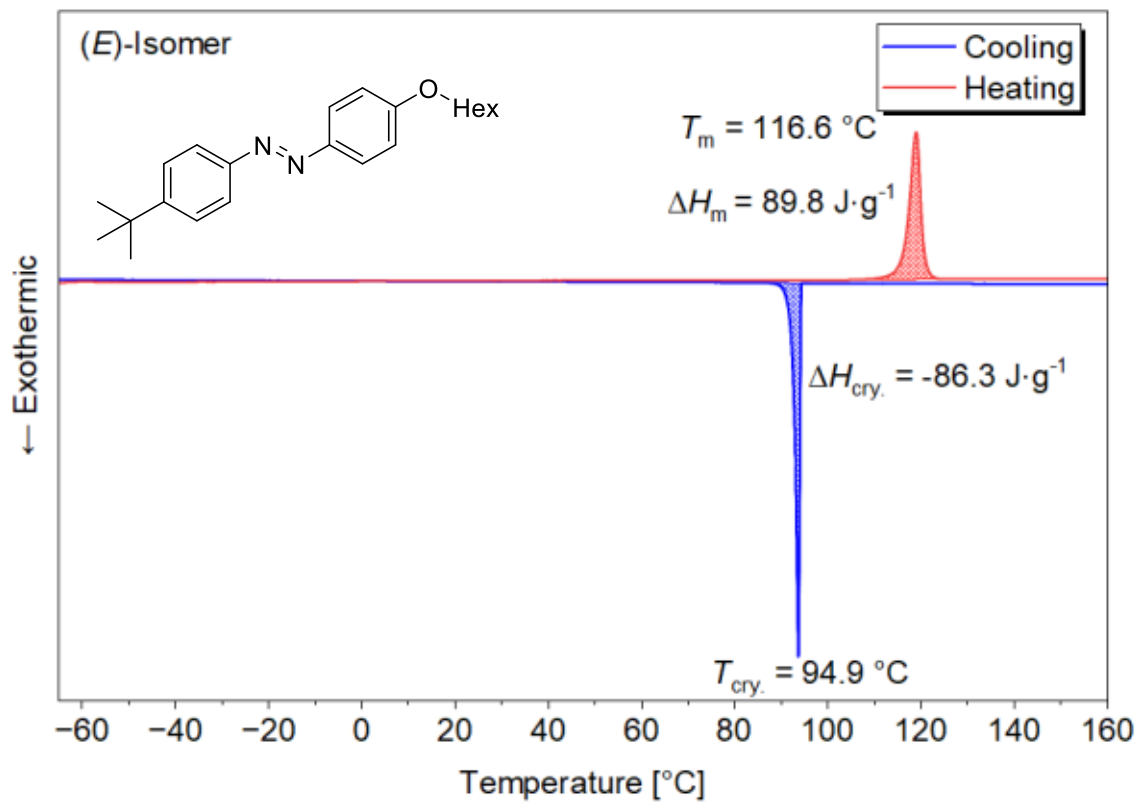
(E)-Isomer
Cooling
Heating
Hex
T_m = 116.6 °C
ΔH_m = 89.8 J·g^{-1}
$\Delta H_{cry.}$ = -86.3 J·g^{-1}
$T_{cry.}$ = 94.9 °C
← Exothermic
Temperature [°C]

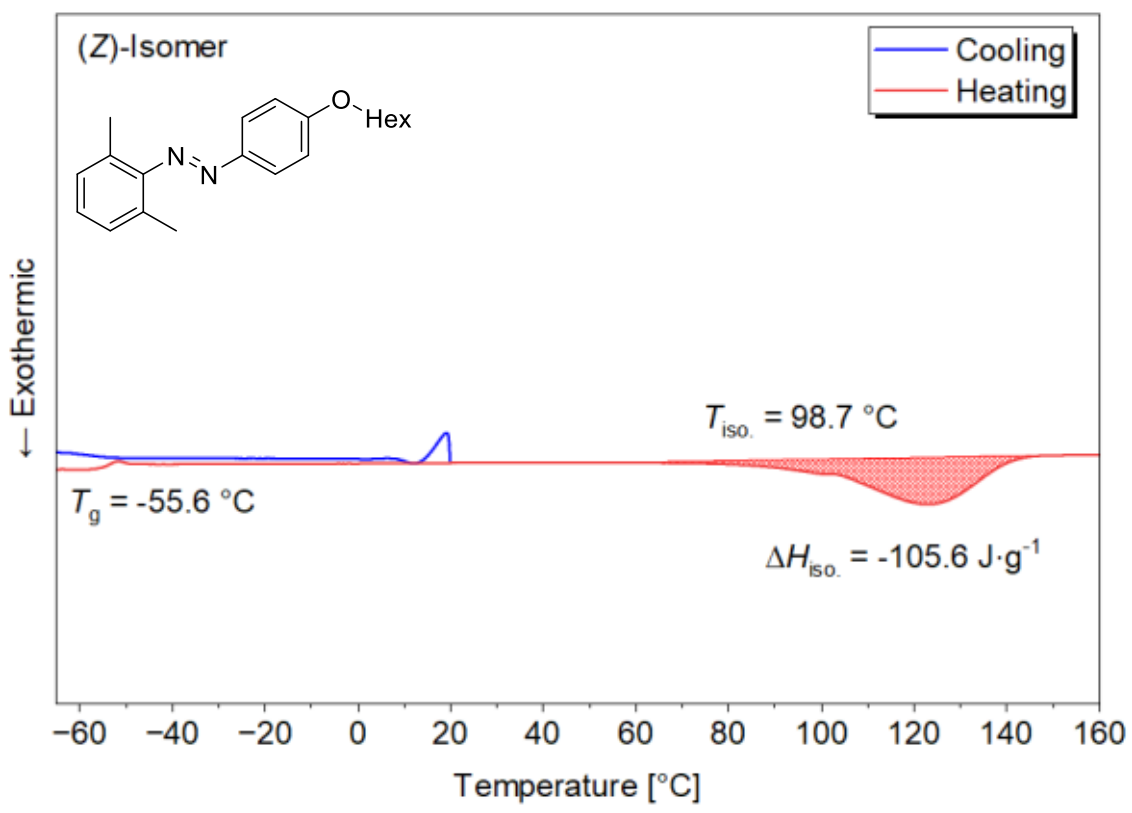
(Z)-Isomer
Cooling
Heating
Hex
$T_{iso.}$ = 98.7 °C
T_g = -55.6 °C
$\Delta H_{iso.}$ = -105.6 J·g^{-1}
← Exothermic
Temperature [°C]

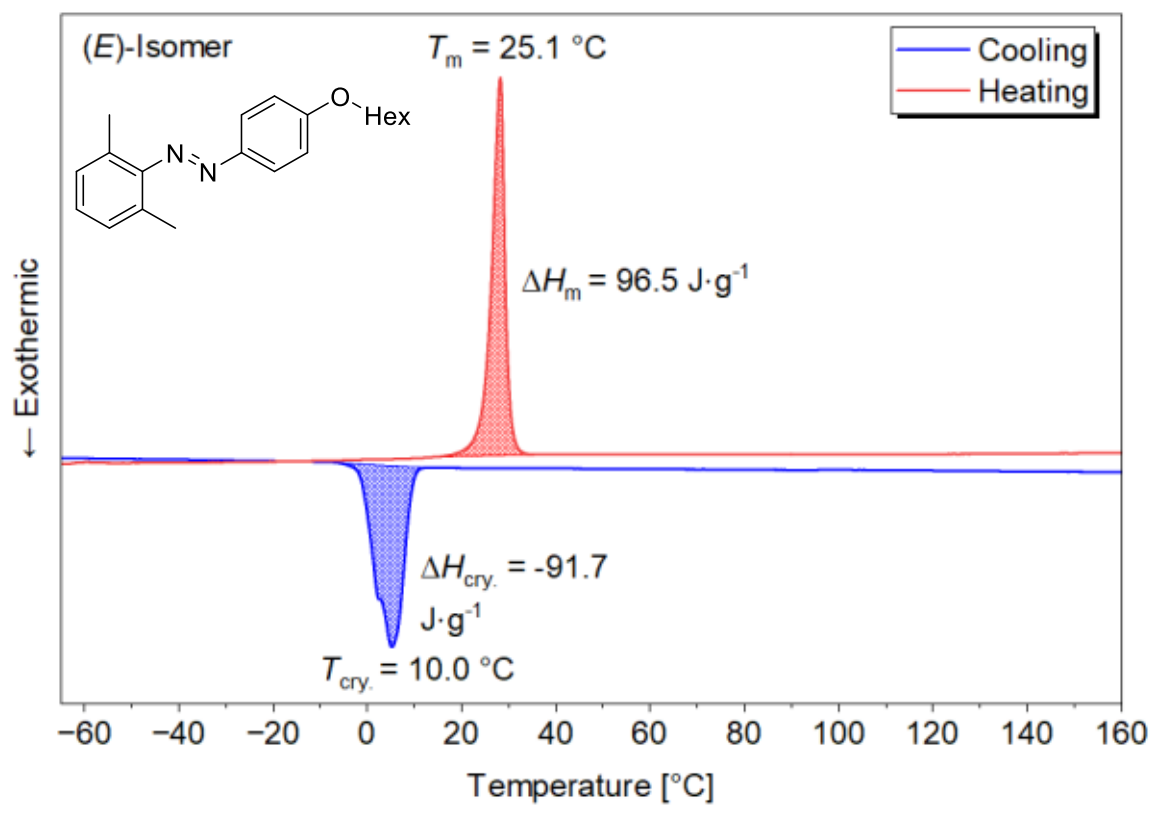
(E)-Isomer
T_m = 25.1 °C
Cooling
Heating
Hex
ΔH_m = 96.5 J·g^{-1}
$\Delta H_{cry.}$ = -91.7 J·g^{-1}
$T_{cry.}$ = 10.0 °C
← Exothermic
Temperature [°C]

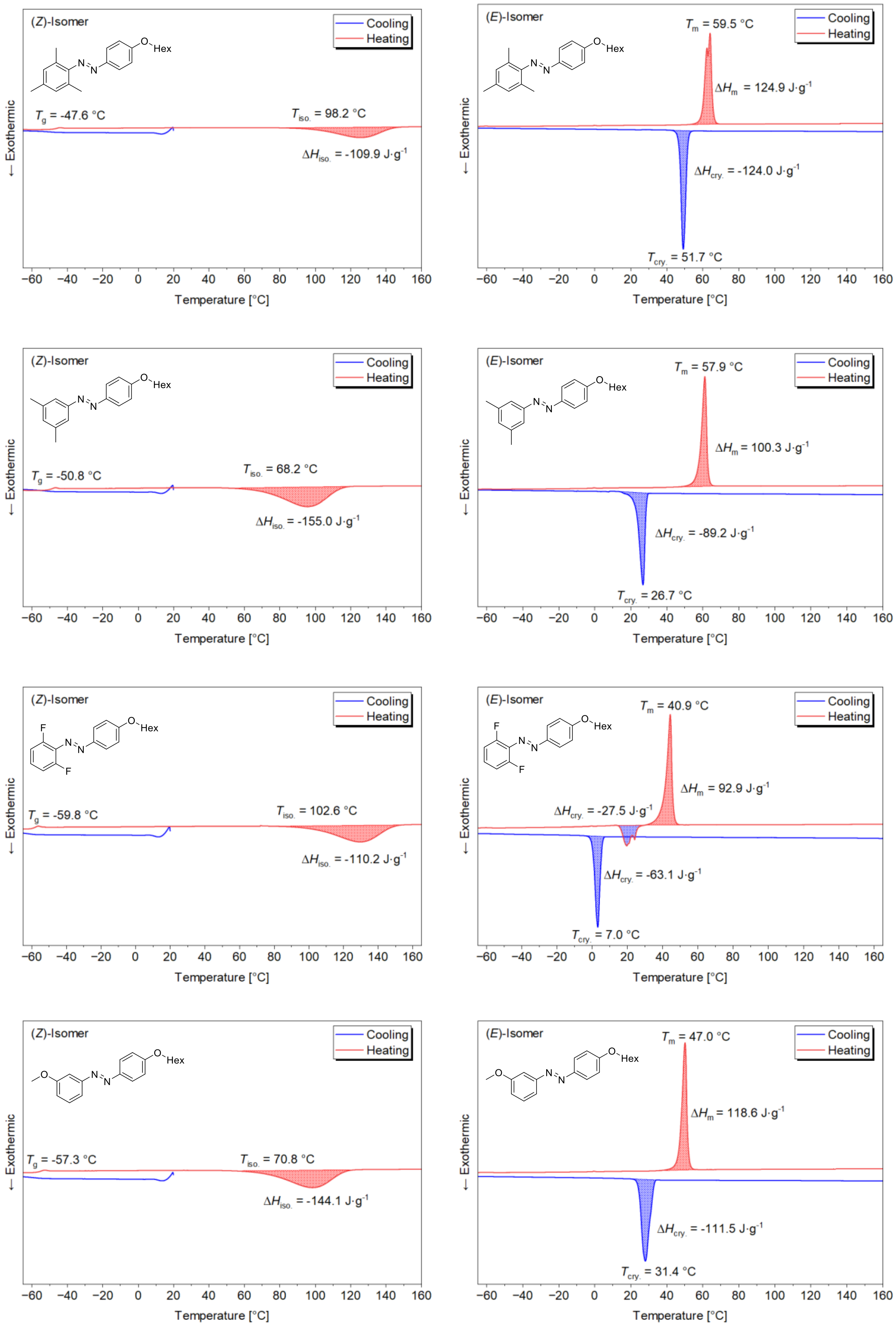

Figure S7: DSC thermograms of photoliquified and discharged *p*-OHex-AB 4a-r with heating and cooling rate of 10 k·min^{-1}.

Table S8: Twofold measurement of the glass transition temperature (T_g) of the (*Z*)-Isomer.

Compound	1. $T_{glass.}$ [°C]	2. T_g [°C]	⌀ T_g [°C]	Δ T_g [°C]
**4a**: R = H	-60.0	-60.4	-60.2	0.2
**4b**: R = 2-Me	-58.9	-58.3	-58.6	0.3
**4c**: R = 2-Et	-60.9	-61.1	-61.0	0.1
**4d**: R = 2-*i*Pr	-55.1	-54.0	-54.6	0.6
**4e**: R = 2-*t*Bu	-37.7	-37.9	-37.8	0.1
**4f**: R = 3-Me	-59.7	-60.0	-59.9	0.1
**4g**: R = 3-Et	-62.2	-62.0	-62.1	0.1
**4h**: R = 3-*i*Pr	-63.3	-63.2	-63.3	0.0
**4i**: R = 3-*t*Bu	-55.0	-54.7	-54.9	0.1
**4j**: R = 4-Me	-56.3	-56.8	-56.6	0.3
**4k**: R = 4-Et	-61.4	-61.7	-61.6	0.2
**4l**: R = 4-*i*Pr	-57.2	-57.4	-57.3	0.1
**4m**: R = 4-*t*Bu	-42.6	-42.9	-42.8	0.1
**4n**: R = 2,6-Me	-55.9	-55.3	-55.6	0.3
**4o**: R = 2,4,6-Me	-47.6	-47.6	-47.6	0.0
**4p**: R = 3,5-Me	-50.8	-50.7	-50.8	0.0
**4q**: R = 2,6-F	-59.8	-59.8	-59.8	0.0
**4r**: R = 3-OMe	-57.2	-57.4	-57.3	0.1

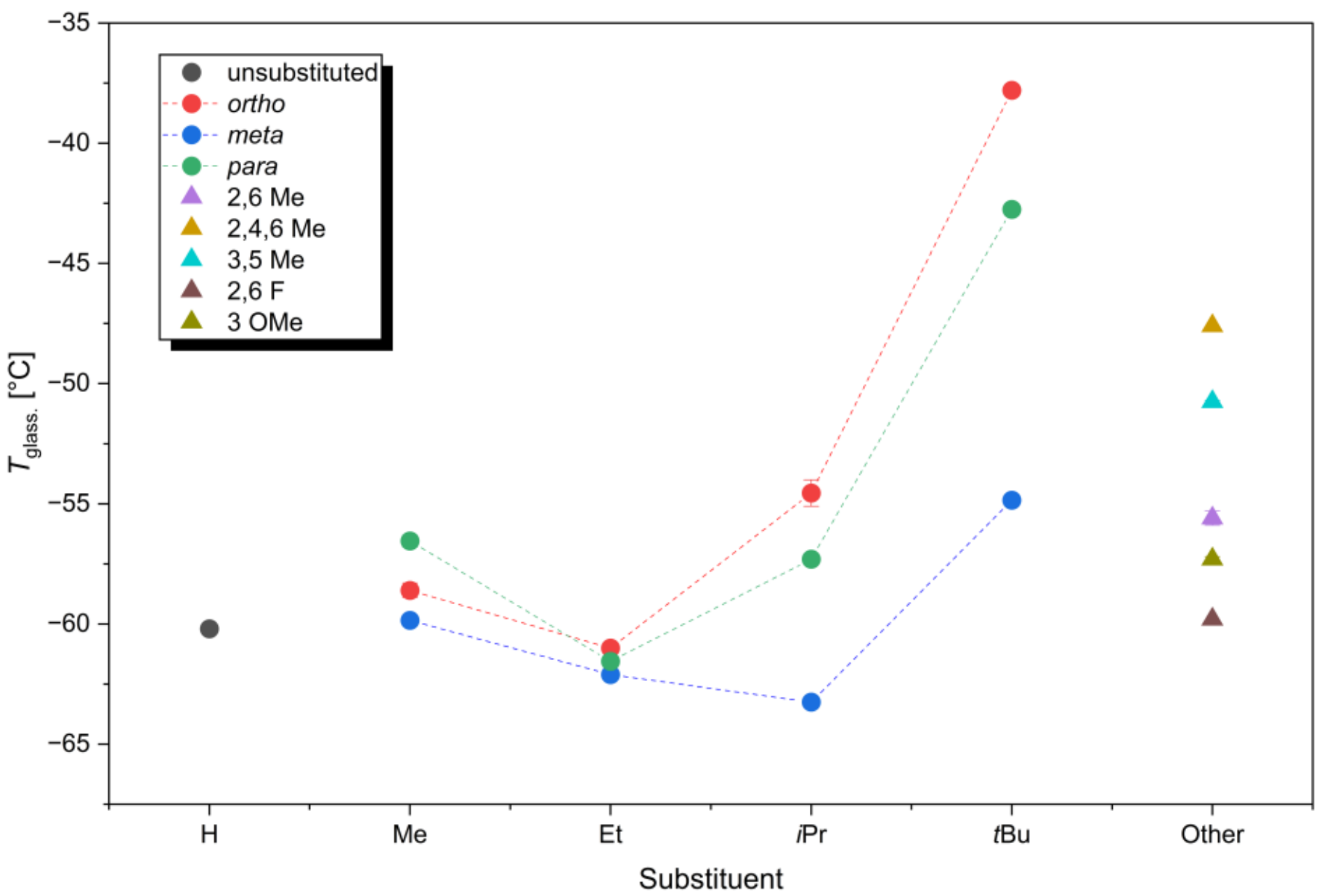

Figure S8: Glass transition temperature $T_{glass.}$ of the (*Z*)-Isomer of substituted *p*-OHex-AB 4a-r.

Table S9: Threefold measurement of the isomerization temperature ($T_{iso.}$) of the (*Z*)-Isomer.

Compound	1. $T_{iso.}$ [°C]	2. $T_{iso.}$ [°C]	⌀ $T_{iso.}$ [°C]	Δ $T_{iso.}$ [°C]
**4a**: R = H	70.4	70.1	70.3	0.2
**4b**: R = 2-Me	72.9	72.1	72.5	0.4
**4c**: R = 2-Et	70.1	70.4	70.3	0.2
**4d**: R = 2-*i*Pr	72.5	72.4	72.5	0.0
**4e**: R = 2-*t*Bu	69.8	69.8	69.8	0.0
**4f**: R = 3-Me	69.7	70.4	70.1	0.4
**4g**: R = 3-Et	71.4	70.4	70.9	0.5
**4h**: R = 3-*i*Pr	72.6	72.9	72.8	0.2
**4i**: R = 3-*t*Bu	74.7	74.7	74.7	0.0
**4j**: R = 4-Me	69.1	70.5	69.8	0.7
**4k**: R = 4-Et	67.4	66.8	67.1	0.3
**4l**: R = 4-*i*Pr	68.2	61.5	64.9	3.4
**4m**: R = 4-*t*Bu	78.2	78.9	78.6	0.4
**4n**: R = 2,6-Me	96.4	101.0	98.7	2.3
**4o**: R = 2,4,6-Me	97.8	98.5	98.2	0.4
**4p**: R = 3,5-Me	68.3	68.0	68.2	0.1
**4q**: R = 2,6-F	101.7	103.4	102.6	0.9
**4r**: R = 3-OMe	71.1	70.5	70.8	0.3

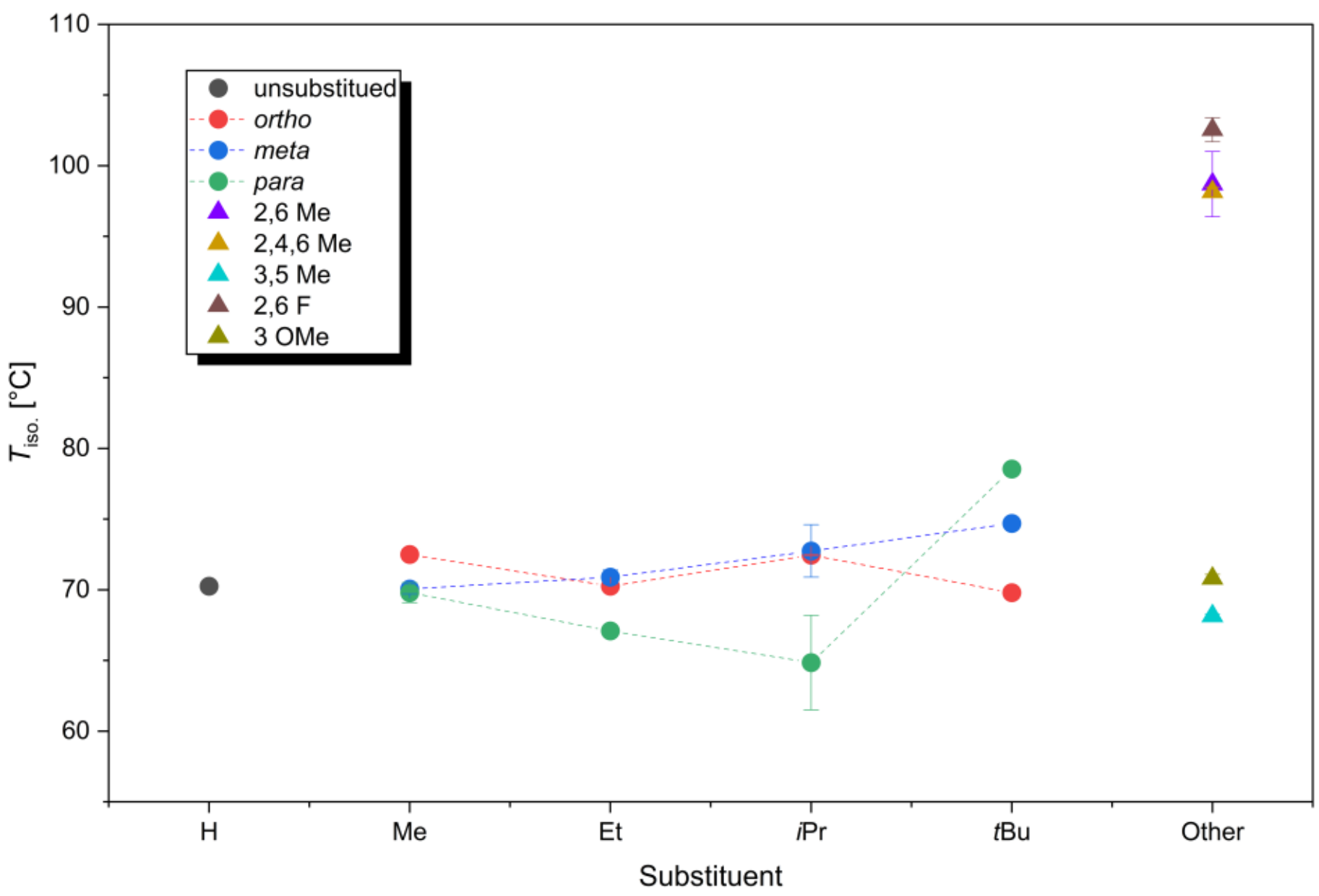

Figure S9: Isomerization temperature $T_{iso.}$ of the (*Z*)-Isomer of substituted *p*-OHex-AB 4a-r.

Table S10: Threefold measurement of the crystallization temperature ($T_{cry.}$) of the (*E*)-Isomer.

Compound	1. $T_{cry.}$ [°C]	2. $T_{cry.}$ [°C]	⌀ $T_{cry.}$ [°C]	Δ$T_{cry.}$ [°C]
**4a**: R = H	48.8	49.4	49.1	0.3
**4b**: R = 2-Me	54.9	54.2	54.6	0.3
**4c**: R = 2-Et	-3.8	-3.6	-3.7	0.1
**4d**: R = 2-*i*Pr	-31.4	-32.2	-31.8	0.4
**4e**: R = 2-*t*Bu	14.6	15.4	15.0	0.4
**4f**: R = 3-Me	38.1	36.6	37.4	0.8
**4g**: R = 3-Et	22.0	24.2	23.1	1.1
**4h**: R = 3-*i*Pr	6.5	5.2	5.9	0.7
**4i**: R = 3-*t*Bu	3.8	3.2	3.5	0.3
**4j**: R = 4-Me	61.0	60.9	61.0	0.1
**4k**: R = 4-Et	44.3	43.2	43.8	0.5
**4l**: R = 4-*i*Pr	74.6	75.6	75.0	0.5
**4m**: R = 4-*t*Bu	95.3	94.4	94.9	0.4
**4n**: R = 2,6-Me	10.4	9.6	10.0	0.4
**4o**: R = 2,4,6-Me	51.7	51.6	51.7	0.1
**4p**: R = 3,5-Me	28.7	24.7	26.7	2.0
**4q**: R = 2,6-F	8.4	5.5	7.0	1.5
**4r**: R = 3-OMe	30.1	32.7	31.4	1.3

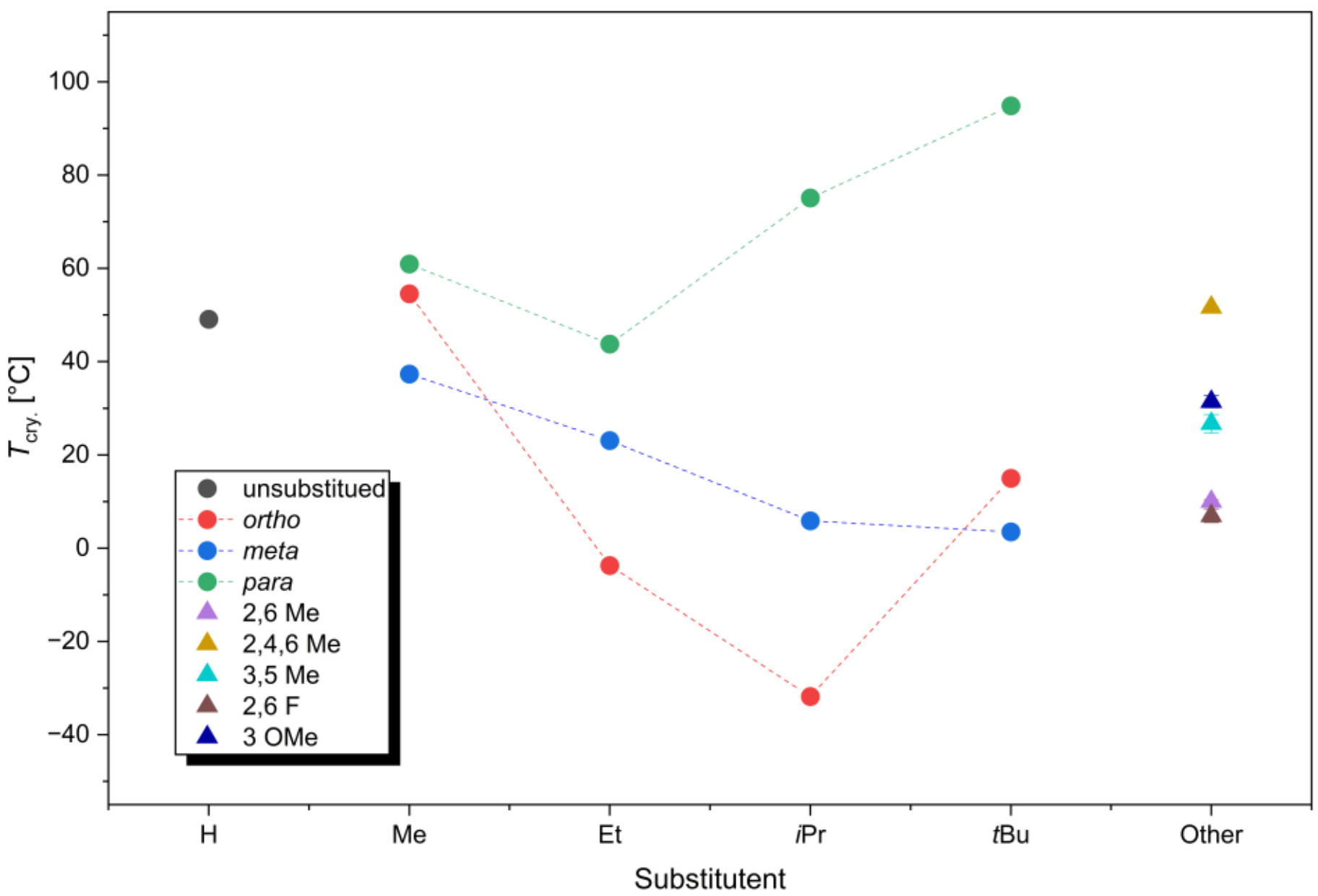

Figure S10: Crystallization temperature $T_{cry.}$ of the (*E*)-Isomer of substituted *p*-OHex-AB 4a-r.

Table S11: Twofold measurement of the melting point ($T_{melt.}$) of the (*E*)-Isomer.

Compound	1. $T_{melt.}$ [°C]	2. $T_{melt.}$ [°C]	⌀ $T_{melt.}$ [°C]	Δ $T_{melt.}$ [°C]
**4a**: R = H	56.5	56.4	56.5	0.1
**4b**: R = 2-Me	66.8	66.8	66.8	0.0
**4c**: R = 2-Et	31.2	31.5	31.4	0.2
**4d**: R = 2-*i*Pr	44.4	44.4	44.4	0.0
**4e**: R = 2-*t*Bu	54.6	54.6	54.6	0.0
**4f**: R = 3-Me	50.8	50.7	50.8	0.1
**4g**: R = 3-Et	44.1	44.3	44.2	0.1
**4h**: R = 3-*i*Pr	37.7	35.6	36.7	1.1
**4i**: R = 3-*t*Bu	54.3	54.6	54.5	0.2
**4j**: R = 4-Me	76.2	77.7	77.0	0.8
**4k**: R = 4-Et	64.5	64.7	64.6	0.1
**4l**: R = 4-*i*Pr	92.7	92.7	92.7	0.0
**4m**: R = 4-*t*Bu	116.7	116.4	11.6	0.1
**4n**: R = 2,6-Me	25.1	25.1	25.1	0.0
**4o**: R = 2,4,6-Me	59.6	59.4	59.5	0.1
**4p**: R = 3,5-Me	57.9	57.8	57.9	0.1
**4q**: R = 2,6-F	40.9	40.9	40.9	0.0
**4r**: R = 3-OMe	47.0	47.0	47.0	0.0

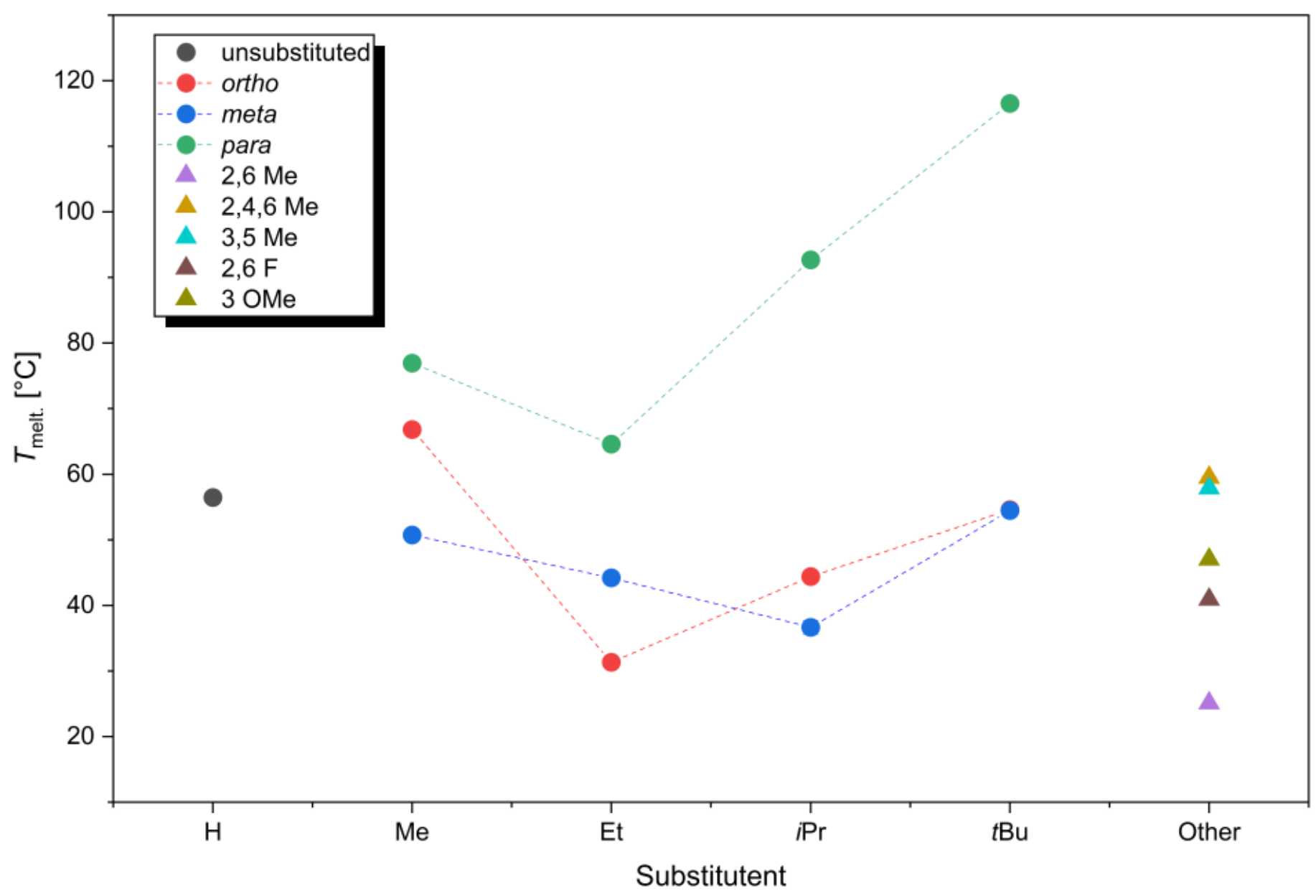

Figure S11: Melting point $T_{melt.}$ of the (*E*)-Isomer of substituted *p*-OHex-AB 4a-r.

Table S12: Twofold measurement of the melting enthalpy ($\Delta H_{melt.}$) of the (*E*)-Isomer.

Compound	1. $\Delta H_{melt.}$ [J/g]	2. $\Delta H_{melt.}$ [J/g]	⌀ $\Delta H_{melt.}$ [J/g]	$\Delta\Delta H_{melt.}$ [J/g]
**4a**: R = H	118.9	117.2	118.1	0.9
**4b**: R = 2-Me	134.6	137.1	135.9	1.3
**4c**: R = 2-Et	92.9	93.9	93.4	0.5
**4d**: R = 2-*i*Pr	101.4	99.7	100.6	0.9
**4e**: R = 2-*t*Bu	92.5	95.8	94.2	1.7
**4f**: R = 3-Me	115.6	115.2	115.4	0.2
**4g**: R = 3-Et	115.3	113.3	114.3	1.0
**4h**: R = 3-*i*Pr	96.3	97.7	97.0	0.7
**4i**: R = 3-*t*Bu	83.8	84.0	83.9	0.1
**4j**: R = 4-Me	127.1	127.6	127.4	0.3
**4k**: R = 4-Et	81.1	79.4	80.3	0.8
**4l**: R = 4-*i*Pr	84.2	86.2	85.2	1.0
**4m**: R = 4-*t*Bu	88.4	91.2	89.8	1.4
**4n**: R = 2,6-Me	97.4	95.5	96.5	1.0
**4o**: R = 2,4,6-Me	125.2	124.6	124.9	0.3
**4p**: R = 3,5-Me	101.5	99.0	100.3	1.3
**4q**: R = 2,6-F	93.9	91.9	92.9	1.0
**4r**: R = 3-OMe	117.3	119.8	118.6	1.3

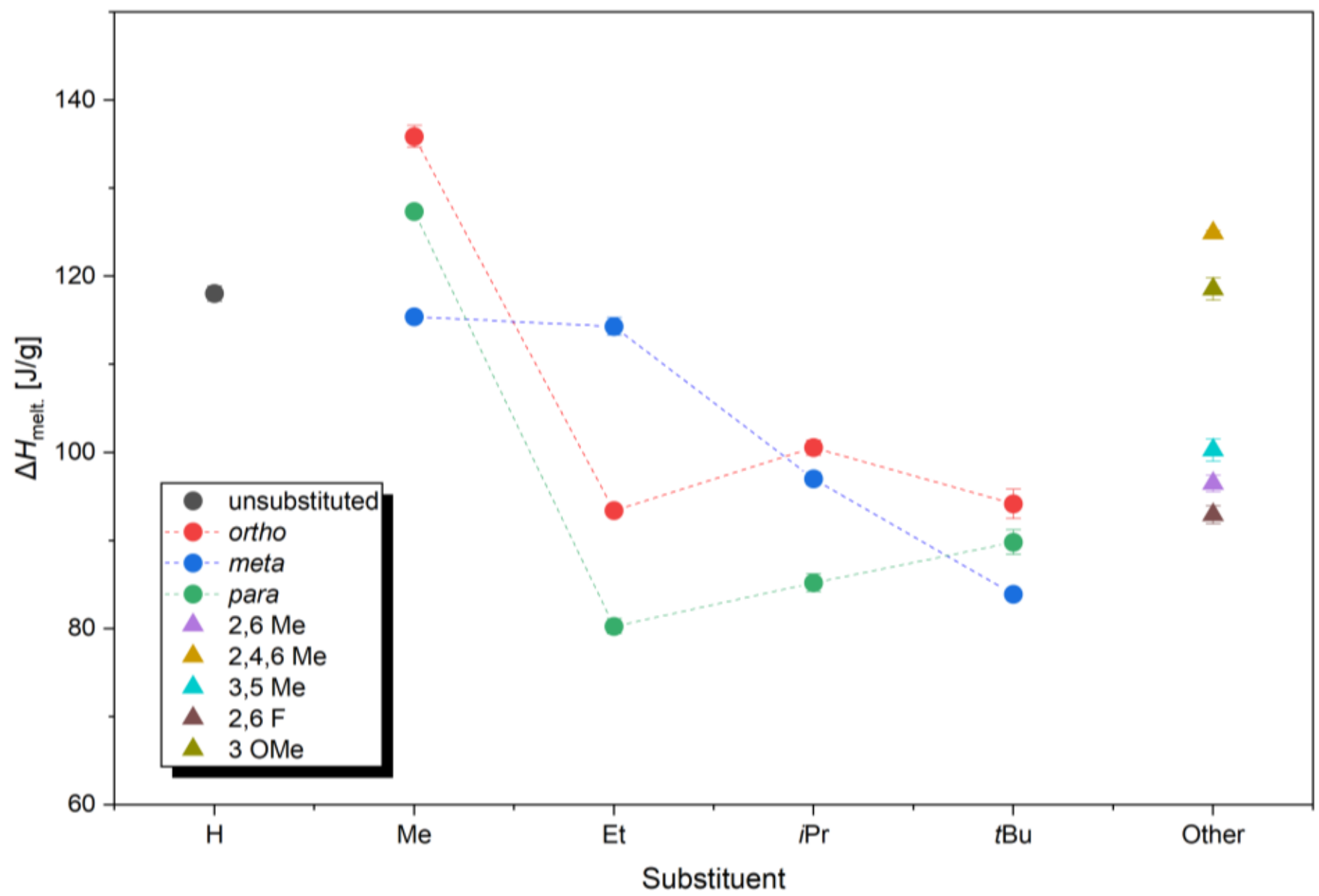

Figure S12: Melting enthalpy ($\Delta H_{melt.}$) of the (*E*)-Isomer of substituted *p*-OHex-AB 4a-r.

Table S13: Twofold measurement of the crystallization enthalpy ($\Delta H_{cry.}$) of the (*E*)-Isomer.

Compound	1. $\Delta H_{cry.}$ [J/g]	2. $\Delta H_{cry.}$ [J/g]	∅ $\Delta H_{cry.}$ [J/g]	$\Delta\Delta H_{cry.}$ [J/g]
**4a**: R = H	-119.3	-118.2	-118.8	0.5
**4b**: R = 2-Me	-131.4	-133.6	-132.5	1.1
**4c**: R = 2-Et	-73.4	-75.4	-74.4	1.0
**4d**: R = 2-*i*Pr	-51.6	-51.3	-51.5	0.2
**4e**: R = 2-*t*Bu	-84.2	-80.8	-82.5	1.7
**4f**: R = 3-Me	-112.2	-112.3	-112.3	0.0
**4g**: R = 3-Et	-111.0	-109.5	-110.3	0.8
**4h**: R = 3-*i*Pr	-85.9	-86.1	-86.0	0.1
**4i**: R = 3-*t*Bu	-64.6	-63.9	-64.3	0.4
**4j**: R = 4-Me	-124.5	-124.5	-124.5	0.0
**4k**: R = 4-Et	-73.8	-71.4	-72.6	1.2
**4l**: R = 4-*i*Pr	-80.2	-82.6	-81.4	1.2
**4m**: R = 4-*t*Bu	-85.2	-87.4	-86.3	1.1
**4n**: R = 2,6-Me	-92.3	-91.1	-91.7	0.6
**4o**: R = 2,4,6-Me	-124.7	-123.3	-124.0	0.7
**4p**: R = 3,5-Me	-91.3	-87.0	-89.2	2.2
**4q**: R = 2,6-F	-91.6	-89.6	-90.6	1.0
**4r**: R = 3-OMe	-109.6	-113.3	-111.5	1.9

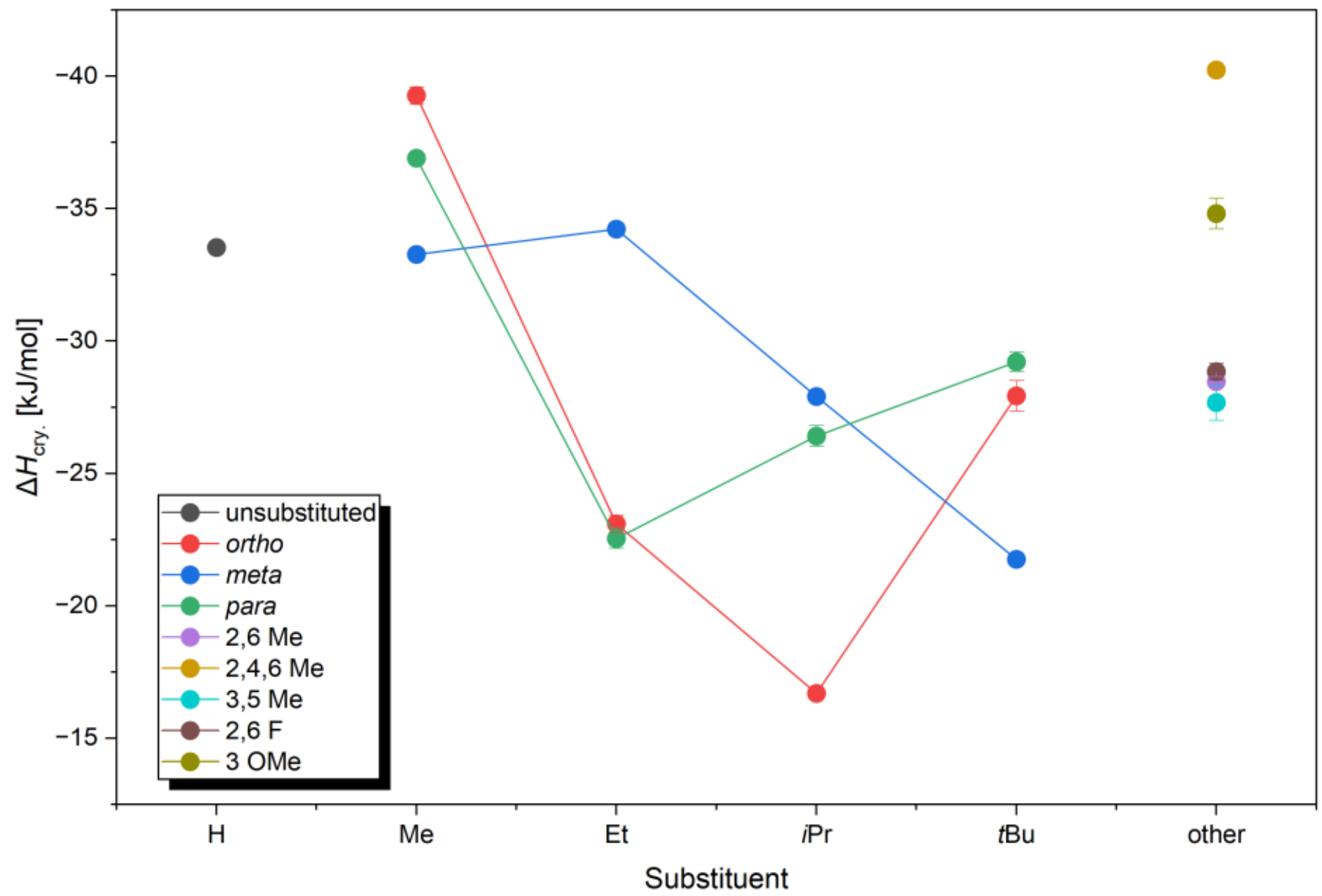

Figure S13: crystallization enthalpy ($\Delta H_{cry.}$) of the (*E*)-Isomer of substituted *p*-OHex-AB 4a-r.

Table S14: Calculation of the isomerization enthalpy ($\Delta H_{iso.}$) at $PSS_{365,\ max.}$ for 100% of the (*Z*)-Isomer.

Compound		$\Delta H_{iso.,\ meas.}$ [J/g]	$PSS_{365,\ meas.}$ [%]	$PSS_{365,\ max.}$ [%]	$\Delta H_{iso.,\ max.}$ [J/g]	$\Delta H_{iso.,\ 100\%}$ [J/g]
**4a**: R = H	1.	-168.3	91.2	93.3	-172.2	-184.5
	2.	-171.8	91.9		-174.4	-186.9
	Ø	-170.1	91.6		-173.3	-185.7
	Δ	1.8	0.4		1.1	1.2
**4b**: R = 2-Me	1.	-154.7	87.9	92.8	-163.3	-176.0
	2.	-160.3	92.8		-160.3	-172.7
	Ø	-157.5	90.4		-161.8	-174.4
	Δ	2.8	2.5		1.5	1.6
**4c**: R = 2-Et	1.	-142.6	91.5	93.2	-145.2	-155.8
	2.	-155.0	93.2		-155.0	-166.3
	Ø	-148.8	92.4		-150.1	-161.1
	Δ	6.2	0.9		4.9	5.2
**4d**: R = 2-iPr	1.	-135.5	92.5	92.7	-135.8	-146.5
	2.	-135.1	92.7		-135.1	-145.7
	Ø	-135.3	92.6		-135.4	-146.1
	Δ	0.2	0.1		0.3	0.4
**4e**: R = 2-tBu	1.	-125.7	89.2	90.0	-126.8	-140.9
	2.	-128.6	89.1		-129.9	-144.3
	Ø	-127.2	89.2		-128.4	-142.6
	Δ	1.5	0.1		1.5	1.7
**4f**: R = 3-Me	1.	-156.2	87.5	91.8	-163.9	-178.5
	2.	-161.3	90.8		-163.1	-177.6
	Ø	-158.8	89.2		-163.5	-178.1
	Δ	2.6	1.7		0.4	0.4
**4g**: R = 3-Et	1.	-165.7	92.7	92.7	-165.7	-178.7
	2.	-163.8	91.9		-165.2	-178.2
	Ø	-164.8	92.3		-165.5	-178.5
	Δ	0.9	0.4		0.2	0.3
**4h**: R = 3-iPr	1.	-159.1	93.0	94.0	-160.8	-171.1
	2.	-162.8	93.9		-163.0	-173.4
	Ø	-161.0	93.5		-161.9	-172.2
	Δ	1.9	0.5		1.1	1.2
**4i**: R = 3-tBu	1.	-147.5	94.2	96.3	-150.8	-156.6
	2.	-147.0	94.0		-150.6	-156.4
	Ø	-147.3	94.1		-150.7	-156.5
	Δ	0.3	0.1		0.1	0.1

Compound		$\Delta H_{iso., meas.}$ [J/g]	$PSS_{365, meas.}$ [%]	$PSS_{365, max.}$ [%]	$\Delta H_{iso., max.}$ [J/g]	$\Delta H_{iso., 100\%}$ [J/g]
**4j**: R = 4-Me	1.	-163.7	86.8	88.3	-166.5	-188.6
	2.	-162.3	83.2		-172.2	-195.1
	Ø	-163.0	85.0		-169.4	-191.8
	Δ	0.7	1.8		2.9	3.2
**4k**: R = 4-Et	1.	-146.7	82.8	92.8	-164.4	-177.2
	2.	-146.4	82.4		-164.9	-177.7
	Ø	-146.6	82.6		-164.6	-177.4
	Δ	0.1	0.2		0.2	0.2
**4l**: R = 4-*i*Pr	1.	-143.9	85.2	86.3	-145.8	-168.9
	2.	-142.2	85.2		-144.0	-166.9
	Ø	-143.1	85.2		-144.9	-167.9
	Δ	0.9	0		0.9	1.0
**4m**: R = 4-*t*Bu	1.	-138.3	83.6	88.9	-147.0	-165.4
	2.	-148.8	85.9		-154.0	-173.2
	Ø	-143.6	84.8		-150.5	-169.3
	Δ	5.3	1.2		3.5	3.9
**4n**: R = 2,6-Me	1.	-107.5	91.5	91.7	-107.7	-117.5
	2.	-103.6	90.9		-104.5	-114.0
	Ø	-105.6	91.2		-106.1	-115.7
	Δ	2.0	0.3		1.6	1.8
**3o**: R = 2,4,6-Me	1.	-111.0	84.4	85.1	-111.9	-131.5
	2.	-108.8	83.2		-111.3	-130.8
	Ø	-109.9	83.8		-111.6	-131.1
	Δ	1.1	0.6		0.3	0.4
**3p**: R = 3,5-Me	1.	-153.0	89.7	91.5	-156.1	-170.6
	2.	-156.9	91.5		-156.9	-171.5
	Ø	-155.0	90.6		-156.5	-171.0
	Δ	2.0	0.9		0.4	0.5
**3q**: R = 2,6-F	1.	-110.9	87.3	87.3	-127.0	-127.0
	2.	-109.4	87.2		-125.3	-125.5
	Ø	-110.2	87.3		-126.2	-126.2
	Δ	0.8	0.0		0.9	0.8
**3r**: R = 3-OMe	1.	-139.5	77.2	83.3	-150.5	-180.7
	2.	-148.7	82.5		-150.1	-180.2
	Ø	-144.1	79.9		-150.3	-180.5
	Δ	4.6	2.7		0.2	0.2

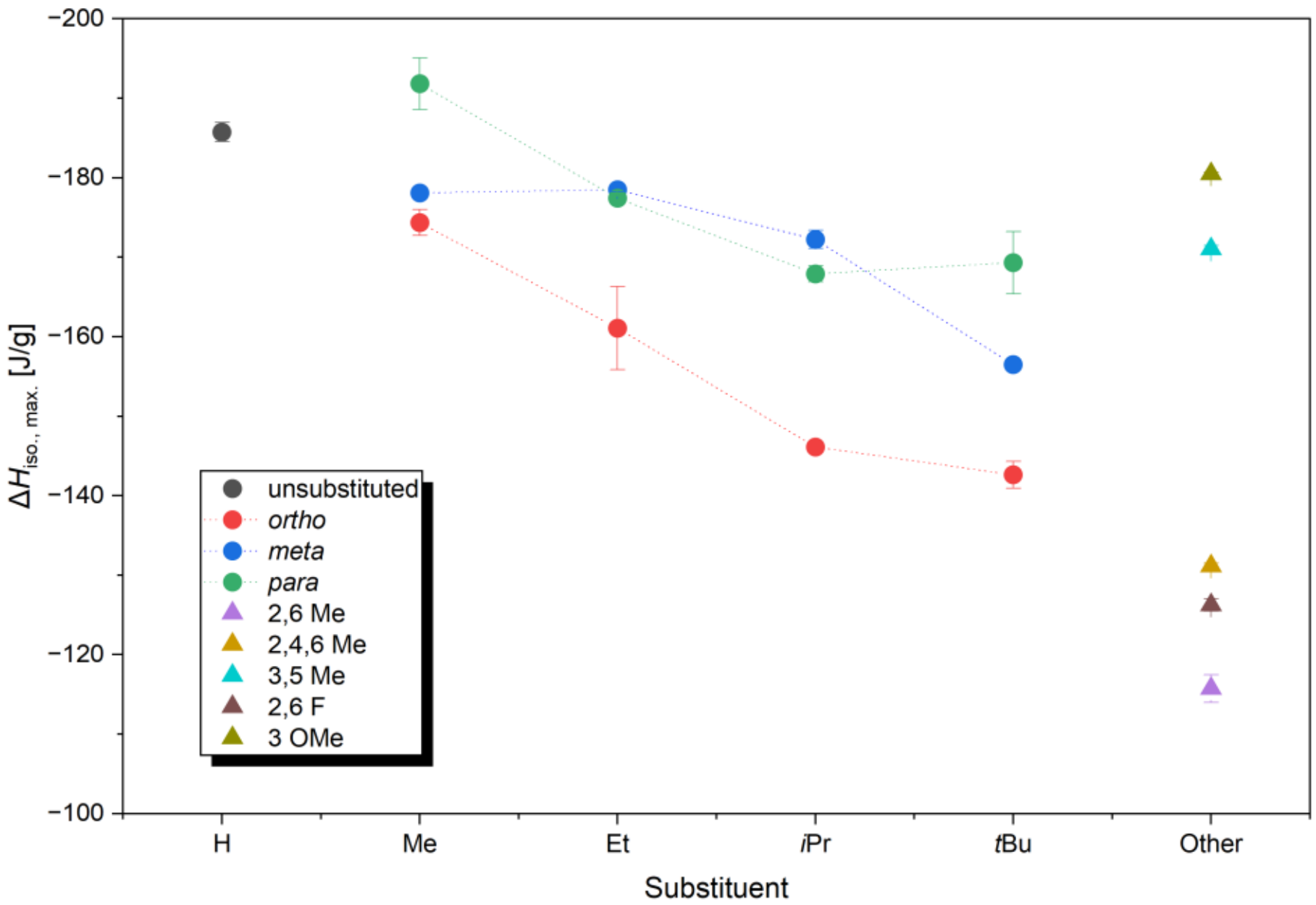

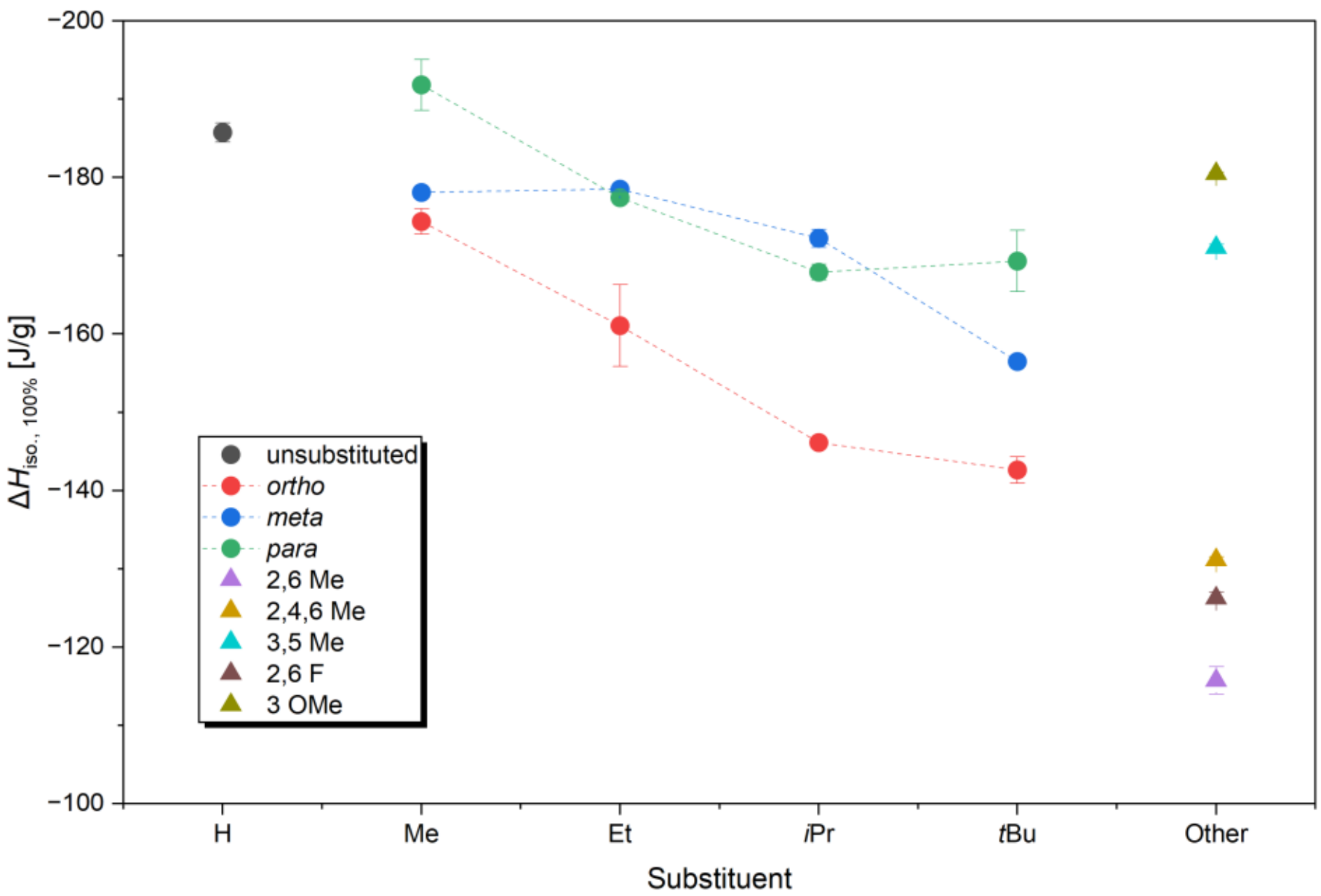

Figure S14: Isomerization enthalpy ($\Delta H_{iso.}$) of the (*E*)-Isomer of substituted *p*-OHex-AB 4a-r for maximal conversion at PSS_{365} (top) and 100% conversion (bottom).

Table S15: Calculated total storage enthalpy ($\Delta H_{tot.}$) at $PSS_{365, max.}$ and for 100% conversion.

Compound	$\Delta H_{tot., max.}$	$\Delta\Delta H_{tot., max.}$	$\Delta H_{tot., 100\%}$	$\Delta\Delta H_{tot., 100\%}$
**4a**: R = H	-292.0	1.7	-304.5	1.8
**4b**: R = 2-Me	-294.3	2.6	-306.9	2.0
**4c**: R = 2-Et	-224.5	5.0	-235.5	6.2
**4d**: R = 2-*i*Pr	-186.9	0.5	-197.6	0.5
**4e**: R = 2-tBu	-210.9	3.2	-225.1	3.4
**4f**: R = 3-Me	-275.7	0.4	-290.3	0.5
**4g**: R = 3-Et	-275.7	1.0	-288.7	1.0
**4h**: R = 3-*i*Pr	-247.9	1.2	-258.2	1.3
**4i**: R = 3-*t*Bu	-215.0	0.4	-220.7	0.5
**4j**: R = 4-Me	-293.9	2.9	-316.3	3.2
**4k**: R = 4-Et	-237.2	1.4	-250.0	1.4
**4l**: R = 4-*i*Pr	-226.3	2.1	-249.3	2.2
**4m**: R = 4-*t*Bu	-236.8	4.6	-255.6	5.0
**4n**: R = 2,6-Me	-197.8	2.2	-207.4	2.4
**4o**: R = 2,4,6-Me	-235.6	1.0	-255.1	1.1
**4p**: R = 3,5-Me	-245.6	2.6	-260.2	2.6
**4q**: R = 2,6-F	-200.8	1.7	-216.8	1.8
**4r**: R = 3-OMe	-261.8	2.0	-291.9	2.1

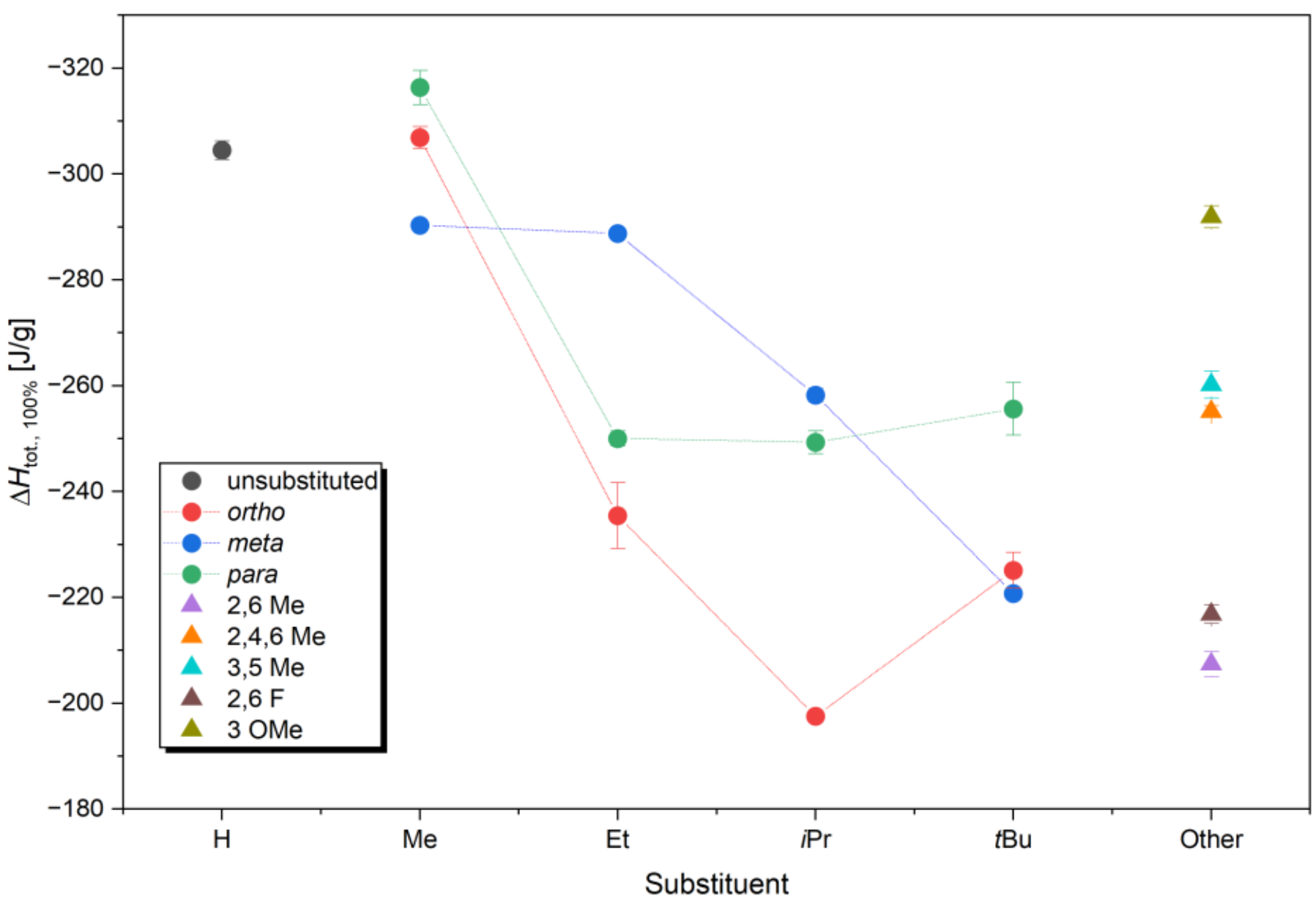

Figure S15: Total storage enthalpy ($\Delta H_{tot.}$) of substituted *p*-OHex-AB 4a-r for 100% conversion.

9. Photoliquefication efficiency

For the determination of the photoliquefication efficiency thin films of crystalline ABs were prepared and irradiated. For preparation of the crystalline thin films a solution was prepared by dissolving 50 mg of each compound in 1000 µL DCM. Then 40 µL of the solution was placed on a glass slide (76 x 26 x 1 mm) (Fig. S16a). The glass slide was placed on a heated aluminum plate to evaporate the solvent. Then, the temperature was increased above the melting point. After complete melting a second glass slide (18 mm x 18 x 0.15 mm) was placed on the molten compound. The molten compounds are distributed between the two glass slides and allowed to cool to room temperature (Fig. S16b). With this method identical crystalline thin films of each compound were prepared. This ensured that the same amount of compound with the same surface area was irradiated in identical conditions for each experiment. Irradiation of the thin films was performed at room temperature with 365 nm with a distance of 6 cm between light source and thin film (Fig. S16c). Each thin film was irradiated separately to ensure similar irradiation conditions. After irradiation the compound was dissolved in acetonitrile (2 mL) and HPLC measurements were preformed to determine the ratio between (*E*)- and (*Z*)-isomers.

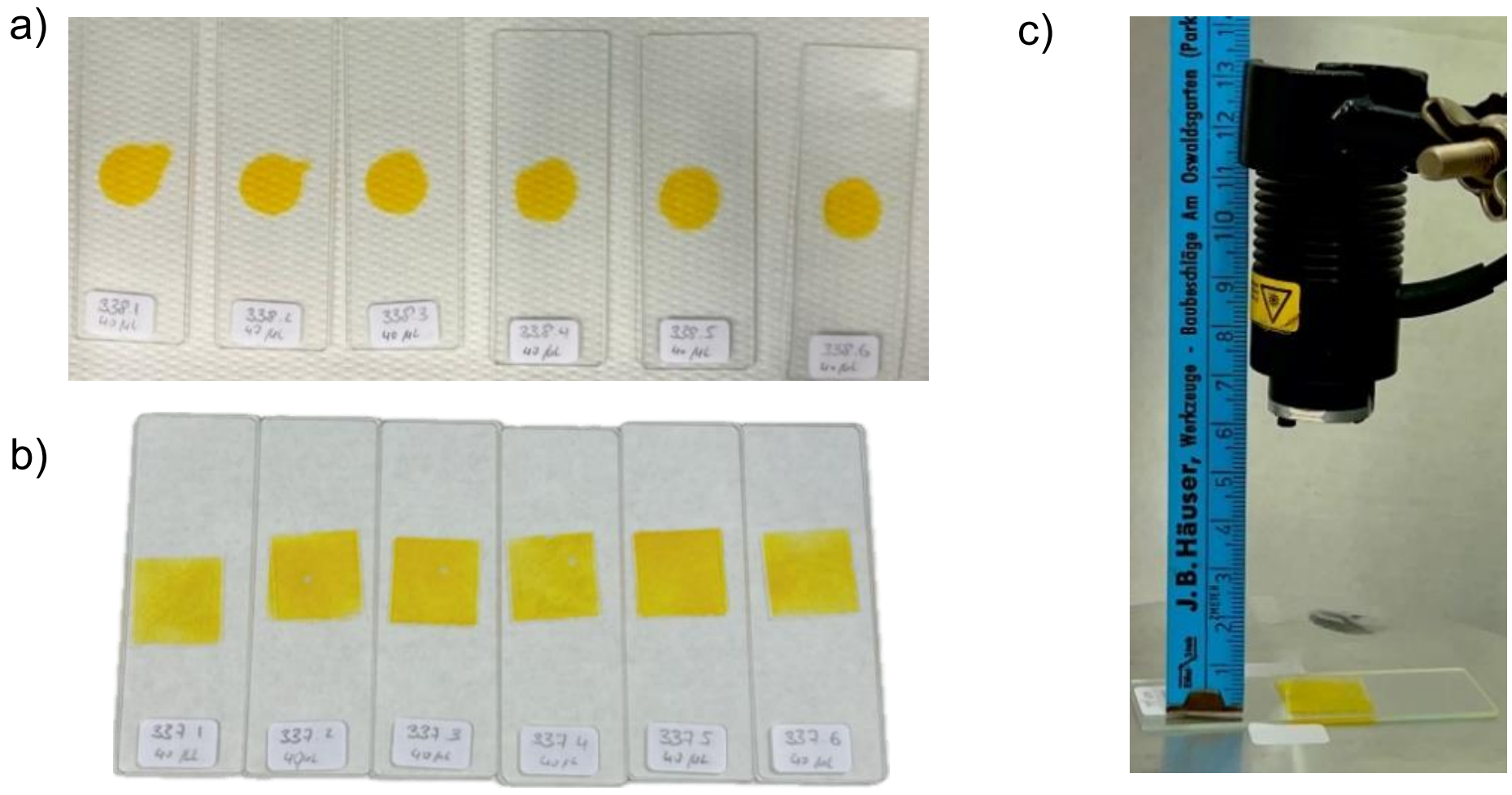

Figure S16: a) AB solution (40 µL) placed on a glass slides (76 x 26 x 1 mm) b) crystalline AB between glass slides c) Irradiation of crystalline AB thin films.

Initially, for each compound, the required irradiation time for complete photoliquefication was determined to select reasonable irradiation intervals. Therefore, each sample was monitored at 30 s intervals, and irradiation was continued until no crystalline structures could be detected:

p-OHex-AB **4a**: 2:30 min.
o-*t*Bu-AB **4e**: 3:30 min.
m-*i*Pr-AB **4h**: 1:00 min.
m-*t*Bu-AB **4i**: 2:00 min.
p-*t*Bu-AB **4m**: 37:30 min.
2,4,6-Me-AB **4o**: 10:30 min.

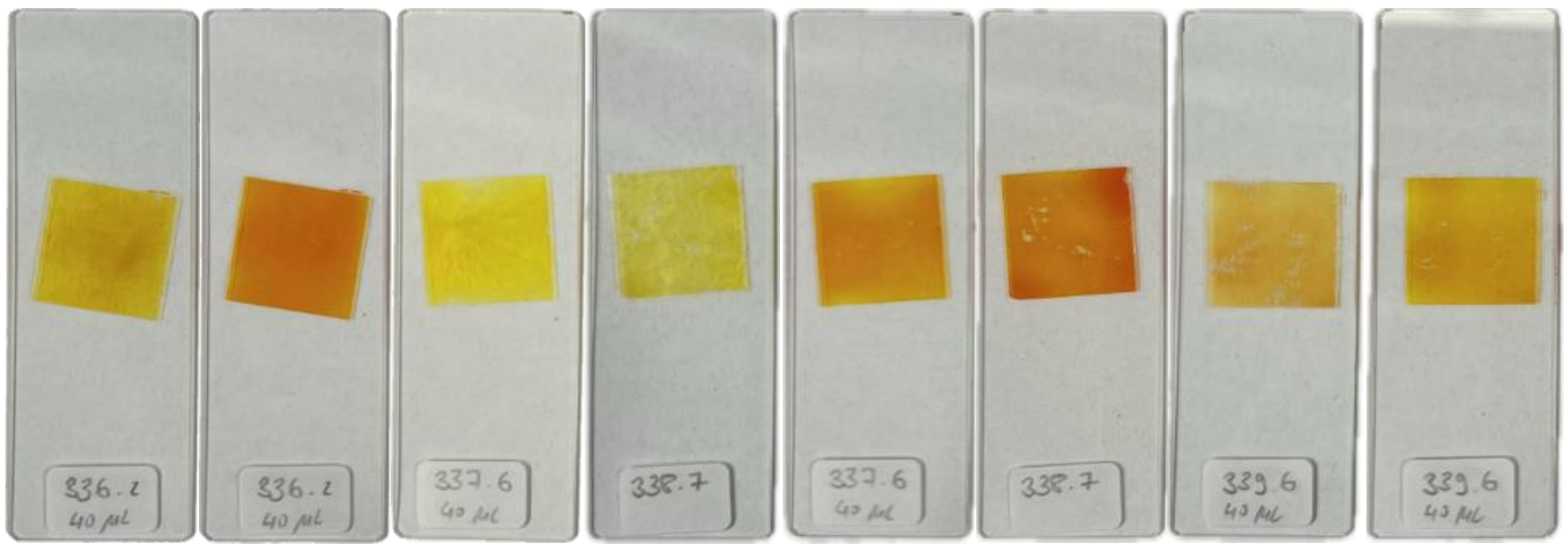

Figure S17: Crystalline thin films before and after irradiation with 365 nm until complete photoliquefication.

Tab. S16: Change of the (*Z*)-content during irradiation with 365 nm at room temperature of crystalline thin films for determination of the photoliquefication efficiency for selected AB.

p-OHex-AB **4a**		*o*-*t*Bu-AB **4e**		*m*-*i*Pr-AB **4h**	
Time [min]	(*Z*)-isomer [%]	Time [min]	(*Z*)-isomer [%]	Time [min]	(*Z*)-isomer [%]
0	0	0	0	0	0
0.5	8.7	0.25	11.7	0.25	11.9
1	21.9	0.5	21.4	0.5	25
1.5	33.9	0.75	32.5	0.75	39.4
2	47.6	1	39	1	50.1
2.5	61.8	1.25	48.2	1.25	58.9
3	77.7	1.5	54.2	1.5	77.3
3.5	87.9	1.75	64.6	1.75	84.2
		2	68.2		
		2.25	74.1		

*m-t*Bu-AB **4i**		*p-t*Bu-AB **4m**		2,4,6-Me-AB **4o**	
Time [min]	(*Z*)-isomer [%]	Time [min]	(*Z*)-isomer [%]	Time [min]	(*Z*)-isomer [%]
0	0	0	0	0	0
0.5	18.7	6	8.2	1.0	2.5
0.75	31.5	13	22.7	2.0	8.3
1.0	37.1	20	35.7	3.0	21.8
1.5	51.9	28	54.7	4.5	43.8
2.0	65.5	35	65.3	6.0	55.7
2.5	88.1			7.5	64.7
				9.0	80.7

10. Solid state structures

Suitable single crystals for X-ray structure determination were selected and transferred in protective perfluoropolyether oil on a microscope slide. The selected and mounted crystals were transferred to the cold gas steam on the diffractometer. The diffraction data were obtained at 100K on a Bruker D8 three circle diffractometer, equipped with a PHOTON 100 CMOS detector and a IμS microfocus sources with Quazar mirror optics (Mo-Kα radiation, λ = 0.71073 Å). The data obtained were integrated with SAINT and a semi-empirical absorption correction from equivalents with SADABS2016/2 was applied.[11] The structures were solved by direct methods using SHELXT-2018/2.[12] Structure refinement was done using SHELXT-2025/1.[13] All non-hydrogen atoms were refined anisotropically and C-H hydrogen atoms were positioned at geometrically calculated positions and refined using a riding model. The isotropic displacement parameters of all hydrogen atoms were fixed to 1.2x or 1.5x (CH_3 hydrogens) the U(eq) value of the atoms they are linked to.

XRD of 1-(3-*tert*-butylphenyl)-2-[4-(hexoxy)phenyl]diazene (4i)

Table S16 Crystal data and structure refinement for CCDC 2547606.

Identification code	CCDC 2547606
Empirical formula	$C_{22}H_{30}N_2O$
Formula weight	338.48
Temperature/K	100.00
Crystal system	triclinic
Space group	P-1
a/Å	7.3592(9)

b/Å	11.5037(12)
c/Å	12.3997(14)
α/°	84.704(4)
β/°	80.427(5)
γ/°	72.791(5)
Volume/Å^3	987.7(2)
Z	2
ρ_{calc}g/cm^3	1.138
μ/mm^{-1}	0.070
F(000)	368.0
Crystal size/mm^3	0.685 × 0.327 × 0.064
Radiation	MoKα (λ = 0.71073)
2Θ range for data collection/°	4.874 to 58.322
Index ranges	-10 ≤ h ≤ 10, -15 ≤ k ≤ 15, -16 ≤ l ≤ 16
Reflections collected	51619
Independent reflections	5295 [R_{int} = 0.0667, R_{sigma} = 0.0351]
Data/restraints/parameters	5295/0/231
Goodness-of-fit on F^2	1.039
Final R indexes [I>=2σ (I)]	R_1 = 0.0446, wR_2 = 0.1241
Final R indexes [all data]	R_1 = 0.0539, wR_2 = 0.1337
Largest diff. peak/hole / e Å^{-3}	0.41/-0.20

Table S17 Atomic Coordinates (×10^4) and Equivalent Isotropic Displacement Parameters (Å^2×10^3) for CCDC 2547606. U_{eq} is defined as 1/3 of the trace of the orthogonalised U_{IJ} tensor.

	x	*y*	*z*	U(eq)
O1	6927.5(11)	2610.0(6)	2679.6(5)	26.80(17)
N1	4739.2(11)	7620.6(7)	1819.1(6)	22.62(17)
N2	4051.1(11)	8249.5(7)	2648.9(6)	21.49(17)
C1	10658.0(16)	-4022.5(9)	1731.0(10)	32.8(2)
C2	10248.4(15)	-2746.3(8)	1194.7(8)	26.8(2)
C3	9325.4(13)	-1754.2(8)	2009.4(7)	22.19(19)
C4	8870.5(13)	-469.3(8)	1484.8(7)	21.70(18)

Table S17 Atomic Coordinates (×10[4]) and Equivalent Isotropic Displacement Parameters (Å[2]×10[3]) for CCDC 2547606. U_{eq} is defined as 1/3 of the trace of the orthogonalised U_{IJ} tensor.

	x	*y*	*z*	U(eq)
C5	8057.6(13)	506.8(8)	2328.9(7)	23.13(19)
C6	7658.2(13)	1777.7(8)	1808.0(7)	22.95(19)
C7	6431.2(13)	3822.9(8)	2414.3(8)	22.92(19)
C8	6545.8(14)	4334.8(9)	1352.0(8)	25.2(2)
C9	5974.5(14)	5595.9(9)	1194.2(8)	24.9(2)
C10	5290.2(13)	6346.8(8)	2075.6(7)	21.78(19)
C11	5192.3(14)	5825.1(9)	3143.4(8)	24.5(2)
C12	5759.6(15)	4579.5(9)	3306.9(8)	26.0(2)
C13	2877.4(12)	10238.3(8)	3327.6(7)	19.51(18)
C14	3516.1(12)	9532.3(8)	2414.7(7)	19.35(18)
C15	3617.4(13)	10083.5(9)	1361.6(7)	22.58(19)
C16	3090.5(14)	11338.0(9)	1253.0(7)	24.7(2)
C17	2455.6(13)	12047.9(8)	2174.7(7)	22.70(19)
C18	2333.2(12)	11509.8(8)	3228.8(7)	19.36(18)
C19	1650.4(13)	12245.5(8)	4259.4(7)	22.08(19)
C20	-120.5(14)	11919.8(9)	4908.1(8)	27.1(2)
C21	1091.1(18)	13615.9(9)	3989.6(9)	33.7(2)
C22	3279.8(15)	11926.8(9)	4962.7(8)	27.9(2)

Table S18 Bond Lengths for CCDC 2547606.

Atom	Atom	Length/Å	Atom	Atom	Length/Å
O1	C6	1.4378(10)	C9	C10	1.3886(12)
O1	C7	1.3572(11)	C10	C11	1.4020(13)
N1	N2	1.2594(10)	C11	C12	1.3745(13)
N1	C10	1.4206(12)	C13	C14	1.3887(11)
N2	C14	1.4255(11)	C13	C18	1.3969(12)
C1	C2	1.5208(13)	C14	C15	1.3984(12)

Table S18 Bond Lengths for CCDC 2547606.

Atom	Atom	Length/Å	Atom	Atom	Length/Å
C2	C3	1.5199(13)	C15	C16	1.3790(13)
C3	C4	1.5240(12)	C16	C17	1.4002(13)
C4	C5	1.5249(12)	C17	C18	1.3944(12)
C5	C6	1.5086(13)	C18	C19	1.5285(11)
C7	C8	1.3922(13)	C19	C20	1.5396(13)
C7	C12	1.4030(12)	C19	C21	1.5267(13)
C8	C9	1.3906(13)	C19	C22	1.5367(13)

Table S18 Bond Angles for CCDC 2547606.

Atom	Atom	Atom	Angle/˚	Atom	Atom	Atom	Angle/˚
C7	O1	C6	118.30(7)	C11	C12	C7	120.62(9)
N2	N1	C10	113.29(8)	C14	C13	C18	121.57(8)
N1	N2	C14	114.37(7)	C13	C14	N2	114.98(7)
C3	C2	C1	112.99(8)	C13	C14	C15	120.45(8)
C2	C3	C4	113.77(7)	C15	C14	N2	124.57(8)
C3	C4	C5	112.47(7)	C16	C15	C14	118.56(8)
C6	C5	C4	112.29(7)	C15	C16	C17	120.90(8)
O1	C6	C5	107.10(7)	C18	C17	C16	121.11(8)
O1	C7	C8	124.98(8)	C13	C18	C19	119.55(7)
O1	C7	C12	115.13(8)	C17	C18	C13	117.42(8)
C8	C7	C12	119.88(9)	C17	C18	C19	123.03(8)
C9	C8	C7	119.12(8)	C18	C19	C20	109.08(7)
C10	C9	C8	121.12(9)	C18	C19	C22	108.96(7)
C9	C10	N1	116.36(8)	C21	C19	C18	112.06(7)
C9	C10	C11	119.45(9)	C21	C19	C20	108.34(8)
C11	C10	N1	124.20(8)	C21	C19	C22	108.58(8)
C12	C11	C10	119.81(8)	C22	C19	C20	109.80(8)

Table S19 Torsion Angles for CCDC 2547606.

A	B	C	D	Angle/˚	A	B	C	D	Angle/˚
O1	C7	C8	C9	179.48(8)	C9	C10	C11	C12	-0.46(14)
O1	C7	C12	C11	-179.28(8)	C10	N1	N2	C14	179.02(7)
N1	N2	C14	C13	-175.82(7)	C10	C11	C12	C7	-0.21(15)
N1	N2	C14	C15	3.58(13)	C12	C7	C8	C9	-0.49(15)
N1	C10	C11	C12	179.97(8)	C13	C14	C15	C16	0.69(13)
N2	N1	C10	C9	176.97(8)	C13	C18	C19	C20	-59.21(10)
N2	N1	C10	C11	-3.45(13)	C13	C18	C19	C21	-179.17(8)
N2	C14	C15	C16	-178.67(8)	C13	C18	C19	C22	60.65(10)
C1	C2	C3	C4	-178.95(8)	C14	C13	C18	C17	-0.03(13)
C2	C3	C4	C5	-176.58(8)	C14	C13	C18	C19	-179.67(8)
C3	C4	C5	C6	178.50(7)	C14	C15	C16	C17	-0.39(14)
C4	C5	C6	O1	-179.26(7)	C15	C16	C17	C18	-0.12(15)
C6	O1	C7	C8	1.02(14)	C16	C17	C18	C13	0.33(13)
C6	O1	C7	C12	-179.01(8)	C16	C17	C18	C19	179.96(8)
C7	O1	C6	C5	-179.10(7)	C17	C18	C19	C20	121.17(9)
C7	C8	C9	C10	-0.19(15)	C17	C18	C19	C21	1.21(13)
C8	C7	C12	C11	0.69(15)	C17	C18	C19	C22	-118.97(9)
C8	C9	C10	N1	-179.73(8)	C18	C13	C14	N2	178.93(7)
C8	C9	C10	C11	0.67(14)	C18	C13	C14	C15	-0.49(13)

Table S20 Hydrogen Atom Coordinates (Å×10^4) and Isotropic Displacement Parameters (Å^2×10^3) for CCDC 2547606.

	x	*y*	*z*	U(eq)
H1A	11248.56	-4622.73	1169.85	49
H1B	9451.58	-4160.37	2103.83	49
H1C	11537.74	-4106.62	2264.86	49
H2A	11469.09	-2620.56	806.17	32
H2B	9384.85	-2674.9	643.93	32
H3A	10201.06	-1819.68	2552.19	27

Table S20 Hydrogen Atom Coordinates (Å×10^4) and Isotropic Displacement Parameters (Å^2×10^3) for CCDC 2547606.

	x	*y*	*z*	U(eq)
H3B	8119.52	-1892.51	2407.91	27
H4A	7928.12	-382.81	977.01	26
H4B	10058.98	-342.67	1050.78	26
H5A	6849.88	394.38	2749.55	28
H5B	8984.62	407.37	2848.5	28
H6A	8853.37	1908.85	1387.02	28
H6B	6699.84	1903.03	1303.09	28
H8	7008.24	3829.34	743.1	30
H9	6053.66	5949.77	470.98	30
H11	4735.14	6331.09	3752.17	29
H12	5696.4	4227.7	4031.11	31
H13	2808.99	9847.24	4036.15	23
H15	4040.08	9604.1	734.63	27
H16	3159.17	11725.86	543.11	30
H17	2102.23	12910.79	2080.36	27
H20A	-1125.38	12075.79	4440.32	41
H20B	-605.91	12419.72	5550.04	41
H20C	242.59	11056.4	5146.74	41
H21A	2205.45	13840.84	3579.73	50
H21B	664.41	14052.92	4670.19	50
H21C	44.63	13833.4	3545.58	50
H22A	3577.5	11061.88	5195.49	42
H22B	2874.41	12422.49	5608.93	42
H22C	4426.5	12092.88	4532.15	42

XRD of 1-(4-*iso*-propylphenyl)-2-[4-(hexoxy)phenyl]diazene (4l)

Table S21 Crystal data and structure refinement for CCDC_2556736.

Identification code	CCDC_2556736
Empirical formula	$C_{21}H_{28}N_2O$
Formula weight	324.45
Temperature/K	100.00
Crystal system	monoclinic
Space group	$P2_1$
a/Å	8.4429(11)
b/Å	7.5990(16)
c/Å	14.620(2)
α/°	90
β/°	94.498(11)
γ/°	90
Volume/Å^3	935.1(3)
ρ_{calc}g/cm^3	1.152
μ/mm^{-1}	0.071
F(000)	352.0
Crystal size/mm^3	0.356 × 0.277 × 0.071
Radiation	MoKα (λ = 0.71073)
2Θ range for data collection/°	4.84 to 54.964
Index ranges	-10 ≤ h ≤ 10, -9 ≤ k ≤ 9, -18 ≤ l ≤ 18
Reflections collected	21774
Independent reflections	4282 [R_{int} = 0.0508, R_{sigma} = 0.0412]
Data/restraints/parameters	4282/1/306
Goodness-of-fit on F^2	1.060
Final R indexes [I>=2σ (I)]	R_1 = 0.0431, wR_2 = 0.1167
Final R indexes [all data]	R_1 = 0.0460, wR_2 = 0.1204
Largest diff. peak/hole / e Å^{-3}	0.28/-0.23
Flack parameter	0.0(5)

Table S22 Fractional Atomic Coordinates (×10[4]) and Equivalent Isotropic Displacement Parameters (Å[2]×10[3]) for CCDC_2556736. U_{eq} is defined as 1/3 of the trace of the orthogonalised U_{IJ} tensor.

Atom	*x*	*y*	*z*	U(eq)
O1A	5727(10)	5497(13)	3400(5)	26.9(15)
N1A	607(3)	5735(3)	5581.1(16)	24.4(6)
N2A	781(3)	5217(3)	6403.7(17)	25.1(6)
C1A	-4658(12)	6370(15)	9246(7)	72(3)
C2A	-4882(3)	5349(4)	8336(2)	33.0(7)
C3A	-5451(5)	3491(6)	8483(3)	42.9(9)
C4A	-3359(4)	5385(5)	7849(3)	22.9(7)
C5A	-1966(6)	4562(6)	8202(3)	28.6(10)
C6A	-607(4)	4551(5)	7722(3)	25.6(9)
C7A	-637(5)	5375(5)	6873(3)	23.1(8)
C8A	-1998(6)	6250(5)	6518(3)	28.9(9)
C9A	-3361(4)	6266(4)	7005(3)	28.1(8)
C10A	1992(3)	5558(4)	5094(2)	20.6(6)
C11A	3370(5)	4663(5)	5393(2)	24.7(7)
C12A	4654(8)	4611(7)	4844(4)	27.3(13)
C13A	4531(7)	5466(7)	3978(4)	21.8(12)
C14A	3157(6)	6317(7)	3699(5)	24.0(10)
C15A	1885(3)	6376(4)	4235(3)	22.9(7)
O1B	5670(30)	5510(30)	3492(14)	28(4)
N1B	60(7)	5930(7)	5971(4)	22.1(14)
N2B	1293(7)	5004(7)	6062(4)	22.7(14)
C1B	-4668(18)	6403(19)	9275(10)	25(3)
C2B	-4274(8)	4757(9)	8765(4)	25.7(15)
C3B	-5851(10)	4095(13)	8305(6)	31(2)
C4B	-3041(14)	5101(13)	8078(7)	25(3)
C5B	-1562(15)	4436(18)	8124(9)	29(3)
C6B	-512(14)	4723(15)	7477(8)	24(3)

Table S22 Fractional Atomic Coordinates (×10^4) and Equivalent Isotropic Displacement Parameters (Å^2×10^3) for CCDC_2556736. U_{eq} is defined as 1/3 of the trace of the orthogonalised U_{IJ} tensor.

Atom	*x*	*y*	*z*	U(eq)
C7B	-960(14)	5663(13)	6705(7)	20(3)
C8B	-2411(13)	6449(15)	6627(7)	25(3)
C9B	-3436(13)	6128(15)	7296(8)	28(3)
C10B	2363(11)	5186(11)	5352(6)	20.0(17)
C11B	3766(12)	4326(12)	5504(6)	23(2)
C12B	4900(18)	4370(19)	4895(11)	22(3)
C13B	4633(17)	5293(19)	4128(9)	15(3)
C14B	3120(20)	6250(30)	3895(10)	31(4)
C15B	2023(9)	6152(11)	4546(7)	20(2)
C16B	7231(2)	4712(3)	3691.9(14)	32.0(4)
C17B	8317(2)	5085(3)	2936.2(15)	35.1(5)
C18B	7814(2)	4171(3)	2038.4(16)	37.3(5)
C19B	8718(3)	4763(3)	1224.0(15)	39.4(5)
C20B	10505(3)	4508(3)	1359.4(16)	40.8(5)
C21B	11305(3)	4964(4)	484.0(19)	49.1(6)

Table S23 Anisotropic Displacement Parameters (Å^2×10^3) for CCDC_2556736. The Anisotropic displacement factor exponent takes the form: $-2\pi^2[h^2a^{*2}U_{11}+2hka^*b^*U_{12}+\ldots]$.

Atom	U_{11}	U_{22}	U_{33}	U_{23}	U_{13}	U_{12}
O1A	24.4(18)	33(2)	25(2)	8.6(15)	13.3(14)	7.5(9)
N1A	24.9(11)	22.6(11)	26.1(12)	0.1(9)	3.7(9)	-1.5(8)
N2A	26.5(11)	25.4(11)	24.0(11)	-0.8(9)	4.5(9)	-0.2(9)
C1A	60(3)	86(5)	76(5)	-42(3)	40(3)	-15(2)
C2A	28.0(13)	35.6(15)	36.8(14)	0.1(13)	12.4(11)	-0.2(12)
C3A	43.7(19)	42(2)	45.8(19)	-0.8(17)	22.2(16)	-11.6(18)
C4A	22.2(15)	22.5(14)	24.5(16)	-0.5(14)	5.7(12)	0.6(12)
C5A	34(2)	28.1(17)	25.2(15)	3.4(11)	10.6(16)	0.1(18)

Table S23 Anisotropic Displacement Parameters (Å^2×10^3) for CCDC_2556736. The Anisotropic displacement factor exponent takes the form: -2π^2[h^{2}a*^{2}U$_{11}$+2hka*b*U$_{12}$+…].

Atom	U_{11}	U_{22}	U_{33}	U_{23}	U_{13}	U_{12}
C6A	27.8(17)	25.6(16)	23.7(18)	4.3(14)	3.8(15)	4.0(11)
C7A	22.4(18)	23.4(16)	23.8(18)	-1.6(14)	4.4(13)	-2.0(14)
C8A	31(2)	28.1(16)	28.0(16)	2.2(12)	7.4(15)	-0.3(16)
C9A	28.4(17)	25.7(16)	29.9(18)	6.9(14)	0.2(14)	2.8(11)
C10A	19.5(13)	19.4(12)	22.9(15)	-1.7(13)	1.6(10)	-1.1(11)
C11A	27(2)	24.6(15)	22.5(14)	3.6(12)	1.3(12)	-0.5(15)
C12A	29(2)	22(2)	30(2)	6.7(14)	0.1(15)	6.2(16)
C13A	29(2)	19.8(18)	17(2)	6.1(16)	4.1(15)	1.1(13)
C14A	22.7(18)	23.7(18)	26(2)	0.2(18)	5.0(17)	0.4(10)
C15A	23.1(14)	22.4(13)	22.8(15)	2.0(12)	0.0(11)	0.2(10)
C16B	24.5(8)	32.4(10)	39.8(10)	5.3(8)	7.4(7)	4.1(7)
C17B	26.2(9)	34.4(11)	46.2(11)	-3.2(8)	13.5(8)	-3.4(8)
C18B	29.3(9)	35.5(11)	49.0(12)	-5.6(9)	14.5(8)	-1.0(8)
C19B	38.9(11)	39.7(11)	40.5(11)	-4.2(9)	9.7(8)	4.2(9)
C20B	37.2(11)	36.8(11)	50.7(12)	4.1(10)	18.3(9)	1.3(9)
C21B	52.3(13)	41.7(13)	57.5(13)	1.7(11)	30.9(11)	1.2(11)

Table S24 Bond Lengths for CCDC_2556736.

Atom	Atom	Length/Å	Atom	Atom	Length/Å
O1A	C13A	1.366(9)	N1B	C7B	1.442(13)
O1A	C16B	1.437(9)	N2B	C10B	1.435(11)
N1A	N2A	1.263(3)	C1B	C2B	1.507(18)
N1A	C10A	1.422(3)	C2B	C3B	1.528(10)
N2A	C7A	1.430(4)	C2B	C4B	1.525(12)
C1A	C2A	1.539(10)	C4B	C5B	1.344(13)
C2A	C3A	1.512(5)	C4B	C9B	1.403(13)
C2A	C4A	1.518(4)	C5B	C6B	1.365(13)

Table S24 Bond Lengths for CCDC_2556736.

Atom	Atom	Length/Å	Atom	Atom	Length/Å
C4A	C5A	1.396(6)	C6B	C7B	1.363(13)
C4A	C9A	1.403(6)	C7B	C8B	1.360(12)
C5A	C6A	1.391(6)	C8B	C9B	1.378(13)
C6A	C7A	1.389(6)	C10B	C11B	1.356(11)
C7A	C8A	1.392(6)	C10B	C15B	1.399(12)
C8A	C9A	1.400(6)	C11B	C12B	1.358(18)
C10A	C11A	1.389(5)	C12B	C13B	1.327(18)
C10A	C15A	1.398(5)	C13B	C14B	1.49(2)
C11A	C12A	1.398(7)	C14B	C15B	1.381(18)
C12A	C13A	1.420(8)	C16B	C17B	1.517(3)
C13A	C14A	1.362(8)	C17B	C18B	1.516(3)
C14A	C15A	1.378(6)	C18B	C19B	1.531(3)
O1B	C13B	1.34(3)	C19B	C20B	1.519(3)
O1B	C16B	1.46(3)	C20B	C21B	1.533(3)
N1B	N2B	1.254(9)			

Table S25 Bond Angles for CCDC_2556736.

Atom	Atom	Atom	Angle/˚	Atom	Atom	Atom	Angle/˚
C13A	O1A	C16B	119.2(7)	C1B	C2B	C3B	105.8(8)
N2A	N1A	C10A	114.2(3)	C1B	C2B	C4B	111.9(8)
N1A	N2A	C7A	113.0(3)	C4B	C2B	C3B	112.5(6)
C3A	C2A	C1A	111.5(5)	C5B	C4B	C2B	125.6(10)
C3A	C2A	C4A	112.0(3)	C5B	C4B	C9B	114.3(12)
C4A	C2A	C1A	110.2(5)	C9B	C4B	C2B	120.1(10)
C5A	C4A	C2A	122.6(4)	C4B	C5B	C6B	123.8(14)
C5A	C4A	C9A	118.8(3)	C7B	C6B	C5B	120.3(13)
C9A	C4A	C2A	118.6(4)	C6B	C7B	N1B	123.1(10)
C6A	C5A	C4A	121.3(4)	C8B	C7B	N1B	117.5(9)

Table S25 Bond Angles for CCDC_2556736.

Atom	Atom	Atom	Angle/˚	Atom	Atom	Atom	Angle/˚
C7A	C6A	C5A	119.4(4)	C8B	C7B	C6B	119.3(12)
C6A	C7A	N2A	115.7(4)	C7B	C8B	C9B	118.3(11)
C6A	C7A	C8A	120.3(4)	C8B	C9B	C4B	123.7(11)
C8A	C7A	N2A	124.0(4)	C11B	C10B	N2B	115.2(8)
C7A	C8A	C9A	120.2(4)	C11B	C10B	C15B	120.6(7)
C8A	C9A	C4A	119.9(3)	C15B	C10B	N2B	124.1(8)
C11A	C10A	N1A	126.2(3)	C10B	C11B	C12B	122.2(9)
C11A	C10A	C15A	119.6(2)	C13B	C12B	C11B	119.0(12)
C15A	C10A	N1A	114.3(3)	O1B	C13B	C14B	112.1(15)
C10A	C11A	C12A	120.1(3)	C12B	C13B	O1B	125.2(17)
C11A	C12A	C13A	119.5(5)	C12B	C13B	C14B	122.6(13)
O1A	C13A	C12A	123.6(6)	C15B	C14B	C13B	115.2(13)
C14A	C13A	O1A	117.4(6)	C14B	C15B	C10B	120.3(11)
C14A	C13A	C12A	119.0(5)	O1A	C16B	C17B	106.1(3)
C13A	C14A	C15A	121.9(5)	O1B	C16B	C17B	111.3(8)
C14A	C15A	C10A	119.8(4)	C18B	C17B	C16B	113.64(17)
C13B	O1B	C16B	116.0(17)	C17B	C18B	C19B	114.58(17)
N2B	N1B	C7B	112.4(7)	C20B	C19B	C18B	114.31(19)
N1B	N2B	C10B	115.7(7)	C19B	C20B	C21B	111.1(2)

Table S26 Torsion Angles for CCDC_2556736.

A	B	C	D	Angle/˚	A	B	C	D	Angle/˚
O1A	C13A	C14A	C15A	-179.4(6)	N1B	C7B	C8B	C9B	176.4(8)
O1A	C16B	C17B	C18B	-66.0(4)	N2B	N1B	C7B	C6B	9.5(11)
N1A	N2A	C7A	C6A	169.2(3)	N2B	N1B	C7B	C8B	-172.9(8)
N1A	N2A	C7A	C8A	-9.3(4)	N2B	C10B	C11B	C12B	179.9(10)
N1A	C10A	C11A	C12A	-179.3(3)	N2B	C10B	C15B	C14B	-179.8(11)
N1A	C10A	C15A	C14A	179.3(3)	C1B	C2B	C4B	C5B	114.3(12)

Table S26 Torsion Angles for CCDC_2556736.

A	B	C	D	Angle/˚	A	B	C	D	Angle/˚
N2A	N1A	C10A	C11A	10.0(4)	C1B	C2B	C4B	C9B	-68.6(10)
N2A	N1A	C10A	C15A	-170.5(2)	C2B	C4B	C5B	C6B	177.5(9)
N2A	C7A	C8A	C9A	176.9(3)	C2B	C4B	C9B	C8B	-177.6(9)
C1A	C2A	C4A	C5A	65.9(6)	C3B	C2B	C4B	C5B	-126.7(11)
C1A	C2A	C4A	C9A	-114.8(5)	C3B	C2B	C4B	C9B	50.4(11)
C2A	C4A	C5A	C6A	177.2(3)	C4B	C5B	C6B	C7B	-3.2(18)
C2A	C4A	C9A	C8A	-176.9(3)	C5B	C4B	C9B	C8B	-0.2(15)
C3A	C2A	C4A	C5A	-58.8(4)	C5B	C6B	C7B	N1B	-176.4(9)
C3A	C2A	C4A	C9A	120.5(4)	C5B	C6B	C7B	C8B	6.1(16)
C4A	C5A	C6A	C7A	0.0(6)	C6B	C7B	C8B	C9B	-5.9(15)
C5A	C4A	C9A	C8A	2.5(5)	C7B	N1B	N2B	C10B	179.5(6)
C5A	C6A	C7A	N2A	-176.7(3)	C7B	C8B	C9B	C4B	3.1(16)
C5A	C6A	C7A	C8A	1.9(5)	C9B	C4B	C5B	C6B	0.2(17)
C6A	C7A	C8A	C9A	-1.6(5)	C10B	C11B	C12B	C13B	0.4(19)
C7A	C8A	C9A	C4A	-0.6(5)	C11B	C10B	C15B	C14B	-0.5(15)
C9A	C4A	C5A	C6A	-2.2(5)	C11B	C12B	C13B	O1B	177.0(16)
C10A	N1A	N2A	C7A	-178.8(2)	C11B	C12B	C13B	C14B	-1(2)
C10A	C11A	C12A	C13A	-0.5(7)	C12B	C13B	C14B	C15B	1(2)
C11A	C10A	C15A	C14A	-1.2(5)	C13B	O1B	C16B	C17B	-178.4(14)
C11A	C12A	C13A	O1A	179.4(6)	C13B	C14B	C15B	C10B	-0.4(19)
C11A	C12A	C13A	C14A	-0.2(8)	C15B	C10B	C11B	C12B	0.6(14)
C12A	C13A	C14A	C15A	0.2(8)	C16B	O1A	C13A	C12A	-3.5(11)
C13A	O1A	C16B	C17B	-175.9(6)	C16B	O1A	C13A	C14A	176.1(6)
C13A	C14A	C15A	C10A	0.5(7)	C16B	O1B	C13B	C12B	-3(3)
C15A	C10A	C11A	C12A	1.2(5)	C16B	O1B	C13B	C14B	176.0(14)
O1B	C13B	C14B	C15B	-177.1(15)	C16B	C17B	C18B	C19B	170.26(18)
O1B	C16B	C17B	C18B	-68.2(10)	C17B	C18B	C19B	C20B	58.5(3)
N1B	N2B	C10B	C11B	173.4(7)	C18B	C19B	C20B	C21B	174.6(2)

Table S26 Torsion Angles for CCDC_2556736.

A	B	C	D	Angle/˚	A	B	C	D	Angle/˚
N1B	N2B	C10B	C15B	-7.4(9)					

Table S27 Hydrogen Atom Coordinates (Å×10^4) and Isotropic Displacement Parameters (Å^2×10^3) for CCDC_2556736.

Atom	*x*	*y*	*z*	U(eq)
H1AA	-3953.65	5705.22	9684.36	108
H1AB	-5691.85	6532	9495.54	108
H1AC	-4186.63	7521.6	9136.3	108
H2A	-5720.68	5966.2	7934.58	40
H3AA	-5677.01	2913.19	7887.86	64
H3AB	-6419.41	3522.42	8810.95	64
H3AC	-4624.76	2832.52	8846.29	64
H5A	-1943.74	3997.64	8783.22	34
H6A	332.17	3985.02	7971.91	31
H8A	-2002.33	6837.13	5944.22	35
H9A	-4285.72	6873.55	6765.07	34
H11A	3441.86	4085.91	5970.88	30
H12A	5601.41	4008.4	5049.48	33
H14A	3073.06	6885.65	3118.69	29
H15A	938.61	6972.1	4020.51	27
H1BA	-4952.2	7345.35	8834.82	38
H1BB	-3742.55	6763.42	9678.12	38
H1BC	-5565.1	6170.86	9644.36	38
H2B	-3845.97	3856.19	9217.33	40(20)
H3BA	-6257.99	4946.04	7841.2	20(16)
H3BB	-6620.13	3957.65	8768.65	30(20)
H3BC	-5685.02	2957.44	8012.17	100(50)
H5B	-1228.6	3724.73	8638.15	40(20)
H6B	536.6	4265.22	7563.61	80(40)

Table S27 Hydrogen Atom Coordinates (Å×10[4]) and Isotropic Displacement Parameters (Å[2]×10[3]) for CCDC_2556736.

**Atom**	***x***	***y***	***z***	**U(eq)**
H8B	-2709.92	7200.91	6123.38	30(20)
H9B	-4468	6631.62	7223.6	100(50)
H11B	3961.25	3670	6054.59	28
H12B	5870.15	3748.45	5017	40(20)
H14B	2925.05	6891.77	3339.11	110(60)
H15B	1032.56	6741.2	4444.92	60(30)
H16A	7111.35	3427.75	3778.2	38
H16B	7667.34	5235.37	4278.51	38
H16C	7108.51	3423.71	3759.71	38
H16D	7717.73	5181.37	4279.96	38
H17A	8343.31	6370.59	2828.6	42
H17B	9407.57	4705.56	3144.09	42
H18A	6666.07	4383.48	1888.8	45
H18B	7964.61	2887.62	2122.57	45
H19A	8309.15	4099.13	672.58	47
H19B	8494.14	6025.41	1106.1	47
H20A	10740.44	3269.6	1530.12	49
H20B	10942.68	5268.1	1868.16	49
H21A	10964.18	4124.87	-1.06	74
H21B	12461.37	4901.73	607.33	74
H21C	11000.92	6157.63	285.96	74

Table S28 Atomic Occupancy for CCDC_2556736.

**Atom**	***Occupancy***	**Atom**	***Occupancy***	**Atom**	***Occupancy***
O1A	0.709(5)	N1A	0.709(5)	N2A	0.709(5)
C1A	0.709(5)	H1AA	0.709(5)	H1AB	0.709(5)
H1AC	0.709(5)	C2A	0.709(5)	H2A	0.709(5)
C3A	0.709(5)	H3AA	0.709(5)	H3AB	0.709(5)

Table S28 Atomic Occupancy for CCDC_2556736.

**Atom**	***Occupancy***	**Atom**	***Occupancy***	**Atom**	***Occupancy***
H3AC	0.709(5)	C4A	0.709(5)	C5A	0.709(5)
H5A	0.709(5)	C6A	0.709(5)	H6A	0.709(5)
C7A	0.709(5)	C8A	0.709(5)	H8A	0.709(5)
C9A	0.709(5)	H9A	0.709(5)	C10A	0.709(5)
C11A	0.709(5)	H11A	0.709(5)	C12A	0.709(5)
H12A	0.709(5)	C13A	0.709(5)	C14A	0.709(5)
H14A	0.709(5)	C15A	0.709(5)	H15A	0.709(5)
O1B	0.291(5)	N1B	0.291(5)	N2B	0.291(5)
C1B	0.291(5)	H1BA	0.291(5)	H1BB	0.291(5)
H1BC	0.291(5)	C2B	0.291(5)	H2B	0.291(5)
C3B	0.291(5)	H3BA	0.291(5)	H3BB	0.291(5)
H3BC	0.291(5)	C4B	0.291(5)	C5B	0.291(5)
H5B	0.291(5)	C6B	0.291(5)	H6B	0.291(5)
C7B	0.291(5)	C8B	0.291(5)	H8B	0.291(5)
C9B	0.291(5)	H9B	0.291(5)	C10B	0.291(5)
C11B	0.291(5)	H11B	0.291(5)	C12B	0.291(5)
H12B	0.291(5)	C13B	0.291(5)	C14B	0.291(5)
H14B	0.291(5)	C15B	0.291(5)	H15B	0.291(5)
H16A	0.709(5)	H16B	0.709(5)	H16C	0.291(5)
H16D	0.291(5)				

XRD of 1-(4-*tert*-butylphenyl)-2-[4-(hexoxy)phenyl]diazene (4m)

Table S29 Crystal data and structure refinement for CCDC_2559134.

Identification code	CCDC_2559134
Empirical formula	$C_{22}H_{30}N_2O$
Formula weight	338.48
Temperature/K	100(2)
Crystal system	monoclinic
Space group	$P2_1$
a/Å	8.752(3)
b/Å	7.6632(16)
c/Å	14.798(4)
α/°	90
β/°	91.747(6)
γ/°	90
Volume/Å^3	992.0(5)
Z	2
ρ_{calc}g/cm^3	1.133
μ/mm^{-1}	0.069
F(000)	368.0
Crystal size/mm^3	0.427 × 0.24 × 0.21
Radiation	MoKα (λ = 0.71073)
2Θ range for data collection/°	4.656 to 55.746
Index ranges	-11 ≤ h ≤ 11, -10 ≤ k ≤ 10, -19 ≤ l ≤ 19
Reflections collected	39867
Independent reflections	4703 [R_{int} = 0.0444, R_{sigma} = 0.0245]
Data/restraints/parameters	4703/1432/495
Goodness-of-fit on F^2	1.034
Final R indexes [I>=2σ (I)]	R_1 = 0.0316, wR_2 = 0.0882
Final R indexes [all data]	R_1 = 0.0337, wR_2 = 0.0911
Largest diff. peak/hole / e Å^{-3}	0.11/-0.12
Flack parameter	-0.1(3)

Table S30 Atomic Coordinates (×10[4]) and Equivalent Isotropic Displacement Parameters (Å^2×10^3) for CCDC_2559134. U_{eq} is defined as 1/3 of the trace of the orthogonalised U_{IJ} tensor.

Atom	*x*	*y*	*z*	U(eq)
O1	4427(6)	5885(8)	6623(4)	29.5(7)
N1	10051(2)	5983(2)	4141.7(14)	32.3(4)
N2	8804(2)	5217(2)	3995.7(15)	29.1(4)
C1	13420(9)	5569(15)	2124(6)	33.0(14)
C2	11967(8)	4801(13)	2002(4)	33.5(10)
C3	10822(4)	4894(4)	2632(3)	28.4(7)
C4	11106(4)	5782(4)	3434(2)	29.0(5)
C5	12512(4)	6561(5)	3586(2)	40.3(7)
C6	13636(6)	6450(8)	2947(4)	40.3(12)
C7	7738(4)	5410(5)	4697(3)	25.5(7)
C8	6353(5)	4574(9)	4552(4)	30.3(8)
C9	5199(9)	4709(10)	5174(4)	29.0(9)
C10	5502(9)	5681(14)	5972(5)	28.6(15)
C11	6880(10)	6524(16)	6123(7)	30.8(12)
C12	7998(6)	6397(7)	5483(3)	28.9(9)
C13	14701(16)	5272(17)	1461(9)	37.6(14)
C14	14138(9)	5898(14)	544(5)	58.6(18)
C15	16138(8)	6260(17)	1754(7)	76(3)
C16	15084(13)	3321(9)	1401(7)	64(2)
C13B	14660(30)	5390(30)	1390(18)	37.6(14)
C14B	14070(20)	5170(30)	428(9)	39(3)
C15B	15670(30)	6990(30)	1446(11)	67(4)
C16B	15540(30)	3740(40)	1651(11)	76(5)
C17	2981(3)	5019(3)	6503.5(17)	28.8(5)
C18	2013(2)	5491(3)	7294.1(13)	27.2(4)
C19	630(2)	4283(3)	7339.6(12)	29.6(4)
C20	-502(3)	4761(3)	8066.2(14)	31.4(5)

Table S30 Atomic Coordinates (×10[4]) and Equivalent Isotropic Displacement Parameters (Å[2]×10[3]) for CCDC_2559134. U_{eq} is defined as 1/3 of the trace of the orthogonalised U_{IJ} tensor.

**Atom**	***x***	***y***	***z***	**U(eq)**
C21	191(6)	4796(6)	9024(3)	43.4(9)
C22	-1091(8)	4949(14)	9714(4)	62(2)
O1A	4159(12)	5620(18)	6485(8)	32.6(17)
N1A	9362(5)	5183(5)	3654(3)	32.9(9)
N2A	9426(5)	5799(5)	4453(3)	31.8(8)
C1A	13250(17)	5440(30)	2111(10)	28(3)
C2A	11916(14)	4680(20)	1785(7)	29.4(19)
C3A	10657(8)	4656(10)	2310(5)	32.0(14)
C4A	10745(7)	5337(8)	3172(4)	26.5(11)
C5A	12066(9)	6069(8)	3497(4)	32.0(12)
C6A	13345(12)	6141(16)	2967(8)	39(2)
C7A	8039(10)	5694(11)	4935(5)	26.8(16)
C8A	6776(11)	4780(20)	4622(9)	37(2)
C9A	5471(18)	4620(30)	5139(11)	43(4)
C10A	5370(19)	5540(30)	5933(12)	25(3)
C11A	6690(20)	6420(40)	6242(15)	33(3)
C12A	7998(15)	6490(18)	5768(6)	34.8(18)
C13A	14627(15)	5656(17)	1517(8)	37(2)
C14A	16063(6)	4813(12)	1947(4)	52.0(15)
C15A	15022(8)	7602(9)	1323(5)	47.5(15)
C16A	14328(14)	4803(18)	599(7)	55(3)
C17A	2730(6)	4928(9)	6123(4)	40.2(12)
C18A	1468(5)	5406(7)	6727(3)	44.0(11)
C19A	1561(5)	4635(6)	7652(3)	38.9(11)
C20A	190(8)	5081(9)	8234(4)	51.2(14)
C21A	180(15)	4189(13)	9093(8)	49(2)
C22A	-955(14)	4830(30)	9763(9)	60(5)

Table S31 Anisotropic Displacement Parameters (Å^2×10^3) for CCDC_2559134. The Anisotropic displacement factor exponent takes the form: -2π^2[h^{2}a*^{2}U$_{11}$+2hka*b*U$_{12}$+…].

Atom	U_{11}	U_{22}	U_{33}	U_{23}	U_{13}	U_{12}
O1	26.1(13)	31.7(18)	31.1(16)	-6.0(10)	9.2(11)	-4.8(10)
N1	28.9(9)	35.9(10)	32.5(9)	-5.9(7)	6.9(8)	-6.6(7)
N2	29.7(9)	29.2(8)	28.6(9)	-1.6(7)	4.9(8)	-3.6(7)
C1	27.5(18)	35(2)	37(2)	3.1(16)	2.9(13)	-9.4(15)
C2	37.2(16)	34.4(17)	29(2)	-7.7(19)	1.2(15)	-4.2(11)
C3	24.6(10)	29.3(13)	31(2)	-2.4(14)	0.4(13)	-6.9(8)
C4	29.2(15)	32.5(12)	25.5(13)	-7.4(10)	5.0(10)	-6.6(10)
C5	38.6(16)	49.2(19)	33.6(12)	-16.5(12)	8.7(11)	-13.9(12)
C6	33.8(17)	51(3)	36.1(15)	-13.0(15)	11.3(11)	-15.3(14)
C7	26.6(17)	25.2(15)	24.9(16)	-4.5(12)	5.6(12)	-2.8(12)
C8	30(2)	33.2(15)	27.4(15)	-5.3(10)	3.9(15)	-5.8(15)
C9	25.7(18)	31.4(16)	30.1(15)	-2.3(12)	6.6(11)	0.1(14)
C10	33(3)	29(3)	24.0(18)	-3.2(16)	8.3(18)	2(2)
C11	28(2)	31.5(16)	33(3)	-5.5(19)	3.7(15)	-1.6(15)
C12	25.4(11)	30.3(15)	31(2)	-4.3(19)	6.8(16)	-2.5(9)
C13	31.3(18)	54(3)	28(2)	-7.9(17)	13.6(14)	-8.0(17)
C14	71(3)	62(4)	45(3)	17(3)	28(2)	15(3)
C15	47(3)	116(7)	66(4)	-37(4)	29(3)	-27(3)
C16	82(5)	58(3)	53(5)	15(2)	39(3)	25(3)
C13B	31.3(18)	54(3)	28(2)	-7.9(17)	13.6(14)	-8.0(17)
C14B	54(6)	47(8)	18(4)	-3(5)	3(3)	22(6)
C15B	61(9)	95(10)	47(7)	-19(7)	25(6)	-42(7)
C16B	77(11)	117(12)	34(7)	26(8)	29(6)	51(9)
C17	26.0(10)	29.9(10)	30.6(12)	0.5(10)	2.7(9)	-0.9(8)
C18	25.6(8)	24.5(8)	32.0(9)	-0.3(7)	6.9(7)	-0.1(7)
C19	23.3(9)	33.6(9)	32.3(9)	-3.8(7)	4.6(7)	-2.8(7)

Table S31 Anisotropic Displacement Parameters (Å^2×10^3) for CCDC_2559134. The Anisotropic displacement factor exponent takes the form: -2π^2[h^{2}a*^{2}U$_{11}$+2hka*b*U$_{12}$+…].

Atom	U_{11}	U_{22}	U_{33}	U_{23}	U_{13}	U_{12}
C20	23.9(9)	36.2(11)	34.2(10)	-2.1(8)	2.8(8)	-0.5(9)
C21	35.5(13)	56(3)	38.9(16)	3.2(18)	4.9(10)	9.9(19)
C22	65(4)	82(4)	40(3)	8(2)	26(2)	8(3)
O1A	34(4)	31(4)	33(3)	-5(2)	6(2)	-1(3)
N1A	34.1(18)	37(2)	27.7(18)	0.2(15)	6.0(16)	-3.7(15)
N2A	31.9(18)	31.3(17)	32.3(19)	1.0(16)	3.7(18)	-0.8(15)
C1A	29(4)	34(5)	21(4)	-3(3)	17(3)	-3(3)
C2A	23(2)	34(3)	32(5)	-7(4)	5(3)	-8(2)
C3A	32(3)	34(3)	30(3)	-2(3)	1(2)	-8.7(19)
C4A	34(3)	28(3)	17(3)	-7.1(19)	-2.1(19)	-5.0(19)
C5A	40(4)	27(3)	29(2)	-2.7(18)	0(2)	-11(2)
C6A	40(4)	35(4)	41(3)	9(2)	-6(3)	-8(3)
C7A	30(3)	25(3)	26(4)	-6(3)	3(2)	-5(2)
C8A	42(6)	45(5)	23(3)	-10(3)	4(4)	-2(4)
C9A	34(6)	48(5)	48(5)	-19(3)	0(3)	-3(4)
C10A	17(3)	22(4)	36(5)	10(3)	0(2)	-6(3)
C11A	29(5)	38(5)	33(5)	-3(4)	4(3)	8(3)
C12A	41(3)	33(3)	31(4)	-10(4)	4(3)	5(2)
C13A	37(4)	50(5)	24(3)	5(3)	3(3)	8(3)
C14A	34(2)	74(4)	49(3)	23(3)	18(2)	17(3)
C15A	43(3)	55(3)	45(3)	21(2)	9(3)	-11(2)
C16A	53(5)	71(6)	41(5)	-5(4)	14(3)	9(4)
C17A	28(2)	53(3)	40(3)	3(3)	-1(2)	-1.5(19)
C18A	32.6(19)	50(3)	49(3)	-1(2)	2.5(18)	2.5(19)
C19A	30(2)	37(2)	49(2)	-3(2)	3.5(19)	1.8(18)
C20A	44(3)	57(4)	53(3)	4(3)	12(3)	11(3)
C21A	48(3)	57(5)	44(4)	2(4)	13(2)	3(5)

Table S31 Anisotropic Displacement Parameters (Å^2×10^3) for CCDC_2559134. The Anisotropic displacement factor exponent takes the form: -2π^2[h^{2}a*^{2}U$_{11}$+2hka*b*U$_{12}$+…].

Atom	U_{11}	U_{22}	U_{33}	U_{23}	U_{13}	U_{12}
C22A	23(3)	111(12)	45(5)	-13(6)	-7(3)	6(4)

Table S32 Bond Lengths for CCDC_2559134.

Atom	Atom	Length/Å	Atom	Atom	Length/Å
O1	C10	1.376(7)	C21	C22	1.544(7)
O1	C17	1.435(4)	O1A	C10A	1.359(15)
N1	N2	1.252(3)	O1A	C17A	1.447(8)
N1	C4	1.426(3)	N1A	N2A	1.273(6)
N2	C7	1.424(5)	N1A	C4A	1.429(7)
C1	C2	1.408(8)	N2A	C7A	1.429(10)
C1	C6	1.401(8)	C1A	C2A	1.379(15)
C1	C13	1.530(15)	C1A	C6A	1.375(14)
C2	C3	1.391(6)	C1A	C13A	1.521(15)
C3	C4	1.384(4)	C2A	C3A	1.368(13)
C4	C5	1.380(4)	C3A	C4A	1.378(7)
C5	C6	1.387(5)	C4A	C5A	1.359(7)
C7	C8	1.382(4)	C5A	C6A	1.387(11)
C7	C12	1.401(4)	C7A	C8A	1.376(9)
C8	C9	1.391(6)	C7A	C12A	1.377(8)
C9	C10	1.415(8)	C8A	C9A	1.400(14)
C10	C11	1.380(8)	C9A	C10A	1.377(16)
C11	C12	1.385(7)	C10A	C11A	1.398(16)
C13	C14	1.508(12)	C11A	C12A	1.363(14)
C13	C15	1.519(12)	C13A	C14A	1.534(12)
C13	C16	1.536(12)	C13A	C15A	1.559(13)
C13B	C14B	1.508(19)	C13A	C16A	1.523(14)

Table S32 Bond Lengths for CCDC_2559134.

Atom	Atom	Length/Å	Atom	Atom	Length/Å
C13B	C15B	1.516(19)	C17A	C18A	1.488(7)
C13B	C16B	1.524(19)	C18A	C19A	1.492(7)
C17	C18	1.509(3)	C19A	C20A	1.537(6)
C18	C19	1.528(2)	C20A	C21A	1.443(11)
C19	C20	1.528(3)	C21A	C22A	1.507(15)
C20	C21	1.525(5)			

Table S33 Torsion Angles for CCDC_2559134.

Atom	Atom	Atom	Angle/˚	Atom	Atom	Atom	Angle/˚
C10	O1	C17	118.7(5)	C20	C21	C22	109.8(4)
N2	N1	C4	113.8(3)	C10A	O1A	C17A	116.3(10)
N1	N2	C7	114.4(3)	N2A	N1A	C4A	114.8(5)
C2	C1	C13	122.2(7)	N1A	N2A	C7A	115.4(5)
C6	C1	C2	114.2(5)	C2A	C1A	C13A	121.3(10)
C6	C1	C13	123.2(7)	C6A	C1A	C2A	120.9(10)
C3	C2	C1	124.0(5)	C6A	C1A	C13A	117.7(12)
C4	C3	C2	119.0(4)	C3A	C2A	C1A	119.7(9)
C3	C4	N1	125.4(3)	C2A	C3A	C4A	119.7(6)
C5	C4	N1	115.3(3)	C3A	C4A	N1A	114.0(6)
C5	C4	C3	119.2(3)	C5A	C4A	N1A	125.6(6)
C4	C5	C6	120.6(3)	C5A	C4A	C3A	120.5(5)
C5	C6	C1	122.8(5)	C4A	C5A	C6A	120.6(7)
C8	C7	N2	115.4(4)	C1A	C6A	C5A	118.6(9)
C8	C7	C12	119.9(4)	C8A	C7A	N2A	123.2(7)
C12	C7	N2	124.6(3)	C8A	C7A	C12A	118.9(10)
C7	C8	C9	120.9(6)	C12A	C7A	N2A	117.9(8)
C8	C9	C10	118.1(6)	C7A	C8A	C9A	121.5(11)
O1	C10	C9	122.0(6)	C10A	C9A	C8A	119.6(13)
O1	C10	C11	116.5(6)	O1A	C10A	C9A	127.3(14)

Table S33 Torsion Angles for CCDC_2559134.

Atom	Atom	Atom	Angle/˚	Atom	Atom	Atom	Angle/˚
C11	C10	C9	121.5(6)	O1A	C10A	C11A	115.7(13)
C10	C11	C12	119.3(7)	C9A	C10A	C11A	116.9(14)
C11	C12	C7	120.3(5)	C12A	C11A	C10A	123.3(16)
C1	C13	C16	110.3(10)	C11A	C12A	C7A	119.2(13)
C14	C13	C1	107.7(10)	C1A	C13A	C14A	111.7(11)
C14	C13	C15	110.1(10)	C1A	C13A	C15A	113.1(12)
C14	C13	C16	108.9(9)	C1A	C13A	C16A	110.6(11)
C15	C13	C1	111.1(9)	C14A	C13A	C15A	107.2(9)
C15	C13	C16	108.8(9)	C16A	C13A	C14A	107.8(10)
C14B	C13B	C15B	108.9(18)	C16A	C13A	C15A	106.2(9)
C14B	C13B	C16B	107.8(17)	O1A		C17A C18A	109.7(6)
C15B	C13B	C16B	111(2)	C17A	C18A	C19A	115.6(4)
O1	C17	C18	107.9(3)	C18A	C19A	C20A	113.9(4)
C17	C18	C19	110.67(16)	C21A	C20A	C19A	114.4(6)
C18	C19	C20	114.68(16)	C20A	C21A	C22A	116.6(10)
C21	C20	C19	114.2(3)				

Table S34 Hydrogen Atom Coordinates (Å×10^4) and Isotropic Displacement Parameters (Å^2×10^3) for CCDC_2559134.

Atom	*x*	*y*	*z*	U(eq)
H2	11755.33	4182.02	1456.65	40
H3	9858.74	4354.87	2513.45	34
H5	12712.81	7178.3	4133.54	48
H6	14593.39	6996.76	3073.29	48
H8	6185.95	3898.03	4019.48	36
H9	4235.14	4164.91	5066.21	35
H11	7059.42	7184.84	6658.48	37

Table S34 Hydrogen Atom Coordinates (Å×10⁴) and Isotropic Displacement Parameters (Å²×10³) for CCDC_2559134.

Atom	*x*	*y*	*z*	U(eq)
H12	8946.53	6981.54	5578.74	35
H14A	14948.92	5752.42	107.41	88
H14B	13858.62	7133.96	579.99	88
H14C	13241.53	5215.01	347.6	88
H15A	16924.06	6093.69	1304.24	114
H15B	16513.68	5817.53	2340.98	114
H15C	15902.31	7505.46	1805.67	114
H16A	15357.73	2876.83	2005.51	95
H16B	15945.36	3155.7	1002.19	95
H16C	14191.3	2684.45	1157.33	95
H14G	14930.89	5084.23	25.26	59
H14H	13439	6186.79	254.86	59
H14I	13445.59	4112.41	379.76	59
H15G	16472.72	6896.97	999.97	101
H15H	16143.71	7074.64	2053.86	101
H15I	15056.54	8036.77	1321.32	101
H16G	16250.98	3453.73	1174.11	113
H16H	14824.08	2776.55	1727.26	113
H16I	16117.16	3942.95	2220.07	113
H17A	3131.43	3739.44	6479.34	35
H17B	2469.35	5397.19	5930.51	35
H18A	1662.62	6714.7	7231.13	33
H18B	2635.17	5396.44	7862.16	33
H19A	996.77	3078.55	7452.18	36
H19B	82.96	4292.12	6743.97	36
H20A	-938.03	5924.68	7924.63	38
H20B	-1352.36	3908.44	8043.47	38

Table S34 Hydrogen Atom Coordinates (Å×10^4) and Isotropic Displacement Parameters (Å^2×10^3) for CCDC_2559134.

Atom	*x*	*y*	*z*	U(eq)
H21A	896.22	5801.23	9092.38	52
H21B	784.46	3714.43	9139.84	52
H22A	-1853.81	4033.2	9596.07	93
H22B	-1579.92	6095.36	9654.97	93
H22C	-654.71	4813.84	10327.39	93
H2A	11871.14	4183.09	1197.41	35
H3A	9724.58	4167.27	2081.91	38
H5A	12112.7	6535.6	4091.62	38
H6A	14266.53	6659.99	3191.25	47
H8A	6790.75	4249.38	4042.77	44
H9A	4658.47	3871.8	4943.9	52
H11A	6669.97	6987.57	6811.69	40
H12A	8872.3	7082.61	6010.08	42
H14D	16895.88	4859.18	1519.7	78
H14E	15846.3	3593.57	2096.63	78
H14F	16364.77	5446.71	2499.26	78
H15D	15918.51	7656.97	942.95	71
H15E	15244.76	8207.78	1894.93	71
H15F	14151.17	8162.63	1008.57	71
H16D	13438.48	5352.87	297.95	82
H16E	14126.56	3555.86	681.99	82
H16F	15225.12	4950.81	226.4	82
H17C	2799.77	3641.68	6074.17	48
H17D	2519.74	5406.42	5510.26	48
H18C	487.95	5042.37	6432.19	53
H18D	1445.12	6693.1	6784.91	53
H19C	2506.96	5056.48	7965.29	47

Table S34 Hydrogen Atom Coordinates (Å×10[4]) and Isotropic Displacement Parameters (Å[2]×10[3]) for CCDC_2559134.

Atom	*x*	*y*	*z*	U(eq)
H19D	1638.32	3350.99	7598.79	47
H20C	184.84	6354.27	8345.92	61
H20D	-762.4	4787.48	7888.57	61
H21C	1215.58	4277.48	9376.73	59
H21D	-23.12	2936.59	8975.87	59
H22D	-1762.68	5492.3	9445.65	90
H22E	-433.19	5577.54	10211.2	90
H22F	-1406.72	3825.9	10068.98	90

Table S35 Atomic Occupancy for CCDC_2559134.

Atom	*Occupancy*	Atom	*Occupancy*	Atom	*Occupancy*
O1	0.675(3)	N1	0.675(3)	N2	0.675(3)
C1	0.675(3)	C2	0.675(3)	H2	0.675(3)
C3	0.675(3)	H3	0.675(3)	C4	0.675(3)
C5	0.675(3)	H5	0.675(3)	C6	0.675(3)
H6	0.675(3)	C7	0.675(3)	C8	0.675(3)
H8	0.675(3)	C9	0.675(3)	H9	0.675(3)
C10	0.675(3)	C11	0.675(3)	H11	0.675(3)
C12	0.675(3)	H12	0.675(3)	C13	0.455(13)
C14	0.455(13)	H14A	0.455(13)	H14B	0.455(13)
H14C	0.455(13)	C15	0.455(13)	H15A	0.455(13)
H15B	0.455(13)	H15C	0.455(13)	C16	0.455(13)
H16A	0.455(13)	H16B	0.455(13)	H16C	0.455(13)
C13B	0.219(12)	C14B	0.219(12)	H14G	0.219(12)
H14H	0.219(12)	H14I	0.219(12)	C15B	0.219(12)
H15G	0.219(12)	H15H	0.219(12)	H15I	0.219(12)
C16B	0.219(12)	H16G	0.219(12)	H16H	0.219(12)

Table S35 Atomic Occupancy for CCDC_2559134.

Atom	*Occupancy*	Atom	*Occupancy*	Atom	*Occupancy*
H16I	0.219(12)	C17	0.675(3)	H17A	0.675(3)
H17B	0.675(3)	C18	0.675(3)	H18A	0.675(3)
H18B	0.675(3)	C19	0.675(3)	H19A	0.675(3)
H19B	0.675(3)	C20	0.675(3)	H20A	0.675(3)
H20B	0.675(3)	C21	0.675(3)	H21A	0.675(3)
H21B	0.675(3)	C22	0.675(3)	H22A	0.675(3)
H22B	0.675(3)	H22C	0.675(3)	O1A	0.325(3)
N1A	0.325(3)	N2A	0.325(3)	C1A	0.325(3)
C2A	0.325(3)	H2A	0.325(3)	C3A	0.325(3)
H3A	0.325(3)	C4A	0.325(3)	C5A	0.325(3)
H5A	0.325(3)	C6A	0.325(3)	H6A	0.325(3)
C7A	0.325(3)	C8A	0.325(3)	H8A	0.325(3)
C9A	0.325(3)	H9A	0.325(3)	C10A	0.325(3)
C11A	0.325(3)	H11A	0.325(3)	C12A	0.325(3)
H12A	0.325(3)	C13A	0.325(3)	C14A	0.325(3)
H14D	0.325(3)	H14E	0.325(3)	H14F	0.325(3)
C15A	0.325(3)	H15D	0.325(3)	H15E	0.325(3)
H15F	0.325(3)	C16A	0.325(3)	H16D	0.325(3)
H16E	0.325(3)	H16F	0.325(3)	C17A	0.325(3)
H17C	0.325(3)	H17D	0.325(3)	C18A	0.325(3)
H18C	0.325(3)	H18D	0.325(3)	C19A	0.325(3)
H19C	0.325(3)	H19D	0.325(3)	C20A	0.325(3)
H20C	0.325(3)	H20D	0.325(3)	C21A	0.325(3)
H21C	0.325(3)	H21D	0.325(3)	C22A	0.325(3)
H22D	0.325(3)	H22E	0.325(3)	H22F	0.325(3)

11. References

[1] P.-Y. Chen, C.-W. Hsu, T.-I. Ho and J.-H. Ho, "The selective synthesis of N-arylbenzene-1,2-diamines or 1-arylbenzimidazoles by irradiating 4-methoxy-4'-substituted-azobenzenes in different solvents," *RSC advances* **11** (2021): 6662.
[2] D. Bahulayan, L. John and M. Lalithambika, "Modified Clays as Efficient Acid–Base Catalyst Systems for Diazotization and Diazocoupling Reactions," *Synthetic Communications* **33** (2003): 863.
[3] C. Zheng, M. Ju, X. Zhang, K. Xie, A. Hou and A. Gao, "A multi-stimulus responsive nanogel actuator with the π-π stacking interaction self-assembly and application for bioinspired smart skin with light, heat and humidity responses and superb UV resistance," *Chemical Engineering Journal* **499** (2024): 156092.
[4] H. Akiyama and N. Tamaoki, "Polymers derived from N‐isopropylacrylamide and azobenzene‐containing acrylamides: Photoresponsive affinity to water," *Journal of Polymer Science Part A: Polymer Chemistry* **42** (2004): 5200.
[5] O. Renier, G. Bousrez and K. Stappert et al., "Photoisomerization and Mesophase Formation in Azo-Ionic Liquids," *Crystal Growth & Design* **20** (2020): 214.
[6] B. Kou, J. Zhang, X. Huai, X. Liang and S.-J. Xiao, "Light-driven reversible strand displacement using glycerol azobenzene inserted DNA," *RSC advances* **5** (2015): 5055.
[7] X. Yi and C. Xi, "Iodine-catalyzed aerobic oxidation of o-alkylazoarenes to 2H-indazoles," *Tetrahedron* **73** (2017): 1311.
[8] J. Maciejewski, A. Sobczuk and A. Claveau et al., "Photochromic Torsional Switch (PTS): a light-driven actuator for the dynamic tuning of π-conjugation extension," *Chemical science* **8** (2017): 361.
[9] A. Gamet, I. Ciss and A. Courtois et al., "Total Synthesis of Photoswitchable Latrunculin B Enables Reversible Control of Actin Polymerization and Cell Migration," *Journal of the American Chemical Society* **148** (2026): 15474.
[10] H. Chi, K. Y. Mya, T. Lin, C. He, F. Wang and W. S. Chin, "Thermally stable azobenzene dyes through hybridization with POSS," *New Journal of Chemistry* **37** (2013): 735.
[11] L. Krause, R. Herbst-Irmer, G. M. Sheldrick and D. Stalke, "Comparison of silver and molybdenum microfocus X-ray sources for single-crystal structure determination," *Journal of applied crystallography* **48** (2015): 3.
[12] G. M. Sheldrick, "SHELXT - integrated space-group and crystal-structure determination," *Acta Crystallographica Section A: Foundations and Advances* **71** (2015): 3.
[13] G. M. Sheldrick, "Crystal structure refinement with SHELXL," *Acta Crystallographica Section C: Structural Chemistry* **71** (2015): 3.
